# Isocampholenic Acid Derivatives as Potential Fragrances

**DOI:** 10.3390/molecules30183794

**Published:** 2025-09-18

**Authors:** Luka Ciber, Tjaša Koželj, Kaja Prosenak, Nejc Petek, Franc Požgan, Jurij Svete, Bogdan Štefane, Uroš Grošelj

**Affiliations:** Faculty of Chemistry and Chemical Technology, University of Ljubljana, Večna Pot 113, SI-1000 Ljubljana, Slovenia; luka.ciber@fkkt.uni-lj.si (L.C.); kozelj.tjasa97@gmail.com (T.K.); kaja.prosenak@gmail.com (K.P.); nejc.petek@fkkt.uni-lj.si (N.P.); franc.pozgan@fkkt.uni-lj.si (F.P.); jurij.svete@fkkt.uni-lj.si (J.S.); bogdan.stefane@fkkt.uni-lj.si (B.Š.)

**Keywords:** isocampholenic acid, α-campholenic acid, DFT calculation, odor properties, esters, ethers, enones, ketones, alcohols, cyclopropanation

## Abstract

(+)-Isocampholenic acid, prepared in two steps from (1*S*)-(+)-10-camphorsulfonic acid in 60% yield (on a scale of 172 mmol), served as the starting compound for the synthesis of small libraries of isocampholenic acid derivatives, comprising a total of 60 compounds, which are of interest due to their olfactory properties. Although isocampholenic acid derivatives are thermodynamically up to 5.9 kcal/mol less stable than endocyclic alkene isomers according to DFT calculation, only minor (up to 2%) isomerization to α-campholenic acid isomers was observed in most cases. The products were fully characterized, and their odor properties were preliminarily assessed by untrained laypersons. This study represents the first systematic exploration of the chemical space of isocampholenic acid. Novel derivatives with distinct odor profiles were discovered, and promising directions for future functionalization for fragrance development were identified.

## 1. Introduction

Biologically, the recognition of volatile molecules plays a crucial role in communication across all species, as the environment is rich in odorous substances released by various sources. Since ancient times, humans have derived pleasure and functional uses from naturally occurring fragrances and spices. Odors consist mainly of hydrophobic volatile organic compounds with molecular weights below 300 Da. These include many chemical classes such as organic acids, alcohols, aldehydes, ethers, esters, amides, amines, hydrocarbons, halogenated hydrocarbons, ketones, nitriles, aromatics, phenols, and other nitrogen- and sulfur-containing compounds including heterocycles [1,2]. Since the 2004 Nobel Prize in Medicine was awarded to Richard Axel and Linda B. Buck “for their discoveries of odorant receptors and the organization of the olfactory system”, we have begun to understand the mechanism of odor perception in terms of the molecular receptors, which will arguably enable the rational design and synthesis of molecules with desired olfactory properties in the future [3,4,5,6,7,8,9].

**Figure 1:**
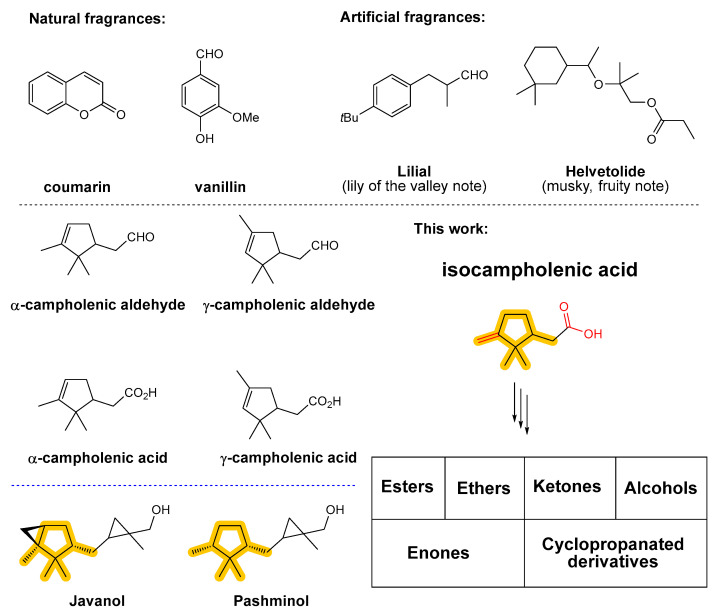
(**top**) Selected natural and synthetic fragrance molecules. (**bottom**) Isocampholenic acid derivatives with yet unknown odor properties and commercial representatives (Javanol and Pashminol), derived from campholenic acid/aldehyde.

Aesthetically appealing odorant substances (fragrances) are now found as ingredients in countless detergents and especially in beauty and personal care products such as cosmetics and perfumes, as well as in other formulations. The idea that only natural ingredients are used in such compositions, for example, in perfumes, is a common misconception. Chemists first synthesized naturally occurring fragrance molecules such as coumarin, vanillin and others, and then fragrances that do not occur in nature, such as the “lily of the valley” olfactory note embodied by Lilial and the synthetic musk note of Helvetolide, which enabled the development of many new sensory impressions (Figure 1, top). Nowadays, the development of perfumes and cosmetics is strongly driven by innovations in synthetic fragrances with unprecedented scents and superior physical properties, including human and environmental safety and biodegradability [10,11,12,13,14,15,16]. Furthermore, given the current production and use of fragrances of all kinds, it would be impossible to rely solely on the availability of natural sources [1,2,17,18]. In order to meet the requirements, new synthetic fragrance molecules with the desired scents and physical properties are constantly being developed.

Terpene-based fragrances, both of natural and synthetic origin, play a key role in the Flavor and Fragrance industry. Derivatives based on α- and γ-campholenic acid/aldehyde have been intensively studied and have resulted in compounds with unique odor profiles [19,20,21,22,23], such as the sandalwood character of Javanol and Pashminol (developed by Givaudan). On this basis, we expect that the compounds prepared from isomeric isocampholenic acid (Figure 1, bottom) [24,25,26], which have not yet been systematically explored, represent potentially interesting new fragrance molecules. A structure and reaction search of isocampholenic acid/aldehyde revealed no studies in the direction of a systematic investigation of isocampholenic acid/aldehyde derivatives as odorants. The availability of isocampholenic acid from camphorsulfonic acid [25] in both enantiomeric forms provides a solid platform for systematically exploring the chemical space of isocampholenic acid, the obvious points of diversification being the carboxylic acid and the exocyclic C=C bond (Figure 1, bottom). We report here on the synthesis of several small libraries of novel isocampholenic acid derivatives and the tentative evaluation of their odor properties (odor profile) by laypersons in the field.

## 2. Results and Discussion

**Scheme 1:**
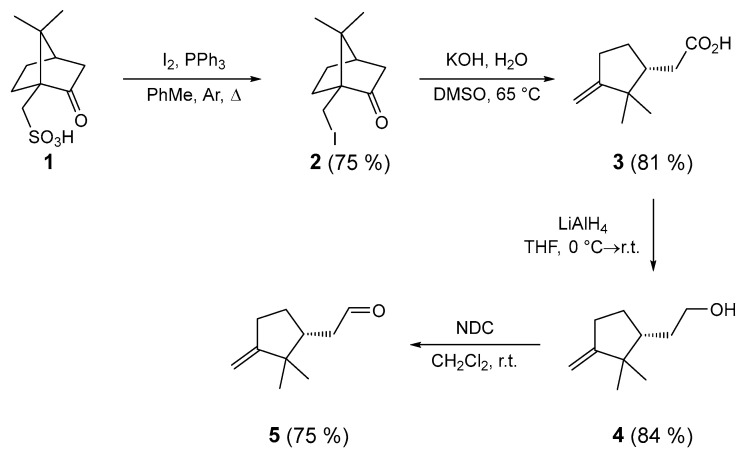
Synthesis of the starting building blocks **3**–**5** from (1*S*)-(+)-10-camphorsulfonic acid (**1**).

*Synthesis*. The synthesis of the starting building blocks **3**–**5** began with commercially available camphorsulfonic acid, which is available in both enantiomeric forms. Starting from (1*S*)-(+)-10-camphorsulfonic acid (**1**) on a 172 mmol scale, (*R*)-2-(2,2-dimethyl-3-methylenecyclopentyl)acetic acid ((+)-isocampholenic acid) (**3**) was prepared in two steps in 60% yield (Scheme 1). First, sulfonic acid **1** was converted to 10-iodocamphor (**2**) [27] by Appel-type transformation with PPh_3_/I_2_ in refluxing toluene, followed by Grob fragmentation with KOH in DMSO/H_2_O to give (+)-isocampholenic acid (**3**) [25]. Alcohol **4** [28] was obtained in 84% yield by reduction of acid **3** with LiAlH_4_ in anhydrous THF, while oxidation of alcohol **4** with freshly prepared 3-carboxypyridinium dichromate (NDC) [29,30] in anhydrous dichloromethane gave aldehyde **5** [31] in 75% yield (Scheme 1).

**Scheme 2:**
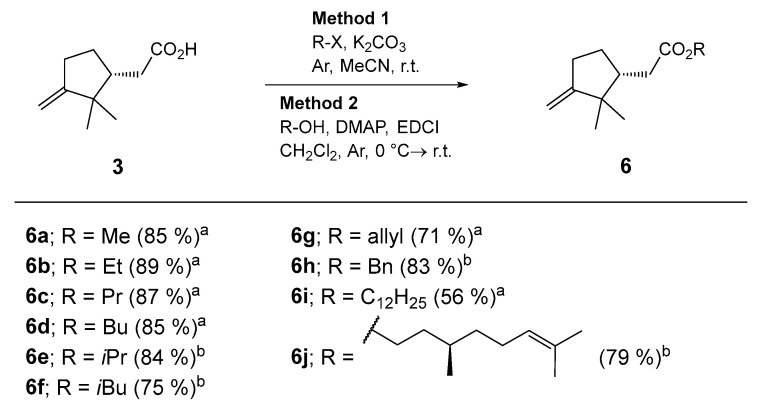
Synthesis of the esters **6a**–**j** from (+)-isocampholenic acid (**3**), ^a^ Method 1, ^b^ Method 2.

The scalability and repeatability of the synthesis of the starting building blocks **3**–**5** enabled the synthesis of the desired compound libraries. First, a library of esters was prepared from (+)-isocampholenic acid (**3**) (Scheme 2). Esters **6a**–**j** were prepared either from aliphatic halides in the presence of K_2_CO_3_ (Method 1) or by coupling alcohols with EDCI-activated acid **3** using catalytic amounts of DMAP (Method 2). Moderate to high yields (56–89%) of the corresponding esters **6** were obtained with both methods. In all cases, the products were isolated by column chromatography. Of the synthesized esters, only methyl **6a** [32], ethyl **6b** [28] and allyl ester **6g** [33] have been described in the literature.

**Scheme 3:**
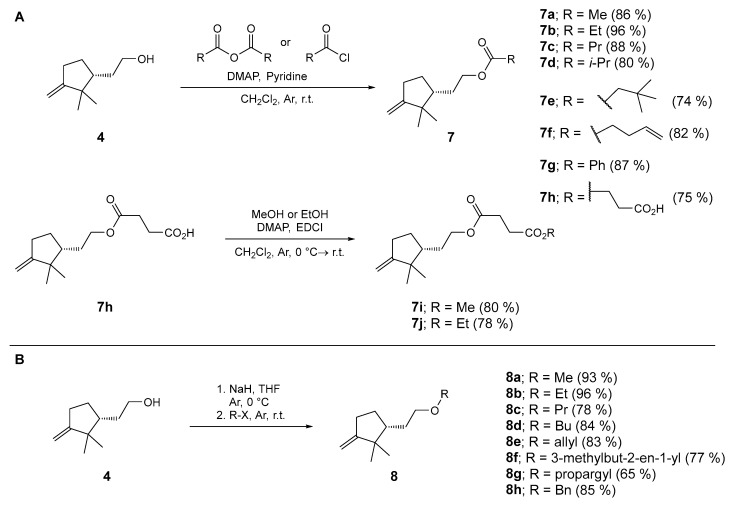
(**A**) Synthesis of the esters **7a**–**i** from alcohol **4**. (**B**) Synthesis of the ethers **8a**–**h** from alcohol **4**.

**Scheme 4:**
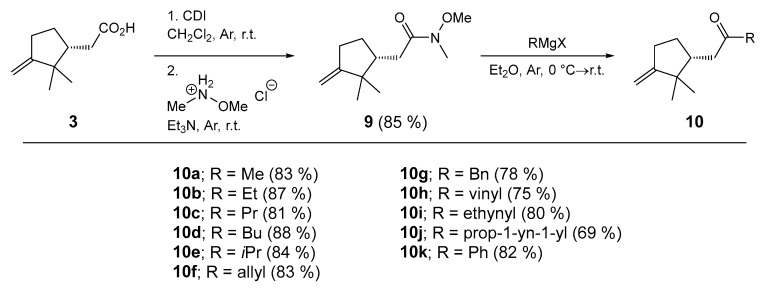
Synthesis of ketones **10a**–**k** from Weinreb amide **9**.

In contrast, esters **7a**–**h** were prepared in similar yields (74–96%) from alcohol **4** and the corresponding acid anhydrides or chloride in the presence of pyridine and DMAP (Scheme 3A). The EDCI-activated succinic anhydride-derived acid ester **7h** was converted with methanol and ethanol to the corresponding di-esters **7i** and **7j** in 80% and 78% yield, respectively (Scheme 3A). A series of ethers **8a**–**h** was then targeted and isolated in 65–96% yield, starting from alcohol **4**, which was first deprotonated with NaH, followed by the S_N_2 reaction with primary aliphatic halides (Scheme 3B).

(+)-Isocampholenic acid (**3**) was used for the synthesis of the library of ketones (Scheme 4). First, the corresponding Weinreb amide **9** was efficiently synthesized in 85% yield from the CDI-activated carboxylic acid **3**. Subsequent treatment of the Weinreb amide **9** with various Grignard reagents led to the formation of the corresponding ketones **10a**–**k** in 69–88% yield. Only ethyl ketone **10b**, previously reported as prepared by thermal isomerization of the substituted isoborneol, has been described in the literature [34].

**Scheme 5:**
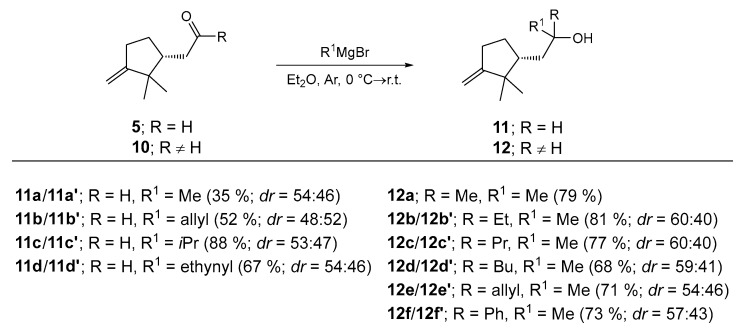
Synthesis of secondary alcohols **11a**–**d** and tertiary alcohols **12a**–**f** from aldehyde **5** and ketones **10a**–**d**,**f**,**k**, respectively. The diastereomeric ratio reflects the order of column chromatography elution of the individual diastereomers of compounds **11a**,**b**,**d** (elute first)/**11a′**,**b′**,**d′** (elute second).

Secondary alcohols **11a**–**d**/**11a′**–**d′** (35–88% yield) and tertiary alcohols **12a**–**f**/**12a′**–**f′** (64–81% yield) were prepared from aldehyde **5** and ketones **10**, respectively, by addition of selected Grignard reagents (Scheme 5). The formation of a new stereocenter of compounds **11a**–**d**/**11a′**–**d′** and **12a**–**f**/**12a′**–**f′** proceeded with low diastereoselectivity close to unity (diastereomeric ratio in the range of 60:40 to 51:49). For the secondary alcohols **11a**,**b**,**d**/**11a′**,**b′**,**d′**, the two diastereomers could be partially separated, i.e., the first eluting diastereomer of compounds **11a**,**b**,**d**, which was isolated by column chromatography, could be isolated in diastereomerically pure form in 20–27% yield. The secondary alcohol **11c**/**11c′** and the tertiary alcohols **12a**–**f**/**12a′**–**f′** could not be separated by column chromatography and were characterized as mixtures.

**Scheme 6:**
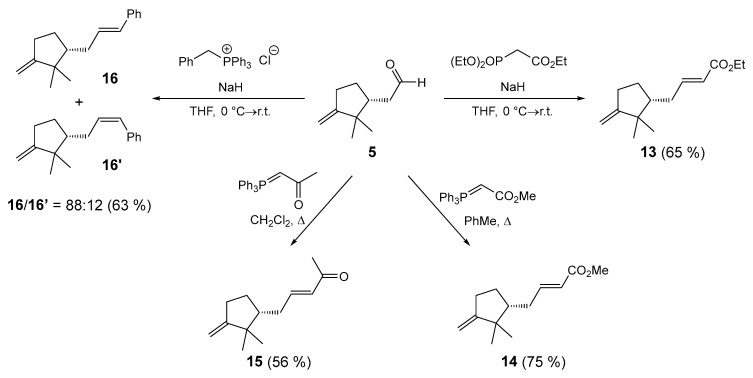
Horner–Wadsworth–Emmons and Wittig reactions of aldehyde **5**. Synthesis of functionalized alkenes **13**–**16**.

The concluding transformations on the carboxyl side of isocampholenic acid were olefinations using the Horner–Wadsworth–Emmons and Wittig reactions. For this purpose, the aldehyde **5** was reacted with triethyl phosphonoacetate using NaH at 0 °C, methyl(triphenylphosphoranylidene)acetate, triphenylphosphoranyliden-2-propanon and benzyltriphenylphosphonium chloride using NaH at 0 °C. In all cases, the corresponding functionalized alkenes **13**–**16** were obtained in 56–75% yield. Compounds **13**–**15** were isolated as a single (*E*)-diastereomers (vicinal H-C=C-H coupling ^3^*J* = 15.6–15.9 Hz), while alkene **16** was isolated as a mixture of the major (*E*)- and the minor (*Z*)-isomers in a ratio of **16**:**16′** = 88:12 (vicinal H-C=C-H coupling ^3^*J*(**16**) = 15.7 Hz, ^3^*J*(**16′**) = 11.7 Hz) (Scheme 6).

**Scheme 7:**
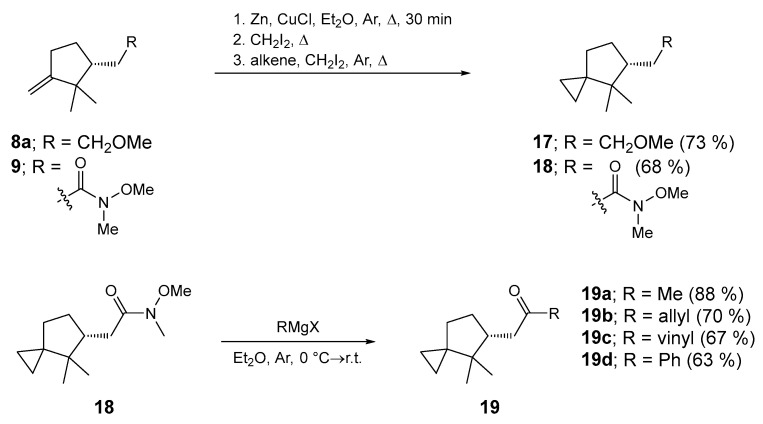
Cyclopropanation of ether **8a** and Weinreb amide **9**. Synthesis of ketones **19a**–**d** from cyclopropanated Weinreb amide **18**.

The functionalization of the exocyclic C=C bond of selected isocampholenic acid derivatives was subsequently explored. Alkene functionality offers numerous opportunities and challenges for both classical and modern functional group transformations [35,36,37,38], aiming to expand the diversity of compound libraries. The reasons to preferentially address the cyclopropanation of the exocyclic C=C bond are both the lability of the exocyclic C=C bond (vide infra) and the odor profile that could be favorably affected by this transformation as demonstrated in the α-campholenic acid series, i.e., Javanol [23]. The Simmons–Smith reaction with diiodomethane and a zinc-copper couple was used for cyclopropanation [39]. Ether **8a** and Weinreb amide **9** were used as model substrates. Despite prolonged reaction times and excess reagents, complete conversion could not be achieved. Thus, even after repeated purification by column chromatography, the desired products **17** and **18** could not be isolated in pure form; they were isolated in 73% and 68% yield, respectively, contaminated with 8% and 5% of the starting alkene (Scheme 7). The cyclopropanated Weinreb amide **18** allowed subsequent synthesis of a series of ketones via the reactions with selected Grignard reagents. In this way, the cyclopropanated ketones **19a**–**d** were prepared in 63–88% yield (Scheme 7). The unsaturated ketone **19c** was isolated in pure form, while the products **19a**,**b**,**d** contained up to 4% of byproducts derived from alkene **9**. See Supplementary Materials for details.

**Scheme 8:**
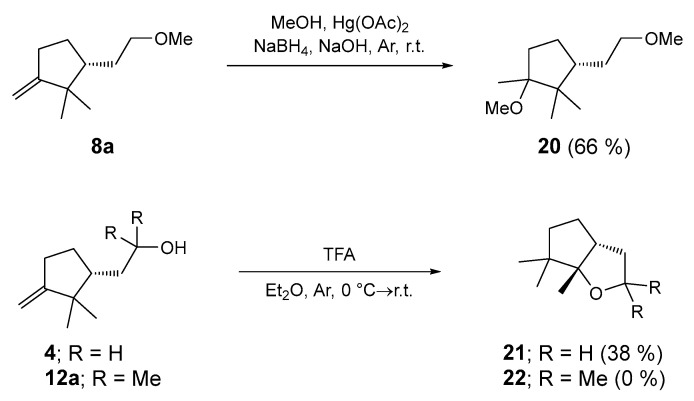
Oxymercuration of ether **8a** in anhydrous methanol and TFA-catalyzed rearrangement-cyclization of alcohol **4**.

The oxymercuration of ether **8a** in anhydrous methanol gave ether **20** in 66% yield as a single stereoisomer following column chromatography (Scheme 8). The absolute configuration of the newly formed stereogenic center was not determined. Finally, treatment of the alcohol **4** with trifluoroacetic acid gave the bicyclic product **21** [40] in 38% yield. It was formed by an acid-catalyzed rearrangement followed by cyclization [25]. All attempts to prepare a similar bicyclic product **22** from the tertiary alcohol **12a** did not yield the desired product (no alkene formation was observed), presumably due to steric hindrance in the product (Scheme 8).

*Structure determination.* The isolated products **6**–**20** were characterized by ^1^H- and ^13^C-NMR, IR and HRMS, with the exception of the HRMS data for compounds **7e**, **8a**–**e**,**g**, **10i**, **16**/**16′**, **17** and **20**, since these compounds did not ionize. The diastereomers of compounds **11c**/**11c′**, **12b**–**f**/**12b′**–**f′** and **16**/**16′**, which could not be separated by column chromatography, were characterized as diastereomeric mixtures. The absolute configuration of the newly formed stereogenic center of compounds **11**/**11′**, **12b**–**f**/**12b′**–**f′** and **20** was not determined.

**Scheme 9:**
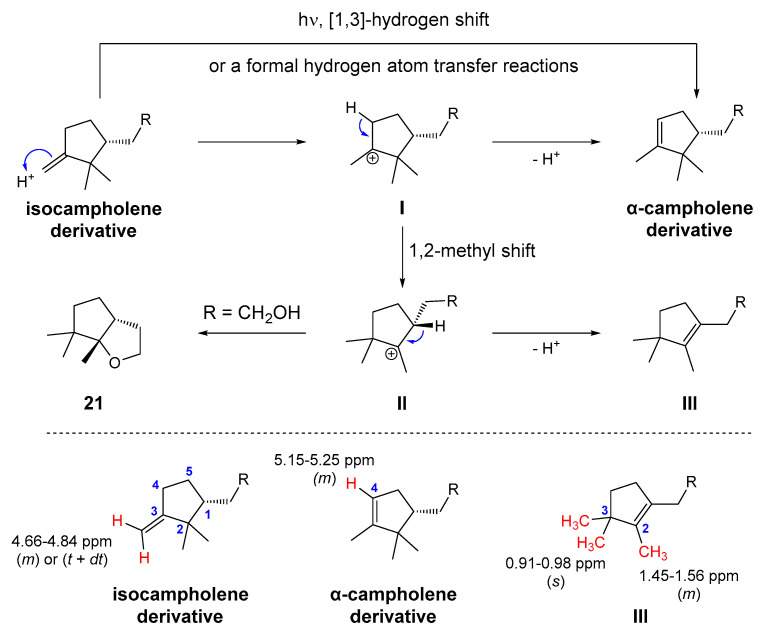
Plausible mechanisms for the isomerization of an exocyclic C=C bond of isocampholenic acid derivatives (**top**). Typical proton chemical shifts of three types of isomeric compounds measured in CDCl_3_ (**bottom**).

*Exocyclic C=C bond isomerization.* According to DFT calculations, isocampholenic acid (**3**), with the exocyclic C=C group, is thermodynamically up to 5.9 kcal/mol less stable than its endocyclic isomers (see Figure S1 in the Supplementary Materials for details). Consequently, the isocampholenic acid derivatives **3**–**16** are susceptible to isomerization of the methylene group via different mechanisms. In the presence of a strong Brønsted acid, the following transformations are expected (Scheme 9): (i) isomerization to α-campholenic derivatives via tertiary carbocation **I** or (ii) the 1,2-methyl shift of **I** and formation of tertiary carbocation **II** followed by ring closure in the presence of a suitably tethered nucleophile (formation of product **21**) or deprotonation of **II** to form the tetrasubstituted alkene **III**, as reported in our earlier report (Scheme 9) [25]. In the present study, strongly Brønsted acidic conditions were avoided during synthesis to prevent acid-catalyzed isomerization. Another plausible mechanism for the isomerization of an exocyclic C=C bond is the pericyclic photochemical [1,3]-hydrogen shift leading to α-campholene derivatives. Similar results would be obtained in (homolytic) redox processes, i.e., formal hydrogen atom transfer reactions, that could be either organo- [41,42] and metal-catalyzed [43,44,45,46,47,48,49] or part of photoredox-catalyzed processes (Scheme 9) [50,51].

α-Campholenic acid and isocampholenic acid derivatives can be easily distinguished by their proton spectra due to the olefinic protons. Data obtained exclusively in deuterated chloroform are compared. In the isocampholenic acid derivatives **3**–**16**, the geminal methylene protons typically appear at 4.66–4.84 ppm either as a multiplet or, if well resolved, as a separate triplet (4.79 ppm, *J* = 2.5 Hz) and doublet of triplet (4.81 ppm, *J* = 0.7; 2.2 Hz), as exemplified for aldehyde **5**. In the α-campholenic acid isomers, the vinylic C(4)-proton appears as a multiplet at 5.15–5.25 ppm [52,53,54,55,56,57,58]. Type **III** isomers, without olefinic protons, are characterized by one C(2)-Me group appearing as a broad singlet or multiplet at 1.45–1.56 ppm and the two geminal C(3)-Me groups appearing as a singlet at 0.91–0.98 ppm [25,59,60,61], making it difficult to distinguish them from the other isomers based on the proton spectra alone (Scheme 9). See Figure S2 in the Supplementary Materials for details. A closer look at the proton spectra of the synthesized isocampholenic acid derivatives **3**–**16** shows that up to 2% of α-campholene isomers are formed, with the exception of esters **6e** (8%), **6h** (12%) and **14** (4%) and ketone **10a** (3%) where up to 12% of the α-campholene isomer was observed. The starting building blocks, compounds **3**–**5**, contain up to 1% of the α-campholene isomer (for detail, see Table S1 in the Supplementary Materials).

**Figure 2:**
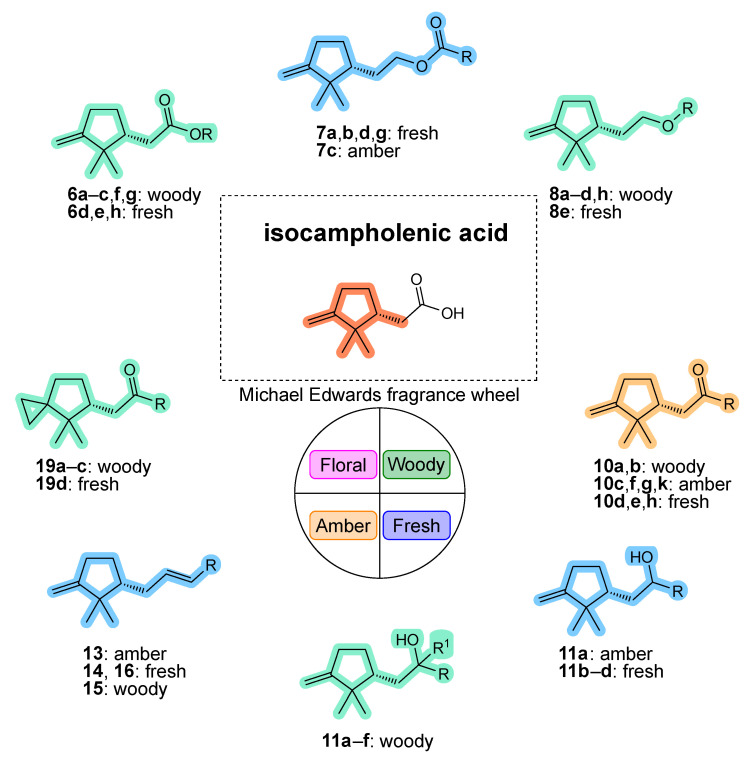
Odor profile distribution of isocampholenic acid derivatives according to the Michael Edwards fragrance wheel.

*Olfactory properties.* The odor profiles of the selected products were determined by an untrained panel of five laypersons, with the average description shown in the Supplementary Materials in Table S1. The amounts of α-campholenic acid-derived isomer impurities or, in the case of cyclopropanated products **17**–**19**, the unreacted alkenes, are also included in Table S1, as determined by proton NMR. The samples were evaluated as neat oils. No dilution experiments or threshold determinations were performed, i.e., methods combining instrumental analysis (GC-MS) with human olfactory perception [62,63,64]. The average odor profiles determined are therefore only a rough description. Figure 2 shows the odor profile distribution (odor notes) of the synthesized isocampholenic acid derivatives according to the Michael Edwards fragrance wheel [65].

## 3. Materials and Methods

Solvents for extractions and chromatography were of technical grade and were distilled prior to use. Extracts were dried over technical grade anhydrous Na_2_SO_4_. The NMR spectra were obtained on a Bruker Avance DPX 300 at 300 MHz for ^1^H and 75.5 MHz for ^13^C nucleus, and on a Bruker UltraShield 500 plus (Bruker, Billerica, MA, USA) at 500 MHz for ^1^H and 126 MHz for ^13^C nucleus, using CDCl_3_ with TMS as the internal standard, as solvent. Mass spectra were recorded on an Agilent 6224 Accurate Mass TOF LC/MS (Agilent Technologies, Santa Clara, CA, USA), and IR spectra on a Perkin-Elmer Spectrum BX FTIR spectrophotometer (PerkinElmer, Waltham, MA, USA). Column chromatography (CC) was performed on silica gel (Silica gel 60, particle size: 0.035–0.070 mm (Sigma-Aldrich, St. Louis, MO, USA)). All the commercially available chemicals used were purchased from Sigma-Aldrich (St. Louis, MO, USA). 3-Carboxypyridinium dichromate (NDC) was freshly prepared and used following the procedure described in the literature [29,30].

### 3.1. Synthesis of (1R,4R)-1-(iodomethyl)-7,7-dimethylbicyclo[2.2.1]heptan-2-one (***2***) [27]

Compound **2** was prepared according to the procedure described in the literature [27]. To a solution of (1*S*)-(+)-10-camphorsulfonic acid (**1**) (1.0 equiv., 172 mmol, 39.95 g) in anhydrous toluene (250 mL) under argon, PPh_3_ (4.0 equiv., 688 mmol, 180.46 g) and I_2_ (2.0 equiv., 344 mmol, 87.31 g) were added. The resulting reaction mixture was heated for 16 h under reflux. The volatile components were evaporated in vacuo. The residue was dissolved in EtOAc (500 mL) and washed with Na_2_SO_3_ (aq. sat., 5 × 100 mL or until the reaction mixture changed color from dark violet to light yellow) and NaCl (aq. sat., 3 × 50 mL). The organic phase was dried under anhydrous Na_2_SO_4_, filtered, and the volatile components were evaporated in vacuo. The solid residue was suspended in petroleum ether (or hexane) (500 mL) and stirred for 12 h at room temperature. The solid residue was filtered, washed with petroleum ether (or hexane) (2 × 50 mL), and the volatile components (filtrate) were evaporated in vacuo to obtain 10-iodocamphor **2**. Yield: 37.77 g (ω = 0.95, 129 mmol, 75%) of yellowish solid. ^1^H-NMR (500 MHz, CDCl_3_): *δ* 0.90 (*s*, 3H), 1.08 (*s*, 3H), 1.35–1.43 (*m*, 1H), 1.57–1.64 (*m*, 1H), 1.91 (*d*, *J* = 18.4 Hz, 1H), 1.95–2.05 (*m*, 2H), 2.16 (*t*, *J* = 4.2 Hz, 1H), 2.40 (*dd*, *J* = 4.9, 18.4 Hz, 1H), 3.12 (*d*, *J* = 10.6 Hz, 1H), 3.31 (*d*, *J* = 10.6 Hz, 1H). ^13^C-NMR (126 MHz, CDCl_3_): *δ* 0.8, 20.3, 20.5, 26.9, 30.7, 43.1, 44.2, 48.4, 59.2, 215.1.

### 3.2. Synthesis of (R)-2-(2,2-dimethyl-3-methylenecyclopentyl)acetic Acid (***3***) [25]

Compound **3** was prepared according to the procedure described in the literature [25]. To a solution of 10-iodocamphor (**2**) (1.0 equiv., ω = 0.95, 42.9 mmol, 12.56 g) in a mixture of DMSO (120 mL) and H_2_O (40 mL) was added KOH (5.0 equiv., 214.5 mmol, 12.04 g). The resulting reaction mixture was stirred at 65 °C for 2 h. NaCl (aq. sat., 100 mL) was added to the cooled reaction mixture (room temperature), followed by extraction with Et_2_O (3 × 100 mL). The organic phase was discarded, and the aqueous phase was carefully acidified with HCl (6 M in H_2_O, 180 mmol, 30 mL) to pH 1–2, followed by extraction with EtOAc (3 × 100 mL). The combined organic phase was dried under anhydrous Na_2_SO_4_, filtered, and the volatiles evaporated in vacuo to give acid **3**. Product **3** was stored under argon in the absence of light. Yield: 5.85 g (34.75 mmol, 81%) of yellowish oil. ^1^H-NMR (500 MHz, CDCl_3_): *δ* 0.85 (*s*, 3H), 1.08 (*s*, 3H), 1.32–1.44 (*m*, 1H), 1.91–2.05 (*m*, 2H), 2.15 (*dd*, *J* = 10.4, 14.9 Hz, 1H), 2.28–2.39 (*m*, 1H), 2.40–2.52 (*m*, 2H), 4.78 (*s*, 1H), 4.80 (*s*, 1H), 11.34 (br *s*, 1H). ^13^C-NMR (126 MHz, CDCl_3_): *δ* 23.5, 26.7, 28.4, 30.4, 35.2, 43.9, 46.4, 103.8, 161.0, 180.5.

### 3.3. Synthesis of (R)-2-(2,2-dimethyl-3-methylenecyclopentyl)ethan-1-ol (***4***) [28]

A solution of (+)-isocampholenic acid (**3**) (1.0 equiv., 25.1 mmol, 4.22 g) in anhydrous THF (200 mL) was cooled to 0 °C under argon, followed by a careful addition of LiAlH_4_ (1 M in THF, 2.0 equiv., 50.2 mmol, 50.2 mL). The resulting reaction mixture was stirred at 0 °C for 1 h and then at room temperature for 20 h. The reaction mixture was cooled to 0 °C, and then cooled H_2_O (0 °C, 100 mL) was carefully added dropwise to quench the excess LiAlH_4_. After stirring the reaction mixture at room temperature for 15 min, most of the THF was evaporated in vacuo. The residue was extracted with Et_2_O (100 mL). The organic phase was washed with NaCl (aq. sat., 3 × 100 mL), dried under anhydrous Na_2_SO_4_, filtered, and the volatiles evaporated in vacuo to give alcohol **4**. Yield: 3.255 g (21.1 mmol, 84%) of a white solid. [*α*]_D_^r.t.^ = +21.0 (0.30, CH_2_Cl_2_). EI-HRMS: *m*/*z* = 155.1429 (MH^+^); C_10_H_19_O: requires *m*/*z* = 155.1430 (MH^+^); *ν*_max_ 3332, 3071, 2958, 2866, 1651, 1462, 1433, 1378, 1362, 1199, 1151, 1055, 1038, 999, 976, 921, 877, 850 cm^−1^. ^1^H-NMR (500 MHz, CDCl_3_): *δ* 0.83 (*s*, 3H), 1.06 (*s*, 3H), 1.24–1.32 (*m*, 1H), 1.34–1.42 (*m*, 1H), 1.54–1.62 (*m*, 1H), 1.67 (br *s*, 1H), 1.69–1.76 (*m*, 1H), 1.82–1.88 (*m*, 1H), 2.25–2.34 (*m*, 1H), 2.42–2.50 (*m*, 1H), 3.60–3.68 (*m*, 1H), 3.70–3.79 (*m*, 1H), 4.75–4.79 (*m*, 2H). ^13^C-NMR (126 MHz, CDCl_3_): *δ* 23.5, 26.6, 28.3, 30.8, 33.2, 44.0, 46.8, 62.4, 103.1, 162.4.

### 3.4. Synthesis of (R)-2-(2,2-dimethyl-3-methylenecyclopentyl)acetaldehyde (***5***) [31]

To a solution of alcohol **4** (1.0 equiv., 5.5 mmol, 848 mg) in anhydrous CH_2_Cl_2_ (15 mL) was added freshly prepared 3-carboxypyridinium dichromate (NDC) [29,30] (1.857 g, 4 mmol, 0.727 equiv.). The resulting reaction mixture was stirred at room temperature for 20 h. The reaction mixture was filtered through a short plug of Silica gel 60, and the plug was washed with CH_2_Cl_2_ (2 × 15 mL). The volatiles were evaporated in vacuo, and the residue was purified by column chromatography (Silica gel 60, petroleum ether/EtOAc = 20:1). The fractions containing the pure product **5** were combined, and the volatile components were evaporated in vacuo. Yield: 630 mg (4.14 mmol, 75%) of a colorless oil. ^1^H-NMR (500 MHz, CDCl_3_): *δ* 0.85 (*s*, 3H), 1.08 (*s*, 3H), 1.28–1.39 (*m*, 1H), 1.88–1.97 (*m*, 1H), 2.01–2.10 (*m*, 1H), 2.25 (*ddd*, *J* = 2.7, 10.3, 16.0 Hz, 1H), 2.31–2.41 (*m*, 1H), 2.44–2.54 (*m*, 2H), 4.79 (*t*, *J* = 2.5 Hz, 1H), 4.81 (*td*, *J* = 0.7, 2.2 Hz, 1H), 9.81 (*dd*, *J* = 1.8, 2.6 Hz, 1H). ^13^C-NMR (126 MHz, CDCl_3_): *δ* 23.5, 26.4, 28.7, 30.6, 43.8, 44.2, 44.9, 103.8, 160.7, 202.7.

### 3.5. Synthesis of Esters ***6*** from (+)-Isocampholenic Acid—General Procedure 1 (GP1)

To a solution of (+)-isocampholenic acid (**3**) (1.0 equiv.) in anhydrous MeCN (2 mL) under argon, K_2_CO_3_ (2.0 equiv.) and the corresponding aliphatic halide were added. The resulting reaction mixture was stirred at room temperature for 20 h. The volatiles were evaporated in vacuo, and the residue was purified by column chromatography (Silica gel 60). The fractions containing the pure product **6** were combined, and the volatiles were evaporated in vacuo. The isolated esters **6** were fully characterized.

### 3.6. Synthesis of Esters ***6*** from (+)-Isocampholenic Acid—General Procedure 2 (GP2)

To a solution of (+)-isocampholenic acid (**3**) (1.0 equiv.) in anhydrous CH_2_Cl_2_ (7 mL) under argon, the corresponding alcohol and DMAP (0.1 equiv.) were added. The reaction mixture was cooled to −5 °C, and then EDCI (2.0 equiv.) was added. The resulting reaction mixture was stirred at −5 °C for 1 h and then at room temperature for 20 h. The volatiles were evaporated in vacuo. The residue was dissolved in EtOAc (50 mL) and washed with NaHSO_4_ (aq., 1 M, 2 × 10 mL), NaHCO_3_ (aq. sat., 2 × 10 mL) and NaCl (aq. sat., 3 × 10 mL). The organic phase was dried under anhydrous Na_2_SO_4_, filtered, and the volatile components evaporated in vacuo. The residue was purified by column chromatography (Silica gel 60). The fractions containing the pure product **6** were combined, and the volatiles were evaporated in vacuo. The isolated esters **6** were fully characterized.

### 3.7. Synthesis of Methyl (R)-2-(2,2-dimethyl-3-methylenecyclopentyl)acetate (***6a***) [32]

Following *GP1*. Prepared from (+)-isocampholenic acid (**3**) (1.0 mmol, 168 mg), K_2_CO_3_ (2.0 mmol, 276 mg), MeCN (2 mL) and iodomethane (1.5 mmol, 49 μL); column chromatography: EtOAc/petroleum ether = 1:10. Yield: 154 mg (0.85 mmol, 85%) of colorless oil. [*α*]_D_^r.t.^ = +21.0 (0.16, CH_2_Cl_2_). EI-HRMS: *m*/*z* = 183.1379 (MH^+^); C_11_H_19_O_2_: requires *m*/*z* = 183.1380 (MH^+^); *ν*_max_ 2958, 1737, 1652, 1435, 1364, 1291, 1253, 1195, 1143, 1060, 999, 880 cm^−1^. ^1^H-NMR (500 MHz, CDCl_3_): *δ* 0.84 (*s*, 3H), 1.07 (*s*, 3H), 1.29–1.41 (*m*, 1H), 1.87–1.94 (*m*, 1H), 1.96–2.04 (*m*, 1H), 2.12 (*dd*, *J* = 10.5, 14.7 Hz, 1H), 2.28–2.50 (*m*, 3H), 3.68 (*s*, 3H), 4.77 (*t*, *J* = 2.5 Hz, 1H), 4.80 (*td*, *J* = 0.8, 2.2 Hz, 1H). ^13^C-NMR (126 MHz, CDCl_3_): *δ* 23.5, 26.7, 28.4, 30.5, 35.1, 43.9, 46.7, 51.6, 103.7, 161.3, 174.2.

### 3.8. Synthesis of Ethyl (R)-2-(2,2-dimethyl-3-methylenecyclopentyl)acetate (***6b***) [28]

Following *GP1*. Prepared from (+)-isocampholenic acid (**3**) (1.0 mmol, 168 mg), K_2_CO_3_ (2.0 mmol, 276 mg), MeCN (2 mL) and bromoethane (1.5 mmol, 114 μL); column chromatography: EtOAc/petroleum ether = 1:10. Yield: 174 mg (0.89 mmol, 89%) of colorless oil. [*α*]_D_^r.t.^ = +7.0 (0.30, CH_2_Cl_2_). EI-HRMS: *m*/*z* = 197.1535 (MH^+^); C_12_H_21_O_2_: requires *m*/*z* = 197.1536 (MH^+^); *ν*_max_ 2961, 1733, 1652, 1464, 1365, 1290, 1252, 1182, 1140, 1031, 879 cm^−1^. ^1^H-NMR (500 MHz, CDCl_3_): *δ* 0.84 (*s*, 3H), 1.07 (*s*, 3H), 1.26 (*t*, *J* = 7.1 Hz, 3H), 1.30–1.41 (*m*, 1H), 1.87–1.94 (*m*, 1H), 1.96–2.04 (*m*, 1H), 2.11 (*dd*, *J* = 10.5, 14.6 Hz, 1H), 2.28–2.50 (*m*, 3H), 4.14 (*q*, *J* = 7.1 Hz, 2H), 4.77 (*t*, *J* = 2.5 Hz, 1H), 4.79 (*td*, *J* = 0.7, 2.1 Hz, 1H). ^13^C-NMR (126 MHz, CDCl_3_): *δ* 14.4, 23.5, 26.7, 28.4, 30.5, 35.4, 43.9, 46.7, 60.4, 103.6, 161.4, 173.7.

### 3.9. Synthesis of Propyl (R)-2-(2,2-dimethyl-3-methylenecyclopentyl)acetate (***6c***)

Following *GP1*. Prepared from (+)-isocampholenic acid (**3**) (1.0 mmol, 168 mg), K_2_CO_3_ (2.0 mmol, 276 mg), MeCN (2 mL) and 1-bromopropane (1.2 mmol, 111 μL); column chromatography: EtOAc/petroleum ether = 1:20. Yield: 184 mg (0.87 mmol, 87%) of colorless oil. [*α*]_D_^r.t.^ = +8.0 (0.26, CH_2_Cl_2_). EI-HRMS: *m*/*z* = 211.1694 (MH^+^); C_13_H_23_O_2_: requires *m*/*z* = 211.1693 (MH^+^); *ν*_max_ 2962, 1734, 1654, 1446, 1365, 1293, 1255, 1186, 1035, 879 cm^−1^. ^1^H-NMR (500 MHz, CDCl_3_): *δ* 0.84 (*s*, 3H), 0.95 (*t*, *J* = 7.4, 3H), 1.07 (*s*, 3H), 1.31–1.41 (*m*, 1H), 1.66 (*h*, *J* = 7.2 Hz, 2H), 1.86–1.94 (*m*, 1H), 1.96–2.04 (*m*, 1H), 2.12 (*dd*, *J* = 10.5, 14.6 Hz, 1H), 2.29–2.50 (*m*, 3H), 4.04 (*t*, *J* = 6.7 Hz, 2H), 4.78 (*t*, *J* = 2.5 Hz, 1H), 4.79–4.81 (*m*, 1H). ^13^C-NMR (126 MHz, CDCl_3_): *δ* 10.6, 22.1, 23.6, 26.7, 28.4, 30.5, 35.4, 44.0, 46.7, 66.1, 103.7, 161.5, 174.0.

### 3.10. Synthesis of Butyl (R)-2-(2,2-dimethyl-3-methylenecyclopentyl)acetate (***6d***)

Following *GP1*. Prepared from (+)-isocampholenic acid (**3**) (1.0 mmol, 168 mg), K_2_CO_3_ (2.0 mmol, 276 mg), MeCN (2 mL) and 1-iodobutane (1.2 mmol, 138 μL); column chromatography: EtOAc/petroleum ether = 1:20. Yield: 192 mg (0.85 mmol, 85%) of colorless oil. [*α*]_D_^r.t.^ = +8.0 (0.29, CH_2_Cl_2_). EI-HRMS: *m*/*z* = 225.1847 (MH^+^); C_14_H_25_O_2_: requires *m*/*z* = 225.1849 (MH^+^); *ν*_max_ 2959, 1734, 1652, 1464, 1364, 1289, 1249, 1179, 1141, 1063, 1020, 879 cm^−1^. ^1^H-NMR (500 MHz, CDCl_3_): *δ* 0.84 (*s*, 3H), 0.94 (*t*, *J* = 7.3 Hz, 3H), 1.07 (*s*, 3H), 1.33–1.44 (*m*, 3H), 1.58–1.66 (*m*, 2H), 1.86–1.94 (m, 1H), 1.96–2.04 (*m*, 1H), 2.11 (*dd*, *J* = 10.5, 14.6 Hz, 1H), 2.28–2.50 (*m*, 3H), 4.08 (*t*, *J* = 6.7 Hz, 2H), 4.77 (*t*, *J* = 2.3 Hz, 1H), 4.80 (*t*, *J* = 1.8 Hz, 1H). ^13^C-NMR (126 MHz, CDCl_3_): *δ* 13.8, 19.3, 23.6, 26.7, 28.4, 30.5, 30.8, 35.4, 44.0, 46.7, 64.4, 103.7, 161.5, 173.9.

### 3.11. Synthesis of Isopropyl (R)-2-(2,2-dimethyl-3-methylenecyclopentyl)acetate (***6e***)

Following *GP2*. Prepared from (+)-isocampholenic acid (**3**) (1.0 mmol, 168 mg), propan-2-ol (2.0 mmol, 121 μL), DMAP (0.1 mmol, 12.2 mg), CH_2_Cl_2_ (7 mL) in EDCI (2.0 mmol, ω = 98%, 391 mg); column chromatography: EtOAc/petroleum ether = 1:20. Yield: 178 mg (0.84 mmol, 84%) of colorless oil. [*α*]_D_^r.t.^ = +11.0 (0.33, CH_2_Cl_2_). EI-HRMS: *m*/*z* = 211.1693 (MH^+^); C_13_H_23_O_2_: requires *m*/*z* = 211.1693 (MH^+^); *ν*_max_ 2961, 1729, 1652, 1467, 1374, 1290, 1181, 1143, 1108, 959, 879 cm^−1^. ^1^H-NMR (500 MHz, CDCl_3_): *δ* 0.83 (*s*, 3H), 1.07 (*s*, 3H), 1.24 (*dd*, *J* = 1.4, 6.3 Hz, 6H), 1.30–1.41 (*m*, 1H), 1.86–1.93 (*m*, 1H), 1.96–2.03 (*m*, 1H), 2.08 (*dd*, *J* = 10.5, 14.3 Hz, 1H), 2.29–2.39 (*m*, 2H), 2.41–2.49 (*m*, 1H), 4.77 (*t*, *J* = 2.5 Hz, 1H), 4.78–4.80 (*m*, 1H), 4.98–5.06 (*m*, 1H). ^13^C-NMR (126 MHz, CDCl_3_): *δ* 22.0, 22.0, 23.6, 26.7, 28.3, 30.5, 35.8, 44.0, 46.8, 67.6, 103.6, 161.5, 173.3.

### 3.12. Synthesis of Isobutyl (R)-2-(2,2-dimethyl-3-methylenecyclopentyl)acetate (***6f***)

Following *GP2*. Prepared from (+)-isocampholenic acid (**3**) (1.0 mmol, 168 mg), butan-2-ol (2.0 mmol, 187 μL), DMAP (0.1 mmol, 12.2 mg), CH_2_Cl_2_ (7 mL) in EDCI (2.0 mmol, ω = 98%, 391 mg); column chromatography: EtOAc/petroleum ether = 1:25. Yield: 169 mg (0.75 mmol, 75%) of colorless oil. [*α*]_D_^r.t.^ = +15.0 (0.34, CH_2_Cl_2_). EI-HRMS: *m*/*z* = 225.1849 (MH^+^); C_14_H_25_O_2_: requires *m*/*z* = 225.1849 (MH^+^); *ν*_max_ 2960, 1734, 1652, 1468, 1379, 1289, 1250, 1177, 1139, 1001, 879, 671 cm^−1^. ^1^H-NMR (500 MHz, CDCl_3_): *δ* 0.84 (*s*, 3H), 0.94 (*d*, *J* = 6.8 Hz, 6H), 1.08 (*s*, 3H), 1.30–1.43 (*m*, 1H), 1.87–2.06 (*m*, 3H), 2.12 (*dd*, *J* = 10.5, 14.6 Hz, 1H), 2.28–2.51 (*m*, 3H), 3.86 (*d*, *J* = 6.7 Hz, 2H), 4.78 (*t*, *J* = 2.5 Hz, 1H), 4.80 (*td*, *J* = 0.7, 2.3 Hz, 1H). ^13^C-NMR (126 MHz, CDCl_3_): *δ* 19.2, 23.5, 26.7, 27.8, 28.5, 30.5, 35.4, 44.0, 46.7, 70.6, 103.7, 161.4, 173.8.

### 3.13. Synthesis of Allyl (R)-2-(2,2-dimethyl-3-methylenecyclopentyl)acetate (***6g***) [33]

Following *GP1*. Prepared from (+)-isocampholenic acid (**3**) (1.0 mmol, 168 mg), K_2_CO_3_ (2.0 mmol, 276 mg), MeCN (2 mL) and 3-bromopropene (1.2 mmol, 107 μL); column chromatography: EtOAc/petroleum ether = 1:10. Yield: 149 mg (0.71 mmol, 71%) of colorless oil. [*α*]_D_^r.t.^ = +10.0 (0.25, CH_2_Cl_2_). EI-HRMS: *m*/*z* = 209.1536 (MH^+^); C_13_H_21_O_2_: requires *m*/*z* = 209.1536 (MH^+^); *ν*_max_ 2960, 1735, 1651, 1462, 1364, 1289, 1176, 1139, 1057, 987, 929, 879 cm^−1^. ^1^H-NMR (500 MHz, CDCl_3_): *δ* 0.84 (*s*, 3H), 1.07 (*s*, 3H), 1.31–1.41 (*m*, 1H), 1.87–1.96 (*m*, 1H), 1.97–2.07 (*m*, 1H), 2.15 (*dd*, *J* = 10.6, 14.7 Hz, 1H), 2.29–2.39 (*m*, 1H), 2.40–2.51 (*m*, 2H), 4.58 (*t*, *J* = 1.4 Hz, 1H), 4.59 (*t*, *J* = 1.4 Hz, 1H), 4.78 (*t*, *J* = 2.5 Hz, 1H), 4.80 (*td*, *J* = 0.7, 2.2 Hz, 1H), 5.24 (*dq*, *J* = 1.3, 10.4 Hz, 1H), 5.33 (*dq*, *J* = 1.5, 17.2 Hz, 1H), 5.93 (*ddt*, *J* = 5.8, 10.4, 17.2 Hz, 1H). ^13^C-NMR (126 MHz, CDCl_3_): *δ* 23.6, 26.7, 28.4, 30.5, 35.3, 44.0, 46.7, 65.2, 103.7, 118.4, 132.4, 161.3, 173.4.

### 3.14. Synthesis of Benzyl (R)-2-(2,2-dimethyl-3-methylenecyclopentyl)acetate (***6h***)

Following *GP2*. Prepared from (+)-isocampholenic acid (**3**) (1.0 mmol, 168 mg), benzyl alcohol (1.8 mmol, 189 μL), DMAP (0.1 mmol, 12.2 mg), CH_2_Cl_2_ (7 mL) in EDCI (2.0 mmol, ω = 98%, 391 mg); column chromatography: EtOAc/petroleum ether = 1:30. Yield: 216 mg (0.83 mmol, 83%) of colorless oil. [*α*]_D_^r.t.^ = +9.0 (0.27, CH_2_Cl_2_). EI-HRMS: *m*/*z* = 259.1698 (MH^+^); C_17_H_23_O_2_: requires *m*/*z* = 259.1693 (MH^+^); *ν*_max_ 2959, 1733, 1652, 1456, 1363, 1289, 1255, 1137, 971, 880, 735, 696 cm^−1^. ^1^H-NMR (500 MHz, CDCl_3_): *δ* 0.80 (*s*, 3H), 1.02 (*s*, 3H), 1.29–1.41 (*m*, 1H), 1.85–1.94 (*m*, 1H), 1.98–2.08 (*m*, 1H), 2.17 (*dd*, *J* = 10.6, 14.8 Hz, 1H), 2.30–2.37 (*m*, 1H), 2.39–2.50 (*m*, 2H), 4.77 (*t*, *J* = 2.3 Hz, 1H), 4.79 (*t*, *J* = 1.8 Hz, 1H), 5.12 (*s*, 2H), 7.30–7.40 (*m*, 5H). ^13^C-NMR (126 MHz, CDCl_3_): *δ* 23.6, 26.7, 28.4, 30.5, 35.4, 44.0, 46.7, 66.3, 103.7, 128.3, 128.4, 128.7, 136.1, 161.3, 173.6.

### 3.15. Synthesis of Dodecyl (R)-2-(2,2-dimethyl-3-methylenecyclopentyl)acetate (***6i***)

Following *GP1*. Prepared from (+)-isocampholenic acid (**3**) (1.0 mmol, 168 mg), K_2_CO_3_ (2.0 mmol, 276 mg), MeCN (2 mL) and 1-bromododecane (1.5 mmol, ω = 0.97, 371 μL); column chromatography: EtOAc/petroleum ether = 1:30. Yield: 188 mg (0.56 mmol, 56%) of colorless oil. [*α*]_D_^r.t.^ = +6.2 (0.121, CH_2_Cl_2_). EI-HRMS: *m*/*z* = 337.3099 (MH^+^); C_22_H_41_O_2_: requires *m*/*z* = 337.3101 (MH^+^); *ν*_max_ 2923, 2854, 1736, 1652, 1465, 1363, 1289, 1178, 1141, 880, 722 cm^−1^. ^1^H-NMR (500 MHz, CDCl_3_): *δ* 0.84 (*s*, 3H), 0.88 (*t*, *J* = 6.9 Hz, 3H), 1.07 (*s*, 3H), 1.20–1.41 (*m*, 19H), 1.58–1.67 (*m*, 2H), 1.85–1.94 (*m*, 1H), 1.95–2.06 (*m*, 1H), 2.11 (*dd*, *J* = 10.5 Hz, 14.6, 1H), 2.28–2.51 (*m*, 3H), 4.07 (*t*, *J* = 6.8 Hz, 2H), 4.77 (*t*, *J* = 2.6, 1H), 4.79–4.81 (*m*, 1H). ^13^C-NMR (126 MHz, CDCl_3_): *δ* 14.3, 22.8, 23.6, 26.1, 26.7, 28.5, 28.8, 29.4, 29.5, 29.7, 29.7, 29.8, 29.8, 30.5, 32.1, 35.5, 44.0, 46.8, 64.7, 103.7, 161.5, 173.9.

### 3.16. Synthesis of (R)-3,7-dimethyloct-6-en-1-yl 2-((R)-2,2-dimethyl-3-methylenecyclopentyl)acetate (***6j***)

Following *GP2*. Prepared from (+)-isocampholenic acid (**3**) (1.0 mmol, 168 mg), (*R*)-(+)-β-citronellol (1.1 mmol, ω = 0.95, 211 μL), DMAP (0.1 mmol, 12.2 mg), CH_2_Cl_2_ (7 mL) in EDCI (2.0 mmol, ω = 98%, 391 mg); column chromatography: EtOAc/petroleum ether = 1:30. Yield: 242 mg (0.79 mmol, 79%) of colorless oil. [*α*]_D_^r.t.^ = +6.8 (0.272, CH_2_Cl_2_). EI-HRMS: *m*/*z* = 307.2635 (MH^+^); C_20_H_35_O_2_: requires *m*/*z* = 307.2632 (MH^+^); *ν*_max_ 2959, 1735, 1652, 1460, 1378, 1290, 1178, 1140, 1058, 982, 880 cm^−1^. ^1^H-NMR (500 MHz, CDCl_3_): *δ* 0.84 (*s*, 3H), 0.92 (*d*, *J* = 6.6 Hz, 3H), 1.07 (*s*, 3H), 1.14–1.23 (*m*, 1H), 1.31–1.40 (*m*, 2H), 1.41–1.48 (*m*, 1H), 1.51–1.59 (*m*, 1H), 1.60 (*s*, 3H), 1.63–1.72 (*m*, 1H), 1.68 (*s*, 3H), 1.86–2.05 (*m*, 4H), 2.10 (*dd*, *J* = 10.5, 14.6 Hz, 1H), 2.28–2.36 (*m*, 1H), 2.39 (*dd*, *J* = 4.2, 14.6 Hz, 1H), 2.42–2.50 (*m*, 1H), 4.05–4.18 (*m*, 2H), 4.77 (*t*, *J* = 2.5 Hz, 1H), 4.79 (*t*, *J* = 2.2 Hz, 1H), 5.05–5.13 (*m*, 1H). ^13^C-NMR (126 MHz, CDCl_3_): *δ* 17.8, 19.5, 23.6, 25.5, 25.9, 26.7, 28.4, 29.6, 30.5, 35.5, 35.6, 37.1, 44.0, 46.7, 63.0, 103.7, 124.7, 131.5, 161.4, 173.9.

### 3.17. Synthesis of Esters ***7*** from Alcohol ***4***—General Procedure 3 (GP3)

To a solution of (*R*)-2-(2,2-dimethyl-3-methylenecyclopentyl)ethan-1-ol (**4**) (1.0 equiv.) in anhydrous CH_2_Cl_2_ (2 mL) under argon, pyridine (3.0 equiv.), DMAP (0.04 equiv.) and the corresponding acid anhydride/acid chloride were added. The resulting reaction mixture was stirred at room temperature for 20 h. The volatiles were evaporated in vacuo. The residue was purified by column chromatography (Silica gel 60). The fractions containing the pure product **7** were combined, and the volatiles were evaporated in vacuo. The isolated esters **7** were fully characterized.

### 3.18. Synthesis of (R)-2-(2,2-dimethyl-3-methylenecyclopentyl)ethyl Acetate (***7a***)

Following *GP3*. Prepared from (*R*)-2-(2,2-dimethyl-3-methylenecyclopentyl)ethan-1-ol (**4**) (1.0 mmol, 154 mg), CH_2_Cl_2_ (2 mL), pyridine (3.0 mmol, 243 μL), DMAP (0.04 mmol, 4.9 mg) and acetic anhydride (1.5 mmol, 142 μL); column chromatography: EtOAc/petroleum ether = 1:20. Yield: 169 mg (0.86 mmol, 86%) of colorless oil. [*α*]_D_^r.t.^ = +10.0 (0.34, CH_2_Cl_2_). EI-HRMS: *m*/*z* = 197.1497 (MH^+^); C_12_H_21_O_2_: requires *m*/*z* = 197.1493 (MH^+^); *ν*_max_ 2959, 2868, 1739, 1652, 1463, 1434, 1364, 1228, 1153, 1038, 968, 878, 636, 606 cm^−1^. ^1^H-NMR (500 MHz, CDCl_3_): *δ* 0.82 (*s*, 3H), 1.06 (*s*, 3H), 1.24–1.37 (*m*, 1H), 1.37–1.48 (*m*, 1H), 1.51–1.59 (*m*, 1H), 1.75–1.82 (*m*, 1H), 1.82–1.90 (*m*, 1H), 2.05 (*s*, 3H), 2.24–2.36 (*m*, 1H), 2.42–2.52 (*m*, 1H), 4.05–4.11 (*m*, 1H), 4.12–4.18 (*m*, 1H), 4.74–4.80 (*m*, 2H). ^13^C-NMR (126 MHz, CDCl_3_): *δ* 21.2, 23.5, 26.6, 28.2, 29.0, 30.8, 44.1, 47.1, 64.2, 103.3, 162.1, 171.3.

### 3.19. Synthesis of (R)-2-(2,2-dimethyl-3-methylenecyclopentyl)ethyl Propionate (***7b***)

Following *GP3*. Prepared from (*R*)-2-(2,2-dimethyl-3-methylenecyclopentyl)ethan-1-ol (**4**) (1.0 mmol, 154 mg), CH_2_Cl_2_ (2 mL), pyridine (3.0 mmol, 243 μL), DMAP (0.04 mmol, 4.9 mg) and propionic anhydride (1.5 mmol, 194 μL); column chromatography: EtOAc/petroleum ether = 1:20. Yield: 203 mg (0.96 mmol, 96%) of colorless oil. [*α*]_D_^r.t.^ = +14.0 (0.35, CH_2_Cl_2_). EI-HRMS: *m*/*z* = 211.1689 (MH^+^); C_13_H_23_O_2_: requires *m*/*z* = 211.1693 (MH^+^); *ν*_max_ 2959, 1736, 1652, 1463, 1384, 1362, 1348, 1274, 1179, 1082, 1022, 958, 878, 807 cm^−1^. ^1^H-NMR (300 MHz, CDCl_3_): *δ* 0.83 (*s*, 3H), 1.06 (*s*, 3H), 1.14 (*t*, *J* = 7.6 Hz, 3H), 1.19–1.58 (*m*, 3H), 1.70–1.95 (*m*, 2H), 2.20–2.37 (*m*, 3H), 2.39–2.54 (*m*, 1H), 4.01–4.23 (*m*, 2H), 4.73–4.82 (*m*, 2H). ^13^C-NMR (75 MHz, CDCl_3_): *δ* 9.3, 23.4, 26.6, 27.8, 28.3, 29.1, 30.8, 44.1, 47.2, 64.1, 103.3, 162.1, 174.6.

### 3.20. Synthesis of (R)-2-(2,2-dimethyl-3-methylenecyclopentyl)ethyl Butyrate (***7c***)

Following *GP3*. Prepared from (*R*)-2-(2,2-dimethyl-3-methylenecyclopentyl)ethan-1-ol (**4**) (1.0 mmol, 154 mg), CH_2_Cl_2_ (2 mL), pyridine (3.0 mmol, 243 μL), DMAP (0.04 mmol, 4.9 mg) and butyric anhydride (1.3 mmol, 215 μL); column chromatography: EtOAc/petroleum ether = 1:40. Yield: 198 mg (0.88 mmol, 88%) of colorless oil. [*α*]_D_^r.t.^ = +12.0 (0.34, CH_2_Cl_2_). EI-HRMS: *m*/*z* = 225.1854 (MH^+^); C_14_H_25_O_2_: requires *m*/*z* = 225.1849 (MH^+^); *ν*_max_ 2960, 2871, 1735, 1652, 1461, 1362, 1252, 1174, 1091, 1048, 992, 975, 933, 878 cm^−1^. ^1^H-NMR (300 MHz, CDCl_3_): *δ* 0.82 (*s*, 3H), 0.95 (*t*, *J* = 7.4 Hz, 3H), 1.06 (*s*, 3H), 1.20–1.95 (*m*, 7H), 2.21–2.37 (*m*, 3H), 2.40–2.54 (*m*, 1H), 4.01–4.23 (*m*, 2H), 4.73–4.82 (*m*, 2H). ^13^C-NMR (75 MHz, CDCl_3_): *δ* 13.8, 18.6, 23.5, 26.6, 28.2, 29.2, 30.8, 36.4, 44.1, 47.2, 63.9, 103.3, 162.2, 173.9.

### 3.21. Synthesis of (R)-2-(2,2-dimethyl-3-methylenecyclopentyl)ethyl Isobutyrate (***7d***)

Following *GP3*. Prepared from (*R*)-2-(2,2-dimethyl-3-methylenecyclopentyl)ethan-1-ol (**4**) (1.0 mmol, 154 mg), CH_2_Cl_2_ (2 mL), pyridine (3.0 mmol, 243 μL), DMAP (0.04 mmol, 4.9 mg) and isobutyric anhydride (1.5 mmol, 249 μL); column chromatography: EtOAc/petroleum ether = 1:30. Yield: 181 mg (0.80 mmol, 80%) of colorless oil. [*α*]_D_^r.t.^ = +15.0 (0.30, CH_2_Cl_2_). EI-HRMS: *m*/*z* = 225.1854 (MH^+^); C_14_H_25_O_2_: requires *m*/*z* = 225.1849 (MH^+^); *ν*_max_ 2960, 1733, 1652, 1469, 1388, 1363, 1344,1258, 1190, 1153, 1075, 990, 971, 879, 754 cm^−1^. ^1^H-NMR (500 MHz, CDCl_3_): *δ* 0.82 (*s*, 3H), 1.06 (*s*, 3H), 1.17 (*d*, *J* = 7.0, 6H), 1.26–1.36 (*m*, 1H), 1.39–1.47 (*m*, 1H), 1.51–1.60 (*m*, 1H), 1.74–1.82 (*m*, 1H), 1.83–1.92 (*m*, 1H), 2.24–2.36 (*m*, 1H), 2.42–2.50 (*m*, 1H), 2.54 (*p*, *J* = 7.0 Hz, 1H), 4.04–4.11 (*m*, 1H), 4.12–4.20 (*m*, 1H), 4.74–4.81 (*m*, 2H). ^13^C-NMR (126 MHz, CDCl_3_): *δ* 19.2, 23.5, 26.6, 28.3, 29.1, 30.8, 34.2, 44.1, 47.2, 64.0, 103.3, 162.2, 177.4.

### 3.22. Synthesis of (R)-2-(2,2-dimethyl-3-methylenecyclopentyl)ethyl 3,3-dimethylbutanoate (***7e***)

Following *GP3*. Prepared from (*R*)-2-(2,2-dimethyl-3-methylenecyclopentyl)ethan-1-ol (**4**) (1.0 mmol, 154 mg), CH_2_Cl_2_ (2 mL), pyridine (3.0 mmol, 243 μL), DMAP (0.04 mmol, 4.9 mg) and 3,3-dimethylbutyryl chloride (1.1 mmol, ω = 0.98, 153 μL); column chromatography: EtOAc/petroleum ether = 1:40. Yield: 187 mg (0.74 mmol, 74%) of colorless oil. [*α*]_D_^r.t.^ = +10.3 (0.25, CH_2_Cl_2_). *ν*_max_ 3071, 2957, 2869, 1733, 1652, 1466, 1434, 1365, 1322, 1226, 1197, 1129, 1049, 1020, 996, 978, 931, 879, 795, 729, 704, 620 cm^−1^. ^1^H-NMR (300 MHz, CDCl_3_): *δ* 0.83 (*s*, 3H), 1.03 (*s*, 9H), 1.06 (*s*, 3H), 1.22–1.48 (*m*, 2H), 1.50–1.64 (*m*, 1H), 1.70–1.95 (*m*, 2H), 2.19 (*s*, 2H), 2.22–2.37 (*m*, 1H), 2.39–2.55 (*m*, 1H), 3.99–4.23 (*m*, 2H), 4.72–4.82 (*m*, 2H). ^13^C-NMR (75 MHz, CDCl_3_): *δ* 23.5, 26.6, 28.2, 29.2, 29.6, 29.8, 30.8, 44.1, 47.2, 48.2, 63.6, 103.3, 162.2, 172.6.

### 3.23. Synthesis of (R)-2-(2,2-dimethyl-3-methylenecyclopentyl)ethyl pent-4-enoate (***7f***)

Following *GP3*. Prepared from (*R*)-2-(2,2-dimethyl-3-methylenecyclopentyl)ethan-1-ol (**4**) (1.0 mmol, 154 mg), CH_2_Cl_2_ (2 mL), pyridine (3.0 mmol, 243 μL), DMAP (0.04 mmol, 4.9 mg) and 4-pentenoic anhydride (1.5 mmol, ω = 0.98, 280 μL); column chromatography: EtOAc/petroleum ether = 1:40. Yield: 194 mg (0.82 mmol, 82%) of colorless oil. [*α*]_D_^r.t.^ = +13.9 (0.28, CH_2_Cl_2_). EI-HRMS: *m*/*z* = 237.1849 (MH^+^); C_15_H_25_O_2_: requires *m*/*z* = 237.1849 (MH^+^); *ν*_max_ 3073, 2959, 2868, 1735, 1651, 1463, 1435, 1362, 1235, 1167, 1102, 1050, 993, 914, 879 cm^−1^. ^1^H-NMR (300 MHz, CDCl_3_): *δ* 0.82 (*s*, 3H), 1.06 (*s*, 3H), 1.20–1.65 (*m*, 3H), 1.70–1.94 (*m*, 2H), 2.20–2.54 (*m*, 6H), 4.02–4.24 (*m*, 2H), 4.72–4.82 (*m*, 2H), 4.95–5.13 (*m*, 2H), 5.73–5.92 (*m*, 1H). ^13^C-NMR (75 MHz, CDCl_3_): *δ* 23.5, 26.6, 28.2, 29.0, 29.1, 30.8, 33.8, 44.1, 47.2, 64.1, 103.3, 115.6, 136.9, 162.1, 173.2.

### 3.24. Synthesis of (R)-2-(2,2-dimethyl-3-methylenecyclopentyl)ethyl Benzoate (***7g***)

Following *GP3*. Prepared from (*R*)-2-(2,2-dimethyl-3-methylenecyclopentyl)ethan-1-ol (**4**) (1.0 mmol, 154 mg), CH_2_Cl_2_ (2 mL), pyridine (3.0 mmol, 243 μL), DMAP (0.04 mmol, 4.9 mg) and benzoic anhydride (1.3 mmol, 297 mg); column chromatography: EtOAc/petroleum ether = 1:30. Yield: 226 mg (0.87 mmol, 87%) of colorless oil. [*α*]_D_^r.t.^ = +8.0 (0.37, CH_2_Cl_2_). EI-HRMS: *m*/*z* = 259.1691 (MH^+^); C_17_H_23_O_2_: requires *m*/*z* = 259.1693 (MH^+^); *ν*_max_ 2958, 1717, 1651, 1602, 1452, 1385, 1362, 1314, 1268, 1175, 1110, 1069, 1026, 958, 878, 708, 687, 675 cm^−1^. ^1^H-NMR (500 MHz, CDCl_3_): *δ* 0.86 (*s*, 3H), 1.10 (*s*, 3H), 1.31–1.44 (*m*, 1H), 1.52–1.61 (*m*, 1H), 1.62–1.70 (*m*, 1H), 1.88–2.00 (*m*, 2H), 2.26–2.38 (*m*, 1H), 2.44–2.54 (*m*, 1H), 4.30–4.44 (*m*, 2H), 4.76–4.82 (*m*, 2H), 7.40–7.48 (*m*, 2H), 7.52–7.59 (*m*, 1H), 8.01–8.08 (*m*, 2H). ^13^C-NMR (126 MHz, CDCl_3_): *δ* 23.5, 26.6, 28.3, 29.2, 30.8, 44.2, 47.4, 64.8, 103.3, 128.5, 129.7, 130.6, 133.0, 162.1, 166.8.

### 3.25. Synthesis of (R)-4-(2-(2,2-dimethyl-3-methylenecyclopentyl)ethoxy)-4-oxobutanoic Acid (***7h***)

Following *GP3*. Prepared from (*R*)-2-(2,2-dimethyl-3-methylenecyclopentyl)ethan-1-ol (**4**) (1.0 mmol, 154 mg), CH_2_Cl_2_ (2 mL), pyridine (3.0 mmol, 243 μL), DMAP (0.04 mmol, 4.9 mg) and benzoic anhydride (1.0 mmol, 100 mg). The residue (after evaporation of the volatiles) was dissolved in EtOAc (50 mL) and washed with NaHSO_4_ (aq., 1 M, 2 × 5 mL) and NaCl (aq. sat., 5 mL). The organic phase was dried under anhydrous Na_2_SO_4_, filtered, and the volatile components evaporated in vacuo. The residue was purified by column chromatography (EtOAc). Yield: 191 mg (0.75 mmol, 75%) of colorless oil. [*α*]_D_^r.t.^ = +4.8 (0.255, CH_2_Cl_2_). EI-HRMS: *m*/*z* = 255.1589 (MH^+^); C_14_H_23_O_4_: requires *m*/*z* = 255.1591 (MH^+^); *ν*_max_ 2958, 1734, 1710, 1651, 1397, 1362, 1163, 1047, 993, 930, 878, 840 cm^−1^. ^1^H-NMR (300 MHz, CDCl_3_): *δ* 0.82 (*s*, 3H), 1.06 (*s*, 3H), 1.20–1.64 (*m*, 3H), 1.71–1.93 (*m*, 2H), 2.21–2.37 (*m*, 1H), 2.39–2.55 (*m*, 1H), 2.56–2.74 (*m*, 4H), 4.04–4.26 (*m*, 2H), 4.72–4.82 (*m*, 2H), 10.33 (br *s*, 1H). ^13^C-NMR (75 MHz, CDCl_3_): *δ* 23.4, 26.6, 28.2, 29.0, 29.0, 29.1, 30.8, 44.1, 47.1, 64.6, 103.3, 162.0, 172.3, 178.3.

### 3.26. Synthesis of (R)-2-(2,2-dimethyl-3-methylenecyclopentyl)ethyl Methyl Succinate (***7i***)

Following *GP2*. Prepared from (*R*)-4-(2-(2,2-dimethyl-3-methylenecyclopentyl)ethoxy)-4-oxobutanoic acid (**7h**) (1.0 mmol, 254 mg), methanol (3.0 mmol, 122 μL), DMAP (0.1 mmol, 12.2 mg), and CH_2_Cl_2_ (7 mL) in EDCI (2.0 mmol, ω = 98%, 391 mg); column chromatography: EtOAc/petroleum ether = 1:10. Yield: 215 mg (0.80 mmol, 80%) of colorless oil. [*α*]_D_^r.t.^ = +10.6 (0.23, CH_2_Cl_2_). EI-HRMS: *m*/*z* = 269.1751 (MH^+^); C_15_H_25_O_4_: requires *m*/*z* = 269.1747 (MH^+^); *ν*_max_ 2957, 1733, 1651, 1436, 1362, 1317, 1155, 996, 879, 846 cm^−1^. ^1^H-NMR (500 MHz, CDCl_3_): *δ* 0.82 (*s*, 3H), 1.06 (*s*, 3H), 1.24–1.36 (*m*, 1H), 1.37–1.48 (*m*, 1H), 1.49–1.59 (*m*, 1H), 1.73–1.90 (*m*, 2H), 2.24–2.36 (*m*, 1H), 2.41–2.52 (*m*, 1H), 2.64 (*s*, 4H), 3.70 (*s*, 3H), 4.06–4.22 (*m*, 2H), 4.74–4.80 (*m*, 2H). ^13^C-NMR (126 MHz, CDCl_3_): *δ* 23.5, 26.6, 28.2, 29.0, 29.1, 29.3, 30.8, 44.1, 47.1, 52.0, 64.5, 103.3, 162.1, 172.5, 172.9.

### 3.27. Synthesis of (R)-2-(2,2-dimethyl-3-methylenecyclopentyl)ethyl Succinate (***7j***)

Following *GP2*. Prepared from (*R*)-4-(2-(2,2-dimethyl-3-methylenecyclopentyl)ethoxy)-4-oxobutanoic acid (**7h**) (1.0 mmol, 254 mg), ethanol (3.0 mmol, 175 μL), DMAP (0.1 mmol, 12.2 mg), and CH_2_Cl_2_ (7 mL) in EDCI (2.0 mmol, ω = 98%, 391 mg); column chromatography: EtOAc/petroleum ether = 1:10. Yield: 220 mg (0.78 mmol, 78%) of colorless oil. [*α*]_D_^r.t.^ = +11.6 (0.23, CH_2_Cl_2_). EI-HRMS: *m*/*z* = 283.1904 (MH^+^); C_16_H_27_O_4_: requires *m*/*z* = 283.1904 (MH^+^); *ν*_max_ 2959, 1732, 1651, 1464, 1363, 1349, 1314, 1154, 1125, 967. 879 cm^−1^. ^1^H-NMR (300 MHz, CDCl_3_): *δ* 0.82 (*s*, 3H), 1.06 (*s*, 3H), 1.26 (*t*, *J* = 7.1 Hz, 3H), 1.22–1.32 (*m*, 1H), 1.36–1.61 (*m*, 2H), 1.70–1.94 (*m*, 2H), 2.21–2.37 (*m*, 1H), 2.38–2.57 (*m*, 1H), 2.62 (*s*, 4H), 4.03–4.26 (*m*, 4H), 4.73–4.82 (*m*, 2H). ^13^C-NMR (75 MHz, CDCl_3_): *δ* 14.3, 23.5, 26.6, 28.2, 29.1, 29.4, 29.4, 30.8, 44.1, 47.2, 60.8, 64.5, 103.3, 162.1, 172.4, 172.5.

### 3.28. Synthesis of Ethers ***8*** from Alcohol ***4***—General Procedure 4 (GP4)

To a solution of (*R*)-2-(2,2-dimethyl-3-methylenecyclopentyl)ethan-1-ol (**4**) (1.0 equiv.) in anhydrous THF (3 mL) under argon at 0 °C was added NaH (1.2 equiv.). The resulting reaction mixture was stirred at 0 °C for 30 min, then the electrophile (aliphatic halide) was added. After stirring at room temperature for 20 h, H_2_O (10 mL) was carefully added. The resulting mixture was extracted with Et_2_O (30 mL). The organic phase was washed with NaCl (aq. sat., 3 × 10 mL), dried under anhydrous Na_2_SO_4_, filtered, and the volatiles evaporated in vacuo. The residue was purified by column chromatography (Silica gel 60). The fractions containing the pure product **8** were combined, and the volatiles were evaporated in vacuo. The isolated ethers **8** were fully characterized with the exception of HRMS, as the products were not ionized.

### 3.29. Synthesis of (R)-2-(2-methoxyethyl)-1,1-dimethyl-5-methylenecyclopentane (***8a***)

Following *GP4*. Prepared from (*R*)-2-(2,2-dimethyl-3-methylenecyclopentyl)ethan-1-ol (**4**) (1.0 mmol, 154 mg), THF (3 mL), NaH (1.2 mmol; ω = 60%, 48.0 mg) and iodomethane (2.0 mmol, 129 μL); column chromatography: EtOAc/petroleum ether = 1:20. Yield: 156 mg (0.93 mmol, 93%) of colorless oil. [*α*]_D_^r.t.^ = +13.0 (0.20, CH_2_Cl_2_). *ν*_max_ 2958, 2866, 1651, 1462, 1386, 1362, 1199, 1152, 1120, 1003, 957, 877, 836, 704 cm^−1^. ^1^H-NMR (500 MHz, CDCl_3_): *δ* 0.82 (*s*, 3H), 1.06 (*s*, 3H), 1.24–1.32 (*m*, 1H), 1.33–1.40 (*m*, 1H), 1.51–1.61 (*m*, 1H), 1.71–1.78 (*m*, 1H), 1.80–1.88 (*m*, 1H), 2.23–2.35 (*m*, 1H), 2.40–2.50 (*m*, 1H), 3.34 (*s*, 3H), 3.37–3.48 (*m*, 2H), 4.73–4.80 (*m*, 2H). ^13^C-NMR (126 MHz, CDCl_3_): *δ* 23.5, 26.6, 28.3, 30.0, 30.9, 44.1, 47.1, 58.7, 72.4, 103.1, 162.6.

### 3.30. Synthesis of (R)-2-(2-ethoxyethyl)-1,1-dimethyl-5-methylenecyclopentane (***8b***)

Following *GP4*. Prepared from (*R*)-2-(2,2-dimethyl-3-methylenecyclopentyl)ethan-1-ol (**4**) (1.0 mmol, 154 mg), THF (3 mL), NaH (1.2 mmol; ω = 60%, 48.0 mg) and bromoethane (2.0 mmol, 153 μL); column chromatography: EtOAc/petroleum ether = 1:20. Yield: 125 mg (0.68 mmol, 68%) of colorless oil. [*α*]_D_^r.t.^ = +17.0 (0.22, CH_2_Cl_2_). *ν*_max_ 2959, 2863, 1651, 1463, 1377, 1362, 1111, 877, 704 cm^−1^. ^1^H-NMR (500 MHz, CDCl_3_): *δ* 0.82 (*s*, 3H), 1.06 (*s*, 3H), 1.21 (*t*, *J* = 7.0 Hz, 3H), 1.25–1.35 (*m*, 1H), 1.36–1.43 (*m*, 1H), 1.50–1.58 (*m*, 1H), 1.73–1.79 (*m*, 1H), 1.79–1.87 (*m*, 1H), 2.23–2.37 (*m*, 1H), 2.40–2.50 (*m*, 1H), 3.37–3.54 (*m*, 4H), 4.73–4.79 (*m*, 2H). ^13^C-NMR (126 MHz, CDCl_3_): *δ* 15.4, 23.5, 26.6, 28.4, 30.2, 30.9, 44.1, 47.3, 66.2, 70.4, 103.0, 162.6.

### 3.31. Synthesis of (R)-1,1-dimethyl-2-methylene-5-(2-propoxyethyl)cyclopentane (***8c***)

Following *GP4*. Prepared from (*R*)-2-(2,2-dimethyl-3-methylenecyclopentyl)ethan-1-ol (**4**) (1.0 mmol, 154 mg), THF (3 mL), NaH (1.2 mmol; ω = 60%, 48.0 mg) and 1-bromopropane (1.2 mmol, 109 μL); column chromatography: EtOAc/petroleum ether = 1:25. Yield: 154 mg (0.78 mmol, 78%) of colorless oil. [*α*]_D_^r.t.^ = +13.0 (0.22, CH_2_Cl_2_). *ν*_max_ 3071, 2959, 2935, 2860, 1651, 1462, 1435, 1362, 1249, 1115, 981, 956, 877, 703 cm^−1^. ^1^H-NMR (500 MHz, CDCl_3_): *δ* 0.82 (*s*, 3H), 0.92 (*t*, *J* = 7.5 Hz, 3H), 1.06 (*s*, 3H), 1.21–1.42 (*m*, 2H), 1.51–1.64 (*m*, 3H), 1.72–1.79 (*m*, 1H), 1.80–1.89 (*m*, 1H), 2.23–2.34 (*m*, 1H), 2.40–2.50 (*m*, 1H), 3.33–3.45 (*m*, 3H), 3.46–3.53 (*m*, 1H), 4.73–4.81 (*m*, 2H). ^13^C-NMR (126 MHz, CDCl_3_): *δ* 10.8, 23.1, 23.5, 26.6, 28.4, 30.1, 30.9, 44.1, 47.3, 70.5, 72.8, 103.0, 162.6.

### 3.32. Synthesis of (R)-2-(2-butoxyethyl)-1,1-dimethyl-5-methylenecyclopentane (***8d***)

Following *GP4*. Prepared from (*R*)-2-(2,2-dimethyl-3-methylenecyclopentyl)ethan-1-ol (**4**) (1.0 mmol, 154 mg), THF (3 mL), NaH (1.2 mmol; ω = 60%, 48.0 mg) and 1-iodobutane (1.0 mmol, 114 μL); column chromatography: EtOAc/petroleum ether = 1:30. Yield: 177 mg (0.84 mmol, 84%) of colorless oil. [*α*]_D_^r.t.^ = +14.0 (0.20, CH_2_Cl_2_). *ν*_max_ 3071, 2957, 2928, 2857, 2796, 1652, 1463, 1434, 1375, 1362, 1302, 1234, 1114, 998, 962, 940, 878, 738, 704 cm^−1^. ^1^H-NMR (500 MHz, CDCl_3_): *δ* 0.82 (*s*, 3H), 0.92 (*t*, *J* = 7.4 Hz, 3H), 1.06 (*s*, 3H), 1.19–1.48 (*m*, 4H), 1.48–1.62 (*m*, 3H), 1.70–1.88 (*m*, 2H), 2.23–2.37 (*m*, 1H), 2.40–2.50 (*m*, 1H), 3.36–3.52 (*m*, 4H), 4.73–4.79 (*m*, 2H). ^13^C-NMR (126 MHz, CDCl_3_): *δ* 14.1, 19.6, 23.6, 26.6, 28.4, 30.1, 30.9, 32.0, 44.1, 47.3, 70.6, 70.9, 103.0, 162.6.

### 3.33. Synthesis of (R)-2-(2-(allyloxy)ethyl)-1,1-dimethyl-5-methylenecyclopentane (***8e***)

Following *GP4*. Prepared from (*R*)-2-(2,2-dimethyl-3-methylenecyclopentyl)ethan-1-ol (**4**) (1.0 mmol, 154 mg), THF (3 mL), NaH (1.2 mmol; ω = 60%, 48.0 mg) and allyl bromide (1.1 mmol, ω = 0.97, 98 μL); column chromatography: EtOAc/petroleum ether = 1:15. Yield: 161 mg (0.83 mmol, 83%) of colorless oil. [*α*]_D_^r.t.^ = +23.0 (0.17, CH_2_Cl_2_). *ν*_max_ 3071, 2958, 2934, 2865, 1651, 1463, 1433, 1362, 1145, 1106, 993, 920, 878, 704, 634 cm^−1^. ^1^H-NMR (500 MHz, CDCl_3_): *δ* 0.82 (*s*, 3H), 1.06 (*s*, 3H), 1.23–1.32 (*m*, 1H), 1.35–1.45 (*m*, 1H), 1.52–1.62 (*m*, 1H), 1.72–1.89 (*m*, 2H), 2.23–2.33 (*m*, 1H), 2.40–2.50 (*m*, 1H), 3.41–3.47 (*m*, 1H), 3.51 (*td*, *J* = 5.2, 8.8 Hz, 1H), 3.92–4.03 (*m*, 2H), 4.73–4.79 (*m*, 2H), 5.17 (*dq*, *J* = 1.4, 10.3 Hz, 1H), 5.28 (*dq*, *J* = 1.7, 17.2 Hz, 1H), 5.93 (*ddt*, *J* = 5.6, 10.4, 17.2, 1H). ^13^C-NMR (126 MHz, CDCl_3_): *δ* 23.5, 26.6, 28.4, 30.1, 30.9, 44.1, 47.2, 70.0, 72.0, 103.0, 116.9, 135.1, 162.6.

### 3.34. Synthesis of (R)-1,1-dimethyl-2-(2-((3-methylbut-2-en-1-yl)oxy)ethyl)-5-methylenecyclopentane (***8f***)

Following *GP4*. Prepared from (*R*)-2-(2,2-dimethyl-3-methylenecyclopentyl)ethan-1-ol (**4**) (1.0 mmol, 154 mg), THF (3 mL), NaH (1.2 mmol; ω = 60%, 48.0 mg) and prenyl bromide (1.0 mmol, ω = 0.95, 121 μL); column chromatography: EtOAc/petroleum ether = 1:20. Yield: 171 mg (0.77 mmol, 77%) of colorless oil. [*α*]_D_^r.t.^ = +2.5 (0.24, CH_2_Cl_2_). EI-HRMS: *m*/*z* = 223.2056 (MH^+^); C_15_H_27_O: requires *m*/*z* = 223.2056 (MH^+^); *ν*_max_ 2929, 2862, 1445, 1377, 1360, 1081, 1014, 799 cm^−1^. ^1^H-NMR (500 MHz, CDCl_3_): *δ* 0.81 (*s*, 3H), 1.06 (*s*, 3H), 1.24–1.34 (*m*, 1H), 1.34–1.43 (*m*, 1H), 1.49–1.61 (*m*, 1H), 1.68 (*s*, 3H), 1.75 (*s*, 3H), 1.76–1.87 (*m*, 2H), 2.23–2.34 (*m*, 1H), 2.40–2.50 (*m*, 1H), 3.42 (*ddd*, *J* = 6.9, 8.1, 9.1 Hz, 1H), 3.49 (*td*, *J* = 5.1, 8.9 Hz, 1H), 3.90–4.01 (*m*, 2H), 4.73–4.79 (*m*, 2H), 5.32–5.40 (*m*, 1H). ^13^C-NMR (75 MHz, CDCl_3_): *δ* 18.1, 23.5, 25.9, 26.7, 28.4, 30.2, 30.9, 44.1, 47.3, 67.4, 69.9, 103.0, 121.5, 136.7, 162.6.

### 3.35. Synthesis of (R)-1,1-dimethyl-2-methylene-5-(2-(prop-2-yn-1-yloxy)ethyl)cyclopentane (***8g***)

Following *GP4*. Prepared from (*R*)-2-(2,2-dimethyl-3-methylenecyclopentyl)ethan-1-ol (**4**) (1.0 mmol, 154 mg), THF (3 mL), NaH (1.2 mmol; ω = 60%, 48.0 mg) and propargyl bromide (1.1 mmol, ω = 0.80, 118 μL); column chromatography: EtOAc/petroleum ether = 1:20. Yield: 125 mg (0.65 mmol, 65%) of colorless oil. [*α*]_D_^r.t.^ = +17.9 (0.19, CH_2_Cl_2_). *ν*_max_ 3306, 2958, 2866, 1651, 1463, 1361, 1093, 1012, 878, 626 cm^−1^. ^1^H-NMR (500 MHz, CDCl_3_): *δ* 0.82 (*s*, 3H), 1.06 (*s*, 3H), 1.26–1.34 (*m*, 1H), 1.37–1.45 (*m*, 1H), 1.52–1.63 (*m*, 1H), 1.74–1.81 (*m*, 1H), 1.82–1.89 (*m*, 1H), 2.23–2.36 (*m*, 1H), 2.43 (*t*, *J* = 2.4 Hz, 1H), 2.43–2.51 (*m*, 1H), 3.53 (*dt*, *J* = 7.4, 8.9 Hz, 1H), 3.60 (*ddd*, *J* = 5.1, 8.1, 9.0 Hz, 1H), 4.10–4.21 (*m*, 2H), 4.73–4.81 (*m*, 2H). ^13^C-NMR (126 MHz, CDCl_3_): *δ* 23.5, 26.6, 28.3, 29.9, 30.8, 44.1, 47.1, 58.2, 69.8, 74.3, 80.1, 103.1, 162.5.

### 3.36. Synthesis of (R)-((2-(2,2-dimethyl-3-methylenecyclopentyl)ethoxy)methyl)benzene (***8h***)

Following *GP4*. Prepared from (*R*)-2-(2,2-dimethyl-3-methylenecyclopentyl)ethan-1-ol (**4**) (1.0 mmol, 154 mg), THF (3 mL), NaH (1.2 mmol; ω = 60%, 48.0 mg) and benzyl bromide (1.1 mmol, ω = 0.98, 134 μL); column chromatography: EtOAc/petroleum ether = 1:20. Yield: 209 mg (0.85 mmol, 85%) of colorless oil. [*α*]_D_^r.t.^ = +14.0 (0.23, CH_2_Cl_2_). EI-HRMS: *m*/*z* = 245.1898 (MH^+^); C_17_H_25_O: requires *m*/*z* = 245.1900 (MH^+^); *ν*_max_ 3067, 2958, 2864, 1651, 1496, 1454, 1361, 1202, 1100, 1028, 1000, 877, 733, 696, 611 cm^−1^. ^1^H-NMR (500 MHz, CDCl_3_): *δ* 0.82 (*s*, 3H), 1.06 (*s*, 3H), 1.20–1.34 (*m*, 1H), 1.36–1.47 (*m*, 1H), 1.59–1.60 (*m*, 1H), 1.75–1.86 (*m*, 2H), 2.27–2.28 (*m*, 1H), 2.39–2.50 (*m*, 1H), 3.45–3.51 (*m*, 1H), 3.52–3.60 (*m*, 1H), 4.49 (*d*, *J* = 11.8 Hz, 1H), 4.53 (*d*, *J* = 11.9 Hz, 1H), 4.73–4.79 (*m*, 2H), 7.25–7.31 (*m*, 1H), 7.32–7.39 (*m*, 4H). ^13^C-NMR (126 MHz, CDCl_3_): *δ* 23.6, 26.6, 28.4, 30.1, 30.9, 44.1, 47.2, 70.1, 73.1, 103.0, 127.6, 127.8, 128.5, 138.7, 162.6.

### 3.37. Synthesis of (R)-2-(2,2-dimethyl-3-methylenecyclopentyl)-N-methoxy-N-methylacetamide (***9***)

To a solution of (+)-isocampholenic acid (**3**) (10 mmol, 1.68 g) in anhydrous CH_2_Cl_2_ (50 mL) under argon at room temperature was added CDI (12 mmol, ω = 0.95, 2.05 g). The resulting reaction mixture was stirred at room temperature for 2 h, then *N*,*O*-dimethylhydroxylamine hydrochloride (30 mmol, 2.93 g) and Et_3_N (30 mmol, 4.18 mL) were added. After stirring the reaction mixture at room temperature for another 20 h, the volatile components were evaporated in vacuo. The residue was dissolved in EtOAc (150 mL) and washed with NaHSO_4_ (aq., 1 M, 2 × 50 mL) and NaCl (aq. sat., 2 × 50 mL). The organic phase was dried under anhydrous Na_2_SO_4_, filtered, and the volatile components evaporated in vacuo. Yield: 1.80 g (8.5 mmol, 85%) of white solid. [*α*]_D_^r.t.^ = +21.0 (0.30, CH_2_Cl_2_). EI-HRMS: *m*/*z* = 212.1606 (MH^+^); C_12_H_22_NO_2_: requires *m*/*z* = 212.1612 (MH^+^); *ν*_max_ 2959, 1662, 1462, 1436, 1413, 1384, 1362, 1178, 1114, 1002, 935, 878, 785, 969 cm^−1^. ^1^H-NMR (500 MHz, CDCl_3_): *δ* 0.87 (*s*, 3H), 1.08 (*s*, 3H), 1.29–1.40 (*m*, 1H), 1.90–1.98 (*m*, 1H), 2.02–2.10 (*m*, 1H), 2.21–2.38 (*m*, 2H), 2.41–2.52 (*m*, 2H), 3.19 (*s*, 3H), 3.69 (*s*, 3H), 4.78 (*t*, *J* = 2.5 Hz, 1H), 4.79 (*t*, *J* = 2.0 Hz, 1H). ^13^C-NMR (126 MHz, CDCl_3_): *δ* 23.7, 26.7, 28.6, 30.6, 32.3, 32.6, 44.0, 46.2, 61.3, 103.5, 161.8, 174.6.

### 3.38. Synthesis of Ketones ***11*** from Weinreb Amide ***10***—General Procedure 5 (GP5)

To a solution of (*R*)-2-(2,2-dimethyl-3-methylenecyclopentyl)-*N*-methoxy-*N*-methylacetamide (**9**) (1.0 equiv.) in anhydrous Et_2_O (7 mL) under argon at 0 °C, the corresponding Grignard reagent (1.5 equiv.) was added slowly. The resulting reaction mixture was stirred at 0 °C for 1 h and then at room temperature for 20 h. The excess Grignard reagent was quenched with NaCl (aq. sat., 3 mL), and the resulting mixture was extracted with Et_2_O (3 × 10 mL). The combined organic phase was washed with NaCl (aq. sat., 3 × 10 mL), dried under anhydrous Na_2_SO_4_, filtered, and the volatiles evaporated in vacuo. The residue was purified by column chromatography (Silica gel 60). The fractions containing the pure product **10** were combined, and the volatiles evaporated in vacuo. The isolated ketones **10** were fully characterized.

### 3.39. Synthesis of (R)-1-(2,2-dimethyl-3-methylenecyclopentyl)propan-2-one (***10a***)

Following *GP5*. Prepared from (*R*)-2-(2,2-dimethyl-3-methylenecyclopentyl)-*N*-methoxy-*N*-methylacetamide (**9**) (2.0 mmol, 422 mg), Et_2_O (7 mL), and methylmagnesium bromide (2.0 M in Et_2_O, 3.0 mmol, 1.5 mL); column chromatography: EtOAc/petroleum ether = 1:10. Yield: 278 mg (1.67 mmol, 83%) of colorless oil. [*α*]_D_^r.t.^ = +15.0 (0.22, CH_2_Cl_2_). EI-HRMS: *m*/*z* = 167.1430 (MH^+^); C_11_H_19_O: requires *m*/*z* = 167.1430 (MH^+^); *ν*_max_ 3071, 2960, 1712, 1651, 1463, 1434, 1362, 1285, 1241, 1170, 1148, 962, 878, 704 cm^−1^. ^1^H-NMR (500 MHz, CDCl_3_): *δ* 0.83 (*s*, 3H), 1.06 (*s*, 3H), 1.21–1.30 (*m*, 1H), 1.86–1.94 (*m*, 1H), 1.97–2.04 (*m*, 1H), 2.17 (*s*, 3H), 2.25 (*dd*, *J* = 10.5, 15.9 Hz, 1H), 2.29–2.38 (*m*, 1H), 2.40–2.48 (*m*, 1H), 2.50 (*dd*, *J* = 3.6, 15.9 Hz, 1H), 4.77 (*t*, *J* = 2.5 Hz, 1H), 4.79 (*td*, *J* = 0.8, 2.2 Hz, 1H). ^13^C-NMR (126 MHz, CDCl_3_): *δ* 23.7, 26.6, 28.4, 30.5, 30.6, 43.9, 44.6, 45.6, 103.6, 161.3, 209.2.

### 3.40. Synthesis of (R)-1-(2,2-dimethyl-3-methylenecyclopentyl)butan-2-one (***10b***) [34]

Following *GP5*. Prepared from (*R*)-2-(2,2-dimethyl-3-methylenecyclopentyl)-*N*-methoxy-*N*-methylacetamide (**9**) (2.0 mmol, 422 mg), Et_2_O (7 mL), and ethylmagnesium bromide (3.0 M in Et_2_O, 3.0 mmol, 1.0 mL); column chromatography: EtOAc/petroleum ether = 1:10. Yield: 314 mg (1.74 mmol, 87%) of colorless oil. [*α*]_D_^r.t.^ = +14.0 (0.23, CH_2_Cl_2_). EI-HRMS: *m*/*z* = 181.1588 (MH^+^); C_12_H_21_O: requires *m*/*z* = 181.1587 (MH^+^); *ν*_max_ 3071, 2960, 1712, 1651, 1460, 1413, 1376, 1363, 1283, 1151, 1114, 1021, 986, 878, 806, 705, 635 cm^−1^. ^1^H-NMR (500 MHz, CDCl_3_): *δ* 0.83 (*s*, 3H), 1.02–1.10 (*m*, 6H), 1.19–1.30 (*m*, 1H), 1.83–1.91 (*m*, 1H), 1.97–2.05 (*m*, 1H), 2.24 (*dd*, *J* = 10.6, 15.7 Hz, 1H), 2.29–2.38 (*m*, 1H), 2.40–2.54 (*m*, 4H), 4.77 (*t*, *J* = 2.5 Hz, 1H), 4.79 (*t*, *J* = 2.3 Hz, 1H). ^13^C-NMR (126 MHz, CDCl_3_): *δ* 8.0, 23.7, 26.6, 28.5, 30.6, 36.5, 43.2, 43.9, 45.7, 103.6, 161.4, 211.8.

### 3.41. Synthesis of (R)-1-(2,2-dimethyl-3-methylenecyclopentyl)pentan-2-one (***10c***)

Following *GP5*. Prepared from (*R*)-2-(2,2-dimethyl-3-methylenecyclopentyl)-*N*-methoxy-*N*-methylacetamide (**9**) (2.0 mmol, 422 mg), Et_2_O (7 mL), and propylmagnesium bromide (2.0 M in Et_2_O, 3.0 mmol, 1.5 mL); column chromatography: EtOAc/petroleum ether = 1:20. Yield: 315 mg (1.62 mmol, 81%) of colorless oil. [*α*]_D_^r.t.^ = +9.0 (0.26, CH_2_Cl_2_). EI-HRMS: *m*/*z* = 195.1743 (MH^+^); C_13_H_23_O: requires *m*/*z* = 195.1743 (MH^+^); *ν*_max_ 3071, 2960, 2873, 1711, 1651, 1462, 1409, 1363, 1285, 1198, 1125, 1026, 878, 740, 63 cm^−1^. ^1^H-NMR (500 MHz, CDCl_3_): *δ* 0.82 (*s*, 3H), 0.92 (*t*, *J* = 7.4 Hz, 3H), 1.06 (*s*, 3H), 1.19–1.30 (*m*, 1H), 1.61 (*h*, *J* = 7.3 Hz, 2H), 1.84–1.92 (*m*, 1H), 1.96–2.05 (*m*, 1H), 2.23 (*dd*, *J* = 10.6, 15.7 Hz, 1H), 2.28–2.49 (*m*, 5H), 4.77 (*t*, *J* = 2.5 Hz, 1H), 4.79 (*t*, *J* = 2.2 Hz, 1H). ^13^C-NMR (126 MHz, CDCl_3_): *δ* 13.9, 17.4, 23.8, 26.6, 28.5, 30.6, 43.6, 43.9, 45.4, 45.6, 103.6, 161.4, 211.4.

### 3.42. Synthesis of (R)-1-(2,2-dimethyl-3-methylenecyclopentyl)hexan-2-one (***10d***)

Following *GP5*. Prepared from (*R*)-2-(2,2-dimethyl-3-methylenecyclopentyl)-*N*-methoxy-*N*-methylacetamide (**9**) (2.0 mmol, 422 mg), Et_2_O (7 mL), and butylmagnesium chloride (2.0 M in Et_2_O, 3.0 mmol, 1.5 mL); column chromatography: EtOAc/petroleum ether = 1:20. Yield: 369 mg (1.77 mmol, 88%) of colorless oil. [*α*]_D_^r.t.^ = +9.0 (0.21, CH_2_Cl_2_). EI-HRMS: *m*/*z* = 209.1897 (MH^+^); C_14_H_25_O: requires *m*/*z* = 209.1900 (MH^+^); *ν*_max_ 2958, 2871, 1712, 1651, 1463, 1435, 1409, 1377, 1363, 1285, 1198, 1126, 1035, 878, 732, 634 cm^−1^. ^1^H-NMR (500 MHz, CDCl_3_): *δ* 0.82 (*s*, 3H), 0.91 (*t*, *J* = 7.4 Hz, 3H), 1.05 (*s*, 3H), 1.18–1.36 (*m*, 3H), 1.52–1.60 (*m*, 2H), 1.84–1.91 (*m*, 1H), 1.97–2.05 (*m*, 1H), 2.23 (*dd*, *J* = 10.6, 15.7 Hz, 1H), 2.28–2.49 (*m*, 5H), 4.77 (*t*, *J* = 2.6 Hz, 1H), 4.79 (*t*, *J* = 2.2 Hz, 1H). ^13^C-NMR (126 MHz, CDCl_3_): *δ* 14.0, 22.5, 23.8, 26.1, 26.6, 28.5, 30.6, 43.2, 43.6, 43.9, 45.6, 103.6, 161.4, 211.5.

### 3.43. Synthesis of (R)-1-(2,2-dimethyl-3-methylenecyclopentyl)-3-methylbutan-2-one (***10e***)

Following *GP5*. Prepared from (*R*)-2-(2,2-dimethyl-3-methylenecyclopentyl)-*N*-methoxy-*N*-methylacetamide (**9**) (2.0 mmol, 422 mg), Et_2_O (7 mL), and isopropylmagnesium bromide (2.0 M in Et_2_O, 3.0 mmol, 1.5 mL); column chromatography: EtOAc/petroleum ether = 1:20. Yield: 327 mg (1.68 mmol, 84%) of colorless oil. [*α*]_D_^r.t.^ = +8.0 (0.24, CH_2_Cl_2_). EI-HRMS: *m*/*z* = 195.1740 (MH^+^); C_13_H_23_O: requires *m*/*z* = 195.1743 (MH^+^); *ν*_max_ 2961, 2871, 1710, 1652, 1464, 1382, 1363, 1286, 1198, 1152, 1089, 1028, 878, 793, 703 cm^−1^. ^1^H-NMR (500 MHz, CDCl_3_): *δ* 0.84 (*s*, 3H), 1.06 (*s*, 3H), 1.10 (*d*, *J* = 7.0 Hz, 6H), 1.17–1.27 (*m*, 1H), 1.83–1.91 (*m*, 1H), 1.99–2.07 (*m*, 1H), 2.26–2.38 (*m*, 2H), 2.39–2.45 (*m*, 1H), 2.48 (*dd*, *J* = 3.5, 16.1 Hz, 1H), 2.63 (*hept*, *J* = 6.9 Hz, 1H), 4.77 (*d*, *J* = 2.5 Hz, 1H), 4.79 (*td*, *J* = 0.8, 2.2 Hz, 1H). ^13^C-NMR (126 MHz, CDCl_3_): *δ* 18.3, 18.4, 23.8, 26.6, 28.6, 30.6, 41.2, 41.3, 43.9, 45.4, 103.5, 161.5, 214.9.

### 3.44. Synthesis of (R)-1-(2,2-dimethyl-3-methylenecyclopentyl)pent-4-en-2-one (***10f***)

Following *GP5*. Prepared from (*R*)-2-(2,2-dimethyl-3-methylenecyclopentyl)-*N*-methoxy-*N*-methylacetamide (**9**) (2.0 mmol, 422 mg), Et_2_O (7 mL), and allylmagnesium bromide (1.0 M in THF, 3.0 mmol, 3.0 mL); column chromatography: EtOAc/petroleum ether = 1:30. Yield: 319 mg (1.66 mmol, 83%) of colorless oil. [*α*]_D_^r.t.^ = +10.0 (0.25, CH_2_Cl_2_). EI-HRMS: *m*/*z* = 193.1563 (MH^+^); C_13_H_21_O: requires *m*/*z* = 193.1587 (MH^+^); *ν*_max_ 3073, 2960, 1714, 1651, 1463, 1434, 1363, 1332, 1290, 1198, 1113, 1039, 992, 918, 878, 706, 663 cm^−1^. ^1^H-NMR (500 MHz, CDCl_3_): *δ* 0.83 (*s*, 3H), 1.05 (*s*, 3H), 1.18–1.30 (*m*, 1H), 1.83–1.94 (*m*, 1H), 1.97–2.06 (*m*, 1H), 2.27 (*dd*, *J* = 10.5, 16.0 Hz, 1H), 2.31–2.39 (*m*, 1H), 2.40–2.48 (*m*, 1H), 2.51 (*dd*, *J* = 3.5, 16.0 Hz, 1H), 3.14–3.25 (*m*, 2H), 4.77 (*t*, *J* = 2.5 Hz, 1H), 4.79 (*td*, *J* = 0.8, 2.2 Hz, 1H), 5.11–5.23 (*m*, 2H), 5.93 (*ddt*, *J* = 7.0, 10.2, 17.2 Hz, 1H). ^13^C-NMR (126 MHz, CDCl_3_): *δ* 23.7, 26.6, 28.5, 30.6, 43.2, 43.9, 45.4, 48.3, 103.6, 118.9, 130.8, 161.3, 208.8.

### 3.45. Synthesis of (R)-1-(2,2-dimethyl-3-methylenecyclopentyl)-3-phenylpropan-2-one (***10g***)

Following *GP5*. Prepared from (*R*)-2-(2,2-dimethyl-3-methylenecyclopentyl)-*N*-methoxy-*N*-methylacetamide (**9**) (2.0 mmol, 422 mg), Et_2_O (7 mL), and benzylmagnesium bromide (1.0 M in THF, 3.0 mmol, 3.0 mL); column chromatography: EtOAc/petroleum ether = 1:20. Yield: 378 mg (1.56 mmol, 78%) of colorless oil. [*α*]_D_^r.t.^ = +4.0 (0.26, CH_2_Cl_2_). EI-HRMS: *m*/*z* = 243.1745 (MH^+^); C_17_H_23_O: requires *m*/*z* = 243.1743 (MH^+^); *ν*_max_ 3066, 3029, 2959, 1712, 1651, 1602, 1495, 1454, 1434, 1363, 1286, 1200, 1108, 1031, 879, 748, 697 cm^−1^. ^1^H-NMR (500 MHz, CDCl_3_): *δ* 0.77 (*s*, 3H), 1.01 (*s*, 3H), 1.09–1.22 (*m*, 1H), 1.79–1.89 (*m*, 1H), 1.95–2.06 (*m*, 1H), 2.24–2.34 (*m*, 2H), 2.36–2.44 (*m*, 1H), 2.51 (*dd*, *J* = 3.6, 16.0 Hz, 1H), 3.71 (*s*, 2H), 4.75 (*t*, *J* = 2.5 Hz, 1H), 4.77 (*t*, *J* = 2.2 Hz, 1H), 7.17–7.23 (*m*, 2H), 7.25–7.28 (*m*, 1H), 7.30–7.36 (*m*, 2H). ^13^C-NMR (126 MHz, CDCl_3_): *δ* 23.7, 26.6, 28.4, 30.6, 42.8, 43.9, 45.5, 50.6, 103.6, 127.1, 128.8, 129.6, 134.3, 161.3, 208.4.

### 3.46. Synthesis of (R)-1-(2,2-dimethyl-3-methylenecyclopentyl)but-3-en-2-one (***10h***)

Following *GP5*. Prepared from (*R*)-2-(2,2-dimethyl-3-methylenecyclopentyl)-*N*-methoxy-*N*-methylacetamide (**9**) (2.0 mmol, 422 mg), Et_2_O (7 mL), and vinylmagnesium bromide (1.0 M in THF, 3.0 mmol, 3.0 mL); column chromatography: EtOAc/petroleum ether = 1:20. Yield: 267 mg (1.50 mmol, 75%) of colorless oil. [*α*]_D_^r.t.^ = +5.0 (0.24, CH_2_Cl_2_). EI-HRMS: *m*/*z* = 179.1424 (MH^+^); C_12_H_19_O: requires *m*/*z* = 179.1430 (MH^+^); *ν*_max_ 3071, 2959, 1681, 1651, 1614, 1463, 1434, 1399, 1363, 1332, 1292, 1194, 1149, 1112, 1068, 985, 961, 915, 878, 809, 634 cm^−1^. ^1^H-NMR (500 MHz, CDCl_3_): *δ* 0.86 (*s*, 3H), 1.08 (*s*, 3H), 1.22–1.34 (*m*, 1H), 1.83–1.93 (*m*, 1H), 1.99–2.09 (*m*, 1H), 2.28–2.50 (*m*, 3H), 2.65 (*dd*, *J* = 3.6, 15.5 Hz, 1H), 4.78 (*t*, *J* = 2.5 Hz, 1H), 4.80 (*td*, *J* = 0.8, 2.2 Hz, 1H), 5.83 (*dd*, *J* = 1.1, 10.6 Hz, 1H), 6.23 (*dd*, *J* = 1.1, 17.6 Hz, 1H), 6.38 (*dd*, *J* = 10.6, 17.6 Hz, 1H). ^13^C-NMR (75 MHz, CDCl_3_): *δ* 23.8, 26.7, 28.5, 30.6, 40.7, 44.1, 46.0, 103.7, 128.1, 136.9, 161.4, 201.0.

### 3.47. Synthesis of (R)-1-(2,2-dimethyl-3-methylenecyclopentyl)but-3-yn-2-one (***10i***)

Following *GP5*. Prepared from (*R*)-2-(2,2-dimethyl-3-methylenecyclopentyl)-*N*-methoxy-*N*-methylacetamide (**9**) (2.0 mmol, 422 mg), Et_2_O (7 mL), and ethynylmagnesium bromide (0.5 M in THF, 3.0 mmol, 6.0 mL); column chromatography: EtOAc/petroleum ether = 1:40. Yield: 282 mg (1.60 mmol, 80%) of colorless oil. [*α*]_D_^r.t.^ = +12.0 (0.297, CH_2_Cl_2_). *ν*_max_ 3258, 2960, 2092, 1678, 1652, 1463, 1435, 1364, 1285, 1237, 1198, 1112, 1070, 880, 807, 694, 649 cm^−1^. ^1^H-NMR (500 MHz, CDCl_3_): *δ* 0.85 (*s*, 3H), 1.09 (*s*, 3H), 1.20–1.38 (*m*, 1H), 1.88–1.98 (*m*, 1H), 2.08–2.18 (*m*, 1H), 2.30–2.51 (*m*, 3H), 2.67 (*dd*, *J* = 3.9, 15.6 Hz, 1H), 3.23 (*s*, 1H), 4.79 (*t*, *J* = 2.5 Hz, 1H), 4.80–4.82 (*m*, 1H). ^13^C-NMR (126 MHz, CDCl_3_): *δ* 23.7, 26.7, 28.2, 30.5, 44.0, 45.7, 46.6, 78.6, 81.8, 103.9, 160.8, 187.5.

### 3.48. Synthesis of (R)-1-(2,2-dimethyl-3-methylenecyclopentyl)pent-3-yn-2-one (***10j***)

Following *GP5*. Prepared from (*R*)-2-(2,2-dimethyl-3-methylenecyclopentyl)-*N*-methoxy-*N*-methylacetamide (**9**) (2.0 mmol, 422 mg), Et_2_O (7 mL), and 1-propynylmagnesium bromide (0.5 M in THF, 3.0 mmol, 6.0 mL); column chromatography: EtOAc/petroleum ether = 1:30. Yield: 263 mg (1.38 mmol, 69%) of colorless oil. [*α*]_D_^r.t.^ = +6.0 (0.243, CH_2_Cl_2_). EI-HRMS: *m*/*z* = 191.1430 (MH^+^); C_13_H_19_O: requires *m*/*z* = 191.1430 (MH^+^); *ν*_max_ 2960, 2218, 1669, 1463, 1435, 1364, 1330, 1286, 1258, 1175, 1143, 1021, 972, 879, 783, 680 cm^−1^. ^1^H-NMR (500 MHz, CDCl_3_): *δ* 0.84 (*s*, 3H), 1.08 (*s*, 3H), 1.18–1.41 (*m*, 1H), 1.85–1.98 (*m*, 1H), 2.01–2.03 (*m*, 3H), 2.05–2.18 (*m*, 1H), 2.24–2.53 (*m*, 3H), 2.61 (*dd*, *J* = 3.7, 15.2 Hz, 1H), 4.74–4.84 (*m*, 2H). ^13^C-NMR (75 MHz, CDCl_3_): *δ* 4.2, 23.7, 26.7, 28.3, 30.6, 44.0, 45.9, 46.6, 80.6, 90.0, 103.7, 161.2, 188.2.

### 3.49. Synthesis of (R)-2-(2,2-dimethyl-3-methylenecyclopentyl)-1-phenylethan-1-one (***10k***)

Following *GP5*. Prepared from (*R*)-2-(2,2-dimethyl-3-methylenecyclopentyl)-*N*-methoxy-*N*-methylacetamide (**9**) (2.0 mmol, 422 mg), Et_2_O (7 mL), and phenylmagnesium bromide (3.0 M in THF, 3.0 mmol, 1.0 mL); column chromatography: EtOAc/petroleum ether = 1:30. Yield: 374 mg (1.64 mmol, 82%) of colorless oil. [*α*]_D_^r.t.^ = +6.0 (0.25, CH_2_Cl_2_). EI-HRMS: *m*/*z* = 229.1594 (MH^+^); C_16_H_21_O: requires *m*/*z* = 229.1587 (MH^+^); *ν*_max_ 3068, 2959, 1682, 1651, 1597, 1580, 1462, 1448, 1363, 1333, 1286, 1205, 1180, 1147, 1075, 1001, 878, 750, 688, 646, 616 cm^−1^. ^1^H-NMR (500 MHz, CDCl_3_): *δ* 0.93 (*s*, 3H), 1.13 (*s*, 3H), 1.28–1.39 (*m*, 1H), 1.87–1.97 (*m*, 1H), 2.21–2.21 (*m*, 1H), 2.28–2.40 (*m*, 1H), 2.41–2.51 (*m*, 1H), 2.78 (*dd*, *J* = 10.5, 15.7 Hz, 1H), 3.06 (*dd*, *J* = 3.5, 15.7 Hz, 1H), 4.77–4.83 (*m*, 2H), 7.46–7.46 (*m*, 2H), 7.53–7.60 (*m*, 1H), 7.93–8.00 (*m*, 2H). ^13^C-NMR (126 MHz, CDCl_3_): *δ* 23.9, 26.8, 28.6, 30.6, 39.4, 44.2, 46.2, 103.6, 128.3, 128.7, 133.1, 137.4, 161.5, 200.6.

### 3.50. Synthesis of Secondary Alcohols ***11*** from Aldehyde ***5***—General Procedure 6 (GP6)

To a solution of aldehyde **5** (1.0 equiv.) in anhydrous Et_2_O (3 mL) under argon at 0 °C, the corresponding Grignard reagent (1.4 equiv.) was added slowly. The resulting reaction mixture was stirred at 0 °C for 1 h and then at room temperature for 20 h. The excess Grignard reagent was quenched with NaCl (aq. sat., 3 mL), and the resulting mixture was extracted with Et_2_O (3 × 10 mL). The combined organic phase was washed with NaCl (aq. sat., 2 × 5 mL), dried under anhydrous Na_2_SO_4_, filtered, and the volatiles evaporated in vacuo. The residue was purified/separated by column chromatography (Silica gel 60). The fractions containing the pure product **11** were combined, and the volatile components were evaporated in vacuo. The isolated secondary alcohols **11** were fully characterized. The two diastereomers formed could, in most cases, be partially separated by column chromatography.

### 3.51. Synthesis of 1-((R)-2,2-dimethyl-3-methylenecyclopentyl)propan-2-ol (***11a***/***11a′***)

Following *GP6*. Prepared from (*R*)-2-(2,2-dimethyl-3-methylenecyclopentyl)acetaldehyde (**5**) (91.3 mg, 0.6 mmol), Et_2_O (3 mL), and methylmagnesium bromide (3.0 M in Et_2_O, 0.84 mmol, 0.28 mL); column chromatography: EtOAc/petroleum ether = 1:5. The two diastereomers formed could be partially separated by column chromatography. Diastereomeric ratio: **11a**/**11a′** = 54:46. Diastereomer **11a** (major): Elutes first from the column. Yield: 20 mg (0.119 mmol, 20%) of colorless oil. [*α*]_D_^r.t.^ = +37.1 (0.11, CH_2_Cl_2_). EI-HRMS: *m*/*z* = 169.1586 (MH^+^); C_11_H_21_O: requires *m*/*z* = 169.1587 (MH^+^); *ν*_max_ 3351, 2960, 2929, 1711, 1461, 1375, 1363, 1068, 877 cm^−1^. ^1^H-NMR (500 MHz, CDCl_3_): *δ* 0.74 (*s*, 3H), 0.99 (*s*, 3H), 1.16 (*d*, *J* = 6.2 Hz, 3H), 1.13–1.24 (*m*, 2H), 1.32 (br *s*, 1H), 1.38–1.47 (*m*, 1H), 1.60–1.70 (*m*, 1H), 1.79–1.89 (*m*, 1H), 2.18–2.30 (*m*, 1H), 2.34–2.44 (*m*, 1H), 3.75–3.84 (*m*, 1H), 4.67–4.73 (*m*, 2H). ^13^C-NMR (126 MHz, CDCl_3_): *δ* 23.5, 24.9, 26.5, 28.2, 30.8, 39.7, 44.0, 46.4, 66.7, 103.0, 162.5. Diastereomer **11a′** (minor): Elutes secondly from the column. Yield: 15 mg (0.089 mmol, 15%; contains 24% of **11a**) of colorless oil. ^1^H-NMR (500 MHz, CDCl_3_): *δ* 0.74 (*s*, 3H), 1.16 (*d*, *J* = 6.1 Hz, 3H) (the remaining signals overlap with the signals of diastereomer **11a**).

### 3.52. Synthesis of 1-((R)-2,2-dimethyl-3-methylenecyclopentyl)pent-4-en-2-ol (***11b***/***11b′***)

Following *GP6*. Prepared from (*R*)-2-(2,2-dimethyl-3-methylenecyclopentyl)acetaldehyde (**5**) (91.3 mg, 0.6 mmol), Et_2_O (3 mL), and allylmagnesium bromide (1.0 M in THF, 0.84 mmol, 0.84 mL); column chromatography: EtOAc/petroleum ether = 1:10. The two diastereomers formed could be partially separated by column chromatography. Diastereomeric ratio: **11b**/**11b′** = 48:53. Diastereomer **11b** (minor): Elutes first from the column. Yield: 32 mg (0.1647 mmol, 27%) of colorless oil. [*α*]_D_^r.t.^ = +21.0 (0.215, CH_2_Cl_2_). EI-HRMS: *m*/*z* = 195.1745 (MH^+^); C_13_H_23_O: requires *m*/*z* = 195.1743 (MH^+^); *ν*_max_ 3366, 3073, 2958, 1710, 1462, 1434, 1362, 1067, 1016, 913, 877 cm^−1^. ^1^H-NMR (500 MHz, CDCl_3_): *δ* 0.74 (*s*, 3H), 1.00 (*s*, 3H), 1.13–1.26 (*m*, 2H), 1.39–1.53 (*m*, 2H), 1.67–1.77 (*m*, 1H), 1.80–1.89 (*m*, 1H), 2.07–2.17 (*m*, 1H), 2.19–2.31 (*m*, 2H), 2.34–2.44 (*m*, 1H), 3.60–3.68 (*m*, 1H), 4.67–4.73 (*m*, 2H), 5.04–5.12 (*m*, 2H), 5.72–5.84 (*m*, 1H). ^13^C-NMR (126 MHz, CDCl_3_): *δ* 23.5, 26.5, 28.1, 30.8, 37.2, 43.3, 44.0, 46.2, 69.2, 103.1, 118.4, 135.0, 162.6. Diastereomer **11b′** (major): Elutes secondly from the column. Yield: 30 mg (0.154 mmol, 25%; contains 19% of **11b**) of colorless oil. ^1^H-NMR (500 MHz, CDCl_3_): *δ* 0.74 (*s*, 3H), 0.99 (*s*, 3H), 1.24–1.37 (*m*, 2H), 1.41–1.54 (*m*, 2H), 1.60 (s, 1H), 1.79–1.87 (*m*, 1H), 1.99–2.07 (*m*, 1H), 2.19–2.27 (*m*, 1H), 2.27–2.35 (*m*, 1H), 2.36–2.44 (*m*, 1H), 3.60–3.70 (*m*, 1H), 4.66–4.73 (*m*, 2H), 5.03–5.12 (*m*, 2H), 5.71–5.83 (*m*, 1H). ^13^C-NMR (126 MHz, CDCl_3_): *δ* 23.4, 26.5, 29.0, 30.9, 37.2, 41.7, 44.3, 47.5, 70.4, 103.1, 118.6, 134.8, 162.2.

### 3.53. Synthesis of 1-((R)-2,2-dimethyl-3-methylenecyclopentyl)-3-methylbutan-2-ol (***11c***/***11c′***)

Following *GP6*. Prepared from (*R*)-2-(2,2-dimethyl-3-methylenecyclopentyl)acetaldehyde (**5**) (91.3 mg, 0.6 mmol), Et_2_O (3 mL), and isopropylmagnesium bromide (2.0 M in Et_2_O, 1.4 mmol, 0.7 mL); column chromatography: EtOAc/petroleum ether = 1:10. The two diastereomers formed could not be separated by column chromatography. Diastereomeric ratio: **11c**/**11c′** = 53:47. Yield: 104 mg (0.1647 mmol, 88%) of colorless oil. [*α*]_D_^r.t.^ = +25.1 (0.28, CH_2_Cl_2_). EI-HRMS: *m*/*z* = 197.1904 (MH^+^); C_13_H_25_O: requires *m*/*z* = 197.1900 (MH^+^); *ν*_max_ 3376, 2958, 2870, 1651, 1464, 1362, 1065, 994, 976, 877 cm^−1^. ^1^H-NMR (500 MHz, CDCl_3_) for both diastereomers: *δ* 0.82 (*d*, *J* = 3.8 Hz, 3H), 0.87–1.00 (*m*, 6H), 1.07 (*d*, *J* = 3.8 Hz, 3H), 1.18–1.31 (*m*, 2H), 1.34–1.47 (*m*, 1H), 1.51–1.58 (*m*, 1H), 1.60–1.81 (*m*, 2H), 1.86–1.96 (*m*, 1H), 2.23–2.37 (*m*, 1H), 2.47–2.47 (*m*, 1H), 3.37–3.55 (*m*, 1H), 4.74–4.80 (*m*, 2H). ^13^C-NMR (126 MHz, CDCl_3_) for both diastereomers: *δ* 15.8, 17.6, 18.9, 19.4, 23.5, 23.6, 26.5, 26.7, 28.0, 29.4, 30.7, 31.0, 32.8, 34.3, 34.6, 34.9, 44.0, 44.4, 46.3, 48.0, 75.1, 76.5, 103.0, 103.1, 162.5, 162.7.

### 3.54. Synthesis of 1-((R)-2,2-dimethyl-3-methylenecyclopentyl)but-3-yn-2-ol (***11d***/***11d′***)

Following *GP6*. Prepared from (*R*)-2-(2,2-dimethyl-3-methylenecyclopentyl)acetaldehyde (**5**) (91.3 mg, 0.6 mmol), Et_2_O (3 mL), and ethynylmagnesium bromide (0.5 M in THF, 0.84 mmol, 1.68 mL); column chromatography: EtOAc/petroleum ether = 1:7. The two diastereomers formed could be partially separated by column chromatography. Diastereomeric ratio: **11d**/**11d′** = 54:46. Diastereomer **11d** (major): Elutes first from the column. Yield: 26 mg (0.146 mmol, 24%) of colorless oil. [*α*]_D_^r.t.^ = +19.7 (0.14, CH_2_Cl_2_). EI-HRMS: *m*/*z* = 179.1432 (MH^+^); C_12_H_19_O: requires *m*/*z* = 179.143 (MH^+^); *ν*_max_ 3348, 3073, 2965, 1732, 1327, 1181, 1045, 892 cm^−1^. ^1^H-NMR (500 MHz, CDCl_3_): *δ* 0.76 (*s*, 3H), 1.01 (*s*, 3H), 1.21–1.33 (*m*, 1H), 1.45–1.56 (*m*, 1H), 1.68–1.83 (*m*, 3H), 1.84–1.94 (*m*, 1H), 2.19–2.31 (*m*, 1H), 2.35–2.46 (*m*, 2H), 4.33–4.39 (*m*, 1H), 4.68–4.74 (*m*, 2H). ^13^C-NMR (126 MHz, CDCl_3_): *δ* 23.57, 26.47, 28.17, 30.79, 38.57, 44.03, 45.97, 61.35, 72.92, 85.64, 103.29, 162.11. Diastereomer **11d′** (minor): Elutes secondly from the column. Yield: 46 mg (0.258 mmol, 43%; contains 17% of **11d**) of colorless oil. ^1^H-NMR (500 MHz, CDCl_3_): *δ* 0.76 (*s*, 3H), 1.02 (*s*, 3H), 1.22–1.32 (*m*, 1H), 1.50–1.58 (*m*, 1H), 1.61–1.68 (*m*, 1H), 1.70–1.77 (*m*, 1H), 1.79–1.88 (*m*, 1H), 1.96 (*s*, 1H), 2.19–2.31 (*m*, 1H), 2.35–2.48 (*m*, 2H), 4.32–4.39 (*m*, 1H), 4.68–4.76 (*m*, 2H). ^13^C-NMR (126 MHz, CDCl_3_): *δ* 23.6, 26.3, 28.3, 30.8, 38.4, 44.1, 46.9, 62.4, 73.4, 84.9, 103.3, 161.9.

### 3.55. Synthesis of Tertiary Alcohols ***12*** from Ketones ***10***—General Procedure 7 (GP7)

To a solution of ketone **10** (1.0 equiv.) in anhydrous Et_2_O (4 mL) under argon at 0 °C, Grignard reagent (2.0 equiv.) was added slowly. The resulting reaction mixture was stirred at 0 °C for 1 h and then at room temperature for 2 h. The excess Grignard reagent was quenched with NaCl (aq. sat., 3 mL), and the resulting mixture was extracted with Et_2_O (3 × 10 mL). The combined organic phase was washed with NaCl (aq. sat., 3 × 10 mL), dried under anhydrous Na_2_SO_4_, filtered, and the volatiles evaporated in vacuo. The residue was purified by column chromatography (Silica gel 60). The fractions containing the pure product **12** were combined, and the volatiles evaporated in vacuo. The isolated tertiary alcohols **12** were fully characterized (as mixtures of two diastereomers).

### 3.56. Synthesis of (R)-1-(2,2-dimethyl-3-methylenecyclopentyl)-2-methylpropan-2-ol (***12a***)

Following *GP7*. Prepared from (*R*)-1-(2,2-dimethyl-3-methylenecyclopentyl)propan-2-one (**10a**) (1.0 mmol, 166 mg), Et_2_O (4 mL), and methylmagnesium bromide (2.0 M in Et_2_O, 2.0 mmol, 1.0 mL); column chromatography: EtOAc/petroleum ether = 1:2. Yield: 144 mg (0.79 mmol, 79%) of colorless oil. [*α*]_D_^r.t.^ = +21.0 (0.13, CH_2_Cl_2_). EI-HRMS: *m*/*z* = 165.1637 (M-OH)H^+^; C_12_H_21_: requires *m*/*z* = 165.1638 (M-OH)H^+^; *ν*_max_ 3380, 3071, 2961, 1652, 1465, 1377, 1362, 1189, 1141, 1047, 955, 905, 877, 770 cm^−1^. ^1^H-NMR (500 MHz, CDCl_3_): *δ* 0.78 (*s*, 3H), 1.04 (*s*, 3H), 1.24 (*s*, 3H), 1.25 (*s*, 3H), 1.31–1.41 (*m*, 2H), 1.52–1.63 (*m*, 2H), 1.97–2.06 (*m*, 1H), 2.24–2.37 (*m*, 1H), 2.43–2.53 (*m*, 1H), 4.74–4.79 (*m*, 2H). ^13^C-NMR (126 MHz, CDCl_3_): *δ* 23.2, 25.7, 29.7, 30.4, 30.6, 31.0, 44.0, 44.7, 46.1, 71.5, 103.0, 161.9.

### 3.57. Synthesis of 1-((R)-2,2-dimethyl-3-methylenecyclopentyl)-2-methylbutan-2-ol (***12b***/***12b′***)

Following *GP7*. Prepared from (*R*)-1-(2,2-dimethyl-3-methylenecyclopentyl)butan-2-one (**10b**) (1.0 mmol, 180 mg), Et_2_O (4 mL), and methylmagnesium bromide (2.0 M in Et_2_O, 2.0 mmol, 1.0 mL); column chromatography: EtOAc/petroleum ether = 1:1. The two diastereomers formed could not be separated by column chromatography. Diastereomeric ratio: 60:40. Yield: 159 mg (0.81 mmol, 81%) of colorless oil. [*α*]_D_^r.t.^ = +26.0 (0.13, CH_2_Cl_2_). EI-HRMS: *m*/*z* = 179.1796 (M-OH)H^+^; C_13_H_23_: requires *m*/*z* = 179.1794 (M-OH)H^+^; *ν*_max_ 3394, 3070, 2961, 2927, 1652, 1461, 1377, 1362, 1297, 1138, 1054, 996, 920, 877, 849, 787, 756 cm^−1^. ^1^H-NMR (500 MHz, CDCl_3_) for both diastereomers: *δ* 0.78 (*s*, 3H), 0.91 (*q*, *J* = 7.6 Hz, 3H), 1.04 (*s*, 3H), 1.17 (*d*, *J* = 4.4 Hz, 3H), 1.30–1.45 (*m*, 2H), 1.50–1.62 (*m*, 4H), 1.96–2.06 (*m*, 1H), 2.24–2.35 (*m*, 1H), 2.43–2.53 (*m*, 1H), 4.74–4.80 (*m*, 2H) (one signal missing). ^13^C-NMR (126 MHz, CDCl_3_) for both diastereomers: *δ* 8.3, 8.6, 23.2, 23.2, 25.8, 25.8, 26.7, 27.6, 30.3, 30.4, 31.0, 34.5, 35.5, 41.5, 41.6, 44.8, 66.8, 45.5, 45.6, 73.3, 73.4, 103.0, 161.9, 162.0.

### 3.58. Synthesis of 1-((R)-2,2-dimethyl-3-methylenecyclopentyl)-2-methylpentan-2-ol (***12c***/***12c′***)

Following *GP7*. Prepared from (*R*)-1-(2,2-dimethyl-3-methylenecyclopentyl)pentan-2-one (**10c**) (1.0 mmol, 194 mg), Et_2_O (4 mL), and methylmagnesium bromide (2.0 M in Et_2_O, 2.0 mmol, 1.0 mL); column chromatography: EtOAc/petroleum ether = 1:5. The two diastereomers formed could not be separated by column chromatography. Diastereomeric ratio: 60:40. Yield: 162 mg (0.77 mmol, 77%) of colorless oil. [*α*]_D_^r.t.^ = +13.0 (0.13, CH_2_Cl_2_). EI-HRMS: *m*/*z* = 193.1952 (M-OH)H^+^; C_14_H_25_: requires *m*/*z* = 193.1951 (M-OH)H^+^; *ν*_max_ 3411, 3070, 2958, 2932, 2872, 1745, 1652, 1464, 1376, 1362, 1291, 1239, 1139, 1079, 1050, 1005, 929, 877, 775, 743 cm^−1^. ^1^H-NMR (500 MHz, CDCl_3_) for both diastereomers: *δ* 0.78 (*s*, 3H), 0.93 (*t*, *J* = 7.2 Hz, 3H), 1.03 (*s*, 3H), 1.15 (*d*, *J* = 5.0 Hz, 1H), 1.18 (*d*, *J* = 4.6 Hz, 3H), 1.29–1.59 (*m*, 8H), 1.95–2.04 (*m*, 1H), 2.24–2.36 (*m*, 1H), 2.43–2.53 (*m*, 1H), 4.74–4.79 (*m*, 2H). ^13^C-NMR (126 MHz, CDCl_3_) for both diastereomers: *δ* 14.8, 14.8, 17.2, 17.5, 23.2, 23.2, 25.7, 25.8, 27.3, 28.1, 30.3, 30.4, 31.0, 42.0, 42.1, 44.6, 44.8, 44.8, 45.5, 45.6, 45.6, 73.2, 73.3, 103.0, 161.9, 162.0.

### 3.59. Synthesis of 1-((R)-2,2-dimethyl-3-methylenecyclopentyl)-2-methylhexan-2-ol (***12d***/***12d′***)

Following *GP7*. Prepared from (*R*)-1-(2,2-Dimethyl-3-methylenecyclopentyl)hexan-2-one (**10d**) (1.0 mmol, 208 mg), Et_2_O (4 mL), and methylmagnesium bromide (2.0 M in Et_2_O, 2.0 mmol, 1.0 mL); column chromatography: EtOAc/petroleum ether = 1:5. The two diastereomers formed could not be separated by column chromatography. Diastereomeric ratio: 59:41. Yield: 153 mg (0.68 mmol, 68%) of colorless oil. [*α*]_D_^r.t.^ = +20.0 (0.25, CH_2_Cl_2_). EI-HRMS: *m*/*z* = 207.2105 (M-OH)H^+^; C_15_H_27_: requires *m*/*z* = 207.2107 (M-OH)H^+^; *ν*_max_ 3404, 2958, 2931, 2869, 1652, 1462, 1376, 1362, 1297, 1139, 1085, 1025, 936, 904, 877, 776, 730 cm^−1^. ^1^H-NMR (500 MHz, CDCl_3_) for both diastereomers: *δ* 0.78 (*s*, 3H), 0.89–0.95 (*m*, 3H), 1.04 (*s*, 3H), 1.14 (*d*, *J* = 6.0 Hz, 1H), 1.18 (*d*, *J* = 4.6 Hz, 3H), 1.28–1.60 (*m*, 10H), 1.96–2.04 (*m*, 1H), 2.24–2.37 (*m*, 1H), 2.43–2.53 (*m*, 1H), 4.74–4.79 (*m*, 2H). ^13^C-NMR (126 MHz, CDCl_3_) for both diastereomers: *δ* 14.3, 23.2, 23.2, 23.4, 25.8, 25.8, 26.2, 26.4, 27.3, 28.2, 30.4, 30.4, 31.0, 41.9, 42.0, 42.0, 43.0, 44.8, 44.8, 45.5, 45.6, 73.2, 73.2, 103.0, 161.9, 162.0.

### 3.60. Synthesis of 1-((R)-2,2-dimethyl-3-methylenecyclopentyl)-2-methylpent-4-en-2-ol (***12e***/***12e′***)

Following *GP7*. Prepared from (*R*)-1-(2,2-dimethyl-3-methylenecyclopentyl)pent-4-en-2-one (**10f**) (1.0 mmol, 192 mg), Et_2_O (4 mL), and methylmagnesium bromide (2.0 M in Et_2_O, 2.0 mmol, 1.0 mL); column chromatography: EtOAc/petroleum ether = 1:10. The two diastereomers formed could not be separated by column chromatography. Diastereomeric ratio: 54:46. Yield: 148 mg (0.71 mmol, 71%) of colorless oil. [*α*]_D_^r.t.^ = +4.5 (0.105, CH_2_Cl_2_). EI-HRMS: *m*/*z* = 191.1791 (M-OH)H^+^; C_14_H_24_O: requires *m*/*z* = 191.1794 (M-OH)H^+^; *ν*_max_ 3428, 3073, 2960, 2927, 1651, 1461, 1434, 1376, 1362, 1293, 1114, 998, 913, 877, 781, 709, 632 cm^−1^. ^1^H-NMR (500 MHz, CDCl_3_) for both diastereomers: *δ* 0.78 (*s*, 3H), 1.04 (*s*, 3H), 1.20 (*d*, *J* = 5.3 Hz, 3H), 1.29–1.42 (*m*, 3H), 1.54–1.65 (*m*, 2H), 1.96–2.08 (*m*, 1H), 2.23–2.35 (*m*, 3H), 2.42–2.54 (*m*, 1H), 4.74–4.79 (*m*, 2H), 5.09–5.20 (*m*, 2H), 5.80–5.95 (*m*, 1H). ^13^C-NMR (126 MHz, CDCl_3_) for both diastereomers: *δ* 23.2, 23.2, 25.8, 25.8, 27.0, 28.0, 30.3, 30.4, 31.0, 42.1, 42.1, 44.8, 44.9, 45.5, 45.7, 46.7, 47.6, 72.5, 72.6, 103.0, 103.0, 119.0, 119.1, 134.1, 134.2, 161.8, 161.9.

### 3.61. Synthesis of 1-((R)-2,2-dimethyl-3-methylenecyclopentyl)-2-phenylpropan-2-ol (***12f***/***12f′***)

Following GP7. Prepared from (*R*)-2-(2,2-dimethyl-3-methylenecyclopentyl)-1-phenylethan-1-one (10k) (1.0 mmol, 228 mg), Et_2_O (4 mL), and methylmagnesium bromide (2.0 M in Et_2_O, 2.0 mmol, 1.0 mL); column chromatography: EtOAc/petroleum ether = 1:10. The two diastereomers formed could not be separated by column chromatography. Diastereomeric ratio: 57:43. Yield: 178 mg (0.73 mmol, 73%) of colorless oil. [α]_D_^r.t.^ = +12.0 (0.11, CH_2_Cl_2_). EI-HRMS: *m*/*z* = 227.1798 (M-OH)H^+^; C_17_H_23_: requires *m*/*z* = 227.1794 (M-OH)H^+^; ν_max_ 3439, 3065, 2959, 1651, 1494, 1446, 1362, 1111, 1068, 1028, 936, 910, 877, 848, 765, 723, 699 cm^−1^. ^1^H-NMR (500 MHz, CDCl_3_) for both diastereomers: δ 0.75 (s, 1.5H), 0.76 (s, 1.5H), 0.88 (s, 1.5H), 0.98 (s, 1.5H), 1.02–1.13 (m, 0.5H), 1.23–1.39 (m, 1.5H), 1.48–1.55 (m, 0.5H), 1.59 (d, J = 3.5 Hz, 3H), 1.63–1.75 (m, 2.5H), 1.90 (dd, J = 1.8, 14.0 Hz, 0.5H), 1.98 (dd, J = 1.3, 14.4 Hz, 0.5H), 2.07–2.20 (m, 1H), 2.27–2.43 (m, 1H), 4.66–4.74 (m, 2H), 7.19–7.26 (m, 1H), 7.30–7.36 (m, 2H), 7.39–7.47 (m, 2H). ^13^C-NMR (126 MHz, CDCl_3_) for both diastereomers: δ 23.3, 23.3, 25.4, 25.6, 29.7, 29.9, 30.8, 30.9, 30.9, 31.6, 44.6, 44.7, 44.7, 44.9, 45.5, 45.8, 74.7, 75.4, 102.8, 102.9, 124.9, 125.0, 126.6, 126.7, 128.2, 128.2, 148.0, 148.6, 161.8, 161.9.

### 3.62. Synthesis of Ethyl-(R,E)-4-(2,2-dimethyl-3-methylenecyclopentyl)but-2-enoate (***13***)

To a suspension of NaH (0.825 mmol, ω = 0.60, 33 mg) in anhydrous THF (2 mL) under argon at 0 °C, triethyl phosphonoacetate (0.55 mmol, 110 μL) was added. The resulting reaction mixture was stirred at 0 °C for 45 min, then a solution of (*R*)-2-(2,2-dimethyl-3-methylenecyclopentyl)acetaldehyde (**5**) (84 mg, 0.55 mmol) in anhydrous THF (1.5 mL) was added. The reaction mixture was stirred at 0 °C for 1 h and then at room temperature for 20 h. The excess NaH was quenched with NaCl (aq. sat., 3 mL), and the mixture was extracted with EtOAc (3 × 10 mL). The combined organic phase was washed with NaCl (aq. sat., 2 × 5 mL), dried under anhydrous Na_2_SO_4_, filtered, and the volatiles evaporated in vacuo. The residue was purified by column chromatography (Silica gel 60; EtOAc/petroleum ether = 1:30). The fractions containing the pure product **13** were combined, and the volatiles were evaporated in vacuo. Yield: 80 mg (0.360 mmol, 65%) of colorless oil. [*α*]_D_^r.t.^ = +8.8 (0.13, CH_2_Cl_2_). EI-HRMS: *m*/*z* = 223.1691 (MH^+^); C_14_H_23_O_2_: requires *m*/*z* = 223.1693 (MH^+^); *ν*_max_ 2958, 1719, 1653, 1463, 1184, 1145, 1043, 978, 878 cm^−1^. ^1^H-NMR (500 MHz, CDCl_3_): *δ* 0.78 (*s*, 3H), 1.01 (*s*, 3H), 1.14–1.28 (*m*, 1H), 1.22 (*t*, *J* = 7.2 Hz, 3H), 1.52–1.62 (*m*, 1H), 1.72–1.82 (*m*, 1H), 1.87–1.97 (*m*, 1H), 2.17–2.32 (*m*, 2H), 2.33–2.43 (*m*, 1H), 4.12 (*q*, *J* = 7.1 Hz, 2H), 4.70 (*t*, *J* = 2.5 Hz, 1H), 4.72 (*dt*, *J* = 1.1, 2.1 Hz, 1H), 5.77 (*dt*, *J* = 1.5, 15.6 Hz, 1H), 6.90 (*ddd*, *J* = 6.9, 7.9, 15.6 Hz, 1H). ^13^C-NMR (126 MHz, CDCl_3_): *δ* 14.4, 23.5, 27.0, 28.2, 30.6, 33.2, 44.2, 49.5, 60.3, 103.6, 122.0, 149.1, 161.9, 166.8.

### 3.63. Synthesis of Methyl (R,E)-4-(2,2-dimethyl-3-methylenecyclopentyl)but-2-enoate (***14***)

To a solution of (*R*)-2-(2,2-dimethyl-3-methylenecyclopentyl)acetaldehyde (**5**) (45.7 mg, 0.3 mmol) in anhydrous toluene (2 mL) under argon at room temperature, methyl (triphenylphosphoranylidene)acetate (0.45 mmol, 150 mg) was added. The resulting reaction mixture was heated under reflux for 24 h. The volatile components were evaporated in vacuo. The residue was purified by column chromatography (Silica gel 60; EtOAc/petroleum ether = 1:20). The fractions containing the pure product **14** were combined, and the volatiles were evaporated in vacuo. Yield: 47 mg (0.2256 mmol, 75%) of colorless oil. [*α*]_D_^r.t.^ = +5.8 (0.135, CH_2_Cl_2_). EI-HRMS: *m*/*z* = 209.1536 (MH^+^); C_13_H_21_O_2_: requires *m*/*z* = 209.1536 (MH^+^); *ν*_max_ 2956, 1723, 1655, 1435, 1269 1194, 1041, 978, 878 cm^−1^. ^1^H-NMR (500 MHz, CDCl_3_): *δ* 0.78 (*s*, 3H), 1.01 (*s*, 3H), 1.17–1.29 (*m*, 1H), 1.52–1.62 (*m*, 1H), 1.72–1.81 (*m*, 1H), 1.87–1.98 (*m*, 1H), 2.17–2.31 (*m*, 2H), 2.33–2.43 (*m*, 1H), 3.66 (*s*, 3H), 4.70 (*t*, *J* = 2.5 Hz, 1H), 4.71–4.74 (*m*, 1H), 5.78 (*dt*, *J* = 1.5, 15.6 Hz, 1H), 6.91 (*ddd*, *J* = 7.0, 7.9, 15.6 Hz, 1H). ^13^C-NMR (126 MHz, CDCl_3_): *δ* 23.5, 27.0, 28.2, 30.6, 33.2, 44.2, 49.5, 51.6, 103.6, 121.6, 149.4, 161.9, 167.2.

### 3.64. Synthesis of (R,E)-5-(2,2-dimethyl-3-methylenecyclopentyl)pent-3-en-2-one (***15***)

To a solution of (*R*)-2-(2,2-dimethyl-3-methylenecyclopentyl)acetaldehyde (**5**) (45.7 mg, 0.3 mmol) in anhydrous CH_2_Cl_2_ (2 mL) under argon at room temperature, 1-triphenylphosphoranyliden-2-propanon (0.45 mmol, 143 mg) was added. The resulting reaction mixture was heated under reflux for 24 h. The volatile components were evaporated in vacuo. The residue was purified by column chromatography (Silica gel 60; EtOAc/petroleum ether = 1:30). The fractions containing the pure product **15** were combined, and the volatiles were evaporated in vacuo. Yield: 32 mg (0.166 mmol, 56%) of colorless oil. [*α*]_D_^r.t.^ = +2.2 (0.275, CH_2_Cl_2_). EI-HRMS: *m*/*z* = 193.1586 (MH^+^); C_13_H_21_O: requires *m*/*z* = 193.1587 (MH^+^); *ν*_max_ 2958, 1698, 1627, 1361, 1251, 977, 878 cm^−1^. ^1^H-NMR (500 MHz, CDCl_3_): *δ* 0.80 (*s*, 3H), 1.02 (*s*, 3H), 1.19–1.30 (*m*, 1H), 1.55–1.63 (*m*, 1H), 1.71–1.80 (*m*, 1H), 1.90–2.01 (*m*, 1H), 2.18 (*s*, 3H), 2.21–2.32 (*m*, 2H), 2.35–2.43 (*m*, 1H), 4.71 (*t*, *J* = 2.5 Hz, 1H), 4.73 (*dt*, *J* = 1.1, 2.2 Hz, 1H), 6.03 (*dt*, *J* = 1.4, 15.8 Hz, 1H), 6.74 (*ddd*, *J* = 6.8, 7.7, 15.9 Hz, 1H). ^13^C-NMR (126 MHz, CDCl_3_): *δ* 23.5, 27.0, 27.0, 28.3, 30.5, 33.5, 44.2, 49.6, 103.6, 132.0, 148.2, 161.7, 198.7.

### 3.65. Synthesis of (R,E)-(3-(2,2-dimethyl-3-methylenecyclopentyl)prop-1-en-1-yl)benzene (***16***) and (R,Z)-(3-(2,2-dimethyl-3-methylenecyclopentyl)prop-1-en-1-yl)benzene (***16′***)

To a suspension of NaH (0.45 mmol, ω = 0.60, 18 mg) in anhydrous THF (0.5 mL) under argon at 0 °C, benzyltriphenylphosphonium chloride (0.3 mmol, 117 mg) was added. The resulting reaction mixture was stirred at 0 °C for 30 min, then a solution of (*R*)-2-(2,2-dimethyl-3-methylenecyclopentyl)acetaldehyde (**5**) (0.3 mmol, 45.6 mg) in anhydrous THF (1.0 mL) was added. The reaction mixture was stirred at 0 °C for 1 h and then at room temperature for 20 h. The excess NaH was quenched with NaCl (aq. sat., 3 mL), and the mixture was extracted with EtOAc (3 × 10 mL). The combined organic phase was washed with NaCl (aq. sat., 2 × 5 mL), dried under anhydrous Na_2_SO_4_, filtered, and the volatiles evaporated in vacuo. The residue was purified by column chromatography (Silica gel 60; petroleum ether). The fractions containing the pure products **16**/**16′** were combined, and the volatiles were evaporated in vacuo. Diastereomeric ratio: **16**/**16′** = 88:12. The two diastereomers formed could not be separated by column chromatography. Yield: 43 mg (0.190 mmol, 63%) of colorless oil. [*α*]_D_^r.t.^ = +38.8 (0.22, CH_2_Cl_2_). EI-HRMS: the product was not ionized; *ν*_max_ 2957, 1651, 1495, 1384, 878, 742, 692 cm^−1^. ^1^H-NMR (500 MHz, CDCl_3_) for **16**: *δ* 0.82 (*s*, 3H), 1.04 (*s*, 3H), 1.23–1.34 (*m*, 1H), 1.53–1.63 (*m*, 1H), 1.77–1.84 (*m*, 1H), 1.89–1.99 (*m*, 1H), 2.18–2.32 (*m*, 2H), 2.34–2.43 (*m*, 1H), 4.69–4.73 (*m*, 2H), 6.15 (*ddd*, *J* = 6.8, 7.5, 15.7 Hz, 1H), 6.33 (*dt*, *J* = 1.5, 15.7 Hz, 1H), 7.10–7.14 (*m*, 1H), 7.20–7.24 (*m*, 2H), 7.26–7.29 (*m*, 2H). ^13^C-NMR (126 MHz, CDCl_3_) for **16**: *δ* 23.5, 27.1, 28.4, 30.7, 33.9, 44.2, 50.6, 103.2, 126.1, 127.0, 128.6, 130.4, 130.7, 138.0, 162.7. ^1^H-NMR (500 MHz, CDCl_3_) for **16′**: *δ* 0.74 (*s*, 3H), 1.02 (*s*, 3H), 2.02–2.09 (*m*, 1H), 4.67–4.69 (*m*, 1H), 5.64 (*dt*, *J* = 7.3, 11.7 Hz, 1H) (the remaining signals overlap with the signals of diastereomer **16**).

### 3.66. Cyclopropanation of the Methylene Group—General Procedure 8 (GP8)

Zinc dust (84.0 mg, 1.285 mmol, 2.57 equiv.) and copper(I) chloride (127 mg, 1.285 mmol, 2.57 equiv.) were introduced into a flame-dried flask under argon, followed by the addition of Et_2_O (3 mL). The resulting mixture was refluxed under argon for 30 min. Diiodomethane (53 μL, 0.65 mmol, 1.3 equiv.) was then added. The reaction mixture turned dark in color, and bubbles began to form. After 5–10 min, alkene (**8a** or **9**) (0.5 mmol) and further diiodomethane (243 μL, 3.0 mmol, 6 equiv.) were added. The resulting reaction mixture was refluxed under argon for 20 h. The cooled reaction mixture (room temperature) was filtered through a plague of Celite^®^ and washed with Et_2_O (30 mL) to remove the solid particles. The organic phase was washed with HCl (aq., 1 M, 2 × 15 mL), H_2_O (2 × 15 mL) and NaCl (aq. sat., 3 × 15 mL). The organic phase was dried under anhydrous Na_2_SO_4_, filtered, and the volatiles evaporated in vacuo. The residue was purified by column chromatography (Silica gel 60). The fractions containing the pure product were combined, and the volatile components were evaporated in vacuo. The isolated cyclopropanated products **17** and **18** were fully characterized.

### 3.67. Synthesis of (R)-5-(2-methoxyethyl)-4,4-dimethylspiro[2.4]heptane (***17***)

Following *GP8*. Prepared from (*R*)-2-(2-methoxyethyl)-1,1-dimethyl-5-methylenecyclopentane (**8a**) (0.5 mmol, 84 mg); column chromatography: EtOAc/petroleum ether = 1:50. Yield: 67 mg (0.367 mmol, 73%) of colorless oil. The product contains 9% of the starting alkene **8a**. [*α*]_D_^r.t.^ = +43.3 (0.155, CH_2_Cl_2_). EI-HRMS: the product was not ionized; *ν*_max_ 2935, 2867, 1468, 1386, 1117, 1070, 841 cm^−1^. ^1^H-NMR (500 MHz, CDCl_3_): *δ* 0.05–0.13 (*m*, 1H), 0.25–0.34 (*m*, 2H), 0.49–0.56 (*m*, 1H), 0.57 (*s*, 3H), 0.70 (*s*, 3H), 1.29–1.41 (*m*, 3H), 1.63–1.82 (*m*, 2H), 1.83–1.96 (*m*, 2H), 3.34 (*s*, 3H), 3.36–3.47 (*m*, 2H). ^13^C-NMR (126 MHz, CDCl_3_): *δ* 6.5, 12.3, 20.2, 22.9, 28.3, 30.7, 30.8, 34.0, 40.9, 47.3, 58.7, 72.6.

### 3.68. Synthesis of (R)-2-(4,4-dimethylspiro[2.4]heptan-5-yl)-N-methoxy-N-methylacetamide (***18***)

Following *GP8*. Product **18** was prepared on a 3 mmol scale. Prepared from zinc dust (504 mg, 7.71 mmol, 2.57 equiv.), copper(I) chloride (763 mg, 7.71 mmol, 2.57 equiv.), Et_2_O (10 mL), diiodomethane (316 μL, 3.9 mmol, 1.3 equiv.), (*R*)-2-(2,2-dimethyl-3-methylenecyclopentyl)-*N*-methoxy-*N*-methylacetamide (**9**) (3.0 mmol, 634 mg), and diiodomethane (1.461 mL, 18 mmol, 6 equiv.) and washed with Et_2_O (60 mL); column chromatography: EtOAc/petroleum ether = 1:5. Yield: 460 mg (2.04 mmol, 68%) of colorless oil. The product contains 5% of the starting alkene **9**. [*α*]_D_^r.t.^ = +36.2 (0.175, CH_2_Cl_2_). EI-HRMS: *m*/*z* = 226.1800 (MH^+^); C_13_H_24_NO_2_: requires *m*/*z* = 226.1802 (MH^+^); *ν*_max_ 2957, 2869, 1663, 1464, 1412, 1176, 1111, 1004 cm–1. ^1^H-NMR (500 MHz, CDCl_3_): *δ* 0.09–0.17 (*m*, 1H), 0.31 (*dd*, *J* = 7.0, 8.4 Hz, 2H), 0.47–0.58 (*m*, 1H), 0.62 (*s*, 3H), 0.73 (*s*, 3H), 1.30–1.47 (*m*, 2H), 1.80–1.91 (*m*, 1H), 1.96–2.07 (*m*, 1H), 2.14–2.24 (*m*, 1H), 2.26–2.34 (*m*, 1H), 2.49 (*dd*, *J* = 3.6, 14.6 Hz, 1H), 3.19 (*s*, 3H), 3.70 (*s*, 3H). ^13^C-NMR (126 MHz, CDCl_3_): *δ* 6.9, 12.0, 20.4, 22.9, 28.6, 30.4, 32.4, 33.4, 33.8, 40.9, 46.4, 61.3, 174.9.

### 3.69. Synthesis of Ketones ***19*** from Weinreb Amide ***18***—General Procedure 9 (GP9)

To a solution of (*R*)-2-(4,4-dimethylspiro[2.4]heptan-5-yl)-*N*-methoxy-*N*-methylacetamide (**18**) (1.0 equiv.; contains 5% of alkene **9**) in anhydrous Et_2_O (5 mL) under argon at 0 °C, the corresponding Grignard reagent (1.6 equiv.) was added slowly. The resulting reaction mixture was stirred at 0 °C for 1 h and then at room temperature for 20 h. The excess Grignard reagent was quenched with NaCl (aq. sat., 3 mL), and the resulting mixture was extracted with Et_2_O (3 × 15 mL). The combined organic phase was washed with NaCl (aq. sat., 3 × 5 mL), dried under anhydrous Na_2_SO_4_, filtered, and the volatiles evaporated in vacuo. The residue was purified by column chromatography (Silica gel 60). The fractions containing the pure product **19** were combined, and the volatiles evaporated in vacuo. The isolated ketones **19** were fully characterized.

### 3.70. Synthesis of (R)-1-(4,4-dimethylspiro[2.4]heptan-5-yl)propan-2-one (***19a***)

Following *GP9*. Prepared from (*R*)-2-(4,4-dimethylspiro[2.4]heptan-5-yl)-*N*-methoxy-*N*-methylacetamide (**18**) (0.5 mmol, 113 mg), Et_2_O (5 mL), and methylmagnesium bromide (3 M in Et_2_O, 0.8 mmol, 267 μL); column chromatography: EtOAc/petroleum ether = 1:20. Yield: 80 mg (0.444 mmol, 88%) of colorless oil. The product contains 4% of the alkene **9**. [*α*]_D_^r.t.^ = +16.2 (0.28, CH_2_Cl_2_). EI-HRMS: *m*/*z* = 181.1586 (MH^+^); C_12_H_21_O: requires *m*/*z* = 181.1587 (MH^+^); *ν*_max_ 2958, 2869, 1714, 1468, 1365, 1287, 1163, 1145, 1011 cm^−1^. ^1^H-NMR (500 MHz, CDCl_3_): *δ* 0.09–0.17 (*m*, 1H), 0.27–0.36 (*m*, 2H), 0.50–0.57 (*m*, 1H), 0.59 (*s*, 3H), 0.70 (*s*, 3H), 1.22–1.34 (*m*, 1H), 1.37–1.46 (*m*, 1H), 1.83–1.93 (*m*, 1H), 1.91–2.03 (*m*, 1H), 2.08–2.15 (*m*, 1H), 2.17 (*s*, 3H), 2.28 (*dd*, *J* = 10.7, 15.5 Hz, 1H), 2.51 (*dd*, *J* = 3.5, 15.6 Hz, 1H). ^13^C-NMR (126 MHz, CDCl_3_): *δ* 6.8, 12.0, 20.4, 23.0, 28.5, 30.3, 30.4, 33.7, 40.9, 45.6, 46.0, 209.7.

### 3.71. Synthesis of (R)-1-(4,4-dimethylspiro[2.4]heptan-5-yl)pent-4-en-2-one (***19b***)

Following *GP9*. Prepared from (*R*)-2-(4,4-dimethylspiro[2.4]heptan-5-yl)-*N*-methoxy-*N*-methylacetamide (**18**) (0.5 mmol, 113 mg), Et_2_O (5 mL), and allylmagnesium bromide (1.0 M in Et_2_O, 0.8 mmol, 0.8 mL); column chromatography: EtOAc/petroleum ether = 1:50. Yield: 73 mg (0.354 mmol, 70%) of colorless oil. The product contains 3% of the alkene **9**. [*α*]_D_^r.t.^ = +21.1 (0.25, CH_2_Cl_2_). EI-HRMS: *m*/*z* = 207.1784 (MH^+^); C_14_H_23_O: requires *m*/*z* = 207.1785 (MH^+^); *ν*_max_ 2958, 2869, 1714, 1638, 1468, 1385, 1365, 1012, 991, 917 cm^−1^. ^1^H-NMR (500 MHz, CDCl_3_): *δ* 0.09–0.16 (*m*, 1H), 0.26–0.36 (*m*, 2H), 0.50–0.57 (*m*, 1H), 0.58 (*s*, 3H), 0.69 (*s*, 3H), 1.20–1.34 (*m*, 1H), 1.37–1.46 (*m*, 1H), 1.82–1.91 (*m*, 1H), 1.92–2.03 (*m*, 1H), 2.09–2.19 (*m*, 1H), 2.30 (*dd*, *J* = 10.7, 15.8 Hz, 1H), 2.52 (*dd*, *J* = 3.5, 15.8 Hz, 1H), 3.14–3.26 (*m*, 2H), 5.11–5.22 (*m*, 2H), 5.94 (*ddt*, *J* = 7.0, 10.2, 17.2 Hz, 1H). ^13^C-NMR (126 MHz, CDCl_3_): *δ* 6.8, 12.0, 20.4, 23.0, 28.5, 30.3, 33.7, 40.9, 44.2, 45.8, 48.2, 118.8, 130.9, 209.2.

### 3.72. Synthesis of (R)-1-(4,4-dimethylspiro[2.4]heptan-5-yl)but-3-en-2-one (***19c***)

Following *GP9*. Prepared from (*R*)-2-(4,4-dimethylspiro[2.4]heptan-5-yl)-*N*-methoxy-*N*-methylacetamide (**18**) (0.5 mmol, 113 mg), Et_2_O (5 mL), and vinylmagnesium bromide (1.0 M in THF, 0.8 mmol, 0.8 mL); column chromatography: EtOAc/petroleum ether = 1:40. Yield: 65 mg (0.338 mmol, 67%) of colorless oil. [*α*]_D_^r.t.^ = +36.6 (0.155, CH_2_Cl_2_). EI-HRMS: *m*/*z* = 193.1584 (MH^+^); C_13_H_21_O: requires *m*/*z* = 193.1587 (MH^+^); *ν*_max_ 2958, 2869, 1680, 1615, 1400, 1365, 1011, 985, 958 cm^−1^. ^1^H-NMR (500 MHz, CDCl_3_): *δ* 0.10–0.17 (*m*, 1H), 0.28–0.36 (*m*, 2H), 0.51–0.58 (*m*, 1H), 0.61 (*s*, 3H), 0.73 (*s*, 3H), 1.24–1.36 (*m*, 1H), 1.37–1.46 (*m*, 1H), 1.83–1.90 (*m*, 1H), 1.91–2.01 (*m*, 1H), 2.12–2.22 (*m*, 1H), 2.44 (*dd*, *J* = 10.7, 15.2 Hz, 1H), 2.67 (*dd*, *J* = 3.7, 15.2 Hz, 1H), 5.81 (*dd*, *J* = 1.2, 10.6 Hz, 1H), 6.23 (*dd*, *J* = 1.2, 17.6 Hz, 1H), 6.39 (*dd*, *J* = 10.6, 17.6 Hz, 1H). ^13^C-NMR (126 MHz, CDCl_3_): *δ* 6.9, 12.1, 20.4, 23.0, 28.5, 30.3, 33.7, 41.0, 41.5, 46.2, 129.0, 136.9, 201.4.

### 3.73. Synthesis of (R)-2-(4,4-dimethylspiro[2.4]heptan-5-yl)-1-phenylethan-1-one (***19d***)

Following *GP9*. Prepared from (*R*)-2-(4,4-dimethylspiro[2.4]heptan-5-yl)-*N*-methoxy-*N*-methylacetamide (**18**) (0.5 mmol, 113 mg), Et_2_O (5 mL), and phenylmagnesium bromide (3.0 M in THF, 0.8 mmol, 267 μL); column chromatography: EtOAc/petroleum ether = 1:20. Yield: 76 mg (0.315 mmol, 63%) of colorless oil. The product contains 3% of the alkene **9**. [*α*]_D_^r.t.^ = +40.1 (0.235, CH_2_Cl_2_). EI-HRMS: *m*/*z* = 243.1744 (MH^+^); C_17_H_23_O: requires *m*/*z* = 243.1743 (MH^+^); *ν*_max_ 2957, 2868, 1682, 1448, 1365, 1286, 1213, 1045, 750, 689 cm^−1^. ^1^H-NMR (500 MHz, CDCl_3_): *δ* 0.10–0.18 (*m*, 1H), 0.29–0.37 (*m*, 2H), 0.51–0.59 (*m*, 1H), 0.66 (*s*, 3H), 0.80 (*s*, 3H), 1.29–1.46 (*m*, 2H), 1.83–1.94 (*m*, 1H), 2.02–2.04 (*m*, 1H), 2.23–2.34 (*m*, 1H), 2.80 (*dd*, *J* = 10.5, 15.4 Hz, 1H), 3.08 (*dd*, *J* = 3.5, 15.5 Hz, 1H), 7.41–7.51 (*m*, 2H), 7.52–7.62 (*m*, 1H), 7.93–8.00 (*m*, 2H). ^13^C-NMR (126 MHz, CDCl_3_): *δ* 6.9, 12.1, 20.6, 23.1, 28.6, 30.4, 33.8, 40.3, 41.2, 46.5, 128.3, 128.7, 133.0, 137.5, 200.9.

### 3.74. Synthesis of (3R)-1-methoxy-3-(2-methoxyethyl)-1,2,2-trimethylcyclopentane (***20***)

To a solution/suspension of Hg(OAc)_2_ (0.65 mmol, 207 mg) in anhydrous methanol (1.5 mL) at 0 °C under argon was added (*R*)-2-(2-methoxyethyl)-1,1-dimethyl-5-methylenecyclopentane (**8a**) (0.5 mmol, 84 mg). After stirring at 0 °C for 15 min, NaOH (aq., 3 M, 0.5 mL) was added. The reaction mixture turned from colorless to yellow. Then, a solution/suspension of NaBH_4_ (5.25 mmol, 199 mg) in NaOH (aq., 3 M, 0.5 mL) was added. The reaction mixture changed color from yellow to dark blue (black). The reaction mixture was stirred at room temperature under argon for 20 h. The reaction mixture was filtered through a pad of Celite^®^ and washed with EtOAc (20 mL). The filtrate was washed with H_2_O (2 × 5 mL) and NaCl (aq. sat., 2 × 5 mL), dried under anhydrous Na_2_SO_4_, filtered, and the volatiles evaporated in vacuo. The residue was purified by column chromatography (Silica gel 60, EtOAc/petroleum ether = 1:10). The fractions containing the pure product **20** were combined, and the volatile components were evaporated in vacuo. Yield: 66 mg (0.330 mmol, 66%) of colorless oil. [*α*]_D_^r.t.^ = +25.2 (0.15, CH_2_Cl_2_). EI-HRMS: the product was not ionized; *ν*_max_ 2935, 2867, 1468, 1386, 1117, 1070, 841 cm^−1^. ^1^H-NMR (500 MHz, CDCl_3_): *δ* 0.67 (*s*, 3H), 0.87 (*s*, 3H), 1.06 (*s*, 3H), 1.15–1.24 (*m*, 1H), 1.28–1.37 (*m*, 1H), 1.37–1.46 (*m*, 1H), 1.64–1.88 (*m*, 2H), 1.90–2.06 (*m*, 2H), 3.13 (*s*, 3H), 3.33 (*s*, 3H), 3.35–3.43 (*m*, 2H). ^13^C-NMR (126 MHz, CDCl_3_): *δ* 16.0, 19.6, 19.7, 27.4, 30.0, 31.1, 43.1, 47.6, 49.4, 58.6, 73.0, 87.7.

### 3.75. Synthesis of (3aR,6aS)-6,6,6a-trimethylhexahydro-2H-cyclopenta[b]furan (***21***) [40]

To a solution of (*R*)-2-(2,2-dimethyl-3-methylenecyclopentyl)ethan-1-ol (**4**) (2.0 mmol, 308 mg) in anhydrous Et_2_O (4 mL) under argon at 0 °C was added TFA (4 mL). The resulting reaction mixture was stirred at 0 °C for 1 h and at room temperature for 1 h. The volatiles were thoroughly evaporated in vacuo. The residue was purified by column chromatography (Silica gel 60, EtOAc/petroleum ether = 1:20). The fractions containing the pure product **21** were combined, and the volatile components were evaporated in vacuo. The isolated ether **21** was fully characterized with the exception of HRMS, as the product was not ionized. Yield: 118 mg (0.76 mmol, 38%) of colorless oil. [*α*]_D_^r.t.^ = +5.0 (0.23, CH_2_Cl_2_). *ν*_max_ 2955, 2864, 1785, 1460, 1398, 1348, 1218, 1141, 943, 821, 776, 731 cm^−1^. ^1^H-NMR (500 MHz, CDCl_3_): *δ* 0.85 (*s*, 3H), 1.01 (*s*, 3H), 1.08 (*s*, 3H), 1.15–1.25 (*m*, 1H), 1.28–1.36 (*m*, 1H), 1.61–1.70 (*m*, 2H), 1.91–2.03 (*m*, 1H), 2.12–2.22 (*m*, 1H), 2.32–2.40 (*m*, 1H), 3.70–3.79 (*m*, 1H), 3.89 (*td*, *J* = 3.6, 8.2 Hz, 1H). ^13^C-NMR (126 MHz, CDCl_3_): *δ* 19.1, 22.2, 25.5, 29.7, 34.9, 39.8, 46.0, 47.2, 68.0, 95.1.

### 3.76. DFT Calculations

Theoretical studies were carried out using AMS 2025.1, SCM, Theoretical Chemistry program [66]. Geometry optimizations of relevant equilibrium structures were performed using the M06-2X [67,68] density functional with the TZ2P basis set as implemented in ADF–AMS 2025.1. Normal mode vibrational analysis on the stationary points allowed us to confirm they are minima (zero imaginary frequencies). The M06-2X functional was chosen, as it is better suited to handling kinetics, thermodynamics, and noncovalent interactions in organic molecular systems [67,69,70,71,72,73,74]. Solvation effect (in dichloromethane) was considered using the COSMO model. An ultrafine grid was employed for all DFT calculations. Unless otherwise stated, relative Gibbs energies (ΔG) reported correspond to the M06-2X/TZ2P level at 298.13 K in dichloromethane. The 3D geometries were generated using GUI of AMS 2025.1.

## 4. Conclusions

The availability of (+)-isocampholenic acid from (1*S*)-(+)-10-camphorsulfonic acid in only two steps at a scale of 172 mmol and 60% yield enabled the first systematic exploration of its chemical space, with diversification at both the carboxylic acid and exocyclic C=C bond. Using established functional group transformations provided a total of 60 products, i.e., esters, ethers, ketones, secondary and tertiary alcohols, enones and selected cyclopropanated analogs. In most cases, a small amount of α-campholenic acid-derived isomer was detected by proton NMR (up to 2%). As shown by DFT calculations, isocampholenic acid is up to 5.9 kcal/mol less stable than its endocyclic isomers. This suggests that targeted stabilization, such as early introduction of a spirocyclopropyl group or other methylene modifications, may further improve stability. Preliminary odor evaluations by an untrained panel revealed diverse scent profiles, underscoring the untapped potential of this scaffold. The present work only begins to chart the chemical and olfactory landscape of isocampholenic acid, offering numerous opportunities for future functionalization and fragrance design.

## Data Availability

The original contributions presented in this study are included in the article. Further inquiries can be directed to the corresponding author.

## Supplementary Materials

The following supporting information can be downloaded at: https://www.mdpi.com/article/10.3390/molecules30183794/s1, Copies of ^1^H- and ^13^C-NMR spectra; DFT calculation; olfactory properties and chemical shifts.

## Abbreviations

The following abbreviations are used in this manuscript:

CDI	1,1′-Carbonyldiimidazole
DFT	Density functional theory
DMAP	4-(Dimethylamino)pyridine
EDCI	*N*-(3-Dimethylaminopropyl)-*N*′-ethylcarbodiimide hydrochloride
THF	Tetrahydrofuran
