# Supplementary material for: Isocampholenic Acid Derivatives as Potential Fragrances
Source: Molecules. 2025 Sep 18;30(18):3794. doi: 10.3390/molecules30183794 (PMC12472598; doi:10.3390/molecules30183794)
Supplement: Supplementary file 1 [file molecules-30-03794-s001.zip › molecules-3871955-supplementary.pdf]

SUPPORTING INFORMATION

Isocampholenic acid derivatives as potential fragrances

Luka Ciber, Tjaša Koželj, Kaja Prosenak, Nejc Petek, Franc Požgan, Jurij Svete, Bogdan Štefane and Uroš Grošelj *

Chair of Organic Chemistry, Faculty of Chemistry and Chemical Technology, University of Ljubljana, Večna pot 113, 1000 Ljubljana, Slovenia; E-mail: uros.groselj@fkkt.uni-lj.si

Table of contents

1. Materials and methods, syntheses, and characterization 2
2. Copies of ^{1}H- and ^{13}C-NMR spectra 77
3. DFT calculation 145
4. Olfactory properties and chemical shifts 153
5. References 159

1. Materials and methods, syntheses, and characterization

Solvents for extractions and chromatography were of technical grade and were distilled prior to use. Extracts were dried over technical grade anhydrous Na_2SO_4. The NMR spectra were obtained on a Bruker Avance DPX 300 at 300 MHz for ^1H and 75.5 MHz for ^{13}C nucleus, and on a Bruker UltraShield 500 plus (Bruker, Billerica, Massachusetts, United States) at 500 MHz for ^1H and 126 MHz for ^{13}C nucleus, using $CDCl_3$ with TMS as the internal standard, as solvent. Mass spectra were recorded on an Agilent 6224 Accurate Mass TOF LC/MS (Agilent Technologies, Santa Clara, California, United States), IR spectra on a Perkin-Elmer Spectrum BX FTIR spectrophotometer (PerkinElmer, Waltham, Massachusetts, United States). Column chromatography (CC) was performed on silica gel (Silica gel 60, particle size: 0.035-0.070 mm (Sigma-Aldrich, St. Louis, Missouri, United States)). All the commercially available chemicals used were purchased from Sigma-Aldrich (St. Louis, Missouri, United States).

3-Carboxypyridinium dichromate (NDC) was freshly prepared and used following the literature procedure.[1,2]

Synthesis of (1*R*,4*R*)-1-(iodomethyl)-7,7-dimethylbicyclo[2.2.1]heptan-2-one (2)[3]

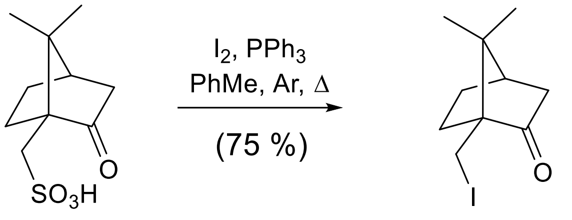

1 **2**

Compound **2** was prepared according to the procedure described in the literature.[3] To a solution of (1*S*)-(+)-10-camphorsulfonic acid (**1**) (1.0 equiv., 172 mmol, 39.95 g) in anhydrous toluene (250 mL) under argon, PPh_3 (4.0 equiv., 688 mmol, 180.46 g) and I_2 (2.0 equiv., 344 mmol, 87.31 g) were added. The resulting reaction mixture was heated for 16 hours under reflux. The volatile components were evaporated *in vacuo*. The residue was dissolved in EtOAc (500 mL) and washed with Na_2SO_3 (aq. sat., 5×100 mL or until the reaction mixture changed color from dark violet to light yellow) and NaCl (aq. sat., 3×50 mL). The organic phase was dried under anhydrous Na_2SO_4, filtered and the volatile components were evaporated *in vacuo*. The solid residue was suspended in petroleum ether (or hexane) (500 mL) and stirred for 12 h at room temperature. The solid residue was filtered, washed with petroleum ether (or hexane) (2×50 mL) and the volatile components (filtrate) were evaporated *in vacuo* to obtain 10-iodocamphor **2**. Yield: 37.77 g (ω = 0.95, 129 mmol, 75 %) of yellowish solid. ^{1}H-NMR (500 MHz, $CDCl_3$): *δ* 0.90 (*s*, 3H), 1.08 (*s*, 3H), 1.35 – 1.43 (*m*, 1H), 1.57 – 1.64 (*m*, 1H), 1.91 (*d*, *J* = 18.4 Hz, 1H), 1.95 – 2.05 (*m*, 2H), 2.16 (*t*, *J* = 4.2 Hz, 1H), 2.40 (*dd*, *J* = 4.9, 18.4 Hz, 1H), 3.12 (*d*, *J* = 10.6 Hz, 1H), 3.31 (*d*, *J* = 10.6 Hz, 1H). ^{13}C-NMR (126 MHz, $CDCl_3$): *δ* 0.83, 20.26, 20.47, 26.87, 30.69, 43.11, 44.22, 48.45, 59.19, 215.14.

Synthesis of (*R*)-2-(2,2-dimethyl-3-methylenecyclopentyl)acetic acid (3)[4]

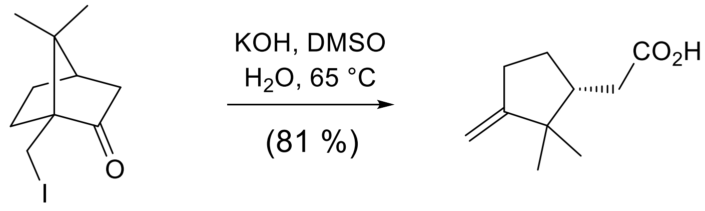

2 **3**

Compound **3** was prepared according to the procedure described in the literature.[4] To a solution of 10-iodocamphor (**2**) (1.0 equiv., ω = 0.95, 42.9 mmol, 12.56 g) in a mixture of DMSO (120 mL) and H_2O (40 mL) was added KOH (5.0 equiv., 214.5 mmol, 12.04 g). The resulting reaction mixture was stirred at 65 °C for 2 hours. NaCl (aq. sat., 100 mL) was added to the cooled reaction mixture (room temperature), followed by extraction with Et_2O (3×100 mL). The organic phase was discarded and the aqueous phase was carefully acidified with HCl (6 M in H_2O, 180 mmol, 30 mL) to pH 1-2, followed by extraction with EtOAc (3×100 mL). The combined organic phase was dried under anhydrous Na_2SO_4, filtered and the volatiles evaporated *in vacuo* to give acid **3**. Product **3** was stored under argon in the absence of light. Yield: 5.85 g (34.75 mmol, 81 %) of yellowish oil. ^{1}H-NMR (500 MHz, $CDCl_3$): δ 0.85 (*s*, 3H), 1.08 (*s*, 3H), 1.32 – 1.44 (*m*, 1H), 1.91 – 2.05 (*m*, 2H), 2.15 (*dd*, *J* = 10.4, 14.9 Hz, 1H), 2.28 – 2.39 (*m*, 1H), 2.40 – 2.52 (*m*, 2H), 4.78 (*s*, 1H), 4.80 (*s*, 1H), 11.34 (br *s*, 1H). ^{13}C-NMR (126 MHz, $CDCl_3$): δ 23.50, 26.67, 28.39, 30.45, 35.21, 43.94, 46.45, 103.83, 161.06, 180.53.

Synthesis of (*R*)-2-(2,2-dimethyl-3-methylenecyclopentyl)ethan-1-ol (4)[5]

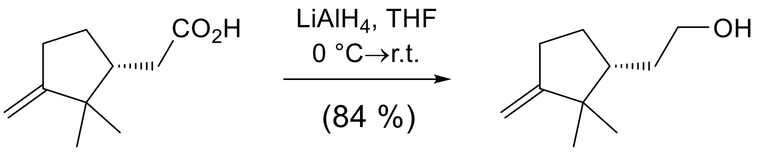

3 **4**

A solution of (+)-isocampholenic acid (**3**) (1.0 equiv., 25.1 mmol, 4.22 g) in anhydrous THF (200 mL) was cooled to 0 °C under argon, followed by a careful addition of $LiAlH_4$ (1 M in THF, 2.0 equiv., 50.2 mmol, 50.2 mL). The resulting reaction mixture was stirred at 0 °C for 1 hour and then at room temperature for 20 hours. The reaction mixture was cooled to 0 °C and then cooled H_2O (0 °C, 100 mL) was carefully added dropwise to quench the excess $LiAlH_4$. After stirring the reaction mixture at room temperature for 15 minutes, most of the THF was evaporated *in vacuo*. The residue was extracted with Et_2O (100 mL). The organic phase was washed with NaCl (aq. sat., 3×100 mL), dried under anhydrous Na_2SO_4, filtered and the volatiles evaporated *in vacuo* to give alcohol **4**. Yield: 3.255 g (21.1 mmol, 84 %) of a white solid. $[\alpha]_D^{r.t.}$ = +21.0 (0.30, CH_2Cl_2). EI-HRMS: *m/z* = 155.1429 (MH^+); $C_{10}H_{19}O$ requires: *m/z* = 155.1430 (MH^+); ν_{max} 3332, 3071, 2958, 2866, 1651, 1462, 1433, 1378, 1362, 1199, 1151, 1055, 1038, 999, 976, 921, 877, 850 cm^{-1}. ^{1}H-NMR (500 MHz, $CDCl_3$): δ 0.83 (*s*, 3H), 1.06 (*s*, 3H), 1.24 – 1.32 (*m*, 1H), 1.34 – 1.42 (*m*, 1H), 1.54 – 1.62 (*m*, 1H), 1.67 (br *s*, 1H), 1.69 – 1.76 (*m*, 1H), 1.82 – 1.88 (*m*, 1H), 2.25 – 2.34 (*m*, 1H), 2.42 – 2.50 (*m*, 1H), 3.60 – 3.68 (*m*, 1H), 3.70 – 3.79 (*m*, 1H), 4.75 – 4.79 (*m*, 2H). ^{13}C-NMR (126 MHz, $CDCl_3$): δ 23.52, 26.58, 28.31, 30.83, 33.19, 44.03, 46.85, 62.45, 103.12, 162.35.

Synthesis of (*R*)-2-(2,2-dimethyl-3-methylenecyclopentyl)acetaldehyde (5)[6]

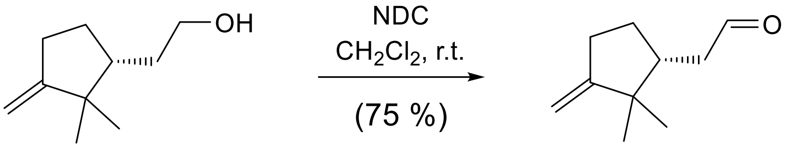

4 **5**

To a solution of alcohol **4** (1.0 equiv., 5.5 mmol, 848 mg) in anhydrous CH_2Cl_2 (15 mL) was added freshly prepared 3-carboxypyridinium dichromate (NDC)[1,2] (1.857 g, 4 mmol, 0.727 equiv.). The resulting reaction mixture was stirred at room temperature for 20 hours. The reaction mixture was filtered through a short plug of Silica gel 60 and the plug was washed with CH_2Cl_2 (2×15 mL). The volatiles were evaporated *in vacuo* and the residue was purified by column chromatography (Silica gel 60, petroleum ether/EtOAc = 20:1). The fractions containing the pure product **5** were combined and the volatile components were evaporated *in vacuo*. Yield: 630 mg (4.14 mmol, 75 %) of a colorless oil. ^{1}H-NMR (500 MHz, $CDCl_3$): δ 0.85 (*s*, 3H), 1.08 (*s*, 3H), 1.28 – 1.39 (*m*, 1H), 1.88 – 1.97 (*m*, 1H), 2.01 – 2.10 (*m*, 1H), 2.25 (*ddd*, *J* = 2.7, 10.3, 16.0 Hz, 1H), 2.31 – 2.41 (*m*, 1H), 2.44 – 2.54 (*m*, 2H), 4.79 (*t*, *J* = 2.5 Hz, 1H), 4.81 (*td*, *J* = 0.7, 2.2 Hz, 1H), 9.81 (*dd*, *J* = 1.8, 2.6 Hz, 1H). ^{13}C-NMR (126 MHz, $CDCl_3$): δ 23.46, 26.42, 28.66, 30.59, 43.85, 44.23, 44.87, 103.79, 160.68, 202.73.

Synthesis of esters 6 from (+)-isocampholenic acid – ***General procedure 1 (GP1)***

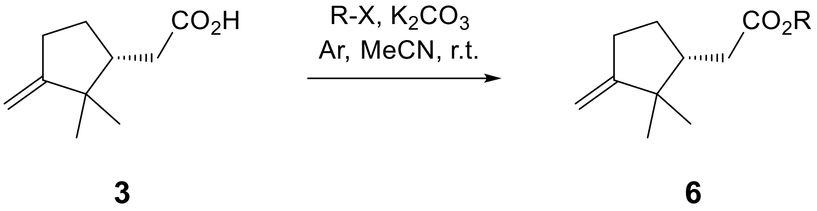

To a solution of (+)-isocampholenic acid (**3**) (1.0 equiv.) in anhydrous MeCN (2 mL) under argon, K_2CO_3 (2.0 equiv.) and the corresponding aliphatic halide were added. The resulting reaction mixture was stirred at room temperature for 20 hours. The volatiles were evaporated *in vacuo* and the residue was purified by column chromatography (Silica gel 60). The fractions containing the pure product **6** were combined and the volatiles were evaporated *in vacuo*. The isolated esters **6** were fully characterized.

Synthesis of esters 6 from (+)-isocampholenic acid – ***General procedure 2 (GP2)***

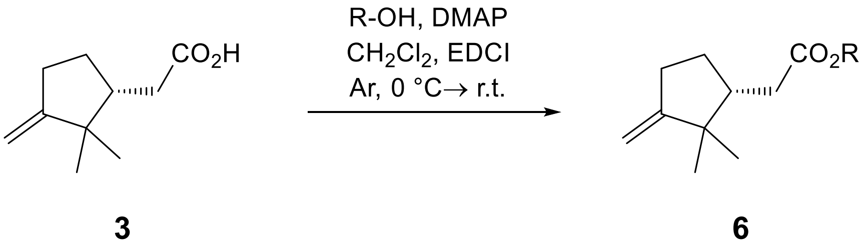

To a solution of (+)-isocampholenic acid (**3**) (1.0 equiv.) in anhydrous CH_2Cl_2 (7 mL) under argon, the corresponding alcohol and DMAP (0.1 equiv.) were added. The reaction mixture was cooled to –5 °C and then EDCI (2.0 equiv.) was added. The resulting reaction mixture was stirred at –5 °C for 1 hour and then at room temperature for 20 hours. The volatiles were evaporated *in vacuo*. The residue was dissolved in EtOAc (50 mL) and washed with $NaHSO_4$ (aq., 1 M, 2×10 mL), $NaHCO_3$ (aq. sat., 2×10 mL) and NaCl (aq. sat., 3×10 mL). The organic phase was dried under anhydrous Na_2SO_4, filtered and the volatile components evaporated *in vacuo*. The residue was purified by column chromatography (Silica gel 60). The fractions containing the pure product **6** were combined and the volatiles were evaporated *in vacuo*. The isolated esters **6** were fully characterized.

Synthesis of methyl (*R*)-2-(2,2-dimethyl-3-methylenecyclopentyl)acetate (6a)[7]

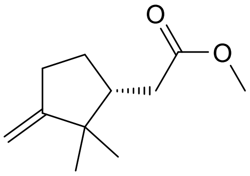

Following *GP1*. Prepared from (+)-isocampholenic acid (**3**) (1.0 mmol, 168 mg), K_2CO_3 (2.0 mmol, 276 mg), MeCN (2 mL) and iodomethane (1.5 mmol, 49 µL); column chromatography: EtOAc/petroleum ether = 1:10. Yield: 154 mg (0.85 mmol, 85 %) of colorless oil. $[\alpha]_D^{r.t.}$ = +21.0 (0.16, CH_2Cl_2). EI-HRMS: *m/z* = 183.1379 (MH^+); $C_{11}H_{19}O_2$ requires: *m/z* = 183.1380 (MH^+); ν_{max} 2958, 1737, 1652, 1435, 1364, 1291, 1253, 1195, 1143, 1060, 999, 880 cm^{-1}. ^1H-NMR (500 MHz, $CDCl_3$): δ 0.84 (*s*, 3H), 1.07 (*s*, 3H), 1.29 – 1.41 (*m*, 1H), 1.87 – 1.94 (*m*, 1H), 1.96 – 2.04 (*m*, 1H), 2.12 (*dd*, *J* = 10.5, 14.7 Hz, 1H), 2.28 – 2.50 (*m*, 3H), 3.68 (*s*, 3H), 4.77 (*t*, *J* = 2.5 Hz, 1H), 4.80 (*td*, *J* = 0.8, 2.2 Hz, 1H). ^{13}C-NMR (126 MHz, $CDCl_3$): δ 23.51, 26.66, 28.43, 30.48, 35.12, 43.92, 46.67, 51.63, 103.71, 161.34, 174.20.

Synthesis of ethyl (*R*)-2-(2,2-dimethyl-3-methylenecyclopentyl)acetate (6b)[5]

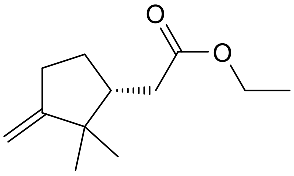

Following *GP1*. Prepared from (+)-isocampholenic acid (**3**) (1.0 mmol, 168 mg), K_2CO_3 (2.0 mmol, 276 mg), MeCN (2 mL) and bromoethane (1.5 mmol, 114 µL); column chromatography: EtOAc/petroleum ether = 1:10. Yield: 174 mg (0.89 mmol, 89 %) of colorless oil. $[\alpha]_D^{r.t.}$ = +7.0 (0.30, CH_2Cl_2). EI-HRMS: *m/z* = 197.1535 (MH^+); $C_{12}H_{21}O_2$ requires: *m/z* = 197.1536 (MH^+); ν_{max} 2961, 1733, 1652, 1464, 1365, 1290, 1252, 1182, 1140, 1031, 879 cm^{-1}. ^{1}H-NMR (500 MHz, $CDCl_3$): δ 0.84 (*s*, 3H), 1.07 (*s*, 3H), 1.26 (*t*, *J* = 7.1 Hz, 3H), 1.30 – 1.41 (*m*, 1H), 1.87 – 1.94 (*m*, 1H), 1.96 – 2.04 (*m*, 1H), 2.11 (*dd*, *J* = 10.5, 14.6 Hz, 1H), 2.28 – 2.50 (*m*, 3H), 4.14 (*q*, *J* = 7.1 Hz, 2H), 4.77 (*t*, *J* = 2.5 Hz, 1H), 4.79 (*td*, *J* = 0.7, 2.1 Hz, 1H). ^{13}C-NMR (126 MHz, $CDCl_3$): δ 14.36, 23.52, 26.67, 28.39, 30.49, 35.40, 43.93, 46.67, 60.36, 103.64, 161.40, 173.73.

Synthesis of propyl (*R*)-2-(2,2-dimethyl-3-methylenecyclopentyl)acetate (6c)

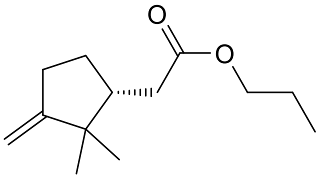

Following *GP1*. Prepared from (+)-isocampholenic acid (**3**) (1.0 mmol, 168 mg), K_2CO_3 (2.0 mmol, 276 mg), MeCN (2 mL) and 1-bromopropane (1.2 mmol, 111 µL); column chromatography: EtOAc/petroleum ether = 1:20. Yield: 184 mg (0.87 mmol, 87 %) of colorless oil. $[\alpha]_D^{r.t.}$ = +8.0 (0.26, CH_2Cl_2). EI-HRMS: *m/z* = 211.1694 (MH^+); $C_{13}H_{23}O_2$ requires: *m/z* = 211.1693 (MH^+); ν_{max} 2962, 1734, 1654, 1446, 1365, 1293, 1255, 1186, 1035, 879 cm^{-1}. ^1H-NMR (500 MHz, $CDCl_3$): δ 0.84 (*s*, 3H), 0.95 (*t*, *J* = 7.4, 3H), 1.07 (*s*, 3H), 1.31 – 1.41 (*m*, 1H), 1.66 (*h*, *J* = 7.2 Hz, 2H), 1.86 – 1.94 (*m*, 1H), 1.96 – 2.04 (*m*, 1H), 2.12 (*dd*, *J* = 10.5, 14.6 Hz, 1H), 2.29 – 2.50 (*m*, 3H), 4.04 (*t*, *J* = 6.7 Hz, 2H), 4.78 (*t*, *J* = 2.5 Hz, 1H), 4.79 – 4.81 (*m*, 1H). ^{13}C-NMR (126 MHz, $CDCl_3$): δ 10.61, 22.11, 23.58, 26.67, 28.43, 30.52, 35.42, 43.97, 46.70, 66.12, 103.69, 161.50, 173.99.

Synthesis of butyl (*R*)-2-(2,2-dimethyl-3-methylenecyclopentyl)acetate (6d)

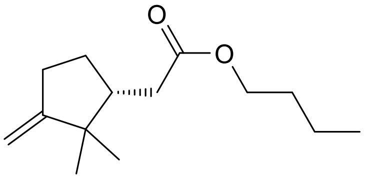

Following *GP1*. Prepared from (+)-isocampholenic acid (**3**) (1.0 mmol, 168 mg), K_2CO_3 (2.0 mmol, 276 mg), MeCN (2 mL) and 1-iodobutane (1.2 mmol, 138 μL); column chromatography: EtOAc/petroleum ether = 1:20. Yield: 192 mg (0.85 mmol, 85 %) of colorless oil. $[\alpha]_D^{r.t.}$ = +8.0 (0.29, CH_2Cl_2). EI-HRMS: *m/z* = 225.1847 (MH^+); $C_{14}H_{25}O_2$ requires: *m/z* = 225.1849 (MH^+); ν_{max} 2959, 1734, 1652, 1464, 1364, 1289, 1249, 1179, 1141, 1063, 1020, 879 cm^{-1}. ^{1}H-NMR (500 MHz, $CDCl_3$): δ 0.84 (*s*, 3H), 0.94 (*t*, *J* = 7.3 Hz, 3H), 1.07 (*s*, 3H), 1.33 – 1.44 (*m*, 3H), 1.58 – 1.66 (*m*, 2H), 1.86 – 1.94 (m, 1H), 1.96 – 2.04 (*m*, 1H), 2.11 (*dd*, *J* = 10.5, 14.6 Hz, 1H), 2.28 – 2.50 (*m*, 3H), 4.08 (*t*, *J* = 6.7 Hz, 2H), 4.77 (*t*, *J* = 2.3 Hz, 1H), 4.80 (*t*, *J* = 1.8 Hz, 1H). ^{13}C-NMR (126 MHz, $CDCl_3$): δ 13.85, 19.31, 23.55, 26.70, 28.44, 30.52, 30.82, 35.44, 43.97, 46.73, 64.36, 103.67, 161.47, 173.90.

Synthesis of isopropyl (*R*)-2-(2,2-dimethyl-3-methylenecyclopentyl)acetate (6e)

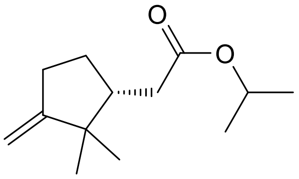

Following *GP2*. Prepared from (+)-isocampholenic acid (**3**) (1.0 mmol, 168 mg), propan-2-ol (2.0 mmol, 121 μL), DMAP (0.1 mmol, 12.2 mg), CH_2Cl_2 (7 mL) in EDCI (2.0 mmol, ω = 98 %, 391 mg); column chromatography: EtOAc/petroleum ether = 1:20. Yield: 178 mg (0.84 mmol, 84 %) of colorless oil. $[\alpha]_D^{r.t.}$ = +11.0 (0.33, CH_2Cl_2). EI-HRMS: *m/z* = 211.1693 (MH^+); $C_{13}H_{23}O_2$ requires: *m/z* = 211.1693 (MH^+); ν_{max} 2961, 1729, 1652, 1467, 1374, 1290, 1181, 1143, 1108, 959, 879 cm^{-1}. ^{1}H-NMR (500 MHz, $CDCl_3$): *δ* 0.83 (*s*, 3H), 1.07 (*s*, 3H), 1.24 (*dd*, *J* = 1.4, 6.3 Hz, 6H), 1.30 – 1.41 (*m*, 1H), 1.86 – 1.93 (*m*, 1H), 1.96 – 2.03 (*m*, 1H), 2.08 (*dd*, *J* = 10.5, 14.3 Hz, 1H), 2.29 – 2.39 (*m*, 2H), 2.41 – 2.49 (*m*, 1H), 4.77 (*t*, *J* = 2.5 Hz, 1H), 4.78 – 4.80 (*m*, 1H), 4.98 – 5.06 (*m*, 1H). ^{13}C-NMR (126 MHz, $CDCl_3$): *δ* 21.96, 22.01, 23.57, 26.72, 28.34, 30.52, 35.78, 43.98, 46.75, 67.60, 103.63, 161.51, 173.30.

Synthesis of isobutyl (*R*)-2-(2,2-dimethyl-3-methylenecyclopentyl)acetate (6f)

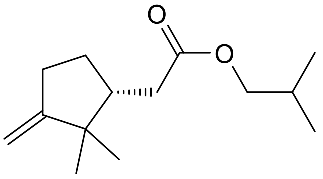

Following *GP2*. Prepared from (+)-isocampholenic acid (**3**) (1.0 mmol, 168 mg), butan-2-ol (2.0 mmol, 187 µL), DMAP (0.1 mmol, 12.2 mg), CH_2Cl_2 (7 mL) in EDCI (2.0 mmol, ω = 98 %, 391 mg); column chromatography: EtOAc/petroleum ether = 1:25. Yield: 169 mg (0.75 mmol, 75 %) of colorless oil. $[\alpha]_D^{r.t.}$ = +15.0 (0.34, CH_2Cl_2). EI-HRMS: *m/z* = 225.1849 (MH^+); $C_{14}H_{25}O_2$ requires: *m/z* = 225.1849 (MH^+); ν_{max} 2960, 1734, 1652, 1468, 1379, 1289, 1250, 1177, 1139, 1001, 879, 671 cm^{-1}. ^{1}H-NMR (500 MHz, $CDCl_3$): δ 0.84 (*s*, 3H), 0.94 (*d*, *J* = 6.8 Hz, 6H), 1.08 (*s*, 3H), 1.30 – 1.43 (*m*, 1H), 1.87 – 2.06 (*m*, 3H), 2.12 (*dd*, *J* = 10.5, 14.6 Hz, 1H), 2.28 – 2.51 (*m*, 3H), 3.86 (*d*, *J* = 6.7 Hz, 2H), 4.78 (*t*, *J* = 2.5 Hz, 1H), 4.80 (*td*, *J* = 0.7, 2.3 Hz, 1H). ^{13}C-NMR (126 MHz, $CDCl_3$): δ 19.25, 23.52, 26.68, 27.82, 28.46, 30.48, 35.41, 43.95, 46.74, 70.62, 103.67, 161.38, 173.82.

Synthesis of allyl (*R*)-2-(2,2-dimethyl-3-methylenecyclopentyl)acetate (6g)[8]

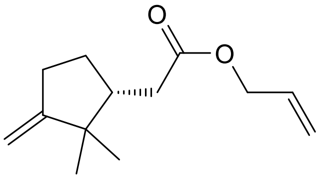

Following *GP1*. Prepared from (+)-isocampholenic acid (**3**) (1.0 mmol, 168 mg), K_2CO_3 (2.0 mmol, 276 mg), MeCN (2 mL) and 3-bromopropene (1.2 mmol, 107 µL); column chromatography: EtOAc/petroleum ether = 1:10. Yield: 149 mg (0.71 mmol, 71 %) of colorless oil. $[\alpha]_D^{r.t.}$ = +10.0 (0.25, CH_2Cl_2). EI-HRMS: *m/z* = 209.1536 (MH^+); $C_{13}H_{21}O_2$ requires: *m/z* = 209.1536 (MH^+); ν_{max} 2960, 1735, 1651, 1462, 1364, 1289, 1176, 1139, 1057, 987, 929, 879 cm^{-1}. ^{1}H-NMR (500 MHz, $CDCl_3$): δ 0.84 (*s*, 3H), 1.07 (*s*, 3H), 1.31 – 1.41 (*m*, 1H), 1.87 – 1.96 (*m*, 1H), 1.97 – 2.07 (*m*, 1H), 2.15 (*dd*, *J* = 10.6, 14.7 Hz, 1H), 2.29 – 2.39 (*m*, 1H), 2.40 – 2.51 (*m*, 2H), 4.58 (*t*, *J* = 1.4 Hz, 1H), 4.59 (*t*, *J* = 1.4 Hz, 1H), 4.78 (*t*, *J* = 2.5 Hz, 1H), 4.80 (*td*, *J* = 0.7, 2.2 Hz, 1H), 5.24 (*dq*, *J* = 1.3, 10.4 Hz, 1H), 5.33 (*dq*, *J* = 1.5, 17.2 Hz, 1H), 5.93 (*ddt*, *J* = 5.8, 10.4, 17.2 Hz, 1H). ^{13}C-NMR (126 MHz, $CDCl_3$): δ 23.55, 26.69, 28.43, 30.50, 35.32, 43.97, 46.66, 65.18, 103.72, 118.39, 132.38, 161.34, 173.39.

Synthesis of benzyl (*R*)-2-(2,2-dimethyl-3-methylenecyclopentyl)acetate (6h)

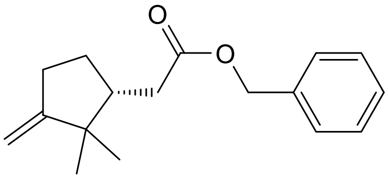

Following *GP2*. Prepared from (+)-isocampholenic acid (**3**) (1.0 mmol, 168 mg), benzyl alcohol (1.8 mmol, 189 µL), DMAP (0.1 mmol, 12.2 mg), CH_2Cl_2 (7 mL) in EDCI (2.0 mmol, ω = 98 %, 391 mg); column chromatography: EtOAc/petroleum ether = 1:30. Yield: 216 mg (0.83 mmol, 83 %) of colorless oil. $[\alpha]_D^{r.t.}$ = +9.0 (0.27, CH_2Cl_2). EI-HRMS: *m/z* = 259.1698 (MH^+); $C_{17}H_{23}O_2$ requires: *m/z* = 259.1693 (MH^+); ν_{max} 2959, 1733, 1652, 1456, 1363, 1289, 1255, 1137, 971, 880, 735, 696 cm^{-1}. ^{1}H-NMR (500 MHz, $CDCl_3$): δ 0.80 (*s*, 3H), 1.02 (*s*, 3H), 1.29 – 1.41 (*m*, 1H), 1.85 – 1.94 (*m*, 1H), 1.98 – 2.08 (*m*, 1H), 2.17 (*dd*, *J* = 10.6, 14.8 Hz, 1H), 2.30 – 2.37 (*m*, 1H), 2.39 – 2.50 (*m*, 2H), 4.77 (*t*, *J* = 2.3 Hz, 1H), 4.79 (*t*, *J* = 1.8 Hz, 1H), 5.12 (*s*, 2H), 7.30 – 7.40 (*m*, 5H). ^{13}C-NMR (126 MHz, $CDCl_3$): δ 23.55, 26.67, 28.41, 30.49, 35.37, 43.97, 46.66, 66.33, 103.72, 128.33, 128.37, 128.67, 136.13, 161.31, 173.56.

Synthesis of dodecyl (*R*)-2-(2,2-dimethyl-3-methylenecyclopentyl)acetate (6i)

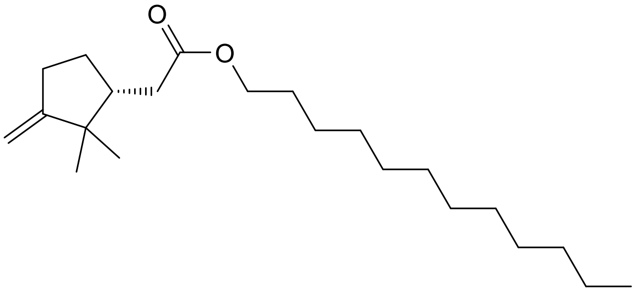

Following *GP1*. Prepared from (+)-isocampholenic acid (**3**) (1.0 mmol, 168 mg), K_2CO_3 (2.0 mmol, 276 mg), MeCN (2 mL) and 1-bromododecane (1.5 mmol, ω = 0.97, 371 μL); column chromatography: EtOAc/petroleum ether = 1:30. Yield: 188 mg (0.56 mmol, 56 %) of colorless oil. $[\alpha]_D^{r.t.}$ = +6.2 (0.121, CH_2Cl_2). EI-HRMS: *m/z* = 337.3099 (MH^+); $C_{22}H_{41}O_2$ requires: *m/z* = 337.3101 (MH^+); ν_{max} 2923, 2854, 1736, 1652, 1465, 1363, 1289, 1178, 1141, 880, 722 cm^{-1}. ^1H-NMR (500 MHz, $CDCl_3$): δ 0.84 (*s*, 3H), 0.88 (*t*, *J* = 6.9 Hz, 3H), 1.07 (*s*, 3H), 1.20 – 1.41 (*m*, 19H), 1.58 – 1.67 (*m*, 2H), 1.85 – 1.94 (*m*, 1H), 1.95 – 2.06 (*m*, 1H), 2.11 (*dd*, *J* = 10.5 Hz, 14.6, 1H), 2.28 – 2.51 (*m*, 3H), 4.07 (*t*, *J* = 6.8 Hz, 2H), 4.77 (*t*, *J*=2.6, 1H), 4.79 – 4.81 (*m*, 1H). ^{13}C-NMR (126 MHz, $CDCl_3$): δ 14.27, 22.84, 23.57, 26.09, 26.72, 28.46, 28.77, 29.38, 29.49, 29.67, 29.71, 29.78, 29.79, 30.53, 32.06, 35.46, 43.98, 46.75, 64.67, 103.68, 161.48, 173.90.

Synthesis of (*R*)-3,7-dimethyloct-6-en-1-yl 2-((*R*)-2,2-dimethyl-3-methylenecyclopentyl)acetate (6j)

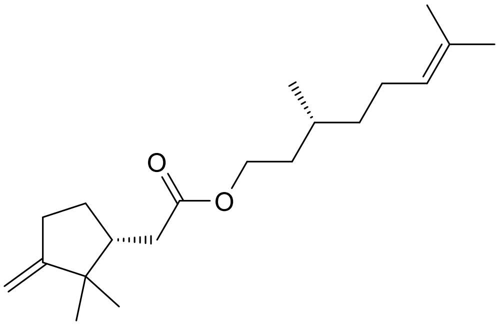

Following *GP2*. Prepared from (+)-isocampholenic acid (**3**) (1.0 mmol, 168 mg), (*R*)-(+)-β-citronellol (1.1 mmol, ω = 0.95, 211 μL), DMAP (0.1 mmol, 12.2 mg), CH_2Cl_2 (7 mL) in EDCI (2.0 mmol, ω = 98 %, 391 mg); column chromatography: EtOAc/petroleum ether = 1:30. Yield: 242 mg (0.79 mmol, 79 %) of colorless oil. $[\alpha]_D^{r.t.}$ = +6.8 (0.272, CH_2Cl_2). EI-HRMS: *m/z* = 307.2635 (MH^+); $C_{20}H_{35}O_2$ requires: *m/z* = 307.2632 (MH^+); ν_{max} 2959, 1735, 1652, 1460, 1378, 1290, 1178, 1140, 1058, 982, 880 cm^{-1}. ^{1}H-NMR (500 MHz, $CDCl_3$): δ 0.84 (*s*, 3H), 0.92 (*d*, *J* = 6.6 Hz, 3H), 1.07 (*s*, 3H), 1.14 – 1.23 (*m*, 1H), 1.31 – 1.40 (*m*, 2H), 1.41 – 1.48 (*m*, 1H), 1.51 – 1.59 (*m*, 1H), 1.60 (*s*, 3H), 1.63 – 1.72 (*m*, 1H), 1.68 (*s*, 3H), 1.86 – 2.05 (*m*, 4H), 2.10 (*dd*, *J* = 10.5, 14.6 Hz, 1H), 2.28 – 2.36 (*m*, 1H), 2.39 (*dd*, *J* = 4.2, 14.6 Hz, 1H), 2.42 – 2.50 (*m*, 1H), 4.05 – 4.18 (*m*, 2H), 4.77 (*t*, *J* = 2.5 Hz, 1H), 4.79 (*t*, *J* = 2.2 Hz, 1H), 5.05 – 5.13 (*m*, 1H). ^{13}C-NMR (126 MHz, $CDCl_3$): δ 17.78, 19.52, 23.56, 25.52, 25.86, 26.71, 28.45, 29.59, 30.52, 35.47, 35.62, 37.10, 43.97, 46.73, 63.01, 103.69, 124.68, 131.47, 161.44, 173.87.

Synthesis of esters 7 from alcohol 4 – ***General procedure 3 (GP3)***

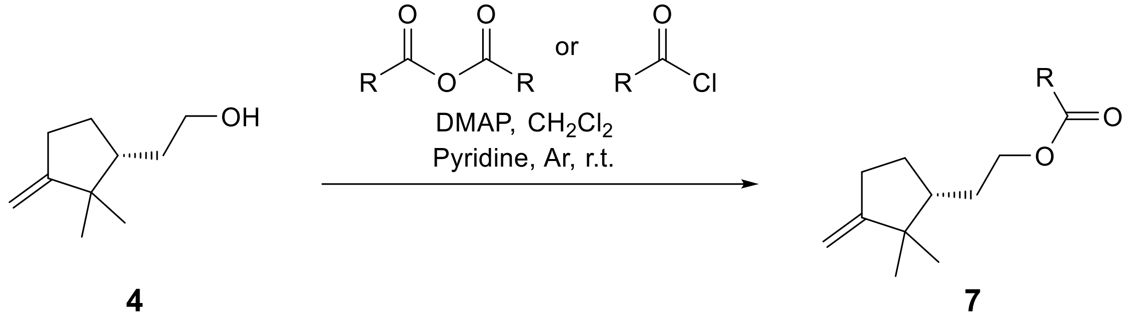

To a solution of (*R*)-2-(2,2-dimethyl-3-methylenecyclopentyl)ethan-1-ol (**4**) (1.0 equiv.) in anhydrous CH_2Cl_2 (2 mL) under argon, pyridine (3.0 equiv.), DMAP (0.04 equiv.) and the corresponding acid anhydride/acid chloride were added. The resulting reaction mixture was stirred at room temperature for 20 hours. The volatiles were evaporated *in vacuo*. The residue was purified by column chromatography (Silica gel 60). The fractions containing the pure product **7** were combined and the volatiles were evaporated *in vacuo*. The isolated esters **7** were fully characterized.

Synthesis of (*R*)-2-(2,2-dimethyl-3-methylenecyclopentyl)ethyl acetate (7a)

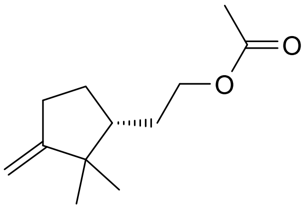

Following *GP3*. Prepared from (*R*)-2-(2,2-dimethyl-3-methylenecyclopentyl)ethan-1-ol (**4**) (1.0 mmol, 154 mg), CH_2Cl_2 (2 mL), pyridine (3.0 mmol, 243 μL), DMAP (0.04 mmol, 4.9 mg) and acetic anhydride (1.5 mmol, 142 μL); column chromatography: EtOAc/petroleum ether = 1:20. Yield: 169 mg (0.86 mmol, 86 %) of colorless oil. $[\alpha]_D^{r.t.}$ = +10.0 (0.34, CH_2Cl_2). EI-HRMS: *m/z* = 197.1497 (MH^+); $C_{12}H_{21}O_2$ requires: *m/z* = 197.1493 (MH^+); ν_{max} 2959, 2868, 1739, 1652, 1463, 1434, 1364, 1228, 1153, 1038, 968, 878, 636, 606 cm^{-1}. ^{1}H-NMR (500 MHz, $CDCl_3$): δ 0.82 (*s*, 3H), 1.06 (*s*, 3H), 1.24 – 1.37 (*m*, 1H), 1.37 – 1.48 (*m*, 1H), 1.51 – 1.59 (*m*, 1H), 1.75 – 1.82 (*m*, 1H), 1.82 – 1.90 (*m*, 1H), 2.05 (*s*, 3H), 2.24 – 2.36 (*m*, 1H), 2.42 – 2.52 (*m*, 1H), 4.05 – 4.11 (*m*, 1H), 4.12 – 4.18 (*m*, 1H), 4.74 – 4.80 (*m*, 2H). ^{13}C-NMR (126 MHz, $CDCl_3$): δ 21.19, 23.46, 26.55, 28.20, 29.03, 30.76, 44.10, 47.12, 64.23, 103.28, 162.10, 171.33.

Synthesis of (*R*)-2-(2,2-dimethyl-3-methylenecyclopentyl)ethyl propionate (7b)

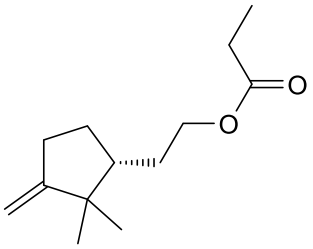

Following *GP3*. Prepared from (*R*)-2-(2,2-dimethyl-3-methylenecyclopentyl)ethan-1-ol (**4**) (1.0 mmol, 154 mg), CH_2Cl_2 (2 mL), pyridine (3.0 mmol, 243 μL), DMAP (0.04 mmol, 4.9 mg) and propionic anhydride (1.5 mmol, 194 μL); column chromatography: EtOAc/petroleum ether = 1:20. Yield: 203 mg (0.96 mmol, 96 %) of colorless oil. $[\alpha]_D^{r.t.}$ = +14.0 (0.35, CH_2Cl_2). EI-HRMS: *m/z* = 211.1689 (MH^+); $C_{13}H_{23}O_2$ requires: *m/z* = 211.1693 (MH^+); ν_{max} 2959, 1736, 1652, 1463, 1384, 1362, 1348, 1274, 1179, 1082, 1022, 958, 878, 807 cm^{-1}. ^{1}H-NMR (300 MHz, $CDCl_3$): δ 0.83 (*s*, 3H), 1.06 (*s*, 3H), 1.14 (*t*, *J* = 7.6 Hz, 3H), 1.19 – 1.58 (*m*, 3H), 1.70 – 1.95 (*m*, 2H), 2.20 – 2.37 (*m*, 3H), 2.39 – 2.54 (*m*, 1H), 4.01 – 4.23 (*m*, 2H), 4.73 – 4.82 (*m*, 2H). ^{13}C-NMR (75 MHz, $CDCl_3$): δ 9.30, 23.45, 26.59, 27.80, 28.27, 29.14, 30.79, 44.11, 47.24, 64.06, 103.26, 162.14, 174.65.

Synthesis of (*R*)-2-(2,2-dimethyl-3-methylenecyclopentyl)ethyl butyrate (7c)

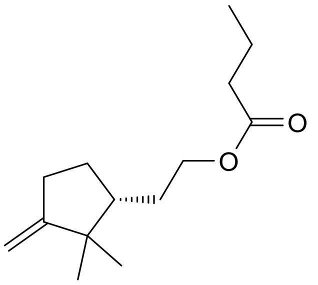

Following *GP3*. Prepared from (*R*)-2-(2,2-dimethyl-3-methylenecyclopentyl)ethan-1-ol (**4**) (1.0 mmol, 154 mg), CH_2Cl_2 (2 mL), pyridine (3.0 mmol, 243 μL), DMAP (0.04 mmol, 4.9 mg) and butyric anhydride (1.3 mmol, 215 μL); column chromatography: EtOAc/petroleum ether = 1:40. Yield: 198 mg (0.88 mmol, 88 %) of colorless oil. $[\alpha]_D^{r.t.}$ = +12.0 (0.34, CH_2Cl_2). EI-HRMS: m/z = 225.1854 (MH^+); $C_{14}H_{25}O_2$ requires: m/z = 225.1849 (MH^+); ν_{max} 2960, 2871, 1735, 1652, 1461, 1362, 1252, 1174, 1091, 1048, 992, 975, 933, 878 cm^{-1}. ^{1}H-NMR (300 MHz, $CDCl_3$): δ 0.82 (*s*, 3H), 0.95 (*t*, J = 7.4 Hz, 3H), 1.06 (*s*, 3H), 1.20 – 1.95 (*m*, 7H), 2.21 – 2.37 (*m*, 3H), 2.40 – 2.54 (*m*, 1H), 4.01 – 4.23 (*m*, 2H), 4.73 – 4.82 (*m*, 2H). ^{13}C-NMR (75 MHz, $CDCl_3$): δ 13.82, 18.62, 23.46, 26.60, 28.25, 29.15, 30.79, 36.45, 44.10, 47.23, 63.94, 103.26, 162.16, 173.87.

Synthesis of (*R*)-2-(2,2-dimethyl-3-methylenecyclopentyl)ethyl isobutyrate (7d)

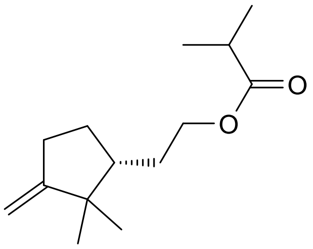

Following *GP3*. Prepared from (*R*)-2-(2,2-dimethyl-3-methylenecyclopentyl)ethan-1-ol (**4**) (1.0 mmol, 154 mg), CH_2Cl_2 (2 mL), pyridine (3.0 mmol, 243 μL), DMAP (0.04 mmol, 4.9 mg) and isobutyric anhydride (1.5 mmol, 249 μL); column chromatography: EtOAc/petroleum ether = 1:30. Yield: 181 mg (0.80 mmol, 80 %) of colorless oil. $[\alpha]_D^{r.t.}$ = +15.0 (0.30, CH_2Cl_2). EI-HRMS: *m/z* = 225.1854 (MH^+); $C_{14}H_{25}O_2$ requires: *m/z* = 225.1849 (MH^+); ν_{max} 2960, 1733, 1652, 1469, 1388, 1363, 1344,1258, 1190, 1153, 1075, 990, 971, 879, 754 cm^{-1}. ^{1}H-NMR (500 MHz, $CDCl_3$): δ 0.82 (*s*, 3H), 1.06 (*s*, 3H), 1.17 (*d*, *J* = 7.0, 6H), 1.26 – 1.36 (*m*, 1H), 1.39 – 1.47 (*m*, 1H), 1.51 – 1.60 (*m*, 1H), 1.74 – 1.82 (*m*, 1H), 1.83 – 1.92 (*m*, 1H), 2.24 – 2.36 (*m*, 1H), 2.42 – 2.50 (*m*, 1H), 2.54 (*p*, *J* = 7.0 Hz, 1H), 4.04 – 4.11 (*m*, 1H), 4.12 – 4.20 (*m*, 1H), 4.74 – 4.81 (*m*, 2H). ^{13}C-NMR (126 MHz, $CDCl_3$): δ 19.15, 23.46, 26.55, 28.26, 29.10, 30.79, 34.19, 44.08, 47.20, 63.96, 103.26, 162.18, 177.35.

Synthesis of (*R*)-2-(2,2-dimethyl-3-methylenecyclopentyl)ethyl 3,3-dimethylbutanoate (7e)

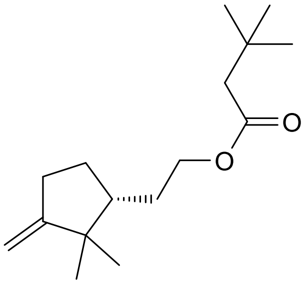

Following *GP3*. Prepared from (*R*)-2-(2,2-dimethyl-3-methylenecyclopentyl)ethan-1-ol (**4**) (1.0 mmol, 154 mg), CH_2Cl_2 (2 mL), pyridine (3.0 mmol, 243 μL), DMAP (0.04 mmol, 4.9 mg) and 3,3-dimethylbutyryl chloride (1.1 mmol, ω = 0.98, 153 μL); column chromatography: EtOAc/petroleum ether = 1:40. Yield: 187 mg (0.74 mmol, 74 %) of colorless oil. $[\alpha]_D^{r.t.}$ = +10.3 (0.25, CH_2Cl_2). ν_{max} 3071, 2957, 2869, 1733, 1652, 1466, 1434, 1365, 1322, 1226, 1197, 1129, 1049, 1020, 996, 978, 931, 879, 795, 729, 704, 620 cm^{-1}. ^{1}H-NMR (300 MHz, $CDCl_3$): δ 0.83 (*s*, 3H), 1.03 (*s*, 9H), 1.06 (*s*, 3H), 1.22 – 1.48 (*m*, 2H), 1.50 – 1.64 (*m*, 1H), 1.70 – 1.95 (*m*, 2H), 2.19 (*s*, 2H), 2.22 – 2.37 (*m*, 1H), 2.39 – 2.55 (*m*, 1H), 3.99 – 4.23 (*m*, 2H), 4.72 – 4.82 (*m*, 2H). ^{13}C-NMR (75 MHz, $CDCl_3$): δ 23.48, 26.64, 28.22, 29.18, 29.60, 29.81, 30.80, 44.09, 47.19, 48.24, 63.65, 103.27, 162.19, 172.57.

Synthesis of (*R*)-2-(2,2-dimethyl-3-methylenecyclopentyl)ethyl pent-4-enoate (7f)

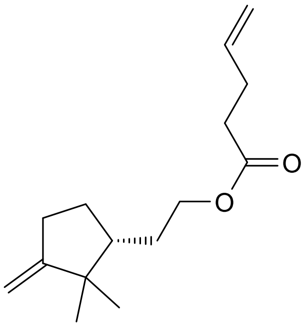

Following *GP3*. Prepared from (*R*)-2-(2,2-dimethyl-3-methylenecyclopentyl)ethan-1-ol (**4**) (1.0 mmol, 154 mg), CH_2Cl_2 (2 mL), pyridine (3.0 mmol, 243 μL), DMAP (0.04 mmol, 4.9 mg) and 4-pentenoic anhydride (1.5 mmol, ω = 0.98, 280 μL); column chromatography: EtOAc/petroleum ether = 1:40. Yield: 194 mg (0.82 mmol, 82 %) of colorless oil. $[\alpha]_D^{r.t.}$ = +13.9 (0.28, CH_2Cl_2). EI-HRMS: *m/z* = 237.1849 (MH^+); $C_{15}H_{25}O_2$ requires: *m/z* = 237.1849 (MH^+); ν_{max} 3073, 2959, 2868, 1735, 1651, 1463, 1435, 1362, 1235, 1167, 1102, 1050, 993, 914, 879 cm^{-1}. ^1H-NMR (300 MHz, $CDCl_3$): δ 0.82 (*s*, 3H), 1.06 (*s*, 3H), 1.20 – 1.65 (*m*, 3H), 1.70 – 1.94 (*m*, 2H), 2.20 – 2.54 (*m*, 6H), 4.02 – 4.24 (*m*, 2H), 4.72 – 4.82 (*m*, 2H), 4.95 – 5.13 (*m*, 2H), 5.73 – 5.92 (*m*, 1H). ^{13}C-NMR (75 MHz, $CDCl_3$): δ 23.46, 26.60, 28.24, 29.04, 29.12, 30.78, 33.77, 44.10, 47.19, 64.12, 103.28, 115.59, 136.87, 162.11, 173.21.

Synthesis of (*R*)-2-(2,2-dimethyl-3-methylenecyclopentyl)ethyl benzoate (7g)

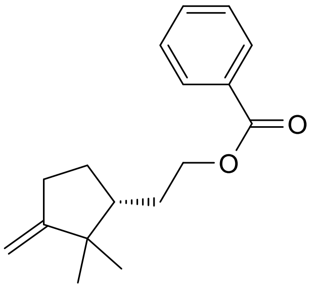

Following *GP3*. Prepared from (*R*)-2-(2,2-dimethyl-3-methylenecyclopentyl)ethan-1-ol (**4**) (1.0 mmol, 154 mg), CH_2Cl_2 (2 mL), pyridine (3.0 mmol, 243 µL), DMAP (0.04 mmol, 4.9 mg) and benzoic anhydride (1.3 mmol, 297 mg); column chromatography: EtOAc/petroleum ether = 1:30. Yield: 226 mg (0.87 mmol, 87 %) of colorless oil. $[\alpha]_D^{r.t.}$ = +8.0 (0.37, CH_2Cl_2). EI-HRMS: *m/z* = 259.1691 (MH^+); $C_{17}H_{23}O_2$ requires: *m/z* = 259.1693 (MH^+); ν_{max} 2958, 1717, 1651, 1602, 1452, 1385, 1362, 1314, 1268, 1175, 1110, 1069, 1026, 958, 878, 708, 687, 675 cm^{-1}. ^1H-NMR (500 MHz, $CDCl_3$): δ 0.86 (*s*, 3H), 1.10 (*s*, 3H), 1.31 – 1.44 (*m*, 1H), 1.52 – 1.61 (*m*, 1H), 1.62 – 1.70 (*m*, 1H), 1.88 – 2.00 (*m*, 2H), 2.26 – 2.38 (*m*, 1H), 2.44 – 2.54 (*m*, 1H), 4.30 – 4.44 (*m*, 2H), 4.76 – 4.82 (*m*, 2H), 7.40 – 7.48 (*m*, 2H), 7.52 – 7.59 (*m*, 1H), 8.01 – 8.08 (*m*, 2H). ^{13}C-NMR (126 MHz, $CDCl_3$): δ 23.48, 26.56, 28.32, 29.17, 30.79, 44.16, 47.36, 64.74, 103.31, 128.48, 129.66, 130.56, 132.97, 162.09, 166.77.

Synthesis of (*R*)-4-(2-(2,2-dimethyl-3-methylenecyclopentyl)ethoxy)-4-oxobutanoic acid (7h)

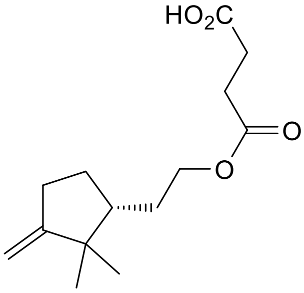

Following *GP3*. Prepared from (*R*)-2-(2,2-dimethyl-3-methylenecyclopentyl)ethan-1-ol (**4**) (1.0 mmol, 154 mg), CH_2Cl_2 (2 mL), pyridine (3.0 mmol, 243 µL), DMAP (0.04 mmol, 4.9 mg) and benzoic anhydride (1.0 mmol, 100 mg). The residue (after evaporation of the volatiles) was dissolved in EtOAc (50 mL) and washed with $NaHSO_4$ (aq., 1 M, 2×5 mL) and NaCl (aq. sat., 5 mL). The organic phase was dried under anhydrous Na_2SO_4, filtered and the volatile components evaporated *in vacuo*. The residue was purified by column chromatography (EtOAc). Yield: 191 mg (0.75 mmol, 75 %) of colorless oil. $[\alpha]_D^{r.t.}$ = +4.8 (0.255, CH_2Cl_2). EI-HRMS: *m/z* = 255.1589 (MH^+); $C_{14}H_{23}O_4$ requires: *m/z* = 255.1591 (MH^+); ν_{max} 2958, 1734, 1710, 1651, 1397, 1362, 1163, 1047, 993, 930, 878, 840 cm^{-1}. ^1H-NMR (300 MHz, $CDCl_3$): δ 0.82 (*s*, 3H), 1.06 (*s*, 3H), 1.20 – 1.64 (*m*, 3H), 1.71 – 1.93 (*m*, 2H), 2.21 – 2.37 (*m*, 1H), 2.39 – 2.55 (*m*, 1H), 2.56 – 2.74 (*m*, 4H), 4.04 – 4.26 (*m*, 2H), 4.72 – 4.82 (*m*, 2H), 10.33 (br *s*, 1H). ^{13}C-NMR (75 MHz, $CDCl_3$): δ 23.44, 26.57, 28.20, 29.01, 29.05, 29.09, 30.75, 44.09, 47.14, 64.64, 103.29, 162.06, 172.28, 178.34.

Synthesis of (*R*)-2-(2,2-dimethyl-3-methylenecyclopentyl)ethyl methyl succinate (7i)

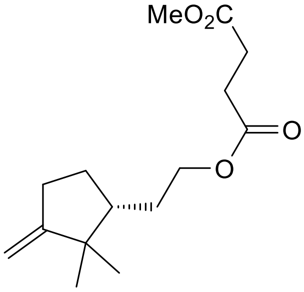

Following *GP2*. Prepared from (*R*)-4-(2-(2,2-dimethyl-3-methylenecyclopentyl)ethoxy)-4-oxobutanoic acid (**7h**) (1.0 mmol, 254 mg), methanol (3.0 mmol, 122 μL), DMAP (0.1 mmol, 12.2 mg), CH_2Cl_2 (7 mL) in EDCI (2.0 mmol, ω = 98 %, 391 mg); column chromatography: EtOAc/petroleum ether = 1:10. Yield: 215 mg (0.80 mmol, 80 %) of colorless oil. $[\alpha]_D^{r.t.}$ = +10.6 (0.23, CH_2Cl_2). EI-HRMS: *m/z* = 269.1751 (MH^+); $C_{15}H_{25}O_4$ requires: *m/z* = 269.1747 (MH^+); ν_{max} 2957, 1733, 1651, 1436, 1362, 1317, 1155, 996, 879, 846 cm^{-1}. ^{1}H-NMR (500 MHz, $CDCl_3$): δ 0.82 (*s*, 3H), 1.06 (*s*, 3H), 1.24 – 1.36 (*m*, 1H), 1.37 – 1.48 (*m*, 1H), 1.49 – 1.59 (*m*, 1H), 1.73 – 1.90 (*m*, 2H), 2.24 – 2.36 (*m*, 1H), 2.41 – 2.52 (*m*, 1H), 2.64 (*s*, 4H), 3.70 (*s*, 3H), 4.06 – 4.22 (*m*, 2H), 4.74 – 4.80 (*m*, 2H). ^{13}C-NMR (126 MHz, $CDCl_3$): δ 23.48, 26.56, 28.19, 29.00, 29.06, 29.31, 30.77, 44.10, 47.11, 52.01, 64.54, 103.31, 162.09, 172.47, 172.94.

Synthesis of (*R*)-2-(2,2-dimethyl-3-methylenecyclopentyl)ethyl ethyl succinate (7j)

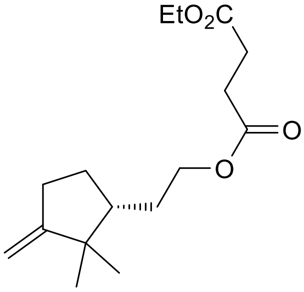

Following *GP2*. Prepared from (*R*)-4-(2-(2,2-dimethyl-3-methylenecyclopentyl)ethoxy)-4-oxobutanoic acid (**7h**) (1.0 mmol, 254 mg), ethanol (3.0 mmol, 175 µL), DMAP (0.1 mmol, 12.2 mg), CH_2Cl_2 (7 mL) in EDCI (2.0 mmol, ω = 98 %, 391 mg); column chromatography: EtOAc/petroleum ether = 1:10. Yield: 220 mg (0.78 mmol, 78 %) of colorless oil. $[\alpha]_D^{r.t.}$ = +11.6 (0.23, CH_2Cl_2). EI-HRMS: *m/z* = 283.1904 (MH^+); $C_{16}H_{27}O_4$ requires: *m/z* = 283.1904 (MH^+); ν_{max} 2959, 1732, 1651, 1464, 1363, 1349, 1314, 1154, 1125, 967. 879 cm^{-1}. ^{1}H-NMR (300 MHz, $CDCl_3$): δ 0.82 (*s*, 3H), 1.06 (*s*, 3H), 1.26 (*t*, *J* = 7.1 Hz, 3H), 1.22 – 1.32 (*m*, 1H), 1.36 – 1.61 (*m*, 2H), 1.70 – 1.94 (*m*, 2H), 2.21 – 2.37 (*m*, 1H), 2.38 – 2.57 (*m*, 1H), 2.62 (*s*, 4H), 4.03 – 4.26 (*m*, 4H), 4.73 – 4.82 (*m*, 2H). ^{13}C-NMR (75 MHz, $CDCl_3$): δ 14.32, 23.46, 26.59, 28.23, 29.06, 29.35, 29.38, 30.78, 44.11, 47.18, 60.82, 64.50, 103.29, 162.08, 172.43, 172.49.

Synthesis of ethers 8 from alcohol 4 – *General procedure 4 (GP4)*

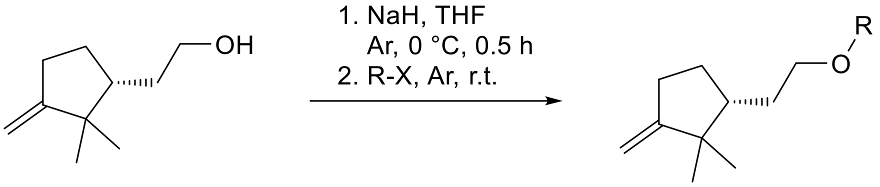

4 **8**

To a solution of (*R*)-2-(2,2-dimethyl-3-methylenecyclopentyl)ethan-1-ol (**4**) (1.0 equiv.) in anhydrous THF (3 mL) under argon at 0 °C was added NaH (1.2 equiv.). The resulting reaction mixture was stirred at 0 °C for 30 minutes, then the electrophile (aliphatic halide) was added. After stirring at room temperature for 20 hours, H_2O (10 mL) was carefully added. The resulting mixture was extracted with Et_2O (30 mL). The organic phase was washed with NaCl (aq. sat., 3×10 mL), dried under anhydrous Na_2SO_4, filtered and the volatiles evaporated *in vacuo*. The residue was purified by column chromatography (Silica gel 60). The fractions containing the pure product **8** were combined and the volatiles were evaporated *in vacuo*. The isolated ethers **8** were fully characterized with the exception of HRMS, as the products were not ionized.

Synthesis of (*R*)-2-(2-methoxyethyl)-1,1-dimethyl-5-methylenecyclopentane (8a)

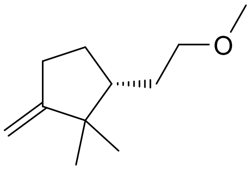

Following *GP4*. Prepared from (*R*)-2-(2,2-dimethyl-3-methylenecyclopentyl)ethan-1-ol (**4**) (1.0 mmol, 154 mg), THF (3 mL), NaH (1.2 mmol; ω = 60 %, 48.0 mg) and iodomethane (2.0 mmol, 129 µL); column chromatography: EtOAc/petroleum ether = 1:20. Yield: 156 mg (0.93 mmol, 93 %) of colorless oil. $[\alpha]_D^{r.t.}$ = +13.0 (0.20, CH_2Cl_2). ν_{max} 2958, 2866, 1651, 1462, 1386, 1362, 1199, 1152, 1120, 1003, 957, 877, 836, 704 cm^{-1}. ^{1}H-NMR (500 MHz, $CDCl_3$): δ 0.82 (*s*, 3H), 1.06 (*s*, 3H), 1.24 – 1.32 (*m*, 1H), 1.33 – 1.40 (*m*, 1H), 1.51 – 1.61 (*m*, 1H), 1.71 – 1.78 (*m*, 1H), 1.80 – 1.88 (*m*, 1H), 2.23 – 2.35 (*m*, 1H), 2.40 – 2.50 (*m*, 1H), 3.34 (*s*, 3H), 3.37 – 3.48 (*m*, 2H), 4.73 – 4.80 (*m*, 2H). ^{13}C-NMR (126 MHz, $CDCl_3$): δ 23.53, 26.62, 28.34, 29.95, 30.86, 44.06, 47.12, 58.70, 72.40, 103.06, 162.55.

Synthesis of (*R*)-2-(2-ethoxyethyl)-1,1-dimethyl-5-methylenecyclopentane (8b)

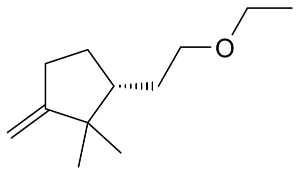

Following *GP4*. Prepared from (*R*)-2-(2,2-dimethyl-3-methylenecyclopentyl)ethan-1-ol (**4**) (1.0 mmol, 154 mg), THF (3 mL), NaH (1.2 mmol; ω = 60 %, 48.0 mg) and bromoethane (2.0 mmol, 153 µL); column chromatography: EtOAc/petroleum ether = 1:20. Yield: 125 mg (0.68 mmol, 68 %) of colorless oil. $[\alpha]_D^{r.t.}$ = +17.0 (0.22, CH_2Cl_2). ν_{max} 2959, 2863, 1651, 1463, 1377, 1362, 1111, 877, 704 cm^{-1}. ^{1}H-NMR (500 MHz, $CDCl_3$): δ 0.82 (*s*, 3H), 1.06 (*s*, 3H), 1.21 (*t*, *J* = 7.0 Hz, 3H), 1.25 – 1.35 (*m*, 1H), 1.36 – 1.43 (*m*, 1H), 1.50 – 1.58 (*m*, 1H), 1.73 – 1.79 (*m*, 1H), 1.79 – 1.87 (*m*, 1H), 2.23 – 2.37 (*m*, 1H), 2.40 – 2.50 (*m*, 1H), 3.37 – 3.54 (*m*, 4H), 4.73 – 4.79 (*m*, 2H). ^{13}C-NMR (126 MHz, $CDCl_3$): δ 15.41, 23.54, 26.60, 28.39, 30.15, 30.88, 44.12, 47.26, 66.24, 70.36, 103.02, 162.59.

Synthesis of (*R*)-1,1-dimethyl-2-methylene-5-(2-propoxyethyl)cyclopentane (8c)

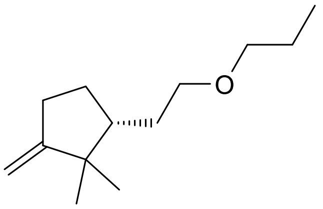

Following *GP4*. Prepared from (*R*)-2-(2,2-dimethyl-3-methylenecyclopentyl)ethan-1-ol (**4**) (1.0 mmol, 154 mg), THF (3 mL), NaH (1.2 mmol; ω = 60 %, 48.0 mg) and 1-bromopropane (1.2 mmol, 109 µL); column chromatography: EtOAc/petroleum ether = 1:25. Yield: 154 mg (0.78 mmol, 78 %) of colorless oil. $[\alpha]_D^{r.t.}$ = +13.0 (0.22, CH_2Cl_2). ν_{max} 3071, 2959, 2935, 2860, 1651, 1462, 1435, 1362, 1249, 1115, 981, 956, 877, 703 cm^{-1}. ^{1}H-NMR (500 MHz, $CDCl_3$): δ 0.82 (*s*, 3H), 0.92 (*t*, *J* = 7.5 Hz, 3H), 1.06 (*s*, 3H), 1.21 – 1.42 (*m*, 2H), 1.51 – 1.64 (*m*, 3H), 1.72 – 1.79 (*m*, 1H), 1.80 – 1.89 (*m*, 1H), 2.23 – 2.34 (*m*, 1H), 2.40 – 2.50 (*m*, 1H), 3.33 – 3.45 (*m*, 3H), 3.46 – 3.53 (*m*, 1H), 4.73 – 4.81 (*m*, 2H). ^{13}C-NMR (126 MHz, $CDCl_3$): δ 10.78, 23.11, 23.54, 26.60, 28.40, 30.10, 30.88, 44.12, 47.28, 70.50, 72.76, 103.00, 162.63.

Synthesis of (*R*)-2-(2-butoxyethyl)-1,1-dimethyl-5-methylenecyclopentane (8d)

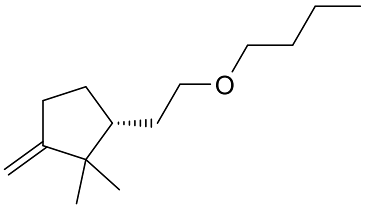

Following *GP4*. Prepared from (*R*)-2-(2,2-dimethyl-3-methylenecyclopentyl)ethan-1-ol (**4**) (1.0 mmol, 154 mg), THF (3 mL), NaH (1.2 mmol; ω = 60 %, 48.0 mg) and 1-iodobutane (1.0 mmol, 114 µL); column chromatography: EtOAc/petroleum ether = 1:30. Yield: 177 mg (0.84 mmol, 84 %) of colorless oil. $[\alpha]_D^{r.t.}$ = +14.0 (0.20, CH_2Cl_2). ν_{max} 3071, 2957, 2928, 2857, 2796, 1652, 1463, 1434, 1375, 1362, 1302, 1234, 1114, 998, 962, 940, 878, 738, 704 cm^{-1}. ^{1}H-NMR (500 MHz, $CDCl_3$): δ 0.82 (*s*, 3H), 0.92 (*t*, *J* = 7.4 Hz, 3H), 1.06 (*s*, 3H), 1.19 – 1.48 (*m*, 4H), 1.48 – 1.62 (*m*, 3H), 1.70 – 1.88 (*m*, 2H), 2.23 – 2.37 (*m*, 1H), 2.40 – 2.50 (*m*, 1H), 3.36 – 3.52 (*m*, 4H), 4.73 – 4.79 (*m*, 2H). ^{13}C-NMR (126 MHz, $CDCl_3$): δ 14.10, 19.55, 23.55, 26.61, 28.40, 30.10, 30.89, 32.04, 44.12, 47.28, 70.55, 70.86, 103.00, 162.65.

Synthesis of (*R*)-2-(2-(allyloxy)ethyl)-1,1-dimethyl-5-methylenecyclopentane (8e)

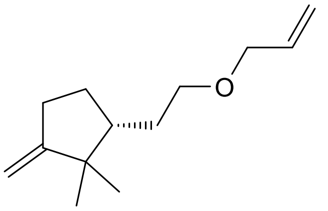

Following *GP4*. Prepared from (*R*)-2-(2,2-dimethyl-3-methylenecyclopentyl)ethan-1-ol (**4**) (1.0 mmol, 154 mg), THF (3 mL), NaH (1.2 mmol; ω = 60 %, 48.0 mg) and allyl bromide (1.1 mmol, ω = 0.97, 98 µL); column chromatography: EtOAc/petroleum ether = 1:15. Yield: 161 mg (0.83 mmol, 83 %) of colorless oil. $[\alpha]_D^{r.t.}$ = +23.0 (0.17, CH_2Cl_2). ν_{max} 3071, 2958, 2934, 2865, 1651, 1463, 1433, 1362, 1145, 1106, 993, 920, 878, 704, 634 cm^{-1}. ^{1}H-NMR (500 MHz, $CDCl_3$): δ 0.82 (*s*, 3H), 1.06 (*s*, 3H), 1.23 – 1.32 (*m*, 1H), 1.35 – 1.45 (*m*, 1H), 1.52 – 1.62 (*m*, 1H), 1.72 – 1.89 (*m*, 2H), 2.23 – 2.33 (*m*, 1H), 2.40 – 2.50 (*m*, 1H), 3.41 – 3.47 (*m*, 1H), 3.51 (*td*, *J* = 5.2, 8.8 Hz, 1H), 3.92 – 4.03 (*m*, 2H), 4.73 – 4.79 (*m*, 2H), 5.17 (*dq*, *J* = 1.4, 10.3 Hz, 1H), 5.28 (*dq*, *J* = 1.7, 17.2 Hz, 1H), 5.93 (*ddt*, *J* = 5.6, 10.4, 17.2, 1H). ^{13}C-NMR (126 MHz, $CDCl_3$): δ 23.53, 26.61, 28.38, 30.09, 30.86, 44.11, 47.22, 70.03, 71.98, 103.04, 116.91, 135.14, 162.56.

Synthesis of (*R*)-1,1-dimethyl-2-(2-((3-methylbut-2-en-1-yl)oxy)ethyl)-5-methylenecyclopentane (8f)

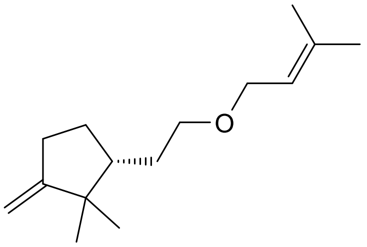

Following *GP4*. Prepared from (*R*)-2-(2,2-dimethyl-3-methylenecyclopentyl)ethan-1-ol (**4**) (1.0 mmol, 154 mg), THF (3 mL), NaH (1.2 mmol; ω = 60 %, 48.0 mg) and prenyl bromide (1.0 mmol, ω = 0.95, 121 µL); column chromatography: EtOAc/petroleum ether = 1:20. Yield: 171 mg (0.77 mmol, 77 %) of colorless oil. $[\alpha]_D^{r.t.}$ = +2.5 (0.24, CH_2Cl_2). EI-HRMS: *m/z* = 223.2056 (MH^+); $C_{15}H_{27}O$ requires: *m/z* = 223.2056 (MH^+); ν_{max} 2929, 2862, 1445, 1377, 1360, 1081, 1014, 799 cm^{-1}. ^{1}H-NMR (500 MHz, $CDCl_3$): δ 0.81 (*s*, 3H), 1.06 (*s*, 3H), 1.24 – 1.34 (*m*, 1H), 1.34 – 1.43 (*m*, 1H), 1.49 – 1.61 (*m*, 1H), 1.68 (*s*, 3H), 1.75 (*s*, 3H), 1.76 – 1.87 (*m*, 2H), 2.23 – 2.34 (*m*, 1H), 2.40 – 2.50 (*m*, 1H), 3.42 (*ddd*, *J* = 6.9, 8.1, 9.1 Hz, 1H), 3.49 (*td*, *J* = 5.1, 8.9 Hz, 1H), 3.90 – 4.01 (*m*, 2H), 4.73 – 4.79 (*m*, 2H), 5.32 – 5.40 (*m*, 1H). ^{13}C-NMR (75 MHz, $CDCl_3$): δ 18.14, 23.54, 25.92, 26.67, 28.39, 30.18, 30.90, 44.12, 47.27, 67.40, 69.88, 103.00, 121.52, 136.71, 162.62.

Synthesis of (*R*)-1,1-dimethyl-2-methylene-5-(2-(prop-2-yn-1-yloxy)ethyl)cyclopentane (8g)

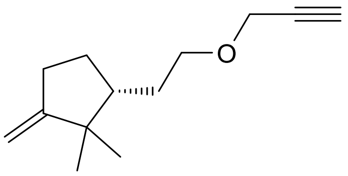

Following *GP4*. Prepared from (*R*)-2-(2,2-dimethyl-3-methylenecyclopentyl)ethan-1-ol (**4**) (1.0 mmol, 154 mg), THF (3 mL), NaH (1.2 mmol; ω = 60 %, 48.0 mg) and propargyl bromide (1.1 mmol, ω = 0.80, 118 μL); column chromatography: EtOAc/petroleum ether = 1:20. Yield: 125 mg (0.65 mmol, 65 %) of colorless oil. $[\alpha]_D^{r.t.}$ = +17.9 (0.19, CH_2Cl_2). ν_{max} 3306, 2958, 2866, 1651, 1463, 1361, 1093, 1012, 878, 626 cm^{-1}. ^{1}H-NMR (500 MHz, $CDCl_3$): δ 0.82 (*s*, 3H), 1.06 (*s*, 3H), 1.26 – 1.34 (*m*, 1H), 1.37 – 1.45 (*m*, 1H), 1.52 – 1.63 (*m*, 1H), 1.74 – 1.81 (*m*, 1H), 1.82 – 1.89 (*m*, 1H), 2.23 – 2.36 (*m*, 1H), 2.43 (*t*, *J* = 2.4 Hz, 1H), 2.43 – 2.51 (*m*, 1H), 3.53 (*dt*, *J* = 7.4, 8.9 Hz, 1H), 3.60 (*ddd*, *J* = 5.1, 8.1, 9.0 Hz, 1H), 4.10 – 4.21 (*m*, 2H), 4.73 – 4.81 (*m*, 2H). ^{13}C-NMR (126 MHz, $CDCl_3$): δ 23.53, 26.62, 28.29, 29.86, 30.84, 44.07, 47.08, 58.17, 69.75, 74.28, 80.11, 103.10, 162.47.

Synthesis of (*R*)-((2-(2,2-dimethyl-3-methylenecyclopentyl)ethoxy)methyl)benzene (8h)

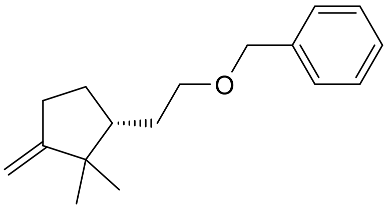

Following *GP4*. Prepared from (*R*)-2-(2,2-dimethyl-3-methylenecyclopentyl)ethan-1-ol (**4**) (1.0 mmol, 154 mg), THF (3 mL), NaH (1.2 mmol; ω = 60 %, 48.0 mg) and benzyl bromide (1.1 mmol, ω = 0.98, 134 µL); column chromatography: EtOAc/petroleum ether = 1:20. Yield: 209 mg (0.85 mmol, 85 %) of colorless oil. $[\alpha]_D^{r.t.}$ = +14.0 (0.23, CH_2Cl_2). EI-HRMS: *m/z* = 245.1898 (MH^+); $C_{17}H_{25}O$ requires: *m/z* = 245.1900 (MH^+); ν_{max} 3067, 2958, 2864, 1651, 1496, 1454, 1361, 1202, 1100, 1028, 1000, 877, 733, 696, 611 cm^{-1}. ^1H-NMR (500 MHz, $CDCl_3$): δ 0.82 (*s*, 3H), 1.06 (*s*, 3H), 1.20 – 1.34 (*m*, 1H), 1.36 – 1.47 (*m*, 1H), 1.59 – 1.60 (*m*, 1H), 1.75 – 1.86 (*m*, 2H), 2.27 – 2.28 (*m*, 1H), 2.39 – 2.50 (*m*, 1H), 3.45 – 3.51 (*m*, 1H), 3.52 – 3.60 (*m*, 1H), 4.49 (*d*, *J* = 11.8 Hz, 1H), 4.53 (*d*, *J* = 11.9 Hz, 1H), 4.73 – 4.79 (*m*, 2H), 7.25 – 7.31 (*m*, 1H), 7.32 – 7.39 (*m*, 4H). ^{13}C-NMR (126 MHz, $CDCl_3$): δ 23.55, 26.63, 28.36, 30.10, 30.87, 44.11, 47.22, 70.06, 73.07, 103.04, 127.65, 127.78, 128.50, 138.72, 162.59.

Synthesis of (*R*)-2-(2,2-dimethyl-3-methylenecyclopentyl)-*N*-methoxy-*N*-methylacetamide (9)

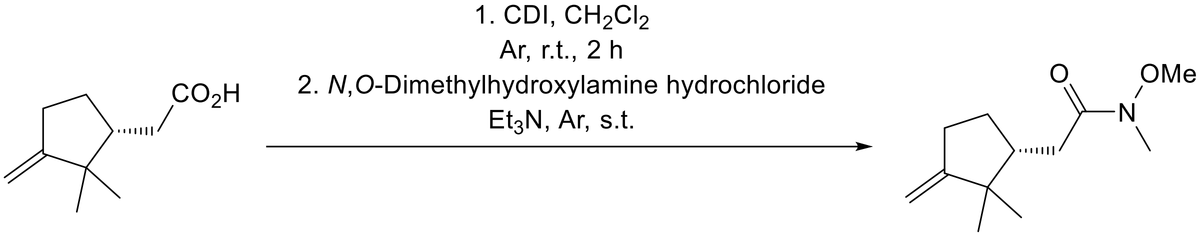

3 **9**

To a solution of (+)-isocampholenic acid (**3**) (10 mmol, 1.68 g) in anhydrous CH_2Cl_2 (50 mL) under argon at room temperature was added CDI (12 mmol, ω = 0.95, 2.05 g). The resulting reaction mixture was stirred at room temperature for 2 hours, then *N*,*O*-dimethylhydroxylamine hydrochloride (30 mmol, 2.93 g) and Et_3N (30 mmol, 4.18 mL) were added. After stirring the reaction mixture at room temperature for another 20 hours, the volatile components were evaporated *in vacuo*. The residue was dissolved in EtOAc (150 mL) and washed with $NaHSO_4$ (aq., 1 M, 2×50 mL) and NaCl (aq. sat., 2×50 mL). The organic phase was dried under anhydrous Na_2SO_4, filtered and the volatile components evaporated *in vacuo*. Yield: 1.80 g (8.5 mmol, 85 %) of white solid. $[\alpha]_D^{r.t.}$ = +21.0 (0.30, CH_2Cl_2). EI-HRMS: *m/z* = 212.1606 (MH^+); $C_{12}H_{22}NO_2$ requires: *m/z* = 212.1612 (MH^+); ν_{max} 2959, 1662, 1462, 1436, 1413, 1384, 1362, 1178, 1114, 1002, 935, 878, 785, 969 cm^{-1}. ^{1}H-NMR (500 MHz, $CDCl_3$): δ 0.87 (*s*, 3H), 1.08 (*s*, 3H), 1.29 – 1.40 (*m*, 1H), 1.90 – 1.98 (*m*, 1H), 2.02 – 2.10 (*m*, 1H), 2.21 – 2.38 (*m*, 2H), 2.41 – 2.52 (*m*, 2H), 3.19 (*s*, 3H), 3.69 (*s*, 3H), 4.78 (*t*, *J* = 2.5 Hz, 1H), 4.79 (*t*, *J* = 2.0 Hz, 1H). ^{13}C-NMR (126 MHz, $CDCl_3$): δ 23.71, 26.68, 28.58, 30.62, 32.32, 32.59, 44.01, 46.17, 61.32, 103.47, 161.75, 174.60.

Synthesis of ketones 11 from Weinreb amide 10 – ***General procedure 5 (GP5)***

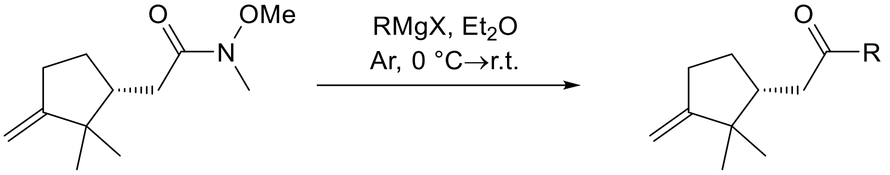

9 **10**

To a solution of (*R*)-2-(2,2-dimethyl-3-methylenecyclopentyl)-*N*-methoxy-*N*-methylacetamide (**9**) (1.0 equiv.) in anhydrous Et_2O (7 mL) under argon at 0 °C, the corresponding Grignard reagent (1.5 equiv.) was added slowly. The resulting reaction mixture was stirred at 0 °C for 1 hour and then at room temperature for 20 hours. The excess Grignard reagent was quenched with NaCl (aq. sat., 3 mL) and the resulting mixture was extracted with Et_2O (3×10 mL). The combined organic phase was washed with NaCl (aq. sat., 3×10 mL), dried under anhydrous Na_2SO_4, filtered and the volatiles evaporated *in vacuo*. The residue was purified by column chromatography (Silica gel 60). The fractions containing the pure product **10** were combined and the volatiles evaporated *in vacuo*. The isolated ketones **10** were fully characterized.

Synthesis of (*R*)-1-(2,2-dimethyl-3-methylenecyclopentyl)propan-2-one (10a)

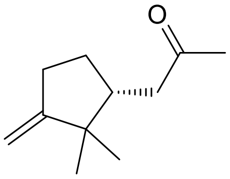

Following *GP5*. Prepared from (*R*)-2-(2,2-dimethyl-3-methylenecyclopentyl)-*N*-methoxy-*N*-methylacetamide (**9**) (2.0 mmol, 422 mg), Et_2O (7 mL), and methylmagnesium bromide (2.0 M in Et_2O, 3.0 mmol, 1.5 mL); column chromatography: EtOAc/petroleum ether = 1:10. Yield: 278 mg (1.67 mmol, 83 %) of colorless oil. $[\alpha]_D^{r.t.}$ = +15.0 (0.22, CH_2Cl_2). EI-HRMS: *m/z* = 167.1430 (MH^+); $C_{11}H_{19}O$ requires: *m/z* = 167.1430 (MH^+); ν_{max} 3071, 2960, 1712, 1651, 1463, 1434, 1362, 1285, 1241, 1170, 1148, 962, 878, 704 cm^{-1}. ^{1}H-NMR (500 MHz, $CDCl_3$): δ 0.83 (*s*, 3H), 1.06 (*s*, 3H), 1.21 – 1.30 (*m*, 1H), 1.86 – 1.94 (*m*, 1H), 1.97 – 2.04 (*m*, 1H), 2.17 (*s*, 3H), 2.25 (*dd*, *J* = 10.5, 15.9 Hz, 1H), 2.29 – 2.38 (*m*, 1H), 2.40 – 2.48 (*m*, 1H), 2.50 (*dd*, *J* = 3.6, 15.9 Hz, 1H), 4.77 (*t*, *J* = 2.5 Hz, 1H), 4.79 (*td*, *J* = 0.8, 2.2 Hz, 1H). ^{13}C-NMR (126 MHz, $CDCl_3$): δ 23.71, 26.63, 28.43, 30.50, 30.59, 43.91, 44.64, 45.59, 103.62, 161.31, 209.25.

Synthesis of (*R*)-1-(2,2-dimethyl-3-methylenecyclopentyl)butan-2-one (10b)[9]

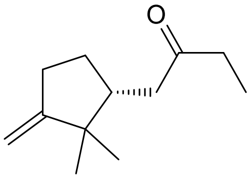

Following *GP5*. Prepared from (*R*)-2-(2,2-dimethyl-3-methylenecyclopentyl)-*N*-methoxy-*N*-methylacetamide (**9**) (2.0 mmol, 422 mg), Et_2O (7 mL), and ethylmagnesium bromide (3.0 M in Et_2O, 3.0 mmol, 1.0 mL); column chromatography: EtOAc/petroleum ether = 1:10. Yield: 314 mg (1.74 mmol, 87 %) of colorless oil. $[\alpha]_D^{r.t.}$ = +14.0 (0.23, CH_2Cl_2). EI-HRMS: *m/z* = 181.1588 (MH^+); $C_{12}H_{21}O$ requires: *m/z* = 181.1587 (MH^+); ν_{max} 3071, 2960, 1712, 1651, 1460, 1413, 1376, 1363, 1283, 1151, 1114, 1021, 986, 878, 806, 705, 635 cm^{-1}. ^{1}H-NMR (500 MHz, $CDCl_3$): δ 0.83 (*s*, 3H), 1.02 – 1.10 (*m*, 6H), 1.19 – 1.30 (*m*, 1H), 1.83 – 1.91 (*m*, 1H), 1-97 – 2.05 (*m*, 1H), 2.24 (*dd*, *J* = 10.6, 15.7 Hz, 1H), 2.29 – 2.38 (*m*, 1H), 2.40 – 2.54 (*m*, 4H), 4.77 (*t*, *J* = 2.5 Hz, 1H), 4.79 (*t*, *J* = 2.3 Hz, 1H). ^{13}C-NMR (126 MHz, $CDCl_3$): δ 8.00, 23.73, 26.63, 28.50, 30.61, 36.52, 43.25, 43.92, 45.68, 103.57, 161.43, 211.79.

Synthesis of (*R*)-1-(2,2-dimethyl-3-methylenecyclopentyl)pentan-2-one (10c)

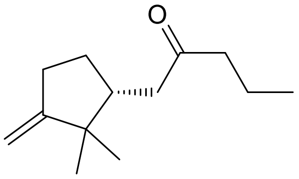

Following *GP5*. Prepared from (*R*)-2-(2,2-dimethyl-3-methylenecyclopentyl)-*N*-methoxy-*N*-methylacetamide (**9**) (2.0 mmol, 422 mg), Et_2O (7 mL), and propylmagnesium bromide (2.0 M in Et_2O, 3.0 mmol, 1.5 mL); column chromatography: EtOAc/petroleum ether = 1:20. Yield: 315 mg (1.62 mmol, 81 %) of colorless oil. $[\alpha]_D^{r.t.}$ = +9.0 (0.26, CH_2Cl_2). EI-HRMS: *m/z* = 195.1743 (MH^+); $C_{13}H_{23}O$ requires: *m/z* = 195.1743 (MH^+); ν_{max} 3071, 2960, 2873, 1711, 1651, 1462, 1409, 1363, 1285, 1198, 1125, 1026, 878, 740, 63 cm^{-1}. ^{1}H-NMR (500 MHz, $CDCl_3$): δ 0.82 (*s*, 3H), 0.92 (*t*, *J* = 7.4 Hz, 3H), 1.06 (*s*, 3H), 1.19 – 1.30 (*m*, 1H), 1.61 (*h*, *J* = 7.3 Hz, 2H), 1.84 – 1.92 (*m*, 1H), 1.96 – 2.05 (*m*, 1H), 2.23 (*dd*, *J* = 10.6, 15.7 Hz, 1H), 2.28 – 2.49 (*m*, 5H), 4.77 (*t*, *J* = 2.5 Hz, 1H), 4.79 (*t*, *J* = 2.2 Hz, 1H). ^{13}C-NMR (126 MHz, $CDCl_3$): δ 13.89, 17.44, 23.75, 26.64, 28.49, 30.62, 43.64, 43.92, 45.36, 45.60, 103.56, 161.44, 211.35.

Synthesis of (*R*)-1-(2,2-dimethyl-3-methylenecyclopentyl)hexan-2-one (10d)

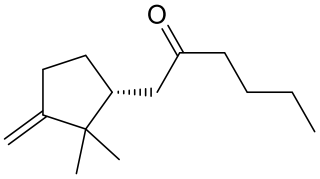

Following *GP5*. Prepared from (*R*)-2-(2,2-dimethyl-3-methylenecyclopentyl)-*N*-methoxy-*N*-methylacetamide (**9**) (2.0 mmol, 422 mg), Et_2O (7 mL), and butylmagnesium chloride (2.0 M in Et_2O, 3.0 mmol, 1.5 mL); column chromatography: EtOAc/petroleum ether = 1:20. Yield: 369 mg (1.77 mmol, 88 %) of colorless oil. $[\alpha]_D^{r.t.}$ = +9.0 (0.21, CH_2Cl_2). EI-HRMS: *m/z* = 209.1897 (MH^+); $C_{14}H_{25}O$ requires: *m/z* = 209.1900 (MH^+); ν_{max} 2958, 2871, 1712, 1651, 1463, 1435, 1409, 1377, 1363, 1285, 1198, 1126, 1035, 878, 732, 634 cm^{-1}. ^1H-NMR (500 MHz, $CDCl_3$): δ 0.82 (*s*, 3H), 0.91 (*t*, *J* = 7.4 Hz, 3H), 1.05 (*s*, 3H), 1.18 – 1.36 (*m*, 3H), 1.52 – 1.60 (*m*, 2H), 1.84 – 1.91 (*m*, 1H), 1.97 – 2.05 (*m*, 1H), 2.23 (*dd*, *J* = 10.6, 15.7 Hz, 1H), 2.28 – 2.49 (*m*, 5H), 4.77 (*t*, *J* = 2.6 Hz, 1H), 4.79 (*t*, *J* = 2.2 Hz, 1H). ^{13}C-NMR (126 MHz, $CDCl_3$): δ 14.02, 22.50, 23.75, 26.11, 26.64, 28.50, 30.62, 43.19, 43.62, 43.92, 45.62, 103.56, 161.45, 211.49.

Synthesis of (*R*)-1-(2,2-dimethyl-3-methylenecyclopentyl)-3-methylbutan-2-one (10e)

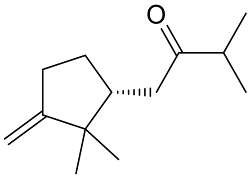

Following *GP5*. Prepared from (*R*)-2-(2,2-dimethyl-3-methylenecyclopentyl)-*N*-methoxy-*N*-methylacetamide (**9**) (2.0 mmol, 422 mg), Et_2O (7 mL), and isopropylmagnesium bromide (2.0 M in Et_2O, 3.0 mmol, 1.5 mL); column chromatography: EtOAc/petroleum ether = 1:20. Yield: 327 mg (1.68 mmol, 84 %) of colorless oil. $[\alpha]_D^{r.t.}$ = +8.0 (0.24, CH_2Cl_2). EI-HRMS: *m/z* = 195.1740 (MH^+); $C_{13}H_{23}O$ requires: *m/z* = 195.1743 (MH^+); ν_{max} 2961, 2871, 1710, 1652, 1464, 1382, 1363, 1286, 1198, 1152, 1089, 1028, 878, 793, 703 cm^{-1}. ^{1}H-NMR (500 MHz, $CDCl_3$): δ 0.84 (*s*, 3H), 1.06 (*s*, 3H), 1.10 (*d*, *J* = 7.0 Hz, 6H), 1.17 – 1.27 (*m*, 1H), 1.83 – 1.91 (*m*, 1H), 1.99 – 2.07 (*m*, 1H), 2.26 – 2.38 (*m*, 2H), 2.39 – 2.45 (*m*, 1H), 2.48 (*dd*, *J* = 3.5, 16.1 Hz, 1H), 2.63 (*hept*, *J* = 6.9 Hz, 1H), 4.77 (*d*, *J* = 2.5 Hz, 1H), 4.79 (*td*, *J* = 0.8, 2.2 Hz, 1H). ^{13}C-NMR (126 MHz, $CDCl_3$): δ 18.33, 18.45, 23.80, 26.65, 28.56, 30.62, 41.18, 41.29, 43.87, 45.45, 103.53, 161.54, 214.91.

Synthesis of (*R*)-1-(2,2-dimethyl-3-methylenecyclopentyl)pent-4-en-2-one (10f)

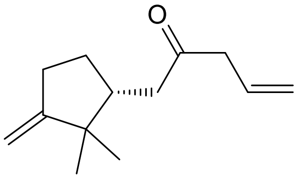

Following *GP5*. Prepared from (*R*)-2-(2,2-dimethyl-3-methylenecyclopentyl)-*N*-methoxy-*N*-methylacetamide (**9**) (2.0 mmol, 422 mg), Et_2O (7 mL), and allylmagnesium bromide (1.0 M in THF, 3.0 mmol, 3.0 mL); column chromatography: EtOAc/petroleum ether = 1:30. Yield: 319 mg (1.66 mmol, 83 %) of colorless oil. $[\alpha]_D^{r.t.}$ = +10.0 (0.25, CH_2Cl_2). EI-HRMS: *m/z* = 193.1563 (MH^+); $C_{13}H_{21}O$ requires: *m/z* = 193.1587 (MH^+); ν_{max} 3073, 2960, 1714, 1651, 1463, 1434, 1363, 1332, 1290, 1198, 1113, 1039, 992, 918, 878, 706, 663 cm^{-1}. ^{1}H-NMR (500 MHz, $CDCl_3$): δ 0.83 (*s*, 3H), 1.05 (*s*, 3H), 1.18 – 1.30 (*m*, 1H), 1.83 – 1.94 (*m*, 1H), 1.97 – 2.06 (*m*, 1H), 2.27 (*dd*, *J* = 10.5, 16.0 Hz, 1H), 2.31 – 2.39 (*m*, 1H), 2.40 – 2.48 (*m*, 1H), 2.51 (*dd*, *J* = 3.5, 16.0 Hz, 1H), 3.14 – 3.25 (*m*, 2H), 4.77 (*t*, *J* = 2.5 Hz, 1H), 4.79 (*td*, *J* = 0.8, 2.2 Hz, 1H), 5.11 – 5.23 (*m*, 2H), 5.93 (*ddt*, *J* = 7.0, 10.2, 17.2 Hz, 1H). ^{13}C-NMR (126 MHz, $CDCl_3$): δ 23.72, 26.62, 28.47, 30.59, 43.19, 43.92, 45.41, 48.28, 103.63, 118.92, 130.79, 161.30, 208.84.

Synthesis of (*R*)-1-(2,2-dimethyl-3-methylenecyclopentyl)-3-phenylpropan-2-one (10g)

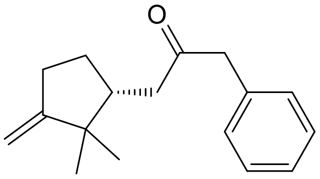

Following *GP5*. Prepared from (*R*)-2-(2,2-dimethyl-3-methylenecyclopentyl)-*N*-methoxy-*N*-methylacetamide (**9**) (2.0 mmol, 422 mg), Et_2O (7 mL), and benzylmagnesium bromide (1.0 M in THF, 3.0 mmol, 3.0 mL); column chromatography: EtOAc/petroleum ether = 1:20. Yield: 378 mg (1.56 mmol, 78 %) of colorless oil. $[\alpha]_D^{r.t.}$ = +4.0 (0.26, CH_2Cl_2). EI-HRMS: *m/z* = 243.1745 (MH^+); $C_{17}H_{23}O$ requires: *m/z* = 243.1743 (MH^+); ν_{max} 3066, 3029, 2959, 1712, 1651, 1602, 1495, 1454, 1434, 1363, 1286, 1200, 1108, 1031, 879, 748, 697 cm^{-1}. ^{1}H-NMR (500 MHz, $CDCl_3$): δ 0.77 (*s*, 3H), 1.01 (*s*, 3H), 1.09 – 1.22 (*m*, 1H), 1.79 – 1.89 (*m*, 1H), 1.95 – 2.06 (*m*, 1H), 2.24 – 2.34 (*m*, 2H), 2.36 – 2.44 (*m*, 1H), 2.51 (*dd*, *J* = 3.6, 16.0 Hz, 1H), 3.71 (*s*, 2H), 4.75 (*t*, *J* = 2.5 Hz, 1H), 4.77 (*t*, *J* = 2.2 Hz, 1H), 7.17 – 7.23 (*m*, 2H), 7.25 – 7.28 (*m*, 1H), 7.30 – 7.36 (*m*, 2H). ^{13}C-NMR (126 MHz, $CDCl_3$): δ 23.68, 26.61, 28.39, 30.57, 42.80, 43.90, 45.52, 50.65, 103.59, 127.14, 128.84, 129.55, 134.33, 161.34, 208.43.

Synthesis of (*R*)-1-(2,2-dimethyl-3-methylenecyclopentyl)but-3-en-2-one (10h)

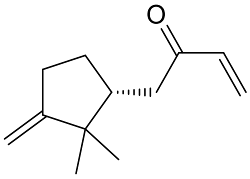

Following *GP5*. Prepared from (*R*)-2-(2,2-dimethyl-3-methylenecyclopentyl)-*N*-methoxy-*N*-methylacetamide (**9**) (2.0 mmol, 422 mg), Et_2O (7 mL), and vinylmagnesium bromide (1.0 M in THF, 3.0 mmol, 3.0 mL); column chromatography: EtOAc/petroleum ether = 1:20. Yield: 267 mg (1.50 mmol, 75 %) of colorless oil. $[\alpha]_D^{r.t.}$ = +5.0 (0.24, CH_2Cl_2). EI-HRMS: *m/z* = 179.1424 (MH^+); $C_{12}H_{19}O$ requires: *m/z* = 179.1430 (MH^+); ν_{max} 3071, 2959, 1681, 1651, 1614, 1463, 1434, 1399, 1363, 1332, 1292, 1194, 1149, 1112, 1068, 985, 961, 915, 878, 809, 634 cm^{-1}. ^1H-NMR (500 MHz, $CDCl_3$): δ 0.86 (*s*, 3H), 1.08 (*s*, 3H), 1.22 – 1.34 (*m*, 1H), 1.83 – 1.93 (*m*, 1H), 1.99 – 2.09 (*m*, 1H), 2.28 – 2.50 (*m*, 3H), 2.65 (*dd*, *J* = 3.6, 15.5 Hz, 1H), 4.78 (*t*, *J* = 2.5 Hz, 1H), 4.80 (*td*, *J* = 0.8, 2.2 Hz, 1H), 5.83 (*dd*, *J* = 1.1, 10.6 Hz, 1H), 6.23 (*dd*, *J* = 1.1, 17.6 Hz, 1H), 6.38 (*dd*, *J* = 10.6, 17.6 Hz, 1H). ^{13}C-NMR (75 MHz, $CDCl_3$): δ 23.75, 26.73, 28.49, 30.63, 40.66, 44.10, 45.95, 103.66, 128.08, 136.92, 161.38, 200.95.

Synthesis of (*R*)-1-(2,2-dimethyl-3-methylenecyclopentyl)but-3-yn-2-one (10i)

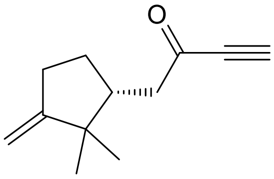

Following *GP5*. Prepared from (*R*)-2-(2,2-dimethyl-3-methylenecyclopentyl)-*N*-methoxy-*N*-methylacetamide (**9**) (2.0 mmol, 422 mg), Et_2O (7 mL), and ethynylmagnesium bromide (0.5 M in THF, 3.0 mmol, 6.0 mL); column chromatography: EtOAc/petroleum ether = 1:40. Yield: 282 mg (1.60 mmol, 80 %) of colorless oil. $[\alpha]_D^{r.t.}$ = +12.0 (0.297, CH_2Cl_2). ν_{max} 3258, 2960, 2092, 1678, 1652, 1463, 1435, 1364, 1285, 1237, 1198, 1112, 1070, 880, 807, 694, 649 cm^{-1}. ^{1}H-NMR (500 MHz, $CDCl_3$): δ 0.85 (*s*, 3H), 1.09 (*s*, 3H), 1.20 – 1.38 (*m*, 1H), 1.88 – 1.98 (*m*, 1H), 2.08 – 2.18 (*m*, 1H), 2.30 – 2.51 (*m*, 3H), 2.67 (*dd*, *J* = 3.9, 15.6 Hz, 1H), 3.23 (*s*, 1H), 4.79 (*t*, *J* = 2.5 Hz, 1H), 4.80 – 4.82 (*m*, 1H). ^{13}C-NMR (126 MHz, $CDCl_3$): δ 23.70, 26.66, 28.19, 30.53, 44.04, 45.70, 46.64, 78.57, 81.75, 103.91, 160.84, 187.51.

Synthesis of (*R*)-1-(2,2-dimethyl-3-methylenecyclopentyl)pent-3-yn-2-one (10j)

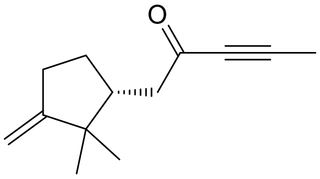

Following *GP5*. Prepared from (*R*)-2-(2,2-dimethyl-3-methylenecyclopentyl)-*N*-methoxy-*N*-methylacetamide (**9**) (2.0 mmol, 422 mg), Et_2O (7 mL), and 1-propynylmagnesium bromide (0.5 M in THF, 3.0 mmol, 6.0 mL); column chromatography: EtOAc/petroleum ether = 1:30. Yield: 263 mg (1.38 mmol, 69 %) of colorless oil. $[\alpha]_D^{r.t.}$ = +6.0 (0.243, CH_2Cl_2). EI-HRMS: m/z = 191.1430 (MH^+); $C_{13}H_{19}O$ requires: m/z = 191.1430 (MH^+); ν_{max} 2960, 2218, 1669, 1463, 1435, 1364, 1330, 1286, 1258, 1175, 1143, 1021, 972, 879, 783, 680 cm^{-1}. ^1H-NMR (500 MHz, $CDCl_3$): δ 0.84 (*s*, 3H), 1.08 (*s*, 3H), 1.18 – 1.41 (*m*, 1H), 1.85 – 1.98 (*m*, 1H), 2.01 – 2.03 (*m*, 3H), 2.05 – 2.18 (*m*, 1H), 2.24 – 2.53 (*m*, 3H), 2.61 (*dd*, J = 3.7, 15.2 Hz, 1H), 4.74 – 4.84 (*m*, 2H). ^{13}C-NMR (75 MHz, $CDCl_3$): δ 4.18, 23.70, 26.70, 28.26, 30.58, 44.05, 45.93, 46.61, 80.62, 90.05, 103.72, 161.17, 188.25.

Synthesis of (*R*)-2-(2,2-dimethyl-3-methylenecyclopentyl)-1-phenylethan-1-one (10k)

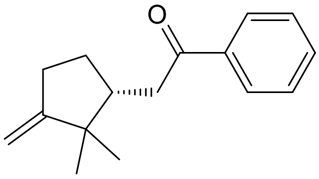

Following *GP5*. Prepared from (*R*)-2-(2,2-dimethyl-3-methylenecyclopentyl)-*N*-methoxy-*N*-methylacetamide (**9**) (2.0 mmol, 422 mg), Et_2O (7 mL), and phenylmagnesium bromide (3.0 M in THF, 3.0 mmol, 1.0 mL); column chromatography: EtOAc/petroleum ether = 1:30. Yield: 374 mg (1.64 mmol, 82 %) of colorless oil. $[\alpha]_D^{r.t.}$ = +6.0 (0.25, CH_2Cl_2). EI-HRMS: *m/z* = 229.1594 (MH^+); $C_{16}H_{21}O$ requires: *m/z* = 229.1587 (MH^+); ν_{max} 3068, 2959, 1682, 1651, 1597, 1580, 1462, 1448, 1363, 1333, 1286, 1205, 1180, 1147, 1075, 1001, 878, 750, 688, 646, 616 cm^{-1}. ^{1}H-NMR (500 MHz, $CDCl_3$): δ 0.93 (*s*, 3H), 1.13 (*s*, 3H), 1.28 – 1.39 (*m*, 1H), 1.87 – 1.97 (*m*, 1H), 2.21 – 2.21 (*m*, 1H), 2.28 – 2.40 (*m*, 1H), 2.41 – 2.51 (*m*, 1H), 2.78 (*dd*, *J* = 10.5, 15.7 Hz, 1H), 3.06 (*dd*, *J* = 3.5, 15.7 Hz, 1H), 4.77 – 4.83 (*m*, 2H), 7.46 – 7.46 (*m*, 2H), 7.53 – 7.60 (*m*, 1H), 7.93 – 8.00 (*m*, 2H). ^{13}C-NMR (126 MHz, $CDCl_3$): δ 23.87, 26.75, 28.56, 30.64, 39.40, 44.19, 46.16, 103.64, 128.26, 128.73, 133.08, 137.38, 161.47, 200.56.

Synthesis of secondary alcohols 11 from aldehyde 5 – ***General procedure 6 (GP6)***

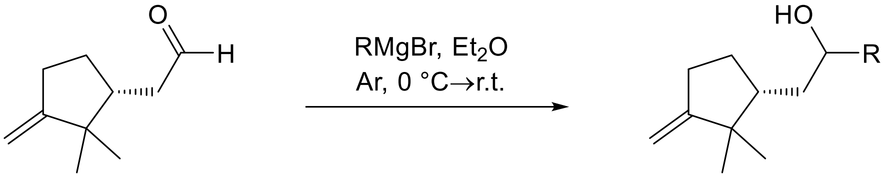

5 **11**

To a solution of aldehyde **5** (1.0 equiv.) in anhydrous Et_2O (3 mL) under argon at 0 °C, the corresponding Grignard reagent (1.4 equiv.) was added slowly. The resulting reaction mixture was stirred at 0 °C for 1 hour and then at room temperature for 20 hours. The excess Grignard reagent was quenched with NaCl (aq. sat., 3 mL) and the resulting mixture was extracted with Et_2O (3×10 mL). The combined organic phase was washed with NaCl (aq. sat., 2×5 mL), dried under anhydrous Na_2SO_4, filtered and the volatiles evaporated *in vacuo*. The residue was purified/separated by column chromatography (Silica gel 60). The fractions containing the pure product **11** were combined and the volatile components were evaporated *in vacuo*. The isolated secondary alcohols **11** were fully characterized. The two diastereomers formed could, in most cases, be partially separated by column chromatography.

Synthesis of 1-((*R*)-2,2-dimethyl-3-methylenecyclopentyl)propan-2-ol (11a/11a')

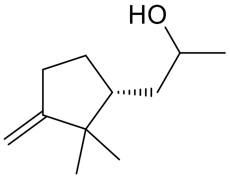

Following *GP6*. Prepared from (*R*)-2-(2,2-dimethyl-3-methylenecyclopentyl)acetaldehyde (**5**) (91.3 mg, 0.6 mmol), Et_2O (3 mL), and methylmagnesium bromide (3.0 M in Et_2O, 0.84 mmol, 0.28 mL); column chromatography: EtOAc/petroleum ether = 1:5. The two diastereomers formed could partially be separated by column chromatography. Diastereomer ratio: **11a**/**11a'** = 54:46. Diastereomer **11a** (major): Elutes first form the column. Yield: 20 mg (0.119 mmol, 20 %) of colorless oil. $[\alpha]_D^{r.t.}$ = +37.1 (0.11, CH_2Cl_2). EI-HRMS: *m/z* = 169.1586 (MH^+); $C_{11}H_{21}O$ requires: *m/z* = 169.1587 (MH^+); ν_{max} 3351, 2960, 2929, 1711, 1461, 1375, 1363, 1068, 877 cm^{-1}. ^1H-NMR (500 MHz, $CDCl_3$): δ 0.74 (*s*, 3H), 0.99 (*s*, 3H), 1.16 (*d*, *J* = 6.2 Hz, 3H), 1.13 – 1.24 (*m*, 2H), 1.32 (br *s*, 1H), 1.38 – 1.47 (*m*, 1H), 1.60 – 1.70 (*m*, 1H), 1.79 – 1.89 (*m*, 1H), 2.18 – 2.30 (*m*, 1H), 2.34 – 2.44 (*m*, 1H), 3.75 – 3.84 (*m*, 1H), 4.67 – 4.73 (*m*, 2H). ^{13}C-NMR (126 MHz, $CDCl_3$): δ 23.53, 24.87, 26.46, 28.20, 30.79, 39.67, 43.96, 46.37, 66.73, 103.05, 162.54. Diastereomer **11a'** (minor): Elutes second form the column. Yield: 15 mg (0.089 mmol, 15 %; contains 24 % of **11a**) of colorless oil. ^1H-NMR (500 MHz, $CDCl_3$): δ 0.74 (*s*, 3H), 1.16 (*d*, *J* = 6.1 Hz, 3H) (the remaining signals overlap with the signals of diastereomer **11a**).

Synthesis of 1-((*R*)-2,2-dimethyl-3-methylenecyclopentyl)pent-4-en-2-ol (11b/11b')

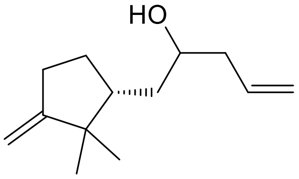

Following *GP6*. Prepared from (*R*)-2-(2,2-dimethyl-3-methylenecyclopentyl)acetaldehyde (**5**) (91.3 mg, 0.6 mmol), Et_2O (3 mL), and allylmagnesium bromide (1.0 M in THF, 0.84 mmol, 0.84 mL); column chromatography: EtOAc/petroleum ether = 1:10. The two diastereomers formed could partially be separated by column chromatography. Diastereomer ratio: **11b**/**11b'** = 48:53. Diastereomer **11b** (minor): Elutes first form the column. Yield: 32 mg (0.1647 mmol, 27 %) of colorless oil. $[\alpha]_D^{r.t.}$ = +21.0 (0.215, CH_2Cl_2). EI-HRMS: *m/z* = 195.1745 (MH^+); $C_{13}H_{23}O$ requires: *m/z* = 195.1743 (MH^+); ν_{max} 3366, 3073, 2958, 1710, 1462, 1434, 1362, 1067, 1016, 913, 877 cm^{-1}. ^{1}H-NMR (500 MHz, $CDCl_3$): δ 0.74 (*s*, 3H), 1.00 (*s*, 3H), 1.13 – 1.26 (*m*, 2H), 1.39 – 1.53 (*m*, 2H), 1.67 – 1.77 (*m*, 1H), 1.80 – 1.89 (*m*, 1H), 2.07 – 2.17 (*m*, 1H), 2.19 – 2.31 (*m*, 2H), 2.34 – 2.44 (*m*, 1H), 3.60 – 3.68 (*m*, 1H), 4.67 – 4.73 (*m*, 2H), 5.04 – 5.12 (*m*, 2H), 5.72 – 5.84 (*m*, 1H). ^{13}C-NMR (126 MHz, $CDCl_3$): δ 23.54, 26.48, 28.13, 30.79, 37.23, 43.26, 44.00, 46.20, 69.21, 103.06, 118.38, 134.98, 162.59. Diastereomer **11b'** (major): Elutes second form the column. Yield: 30 mg (0.154 mmol, 25 %; contains 19 % of **11b**) of colorless oil. ^{1}H-NMR (500 MHz, $CDCl_3$): δ 0.74 (*s*, 3H), 0.99 (*s*, 3H), 1.24 – 1.37 (*m*, 2H), 1.41 – 1.54 (*m*, 2H), 1.60 (s, 1H), 1.79 – 1.87 (*m*, 1H), 1.99 – 2.07 (*m*, 1H), 2.19 – 2.27 (*m*, 1H), 2.27 – 2.35 (*m*, 1H), 2.36 – 2.44 (*m*, 1H), 3.60 – 3.70 (*m*, 1H), 4.66 – 4.73 (*m*, 2H), 5.03 – 5.12 (*m*, 2H), 5.71 – 5.83 (*m*, 1H). ^{13}C-NMR (126 MHz, $CDCl_3$): δ 23.44, 26.52, 28.98, 30.91, 37.23, 41.69, 44.29, 47.54, 70.40, 103.14, 118.58, 134.75, 162.25.

Synthesis of 1-((*R*)-2,2-dimethyl-3-methylenecyclopentyl)-3-methylbutan-2-ol (11c/11c')

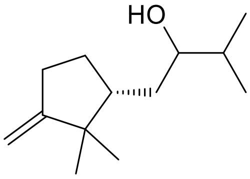

Following *GP6*. Prepared from (*R*)-2-(2,2-dimethyl-3-methylenecyclopentyl)acetaldehyde (**5**) (91.3 mg, 0.6 mmol), Et_2O (3 mL), and isopropylmagnesium bromide (2.0 M in Et_2O, 1.4 mmol, 0.7 mL); column chromatography: EtOAc/petroleum ether = 1:10. The two diastereomers formed could not be separated by column chromatography. Diastereomer ratio: **11c/11c'** = 53:47. Yield: 104 mg (0.1647 mmol, 88 %) of colorless oil. $[\alpha]_D^{r.t.}$ = +25.1 (0.28, CH_2Cl_2). EI-HRMS: *m/z* = 197.1904 (MH^+); $C_{13}H_{25}O$ requires: *m/z* = 197.1900 (MH^+); ν_{max} 3376, 2958, 2870, 1651, 1464, 1362, 1065, 994, 976, 877 cm^{-1}. ^{1}H-NMR (500 MHz, $CDCl_3$) for both diastereomers: δ 0.82 (*d*, *J* = 3.8 Hz, 3H), 0.87 – 1.00 (*m*, 6H), 1.07 (*d*, *J* = 3.8 Hz, 3H), 1.18 – 1.31 (*m*, 2H), 1.34 – 1.47 (*m*, 1H), 1.51 – 1.58 (*m*, 1H), 1.60 – 1.81 (*m*, 2H), 1.86 – 1.96 (*m*, 1H), 2.23 – 2.37 (*m*, 1H), 2.47 – 2.47 (*m*, 1H), 3.37 – 3.55 (*m*, 1H), 4.74 – 4.80 (*m*, 2H). ^{13}C-NMR (126 MHz, $CDCl_3$) for both diastereomers: δ 15.84, 17.55, 18.92, 19.45, 23.49, 23.56, 26.47, 26.72, 28.05, 29.41, 30.74, 31.02, 32.83, 34.32, 34.59, 34.90, 44.02, 44.38, 46.34, 48.04, 75.12, 76.46, 102.99, 103.10, 162.48, 162.74.

Synthesis of 1-((*R*)-2,2-dimethyl-3-methylenecyclopentyl)but-3-yn-2-ol (11d/11d')

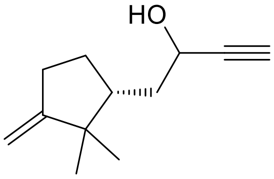

Following *GP6*. Prepared from (*R*)-2-(2,2-dimethyl-3-methylenecyclopentyl)acetaldehyde (**5**) (91.3 mg, 0.6 mmol), Et_2O (3 mL), ethynylmagnesium bromide (0.5 M in THF, 0.84 mmol, 1.68 mL); column chromatography: EtOAc/petroleum ether = 1:7. The two diastereomers formed could partially be separated by column chromatography. Diastereomer ratio: **11d**/**11d'** = 54:46. Diastereomer **11d** (major): Elutes first form the column. Yield: 26 mg (0.146 mmol, 24 %) of colorless oil. $[\alpha]_D^{r.t.}$ = +19.7 (0.14, CH_2Cl_2). EI-HRMS: *m/z* = 179.1432 (MH^+); $C_{12}H_{19}O$ requires: *m/z* = 179.143 (MH^+); ν_{max} 3348, 3073, 2965, 1732, 1327, 1181, 1045, 892 cm^{-1}. ^{1}H-NMR (500 MHz, $CDCl_3$): δ 0.76 (*s*, 3H), 1.01 (*s*, 3H), 1.21 – 1.33 (*m*, 1H), 1.45 – 1.56 (*m*, 1H), 1.68 – 1.83 (*m*, 3H), 1.84 – 1.94 (*m*, 1H), 2.19 – 2.31 (*m*, 1H), 2.35 – 2.46 (*m*, 2H), 4.33 – 4.39 (*m*, 1H), 4.68 – 4.74 (*m*, 2H). ^{13}C-NMR (126 MHz, $CDCl_3$): δ 23.57, 26.47, 28.17, 30.79, 38.57, 44.03, 45.97, 61.35, 72.92, 85.64, 103.29, 162.11. Diastereomer **11d'** (minor): Elutes second form the column. Yield: 46 mg (0.258 mmol, 43 %; contains 17 % of **11d**) of colorless oil. ^{1}H-NMR (500 MHz, $CDCl_3$): δ 0.76 (*s*, 3H), 1.02 (*s*, 3H), 1.22 – 1.32 (*m*, 1H), 1.50 – 1.58 (*m*, 1H), 1.61 – 1.68 (*m*, 1H), 1.70 – 1.77 (*m*, 1H), 1.79 – 1.88 (*m*, 1H), 1.96 (*s*, 1H), 2.19 – 2.31 (*m*, 1H), 2.35 – 2.48 (*m*, 2H), 4.32 – 4.39 (*m*, 1H), 4.68 – 4.76 (*m*, 2H). ^{13}C-NMR (126 MHz, $CDCl_3$): δ 23.63, 26.28, 28.33, 30.84, 38.36, 44.14, 46.88, 62.38, 73.45, 84.92, 103.29, 161.88.

Synthesis of tertiary alcohols 12 from ketones 10 – ***General procedure 7 (GP7)***

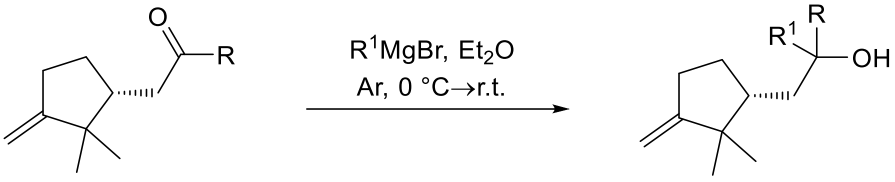

10 **12**

To a solution of ketone **10** (1.0 equiv.) in anhydrous Et_2O (4 mL) under argon at 0 °C, Grignard reagent (2.0 equiv.) was added slowly. The resulting reaction mixture was stirred at 0 °C for 1 hour and then at room temperature for 2 hours. The excess Grignard reagent was quenched with NaCl (aq. sat., 3 mL) and the resulting mixture was extracted with Et_2O (3×10 mL). The combined organic phase was washed with NaCl (aq. sat., 3×10 mL), dried under anhydrous Na_2SO_4, filtered and the volatiles evaporated *in vacuo*. The residue was purified by column chromatography (Silica gel 60). The fractions containing the pure product **12** were combined and the volatiles evaporated *in vacuo*. The isolated tertiary alcohols **12** were fully characterized (as mixtures of two diastereomers).

Synthesis of (*R*)-1-(2,2-dimethyl-3-methylenecyclopentyl)-2-methylpropan-2-ol (12a)

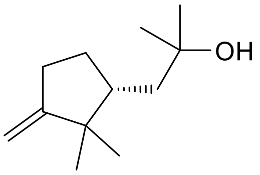

Following *GP7*. Prepared from (*R*)-1-(2,2-dimethyl-3-methylenecyclopentyl)propan-2-one (**10a**) (1.0 mmol, 166 mg), Et_2O (4 mL), and methylmagnesium bromide (2.0 M in Et_2O, 2.0 mmol, 1.0 mL); column chromatography: EtOAc/petroleum ether = 1:2. Yield: 144 mg (0.79 mmol, 79 %) of colorless oil. $[\alpha]_D^{r.t.}$ = +21.0 (0.13, CH_2Cl_2). EI-HRMS: *m/z* = 165.1637 (M-OH)H^+; $C_{12}H_{21}$ requires: *m/z* = 165.1638 (M-OH)H^+; ν_{max} 3380, 3071, 2961, 1652, 1465, 1377, 1362, 1189, 1141, 1047, 955, 905, 877, 770 cm^{-1}. ^{1}H-NMR (500 MHz, $CDCl_3$): δ 0.78 (*s*, 3H), 1.04 (*s*, 3H), 1.24 (*s*, 3H), 1.25 (*s*, 3H), 1.31 – 1.41 (*m*, 2H), 1.52 – 1.63 (*m*, 2H), 1.97 – 2.06 (*m*, 1H), 2.24 – 2.37 (*m*, 1H), 2.43 – 2.53 (*m*, 1H), 4.74 – 4.79 (*m*, 2H). ^{13}C-NMR (126 MHz, $CDCl_3$): δ 23.23, 25.72, 29.69, 30.39, 30.59, 30.97, 44.05, 44.74, 46.07, 71.49, 103.00, 161.90.

Synthesis of 1-((*R*)-2,2-dimethyl-3-methylenecyclopentyl)-2-methylbutan-2-ol (12b/12b')

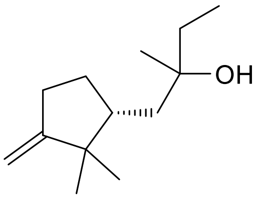

Following *GP7*. Prepared from (*R*)-1-(2,2-dimethyl-3-methylenecyclopentyl)butan-2-one (**10b**) (1.0 mmol, 180 mg), Et_2O (4 mL), and methylmagnesium bromide (2.0 M in Et_2O, 2.0 mmol, 1.0 mL); column chromatography: EtOAc/petroleum ether = 1:1. The two diastereomers formed could not be separated by column chromatography. Diastereomer ratio: 60:40. Yield: 159 mg (0.81 mmol, 81 %) of colorless oil. $[\alpha]_D^{r.t.}$ = +26.0 (0.13, CH_2Cl_2). EI-HRMS: *m/z* = 179.1796 $(M\text{-}OH)H^+$; $C_{13}H_{23}$ requires: *m/z* = 179.1794 $(M\text{-}OH)H^+$; ν_{max} 3394, 3070, 2961, 2927, 1652, 1461, 1377, 1362, 1297, 1138, 1054, 996, 920, 877, 849, 787, 756 cm^{-1}. ^1H-NMR (500 MHz, $CDCl_3$) for both diastereomers: δ 0.78 (*s*, 3H), 0.91 (*q*, *J* = 7.6 Hz, 3H), 1.04 (*s*, 3H), 1.17 (*d*, *J* = 4.4 Hz, 3H), 1.30 – 1.45 (*m*, 2H), 1.50 – 1.62 (*m*, 4H), 1.96 – 2.06 (*m*, 1H), 2.24 – 2.35 (*m*, 1H), 2.43 – 2.53 (*m*, 1H), 4.74 – 4.80 (*m*, 2H) (one signal missing). ^{13}C-NMR (126 MHz, $CDCl_3$) for both diastereomers: δ 8.27, 8.55, 23.21, 23.23, 25.76, 25.79, 26.72, 27.55, 30.34, 30.40, 31.00, 34.51, 35.50, 41.47, 41.56, 44.80, 66.81, 45.50, 45.64, 73.34, 73.39, 102.95, 161.91, 161.96.

Synthesis of 1-((*R*)-2,2-dimethyl-3-methylenecyclopentyl)-2-methylpentan-2-ol (12c/12c')

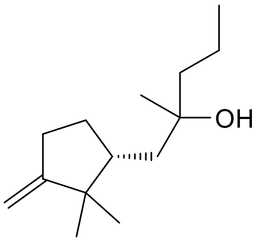

Following *GP7*. Prepared from (*R*)-1-(2,2-dimethyl-3-methylenecyclopentyl)pentan-2-one (**10c**) (1.0 mmol, 194 mg), Et_2O (4 mL), and methylmagnesium bromide (2.0 M in Et_2O, 2.0 mmol, 1.0 mL); column chromatography: EtOAc/petroleum ether = 1:5. The two diastereomers formed could not be separated by column chromatography. Diastereomer ratio: 60:40. Yield: 162 mg (0.77 mmol, 77 %) of colorless oil. $[\alpha]_D^{r.t.}$ = +13.0 (0.13, CH_2Cl_2). EI-HRMS: *m/z* = 193.1952 (M-OH)H^+; $C_{14}H_{25}$ requires: *m/z* = 193.1951 (M-OH)H^+; ν_{max} 3411, 3070, 2958, 2932, 2872, 1745, 1652, 1464, 1376, 1362, 1291, 1239, 1139, 1079, 1050, 1005, 929, 877, 775, 743 cm^{-1}. ^{1}H-NMR (500 MHz, $CDCl_3$) for both diastereomers: δ 0.78 (*s*, 3H), 0.93 (*t*, *J* = 7.2 Hz, 3H), 1.03 (*s*, 3H), 1.15 (*d*, *J* = 5.0 Hz, 1H), 1.18 (*d*, *J* = 4.6 Hz, 3H), 1.29 – 1.59 (*m*, 8H), 1.95 – 2.04 (*m*, 1H), 2.24 – 2.36 (*m*, 1H), 2.43 – 2.53 (*m*, 1H), 4.74 – 4.79 (*m*, 2H). ^{13}C-NMR (126 MHz, $CDCl_3$) for both diastereomers: δ 14.80, 14.82, 17.22, 17.48, 23.20, 23.23, 25.73, 25.76, 27.26, 28.14, 30.34, 30.40, 30.99, 41.99, 42.06, 44.58, 44.80, 44.81, 45.54, 45.57, 45.64, 73.24, 73.26, 102.95, 161.91, 161.95.

Synthesis of 1-((*R*)-2,2-dimethyl-3-methylenecyclopentyl)-2-methylhexan-2-ol (12d/12d')

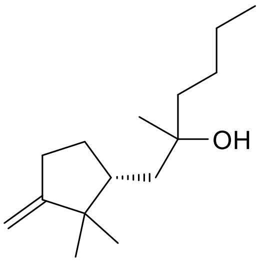

Following *GP7*. Prepared from (*R*)-1-(2,2-Dimethyl-3-methylenecyclopentyl)hexan-2-one (**10d**) (1.0 mmol, 208 mg), Et_2O (4 mL), and methylmagnesium bromide (2.0 M in Et_2O, 2.0 mmol, 1.0 mL); column chromatography: EtOAc/petroleum ether = 1:5. The two diastereomers formed could not be separated by column chromatography. Diastereomer ratio: 59:41. Yield: 153 mg (0.68 mmol, 68 %) of colorless oil. $[\alpha]_D^{r.t.}$ = +20.0 (0.25, CH_2Cl_2). EI-HRMS: *m/z* = 207.2105 $(M\text{-}OH)H^+$; $C_{15}H_{27}$ requires: *m/z* = 207.2107 $(M\text{-}OH)H^+$; ν_{max} 3404, 2958, 2931, 2869, 1652, 1462, 1376, 1362, 1297, 1139, 1085, 1025, 936, 904, 877, 776, 730 cm^{-1}. ^1H-NMR (500 MHz, $CDCl_3$) for both diastereomers: δ 0.78 (*s*, 3H), 0.89 – 0.95 (*m*, 3H), 1.04 (*s*, 3H), 1.14 (*d*, *J* = 6.0 Hz, 1H), 1.18 (*d*, *J* = 4.6 Hz, 3H), 1.28 – 1.60 (*m*, 10H), 1.96 – 2.04 (*m*, 1H), 2.24 – 2.37 (*m*, 1H), 2.43 – 2.53 (*m*, 1H), 4.74 – 4.79 (*m*, 2H). ^{13}C-NMR (126 MHz, $CDCl_3$) for both diastereomers: δ 14.29, 23.21, 23.24, 23.44, 25.75, 25.78, 26.17, 26.45, 27.28, 28.16, 30.35, 30.41, 31.00, 41.93, 41.95, 42.02, 42.97, 44.81, 44.83, 45.53, 45.65, 73.24, 73.25, 102.95, 161.93, 161.97.

Synthesis of 1-((*R*)-2,2-dimethyl-3-methylenecyclopentyl)-2-methylpent-4-en-2-ol (12e/12e')

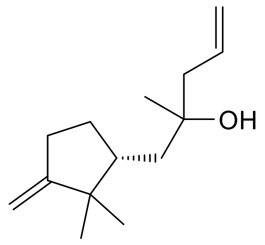

Following *GP7*. Prepared from (*R*)-1-(2,2-dimethyl-3-methylenecyclopentyl)pent-4-en-2-one (**10f**) (1.0 mmol, 192 mg), Et_2O (4 mL), and methylmagnesium bromide (2.0 M in Et_2O, 2.0 mmol, 1.0 mL); column chromatography: EtOAc/petroleum ether = 1:10. The two diastereomers formed could not be separated by column chromatography. Diastereomer ratio: 54:46. Yield: 148 mg (0.71 mmol, 71 %) of colorless oil. $[\alpha]_D^{r.t.}$ = +4.5 (0.105, CH_2Cl_2). EI-HRMS: *m/z* = 191.1791 $(M\text{-}OH)H^+$; $C_{14}H_{24}O$ requires: *m/z* = 191.1794 $(M\text{-}OH)H^+$; ν_{max} 3428, 3073, 2960, 2927, 1651, 1461, 1434, 1376, 1362, 1293, 1114, 998, 913, 877, 781, 709, 632 cm^{-1}. ^1H-NMR (500 MHz, $CDCl_3$) for both diastereomers: δ 0.78 (*s*, 3H), 1.04 (*s*, 3H), 1.20 (*d*, *J* = 5.3 Hz, 3H), 1.29 – 1.42 (*m*, 3H), 1.54 – 1.65 (*m*, 2H), 1.96 – 2.08 (*m*, 1H), 2.23 – 2.35 (*m*, 3H), 2.42 – 2.54 (*m*, 1H), 4.74 – 4.79 (*m*, 2H), 5.09 – 5.20 (*m*, 2H), 5.80 – 5.95 (*m*, 1H). ^{13}C-NMR (126 MHz, $CDCl_3$) for both diastereomers: δ 23.22, 23.24, 25.75, 25.82, 27.03, 27.96, 30.30, 30.44, 30.99, 42.06, 42.09, 44.81, 44.86, 45.47, 45.66, 46.69, 47.58, 72.52, 72.59, 102.99, 103.01, 119.04, 119.13, 134.08, 134.19, 161.85, 161.92.

Synthesis of 1-((*R*)-2,2-dimethyl-3-methylenecyclopentyl)-2-phenylpropan-2-ol (12f/12f')

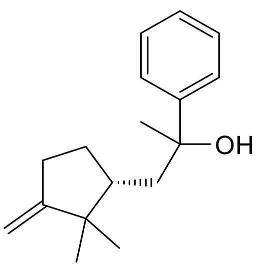

Following *GP7*. Prepared from (*R*)-2-(2,2-dimethyl-3-methylenecyclopentyl)-1-phenylethan-1-one (**10k**) (1.0 mmol, 228 mg), Et_2O (4 mL), and methylmagnesium bromide (2.0 M in Et_2O, 2.0 mmol, 1.0 mL); column chromatography: EtOAc/petroleum ether = 1:10. The two diastereomers formed could not be separated by column chromatography. Diastereomer ratio: 57:43. Yield: 178 mg (0.73 mmol, 73 %) of colorless oil. $[\alpha]_D^{r.t.}$ = +12.0 (0.11, CH_2Cl_2). EI-HRMS: *m/z* = 227.1798 $(M\text{-}OH)H^+$; $C_{17}H_{23}$ requires: *m/z* = 227.1794 $(M\text{-}OH)H^+$; ν_{max} 3439, 3065, 2959, 1651, 1494, 1446, 1362, 1111, 1068, 1028, 936, 910, 877, 848, 765, 723, 699 cm^{-1}. ^1H-NMR (500 MHz, $CDCl_3$) for both diastereomers: δ 0.75 (*s*, 1.5H), 0.76 (*s*, 1.5H), 0.88 (*s*, 1.5H), 0.98 (*s*, 1.5H), 1.02 – 1.13 (*m*, 0.5H), 1.23 – 1.39 (*m*, 1.5H), 1.48 – 1.55 (*m*, 0.5H), 1.59 (*d*, *J* = 3.5 Hz, 3H), 1.63 – 1.75 (*m*, 2.5H), 1.90 (*dd*, *J* = 1.8, 14.0 Hz, 0.5H), 1.98 (*dd*, *J* = 1.3, 14.4 Hz, 0.5H), 2.07 – 2.20 (*m*, 1H), 2.27 – 2.43 (*m*, 1H), 4.66 – 4.74 (*m*, 2H), 7.19 – 7.26 (*m*, 1H), 7.30 – 7.36 (*m*, 2H), 7.39 – 7.47 (*m*, 2H). ^{13}C-NMR (126 MHz, $CDCl_3$) for both diastereomers: δ 23.26, 23.28, 25.44, 25.62, 29.68, 29.92, 30.83, 30.92, 30.93, 31.64, 44.55, 44.66, 44.67, 44.89, 45.53, 45.79, 74.68, 75.43, 102.84, 102.88, 124.92, 124.99, 126.64, 126.67, 128.21, 128.24, 147.95, 148.61, 161.79, 161.90.

Synthesis of ethyl (*R*,*E*)-4-(2,2-dimethyl-3-methylenecyclopentyl)but-2-enoate (13)

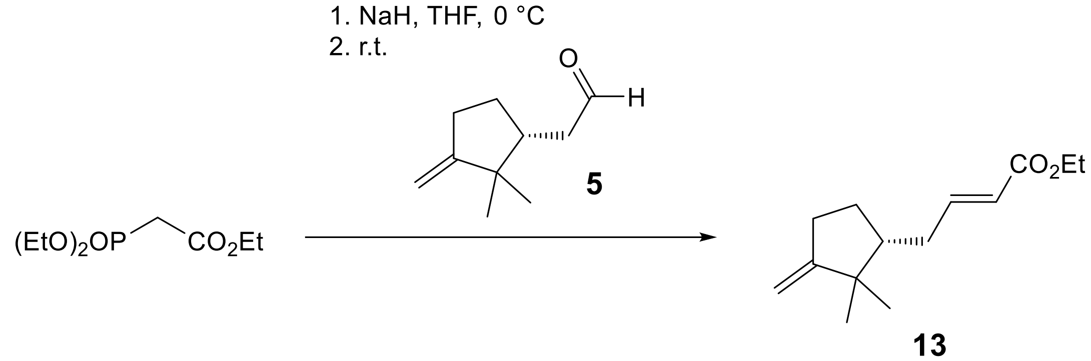

To a suspension of NaH (0.825 mmol, ω = 0.60, 33 mg) in anhydrous THF (2 mL) under argon at 0 °C triethyl phosphonoacetate (0.55 mmol, 110 µL) was added. The resulting reaction mixture was stirred at 0 °C for 45 minutes, then a solution of (*R*)-2-(2,2-dimethyl-3-methylenecyclopentyl)acetaldehyde (**5**) (84 mg, 0.55 mmol) in anhydrous THF (1.5 mL) was added. The reaction mixture was stirred at 0 °C for 1 hour and then at room temperature for 20 hours. The excess NaH was quenched with NaCl (aq. sat., 3 mL) and the mixture was extracted with EtOAc (3×10 mL). The combined organic phase was washed with NaCl (aq. sat., 2×5 mL), dried under anhydrous Na_2SO_4, filtered and the volatiles evaporated *in vacuo*. The residue was purified by column chromatography (Silica gel 60; EtOAc/petroleum ether = 1:30). The fractions containing the pure product **13** were combined and the volatiles were evaporated *in vacuo*. Yield: 80 mg (0.360 mmol, 65 %) of colorless oil. $[\alpha]_D^{r.t.}$ = +8.8 (0.13, CH_2Cl_2). EI-HRMS: *m/z* = 223.1691 (MH^+); $C_{14}H_{23}O_2$ requires: *m/z* = 223.1693 (MH^+); ν_{max} 2958, 1719, 1653, 1463, 1184, 1145, 1043, 978, 878 cm^{-1}. ^{1}H-NMR (500 MHz, $CDCl_3$): δ 0.78 (*s*, 3H), 1.01 (*s*, 3H), 1.14 – 1.28 (*m*, 1H), 1.22 (*t*, *J* = 7.2 Hz, 3H), 1.52 – 1.62 (*m*, 1H), 1.72 – 1.82 (*m*, 1H), 1.87 – 1.97 (*m*, 1H), 2.17 – 2.32 (*m*, 2H), 2.33 – 2.43 (*m*, 1H), 4.12 (*q*, *J* = 7.1 Hz, 2H), 4.70 (*t*, *J* = 2.5 Hz, 1H), 4.72 (*dt*, *J* = 1.1, 2.1 Hz, 1H), 5.77 (*dt*, *J* = 1.5, 15.6 Hz, 1H), 6.90 (*ddd*, *J* = 6.9, 7.9, 15.6 Hz, 1H). ^{13}C-NMR (126 MHz, $CDCl_3$): δ 14.43, 23.49, 26.96, 28.24, 30.56, 33.20, 44.19, 49.53, 60.32, 103.55, 122.01, 149.06, 161.90, 166.81.

Synthesis of methyl (*R*,*E*)-4-(2,2-dimethyl-3-methylenecyclopentyl)but-2-enoate (14)

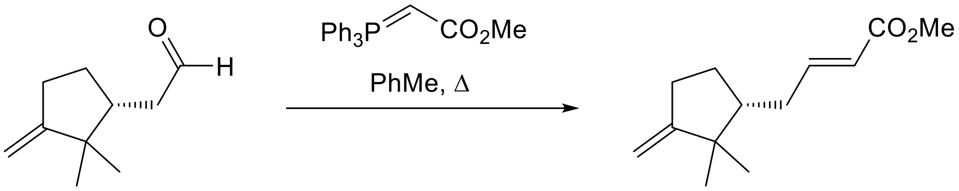

5 **14**

To a solution of (*R*)-2-(2,2-dimethyl-3-methylenecyclopentyl)acetaldehyde (**5**) (45.7 mg, 0.3 mmol) in anhydrous toluene (2 mL) under argon at room temperature methyl (triphenylphosphoranylidene)acetate (0.45 mmol, 150 mg) was added. The resulting reaction mixture was heated under reflux for 24 hours. The volatile components were evaporated *in vacuo*. The residue was purified by column chromatography (Silica gel 60; EtOAc/petroleum ether = 1:20). The fractions containing the pure product **14** were combined and the volatiles were evaporated *in vacuo*. Yield: 47 mg (0.2256 mmol, 75 %) of colorless oil. $[\alpha]_D^{r.t.}$ = +5.8 (0.135, CH_2Cl_2). EI-HRMS: *m/z* = 209.1536 (MH^+); $C_{13}H_{21}O_2$ requires: *m/z* = 209.1536 (MH^+); ν_{max} 2956, 1723, 1655, 1435, 1269 1194, 1041, 978, 878 cm^{-1}. ^{1}H-NMR (500 MHz, $CDCl_3$): δ 0.78 (*s*, 3H), 1.01 (*s*, 3H), 1.17 – 1.29 (*m*, 1H), 1.52 – 1.62 (*m*, 1H), 1.72 – 1.81 (*m*, 1H), 1.87 – 1.98 (*m*, 1H), 2.17 – 2.31 (*m*, 2H), 2.33 – 2.43 (*m*, 1H), 3.66 (*s*, 3H), 4.70 (*t*, *J* = 2.5 Hz, 1H), 4.71 – 4.74 (*m*, 1H), 5.78 (*dt*, *J* = 1.5, 15.6 Hz, 1H), 6.91 (*ddd*, *J* = 7.0, 7.9, 15.6 Hz, 1H). ^{13}C-NMR (126 MHz, $CDCl_3$): δ 23.49, 26.96, 28.23, 30.55, 33.24, 44.19, 49.53, 51.56, 103.57, 121.58, 149.42, 161.86, 167.22.

Synthesis of (*R*,*E*)-5-(2,2-dimethyl-3-methylenecyclopentyl)pent-3-en-2-one (15)

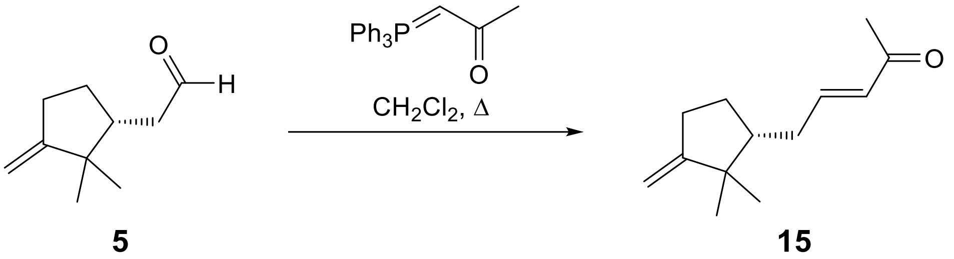

To a solution of (*R*)-2-(2,2-dimethyl-3-methylenecyclopentyl)acetaldehyde (**5**) (45.7 mg, 0.3 mmol) in anhydrous CH_2Cl_2 (2 mL) under argon at room temperature 1-triphenylphosphoranyliden-2-propanon (0.45 mmol, 143 mg) was added. The resulting reaction mixture was heated under reflux for 24 hours. The volatile components were evaporated *in vacuo*. The residue was purified by column chromatography (Silica gel 60; EtOAc/petroleum ether = 1:30). The fractions containing the pure product **15** were combined and the volatiles were evaporated *in vacuo*. Yield: 32 mg (0.166 mmol, 56 %) of colorless oil. $[\alpha]_D^{r.t.}$ = +2.2 (0.275, CH_2Cl_2). EI-HRMS: *m/z* = 193.1586 (MH^+); $C_{13}H_{21}O$ requires: *m/z* = 193.1587 (MH^+); ν_{max} 2958, 1698, 1627, 1361, 1251, 977, 878 cm^{-1}. ^{1}H-NMR (500 MHz, $CDCl_3$): δ 0.80 (*s*, 3H), 1.02 (*s*, 3H), 1.19 – 1.30 (*m*, 1H), 1.55 – 1.63 (*m*, 1H), 1.71 – 1.80 (*m*, 1H), 1.90 – 2.01 (*m*, 1H), 2.18 (*s*, 3H), 2.21 – 2.32 (*m*, 2H), 2.35 – 2.43 (*m*, 1H), 4.71 (*t*, *J* = 2.5 Hz, 1H), 4.73 (*dt*, *J* = 1.1, 2.2 Hz, 1H), 6.03 (*dt*, *J* = 1.4, 15.8 Hz, 1H), 6.74 (*ddd*, *J* = 6.8, 7.7, 15.9 Hz, 1H). ^{13}C-NMR (126 MHz, $CDCl_3$): δ 23.50, 26.99, 27.05, 28.27, 30.54, 33.52, 44.23, 49.63, 103.65, 132.02, 148.21, 161.72, 198.74.

Synthesis of (*R*,*E*)-(3-(2,2-dimethyl-3-methylenecyclopentyl)prop-1-en-1-yl)benzene (16) and (*R*,*Z*)-(3-(2,2-dimethyl-3-methylenecyclopentyl)prop-1-en-1-yl)benzene (16')

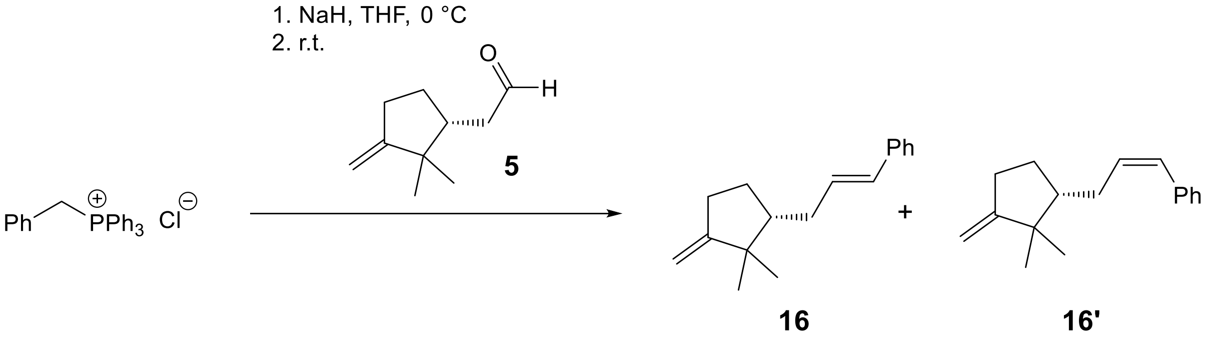

To a suspension of NaH (0.45 mmol, ω = 0.60, 18 mg) in anhydrous THF (0.5 mL) under argon at 0 °C benzyltriphenylphosphonium chloride (0.3 mmol, 117 mg) was added. The resulting reaction mixture was stirred at 0 °C for 30 minutes, then a solution of (*R*)-2-(2,2-dimethyl-3-methylenecyclopentyl)acetaldehyde (**5**) (0.3 mmol, 45.6 mg) in anhydrous THF (1.0 mL) was added. The reaction mixture was stirred at 0 °C for 1 hour and then at room temperature for 20 hours. The excess NaH was quenched with NaCl (aq. sat., 3 mL) and the mixture was extracted with EtOAc (3×10 mL). The combined organic phase was washed with NaCl (aq. sat., 2×5 mL), dried under anhydrous Na_2SO_4, filtered and the volatiles evaporated *in vacuo*. The residue was purified by column chromatography (Silica gel 60; petroleum ether). The fractions containing the pure products **16**/**16'** were combined and the volatiles were evaporated *in vacuo*. Diastereomer ratio: **16**/**16'** = 88:12. The two diastereomers formed could not be separated by column chromatography. Yield: 43 mg (0.190 mmol, 63 %) of colorless oil. $[\alpha]_D^{r.t.}$ = +38.8 (0.22, CH_2Cl_2). EI-HRMS: the product was not ionized; ν_{max} 2957, 1651, 1495, 1384, 878, 742, 692 cm^{-1}. ^{1}H-NMR (500 MHz, $CDCl_3$) for **16**: *δ* 0.82 (*s*, 3H), 1.04 (*s*, 3H), 1.23 – 1.34 (*m*, 1H), 1.53 – 1.63 (*m*, 1H), 1.77 – 1.84 (*m*, 1H), 1.89 – 1.99 (*m*, 1H), 2.18 – 2.32 (*m*, 2H), 2.34 – 2.43 (*m*, 1H), 4.69 – 4.73 (*m*, 2H), 6.15 (*ddd*, *J* = 6.8, 7.5, 15.7 Hz, 1H), 6.33 (*dt*, *J* = 1.5, 15.7 Hz, 1H), 7.10 – 7.14 (*m*, 1H), 7.20 – 7.24 (*m*, 2H), 7.26 – 7.29 (*m*, 2H). ^{13}C-NMR (126 MHz, $CDCl_3$) for **16**: *δ* 23.54, 27.12, 28.37, 30.69, 33.94, 44.17, 50.60, 103.17, 126.06, 126.95, 128.64, 130.41, 130.66, 138.03, 162.74. ^{1}H-NMR (500 MHz, $CDCl_3$) for **16'**: *δ* 0.74 (*s*, 3H), 1.02 (*s*, 3H), 2.02 – 2.09 (*m*, 1H), 4.67 – 4.69 (*m*, 1H), 5.64 (*dt*, *J* = 7.3, 11.7 Hz, 1H) (the remaining signals overlap with the signals of diastereomer **16**).

Cyclopropanation of the methylene group – ***General procedure 8 (GP8)***

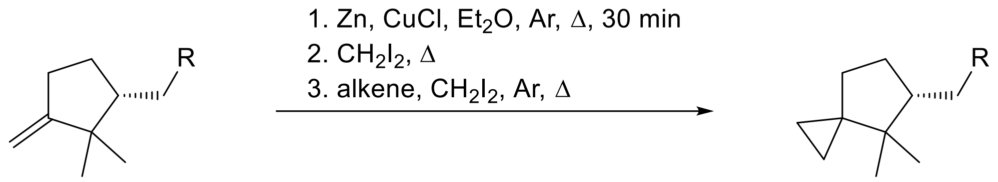

8a, **9** **17**, **18**

Zinc dust (84.0 mg, 1.285 mmol, 2.57 equiv.) and copper(I) chloride (127 mg, 1.285 mmol, 2.57 equiv.) were introduced into a flame-dried flask under argon, followed by the addition of Et_2O (3 mL). The resulting mixture was refluxed under argon for 30 minutes. Diiodomethane (53 µL, 0.65 mmol, 1.3 equiv.) was then added. The reaction mixture turned dark in color and bubbles began to form. After 5-10 minutes, alkene (**8a** or **9**) (0.5 mmol) and further diiodomethane (243 µL, 3.0 mmol, 6 equiv.) were added. The resulting reaction mixture was refluxed under argon for 20 hours. The cooled reaction mixture (room temperature) was filtered through a plague of Celite® and washed with Et_2O (30 mL) to remove the solid particles. The organic phase was washed with HCl (aq., 1 M, 2×15 mL), H_2O (2×15 mL) and NaCl (aq. sat., 3×15 mL). The organic phase was dried under anhydrous Na_2SO_4, filtered and the volatiles evaporated *in vacuo*. The residue was purified by column chromatography (Silica gel 60). The fractions containing the pure product were combined and the volatile components were evaporated *in vacuo*. The isolated cyclopropanated products **17** and **18** were fully characterized.

Synthesis of (*R*)-5-(2-methoxyethyl)-4,4-dimethylspiro[2.4]heptane (17)

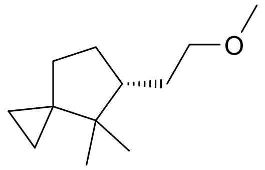

Following *GP8*. Prepared from (*R*)-2-(2-methoxyethyl)-1,1-dimethyl-5-methylenecyclopentane (**8a**) (0.5 mmol, 84 mg); column chromatography: EtOAc/petroleum ether = 1:50. Yield: 67 mg (0.367 mmol, 73 %) of colorless oil. The product contains 9 % of the starting alkene **8a**. $[\alpha]_D^{r.t.}$ = +43.3 (0.155, CH_2Cl_2). EI-HRMS: the product was not ionized; ν_{max} 2935, 2867, 1468, 1386, 1117, 1070, 841 cm^{-1}. ^{1}H-NMR (500 MHz, $CDCl_3$): δ 0.05 – 0.13 (*m*, 1H), 0.25 – 0.34 (*m*, 2H), 0.49 – 0.56 (*m*, 1H), 0.57 (*s*, 3H), 0.70 (*s*, 3H), 1.29 – 1.41 (*m*, 3H), 1.63 – 1.82 (*m*, 2H), 1.83 – 1.96 (*m*, 2H), 3.34 (*s*, 3H), 3.36 – 3.47 (*m*, 2H). ^{13}C-NMR (126 MHz, $CDCl_3$): δ 6.48, 12.27, 20.23, 22.88, 28.32, 30.67, 30.81, 33.95, 40.87, 47.26, 58.68, 72.61.

Synthesis of (*R*)-2-(4,4-dimethylspiro[2.4]heptan-5-yl)-*N*-methoxy-*N*-methylacetamide (18)

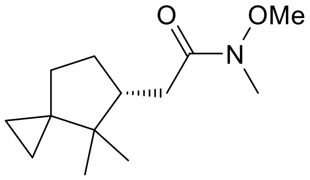

Following *GP8*. Product **18** was prepared on a 3 mmol scale. Prepared from zinc dust (504 mg, 7.71 mmol, 2.57 equiv.), copper(I) chloride (763 mg, 7.71 mmol, 2.57 equiv.), Et_2O (10 mL), diiodomethane (316 µL, 3.9 mmol, 1.3 equiv.), (*R*)-2-(2,2-dimethyl-3-methylenecyclopentyl)-*N*-methoxy-*N*-methylacetamide (**9**) (3.0 mmol, 634 mg), diiodomethane (1.461 mL, 18 mmol, 6 equiv.), washed with Et_2O (60 mL); column chromatography: EtOAc/petroleum ether = 1:5. Yield: 460 mg (2.04 mmol, 68 %) of colorless oil. The product contains 5 % of the starting alkene **9**. $[\alpha]_D^{r.t.}$ = +36.2 (0.175, CH_2Cl_2). EI-HRMS: *m/z* = 226.1800 (MH^+); $C_{13}H_{24}NO_2$ requires: *m/z* = 226.1802 (MH^+); ν_{max} 2957, 2869, 1663, 1464, 1412, 1176, 1111, 1004 cm^{-1}. ^{1}H-NMR (500 MHz, $CDCl_3$): δ 0.09 – 0.17 (*m*, 1H), 0.31 (*dd*, *J* = 7.0, 8.4 Hz, 2H), 0.47 – 0.58 (*m*, 1H), 0.62 (*s*, 3H), 0.73 (*s*, 3H), 1.30 – 1.47 (*m*, 2H), 1.80 – 1.91 (*m*, 1H), 1.96 – 2.07 (*m*, 1H), 2.14 – 2.24 (*m*, 1H), 2.26 – 2.34 (*m*, 1H), 2.49 (*dd*, *J* = 3.6, 14.6 Hz, 1H), 3.19 (*s*, 3H), 3.70 (*s*, 3H). ^{13}C-NMR (126 MHz, $CDCl_3$): δ 6.91, 12.02, 20.38, 22.93, 28.56, 30.36, 32.36, 33.40, 33.76, 40.89, 46.41, 61.30, 174.94.

Synthesis of ketones 19 from Weinreb amide 18 – ***General procedure 9 (GP9)***

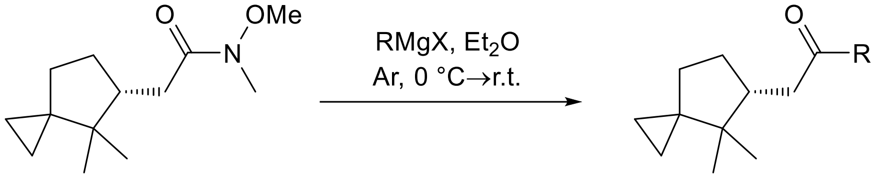

18 **19**

To a solution of (*R*)-2-(4,4-dimethylspiro[2.4]heptan-5-yl)-*N*-methoxy-*N*-methylacetamide (**18**) (1.0 equiv.; contains 5 % of alkene **9**) in anhydrous Et_2O (5 mL) under argon at 0 °C, the corresponding Grignard reagent (1.6 equiv.) was added slowly. The resulting reaction mixture was stirred at 0 °C for 1 hour and then at room temperature for 20 hours. The excess Grignard reagent was quenched with NaCl (aq. sat., 3 mL) and the resulting mixture was extracted with Et_2O (3×15 mL). The combined organic phase was washed with NaCl (aq. sat., 3×5 mL), dried under anhydrous Na_2SO_4, filtered and the volatiles evaporated *in vacuo*. The residue was purified by column chromatography (Silica gel 60). The fractions containing the pure product **19** were combined and the volatiles evaporated *in vacuo*. The isolated ketones **19** were fully characterized.

Synthesis of (*R*)-1-(4,4-dimethylspiro[2.4]heptan-5-yl)propan-2-one (19a)

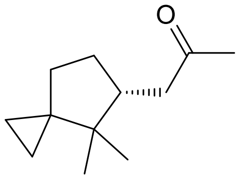

Following *GP9*. Prepared from (*R*)-2-(4,4-dimethylspiro[2.4]heptan-5-yl)-*N*-methoxy-*N*-methylacetamide (**18**) (0.5 mmol, 113 mg), Et_2O (5 mL), and methylmagnesium bromide (3 M in Et_2O, 0.8 mmol, 267 μL); column chromatography: EtOAc/petroleum ether = 1:20. Yield: 80 mg (0.444 mmol, 88 %) of colorless oil. The product contains 4 % of the alkene **9**. $[\alpha]_D^{r.t.}$ = +16.2 (0.28, CH_2Cl_2). EI-HRMS: *m/z* = 181.1586 (MH^+); $C_{12}H_{21}O$ requires: *m/z* = 181.1587 (MH^+); ν_{max} 2958, 2869, 1714, 1468, 1365, 1287, 1163, 1145, 1011 cm^{-1}. ^1H-NMR (500 MHz, $CDCl_3$): δ 0.09 – 0.17 (*m*, 1H), 0.27 – 0.36 (*m*, 2H), 0.50 – 0.57 (*m*, 1H), 0.59 (*s*, 3H), 0.70 (*s*, 3H), 1.22 – 1.34 (*m*, 1H), 1.37 – 1.46 (*m*, 1H), 1.83 – 1.93 (*m*, 1H), 1.91 – 2.03 (*m*, 1H), 2.08 – 2.15 (*m*, 1H), 2.17 (*s*, 3H), 2.28 (*dd*, *J* = 10.7, 15.5 Hz, 1H), 2.51 (*dd*, *J* = 3.5, 15.6 Hz, 1H). ^{13}C-NMR (126 MHz, $CDCl_3$): δ 6.83, 12.03, 20.43, 22.97, 28.47, 30.28, 30.44, 33.74, 40.88, 45.63, 45.99, 209.66.

Synthesis of (*R*)-1-(4,4-dimethylspiro[2.4]heptan-5-yl)pent-4-en-2-one (19b)

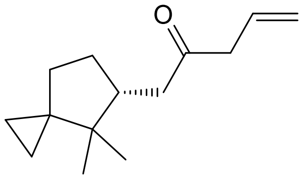

Following *GP9*. Prepared from (*R*)-2-(4,4-dimethylspiro[2.4]heptan-5-yl)-*N*-methoxy-*N*-methylacetamide (**18**) (0.5 mmol, 113 mg), Et_2O (5 mL), and allylmagnesium bromide (1.0 M in Et_2O, 0.8 mmol, 0.8 mL); column chromatography: EtOAc/petroleum ether = 1:50. Yield: 73 mg (0.354 mmol, 70 %) of colorless oil. The product contains 3 % of the alkene **9**. $[\alpha]_D^{r.t.}$ = +21.1 (0.25, CH_2Cl_2). EI-HRMS: *m/z* = 207.1784 (MH^+); $C_{14}H_{23}O$ requires: *m/z* = 207.1785 (MH^+); ν_{max} 2958, 2869, 1714, 1638, 1468, 1385, 1365, 1012, 991, 917 cm^{-1}. ^{1}H-NMR (500 MHz, $CDCl_3$): δ 0.09 – 0.16 (*m*, 1H), 0.26 – 0.36 (*m*, 2H), 0.50 – 0.57 (*m*, 1H), 0.58 (*s*, 3H), 0.69 (*s*, 3H), 1.20 – 1.34 (*m*, 1H), 1.37 – 1.46 (*m*, 1H), 1.82 – 1.91 (*m*, 1H), 1.92 – 2.03 (*m*, 1H), 2.09 – 2.19 (*m*, 1H), 2.30 (*dd*, *J* = 10.7, 15.8 Hz, 1H), 2.52 (*dd*, *J* = 3.5, 15.8 Hz, 1H), 3.14 – 3.26 (*m*, 2H), 5.11 – 5.22 (*m*, 2H), 5.94 (*ddt*, *J* = 7.0, 10.2, 17.2 Hz, 1H). ^{13}C-NMR (126 MHz, $CDCl_3$): δ 6.81, 12.04, 20.43, 22.96, 28.51, 30.27, 33.73, 40.87, 44.15, 45.78, 48.20, 118.81, 130.94, 209.21.

Synthesis of (*R*)-1-(4,4-dimethylspiro[2.4]heptan-5-yl)but-3-en-2-one (19c)

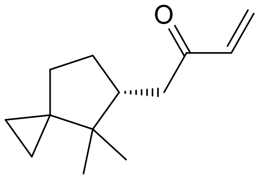

Following *GP9*. Prepared from (*R*)-2-(4,4-dimethylspiro[2.4]heptan-5-yl)-*N*-methoxy-*N*-methylacetamide (**18**) (0.5 mmol, 113 mg), Et_2O (5 mL), and vinylmagnesium bromide (1.0 M in THF, 0.8 mmol, 0.8 mL); column chromatography: EtOAc/petroleum ether = 1:40. Yield: 65 mg (0.338 mmol, 67 %) of colorless oil. $[\alpha]_D^{r.t.}$ = +36.6 (0.155, CH_2Cl_2). EI-HRMS: *m/z* = 193.1584 (MH^+); $C_{13}H_{21}O$ requires: *m/z* = 193.1587 (MH^+); ν_{max} 2958, 2869, 1680, 1615, 1400, 1365, 1011, 985, 958 cm^{-1}. ^{1}H-NMR (500 MHz, $CDCl_3$): δ 0.10 – 0.17 (*m*, 1H), 0.28 – 0.36 (*m*, 2H), 0.51 – 0.58 (*m*, 1H), 0.61 (*s*, 3H), 0.73 (*s*, 3H), 1.24 – 1.36 (*m*, 1H), 1.37 – 1.46 (*m*, 1H), 1.83 – 1.90 (*m*, 1H), 1.91 – 2.01 (*m*, 1H), 2.12 – 2.22 (*m*, 1H), 2.44 (*dd*, *J* = 10.7, 15.2 Hz, 1H), 2.67 (*dd*, *J* = 3.7, 15.2 Hz, 1H), 5.81 (*dd*, *J* = 1.2, 10.6 Hz, 1H), 6.23 (*dd*, *J* = 1.2, 17.6 Hz, 1H), 6.39 (*dd*, *J* = 10.6, 17.6 Hz, 1H). ^{13}C-NMR (126 MHz, $CDCl_3$): δ 6.86, 12.06, 20.45, 23.00, 28.46, 30.30, 33.73, 41.02, 41.53, 46.23, 127.99, 136.92, 201.35.

Synthesis of (*R*)-2-(4,4-dimethylspiro[2.4]heptan-5-yl)-1-phenylethan-1-one (19d)

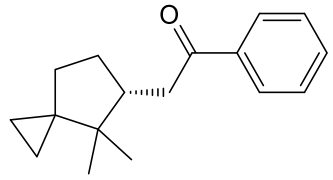

Following *GP9*. Prepared from (*R*)-2-(4,4-dimethylspiro[2.4]heptan-5-yl)-*N*-methoxy-*N*-methylacetamide (**18**) (0.5 mmol, 113 mg), Et_2O (5 mL), and phenylmagnesium bromide (3.0 M in THF, 0.8 mmol, 267 μL); column chromatography: EtOAc/petroleum ether = 1:20. Yield: 76 mg (0.315 mmol, 63 %) of colorless oil. The product contains 3 % of the alkene **9**. $[\alpha]_D^{r.t.}$ = +40.1 (0.235, CH_2Cl_2). EI-HRMS: *m/z* = 243.1744 (MH^+); $C_{17}H_{23}O$ requires: *m/z* = 243.1743 (MH^+); ν_{max} 2957, 2868, 1682, 1448, 1365, 1286, 1213, 1045, 750, 689 cm^{-1}. ^{1}H-NMR (500 MHz, $CDCl_3$): δ 0.10 – 0.18 (*m*, 1H), 0.29 – 0.37 (*m*, 2H), 0.51 – 0.59 (*m*, 1H), 0.66 (*s*, 3H), 0.80 (*s*, 3H), 1.29 – 1.46 (*m*, 2H), 1.83 – 1.94 (*m*, 1H), 2.02 – 2.04 (*m*, 1H), 2.23 – 2.34 (*m*, 1H), 2.80 (*dd*, *J* = 10.5, 15.4 Hz, 1H), 3.08 (*dd*, *J* = 3.5, 15.5 Hz, 1H), 7.41 – 7.51 (*m*, 2H), 7.52 – 7.62 (*m*, 1H), 7.93 – 8.00 (*m*, 2H). ^{13}C-NMR (126 MHz, $CDCl_3$): δ 6.89, 12.11, 20.58, 23.08, 28.58, 30.37, 33.77, 40.32, 41.15, 46.54, 128.29, 128.71, 132.99, 137.50, 200.93.

Synthesis of (3*R*)-1-methoxy-3-(2-methoxyethyl)-1,2,2-trimethylcyclopentane (20)

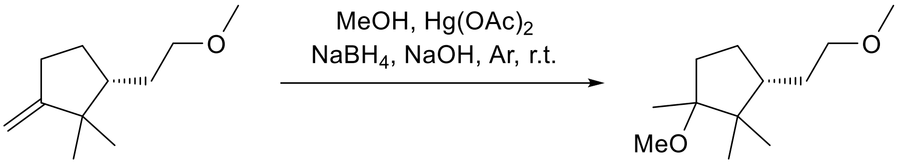

8a **20**

To a solution/suspension of $Hg(OAc)_2$ (0.65 mmol, 207 mg) in anhydrous methanol (1.5 mL) at 0 ºC under argon was added (*R*)-2-(2-methoxyethyl)-1,1-dimethyl-5-methylenecyclopentane (**8a**) (0.5 mmol, 84 mg). After stirring at 0 °C for 15 minutes, NaOH (aq., 3 M, 0.5 mL) was added. The reaction mixture turned from colorless to yellow. Then a solution/suspension of $NaBH_4$ (5.25 mmol, 199 mg) in NaOH (aq., 3 M, 0.5 mL) was added. The reaction mixture changed color from yellow to dark blue (black). The reaction mixture was stirred at room temperature under argon for 20 hours. The reaction mixture was filtered through a plague of Celite® and washed with EtOAc (20 mL). The filtrate was washed with H_2O (2×5 mL) and NaCl (aq. sat., 2×5 mL), dried under anhydrous Na_2SO_4, filtered and the volatiles evaporated *in vacuo*. The residue was purified by column chromatography (Silica gel 60, EtOAc/petroleum ether = 1:10). The fractions containing the pure product **20** were combined and the volatile components were evaporated *in vacuo*. Yield: 66 mg (0.330 mmol, 66 %) of colorless oil. $[\alpha]_D^{r.t.}$ = +25.2 (0.15, CH_2Cl_2). EI-HRMS: the product was not ionized; ν_{max} 2935, 2867, 1468, 1386, 1117, 1070, 841 cm^{-1}. ^{1}H-NMR (500 MHz, $CDCl_3$): δ 0.67 (*s*, 3H), 0.87 (*s*, 3H), 1.06 (*s*, 3H), 1.15 – 1.24 (*m*, 1H), 1.28 – 1.37 (*m*, 1H), 1.37 – 1.46 (*m*, 1H), 1.64 – 1.88 (*m*, 2H), 1.90 – 2.06 (*m*, 2H), 3.13 (*s*, 3H), 3.33 (*s*, 3H), 3.35 – 3.43 (*m*, 2H). ^{13}C-NMR (126 MHz, $CDCl_3$): δ 16.03, 19.56, 19.68, 27.40, 29.98, 31.08, 43.14, 47.62, 49.43, 58.64, 72.97, 87.74.

Synthesis of (3a*R*,6a*S*)-6,6,6a-trimethylhexahydro-2*H*-cyclopenta[*b*]furan (21)[10]

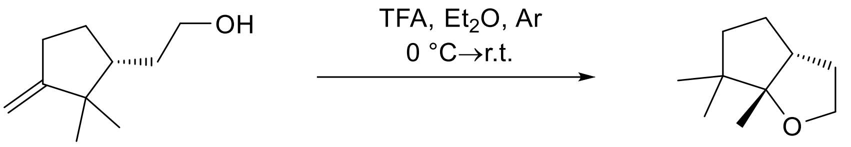

4 **21**

To a solution of (*R*)-2-(2,2-dimethyl-3-methylenecyclopentyl)ethan-1-ol (**4**) (2.0 mmol, 308 mg) in anhydrous Et_2O (4 mL) under argon at 0 °C was added TFA (4 mL). The resulting reaction mixture was stirred at 0 °C for 1 hour and at room temperature for 1 hour. The volatiles were thoroughly evaporated *in vacuo*. The residue was purified by column chromatography (Silica gel 60, EtOAc/petroleum ether = 1:20). The fractions containing the pure product **21** were combined and the volatile components were evaporated *in vacuo*. The isolated ether **21** was fully characterized with the exception of HRMS, as the product was not ionized. Yield: 118 mg (0.76 mmol, 38 %) of colorless oil. $[\alpha]_D^{r.t.}$ = +5.0 (0.23, CH_2Cl_2). ν_{max} 2955, 2864, 1785, 1460, 1398, 1348, 1218, 1141, 943, 821, 776, 731 cm^{-1}. ^{1}H-NMR (500 MHz, $CDCl_3$): δ 0.85 (*s*, 3H), 1.01 (*s*, 3H), 1.08 (*s*, 3H), 1.15 – 1.25 (*m*, 1H), 1.28 – 1.36 (*m*, 1H), 1.61 – 1.70 (*m*, 2H), 1.91 – 2.03 (*m*, 1H), 2.12 – 2.22 (*m*, 1H), 2.32 – 2.40 (*m*, 1H), 3.70 – 3.79 (*m*, 1H), 3.89 (*td*, *J* = 3.6, 8.2 Hz, 1H). ^{13}C-NMR (126 MHz, $CDCl_3$): δ 19.10, 22.24, 25.49, 29.71, 34.89, 39.81, 45.96, 47.22, 67.97, 95.08.

2. Copies of ^{1}H- and ^{13}C-NMR spectra

(1*R*,4*R*)-1-(Iodomethyl)-7,7-dimethylbicyclo[2.2.1]heptan-2-one (2)

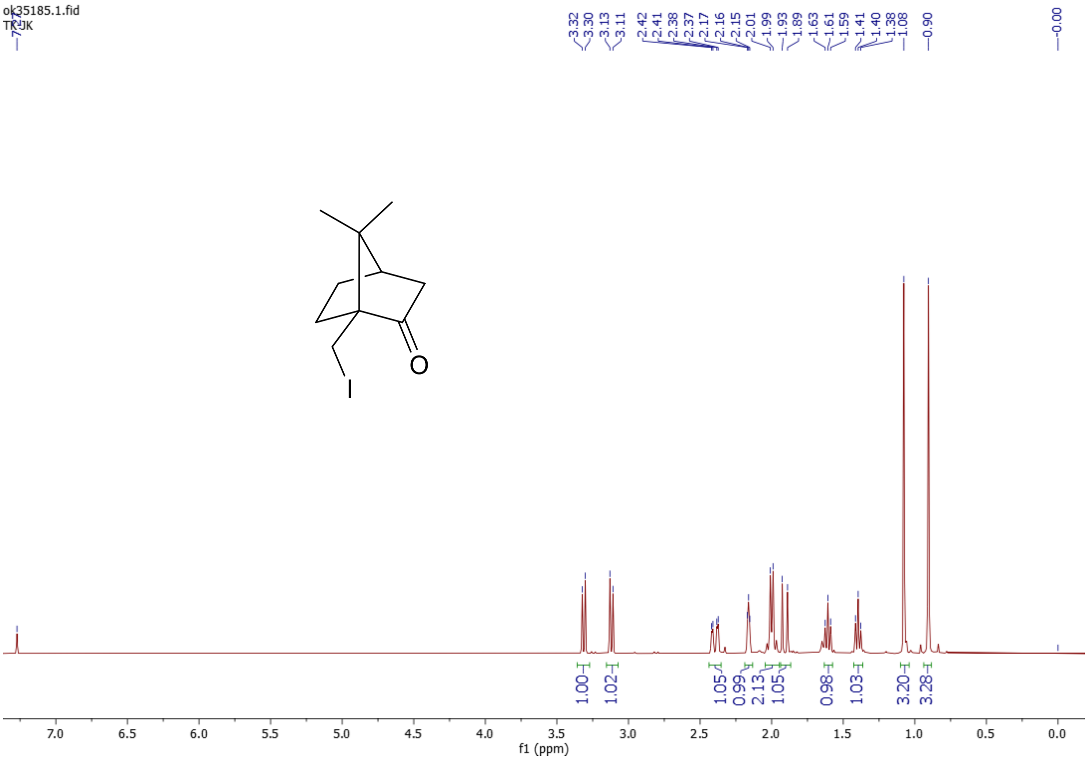

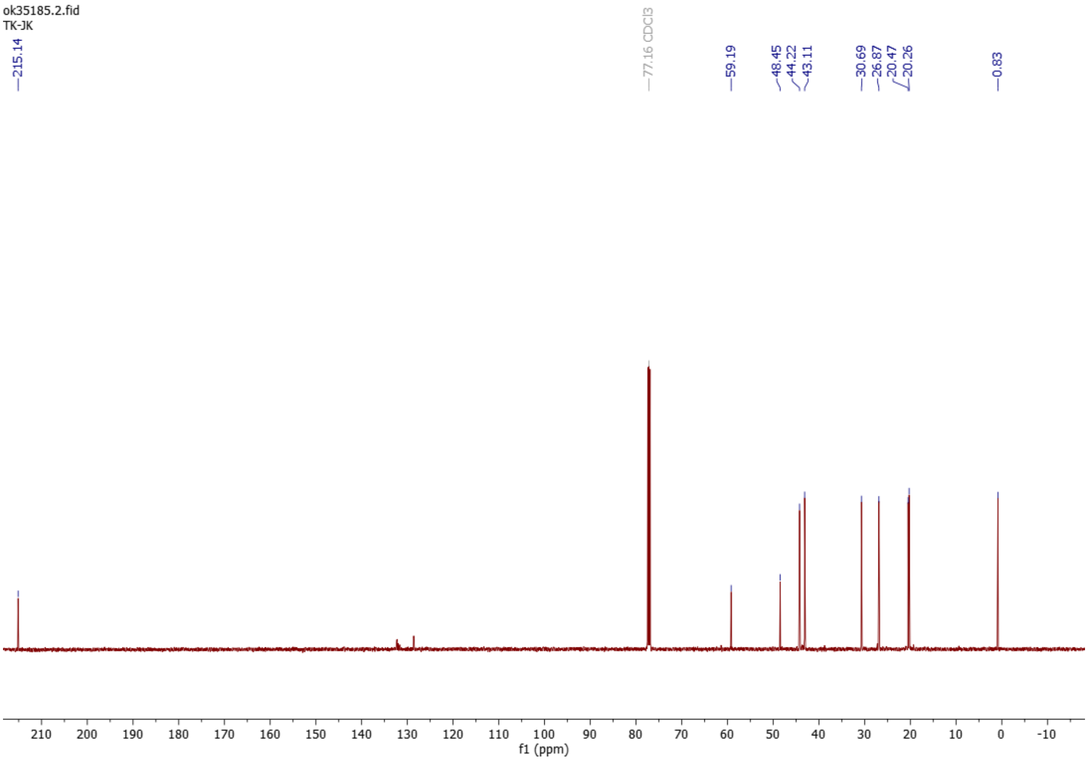

(*R*)-2-(2,2-Dimethyl-3-methylenecyclopentyl)acetic acid (3)

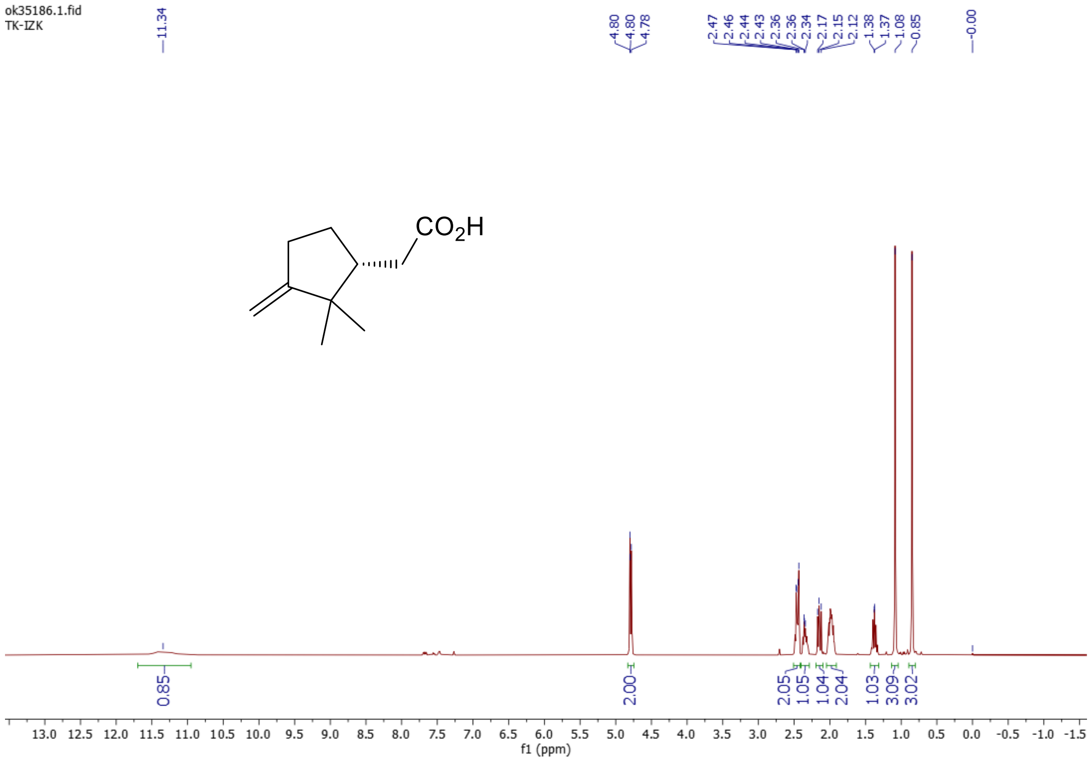

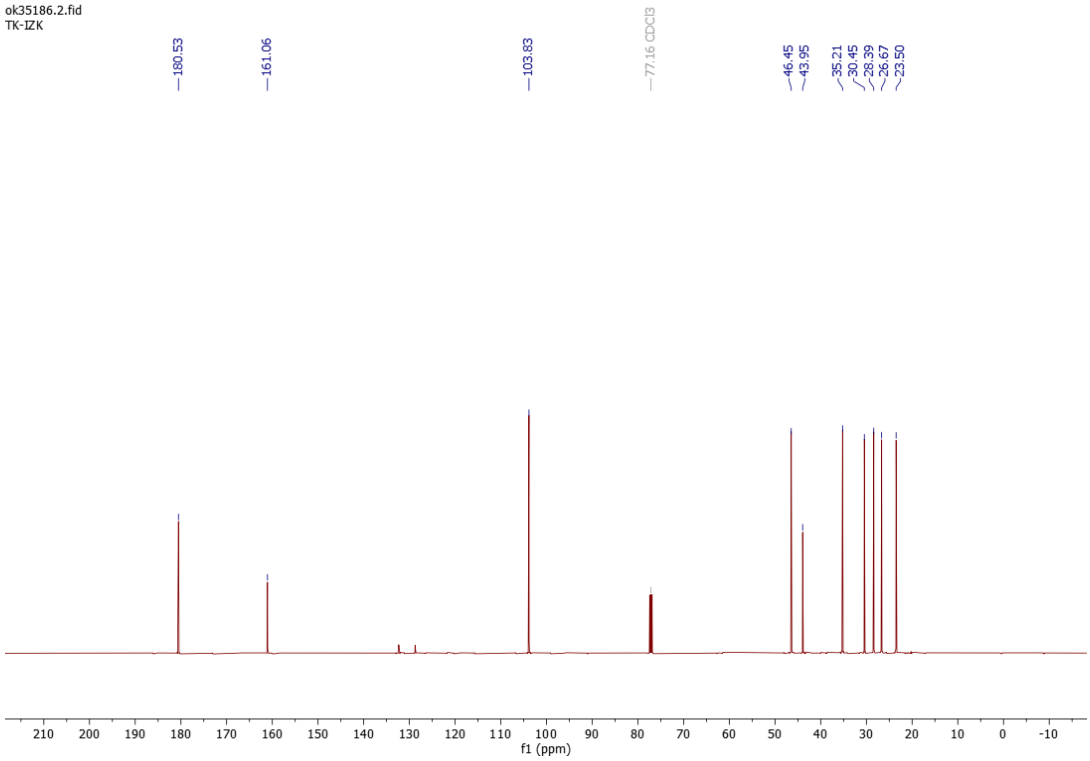

(*R*)-2-(2,2-Dimethyl-3-methylenecyclopentyl)ethan-1-ol (4)

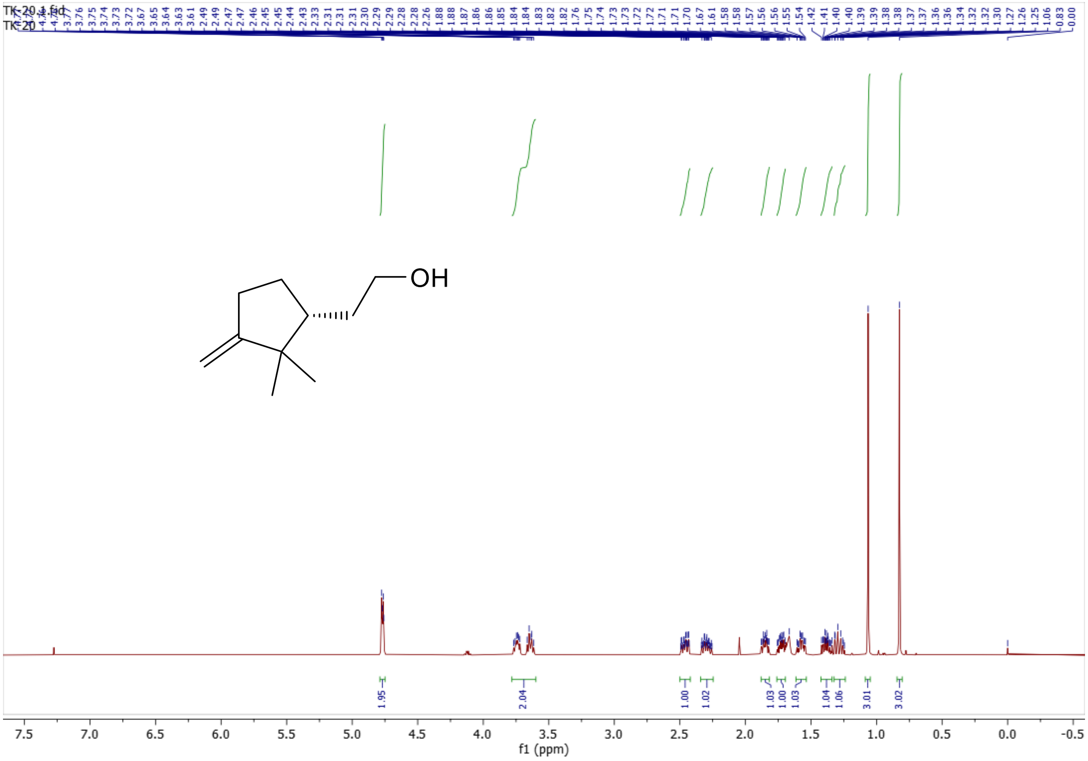

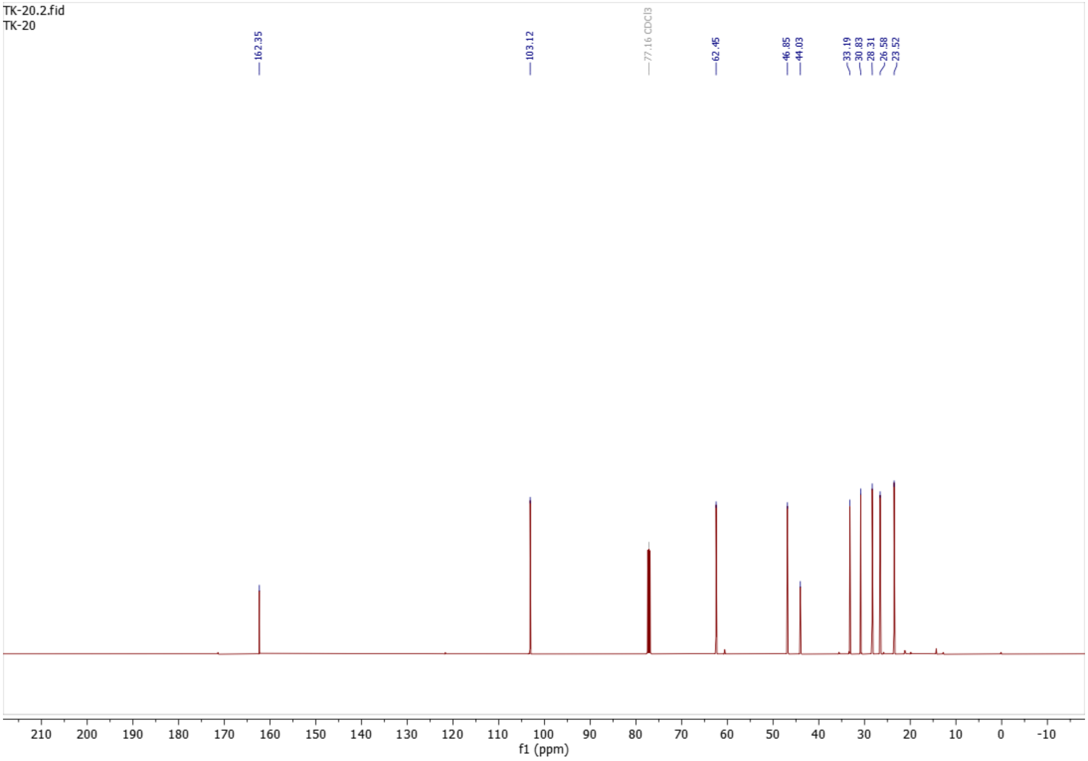

(*R*)-2-(2,2-Dimethyl-3-methylenecyclopentyl)acetaldehyde (5)

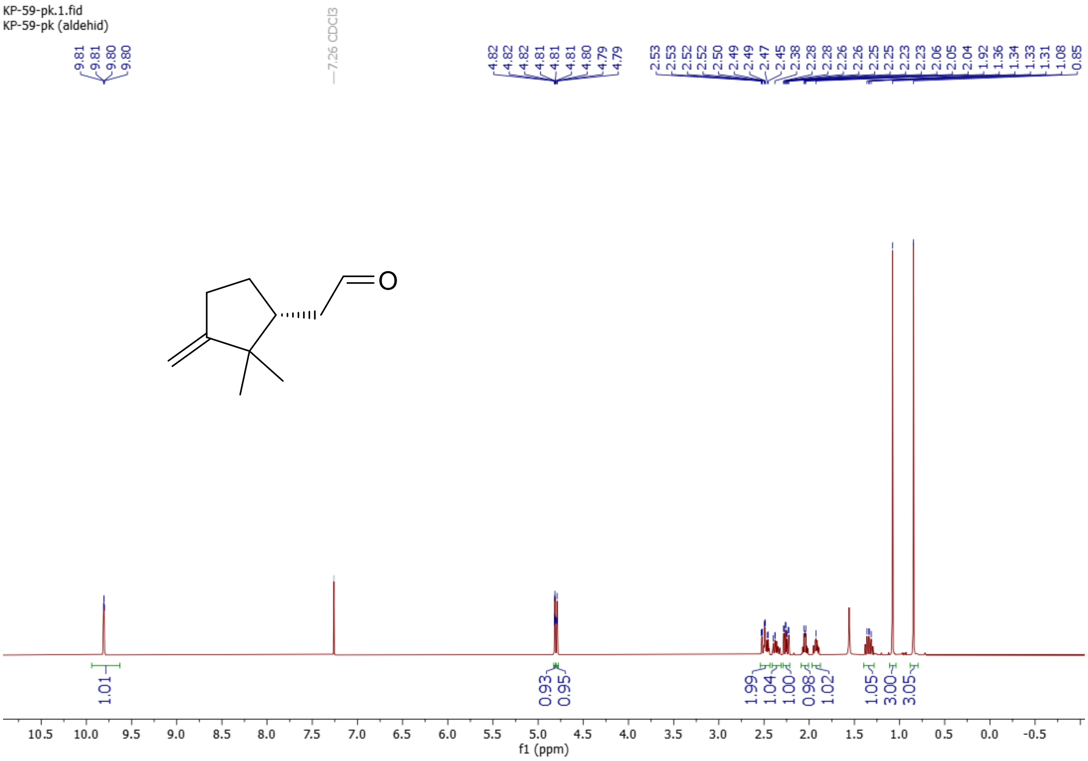

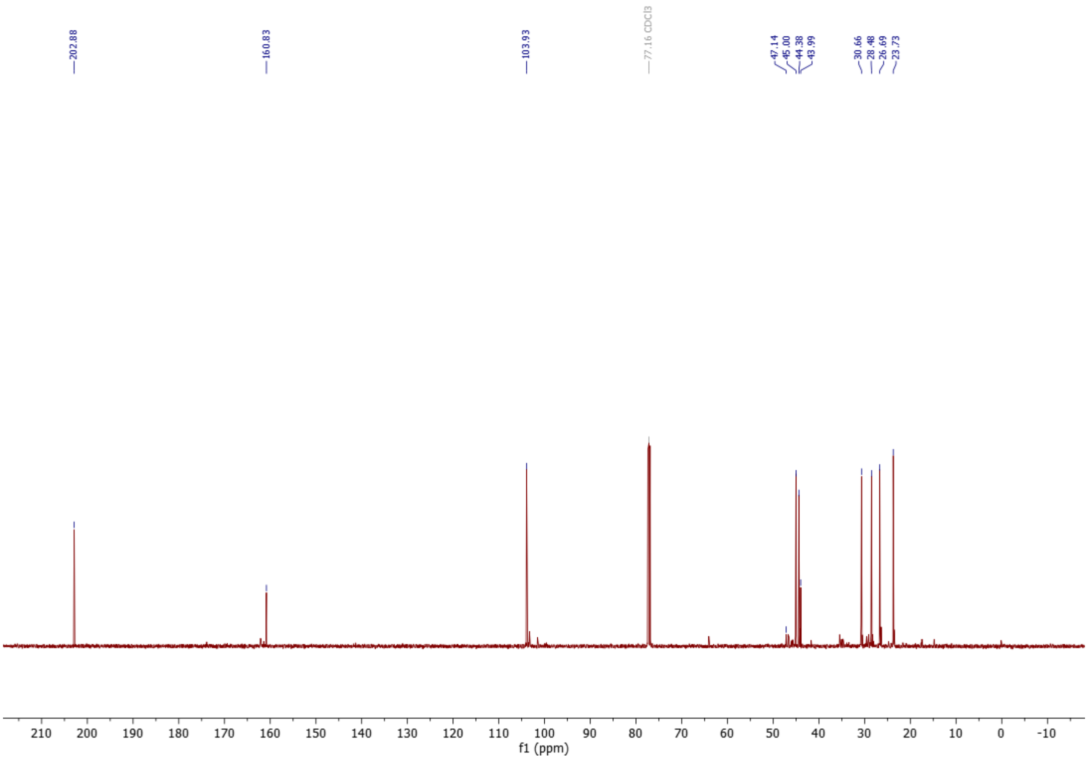

Methyl (*R*)-2-(2,2-dimethyl-3-methylenecyclopentyl)acetate (6a)

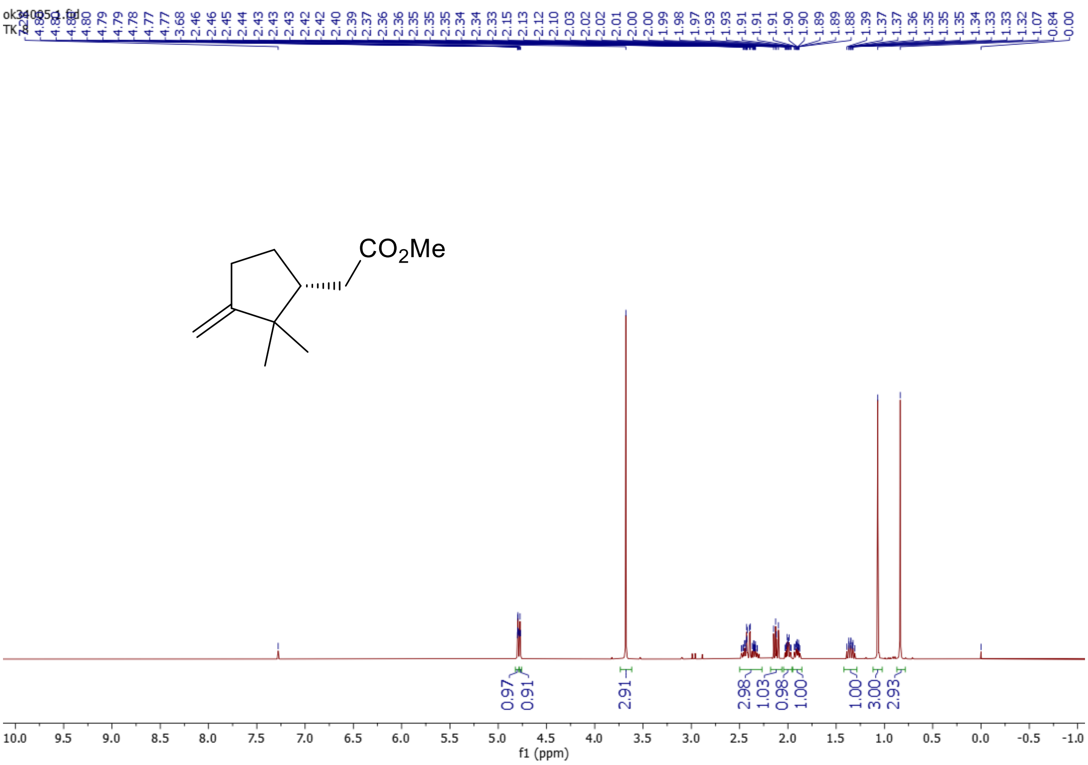

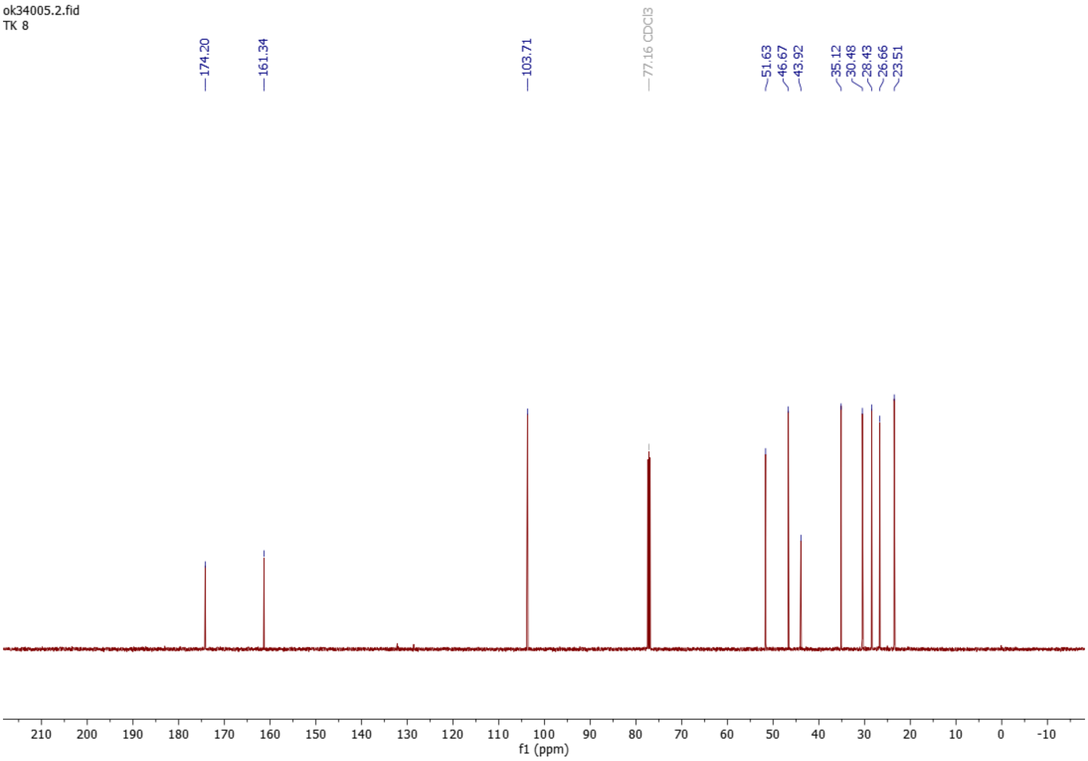

Ethyl (*R*)-2-(2,2-dimethyl-3-methylenecyclopentyl)acetate (6b)

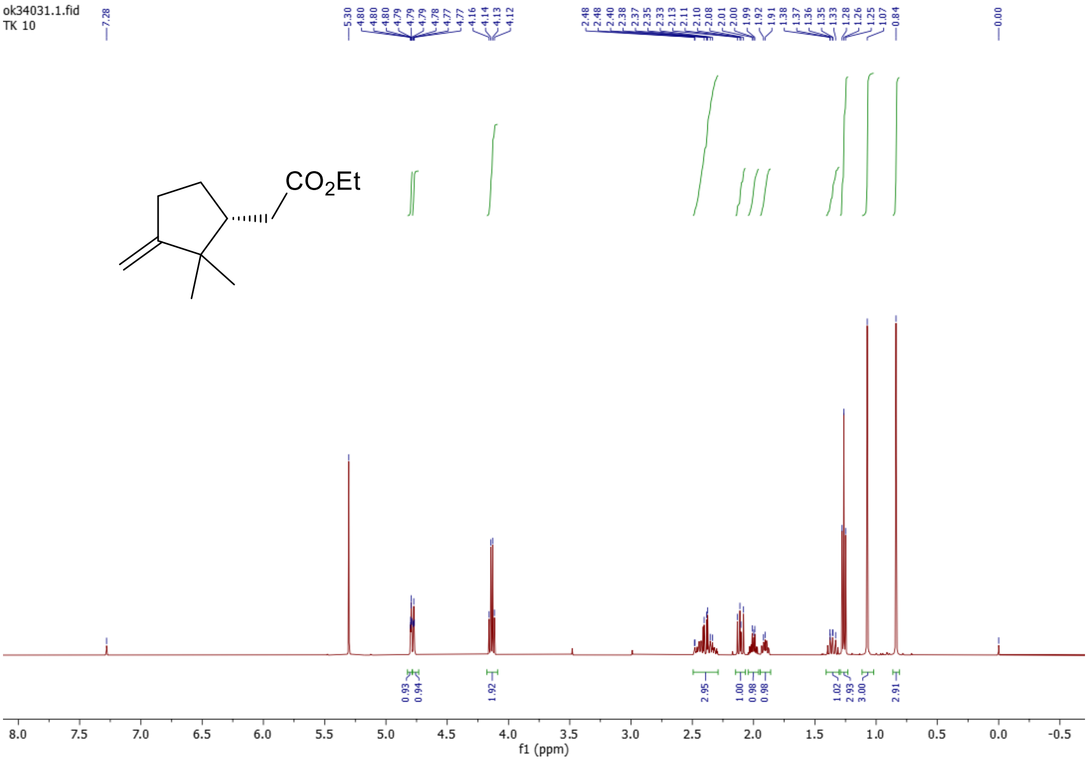

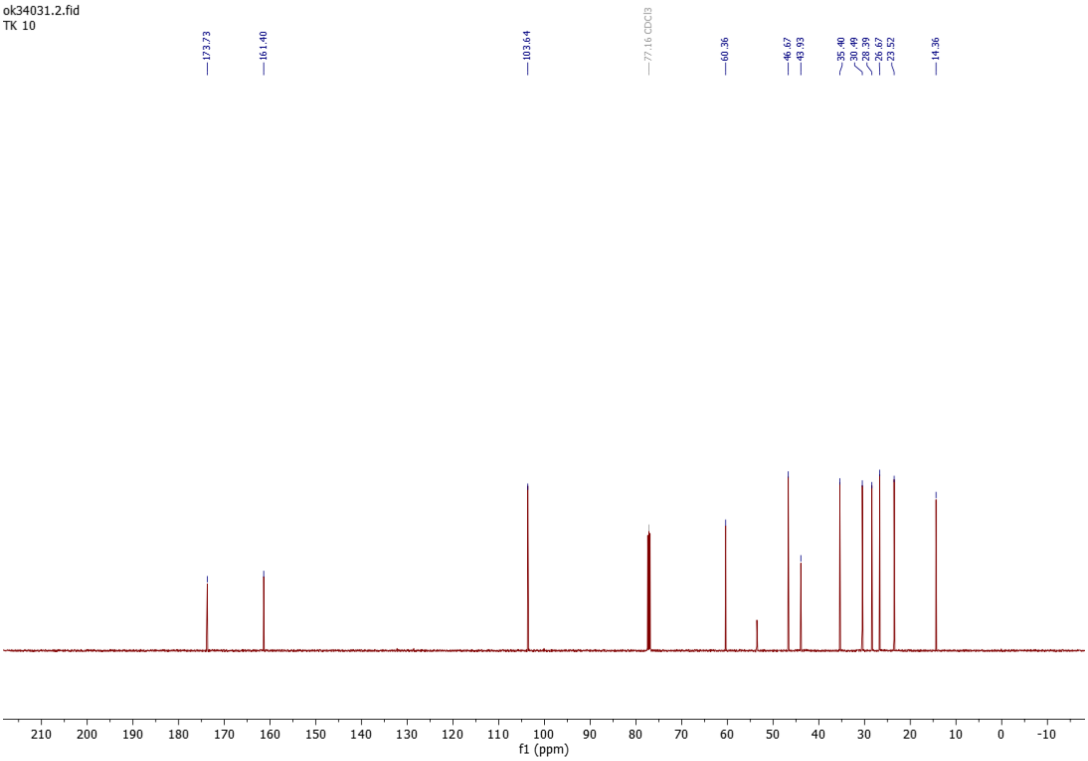

Propyl (*R*)-2-(2,2-dimethyl-3-methylenecyclopentyl)acetate (6c)

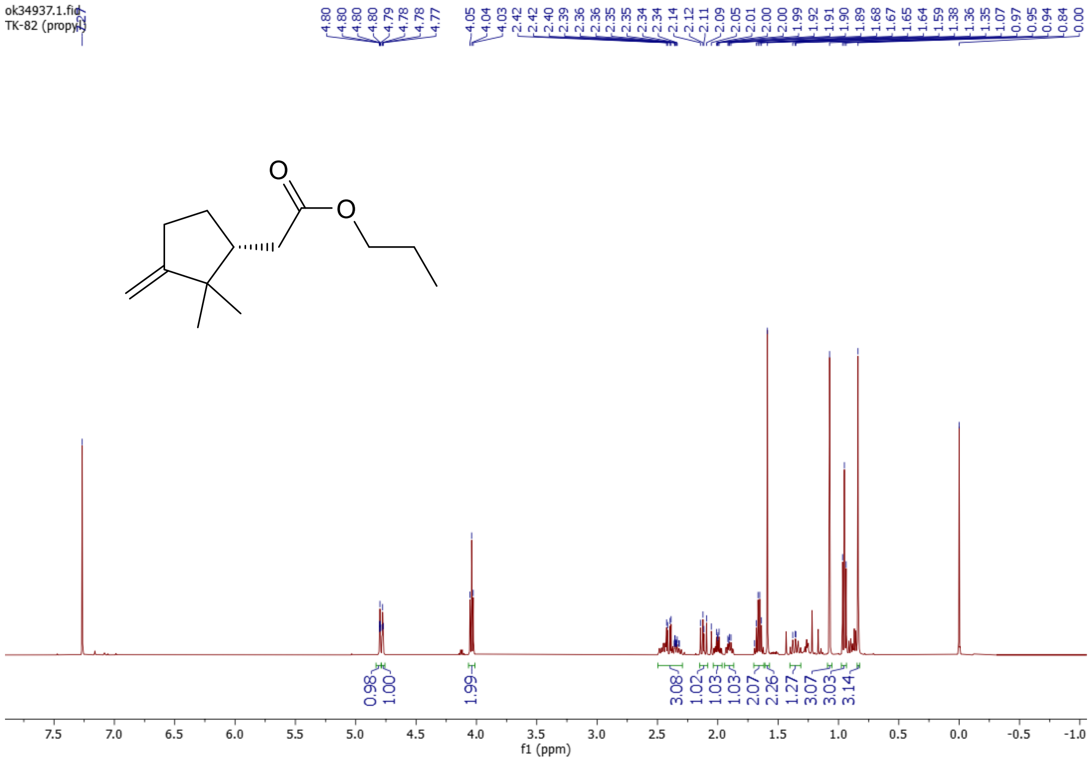

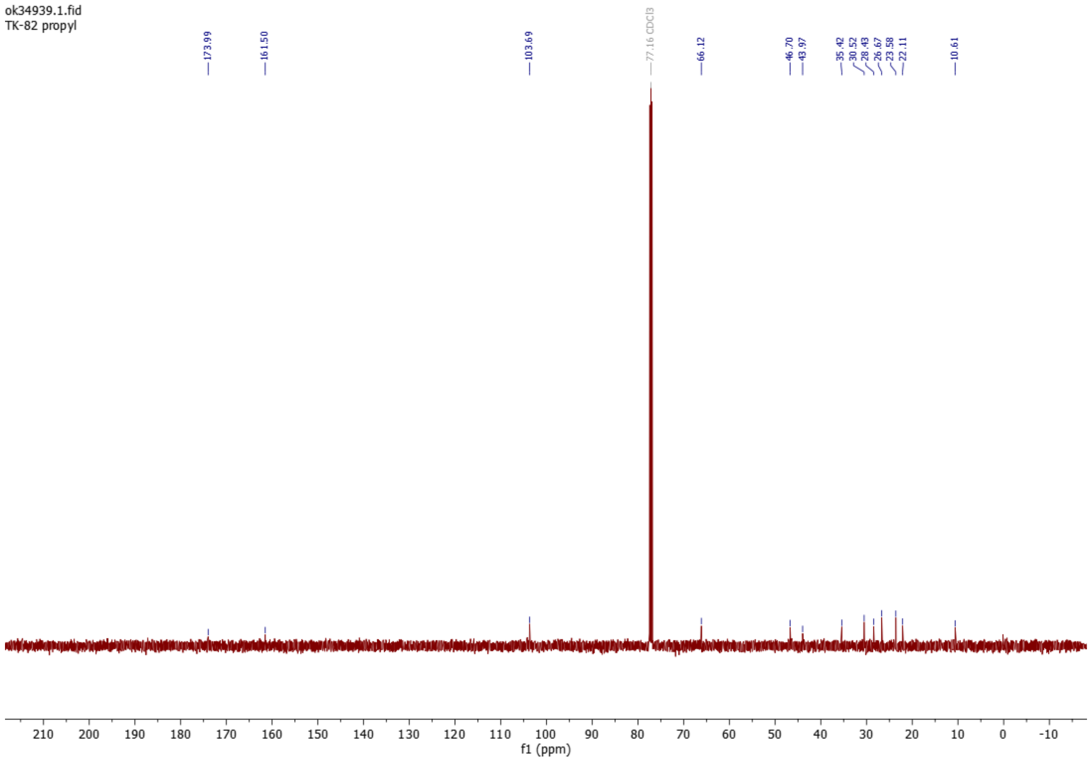

Butyl (*R*)-2-(2,2-dimethyl-3-methylenecyclopentyl)acetate (6d)

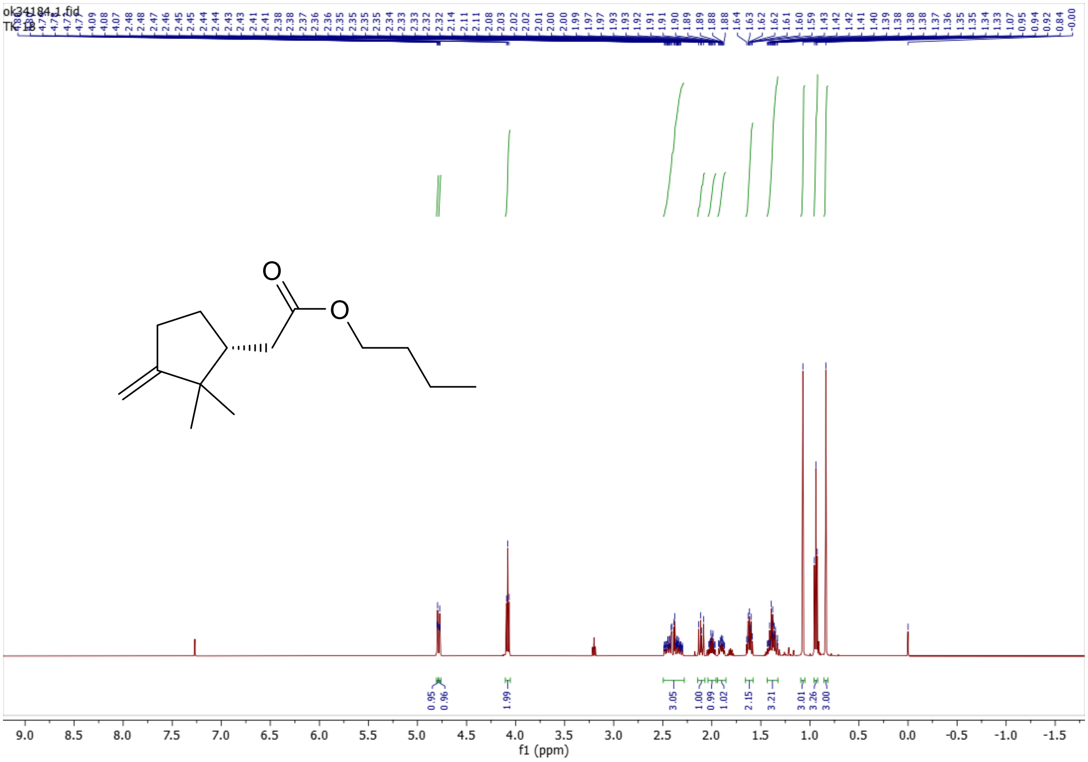

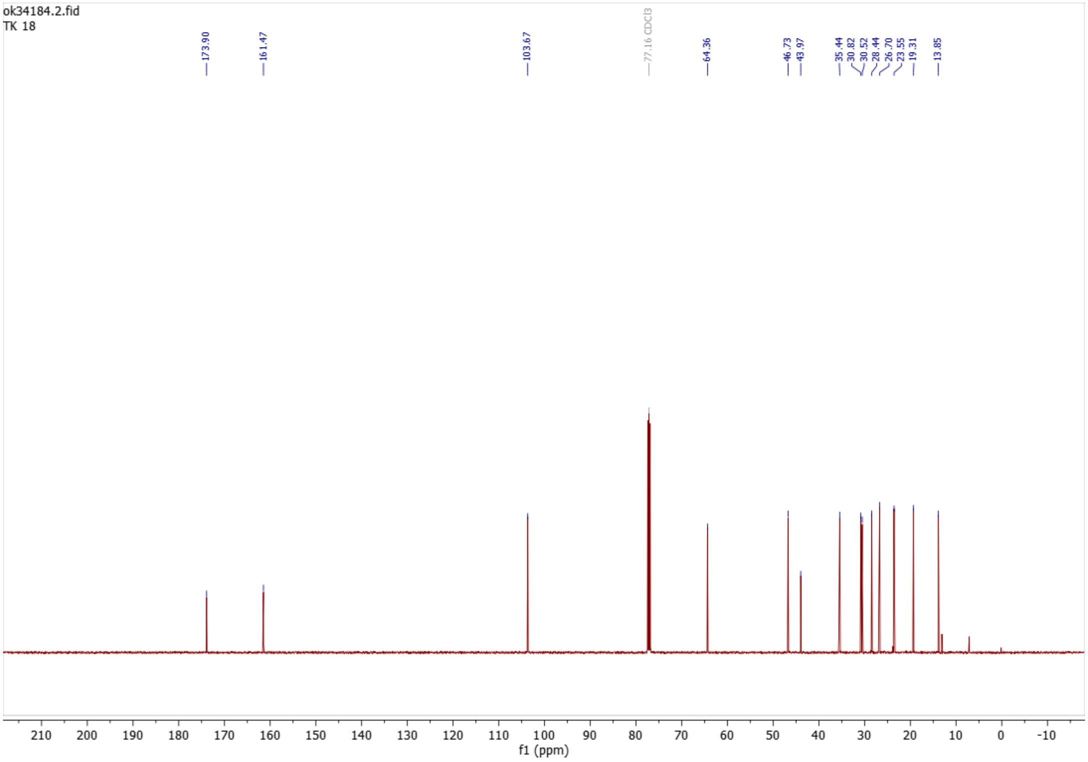

Isopropyl (*R*)-2-(2,2-dimethyl-3-methylenecyclopentyl)acetate (6e)

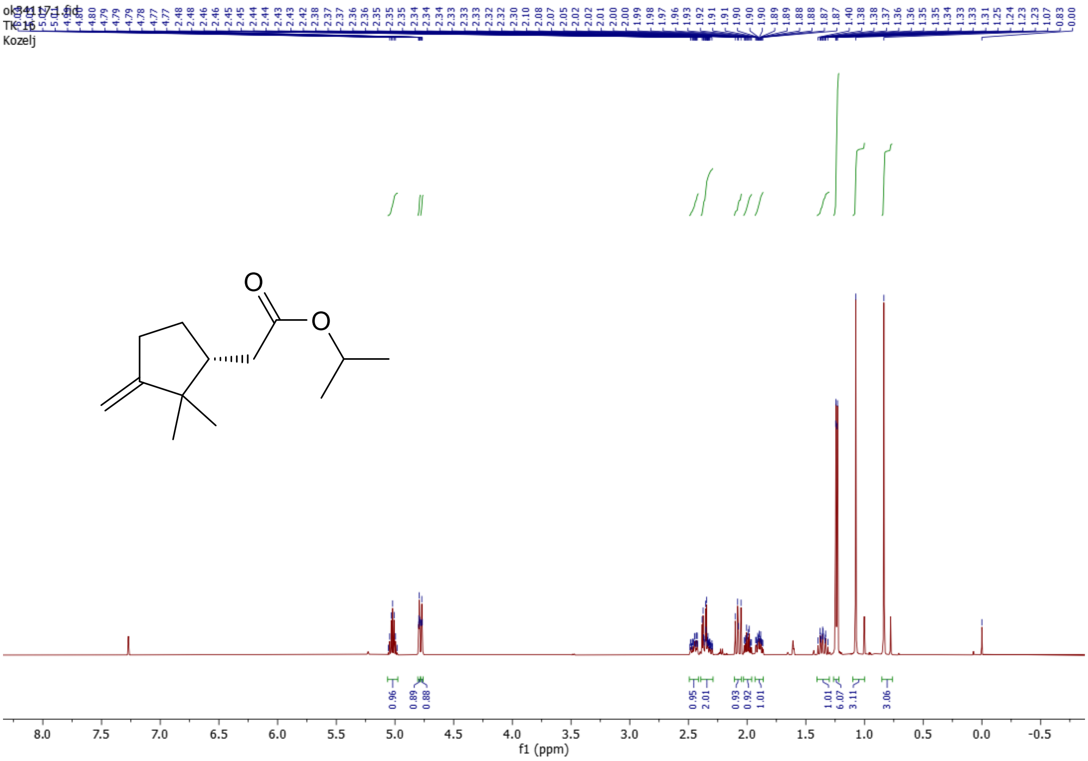

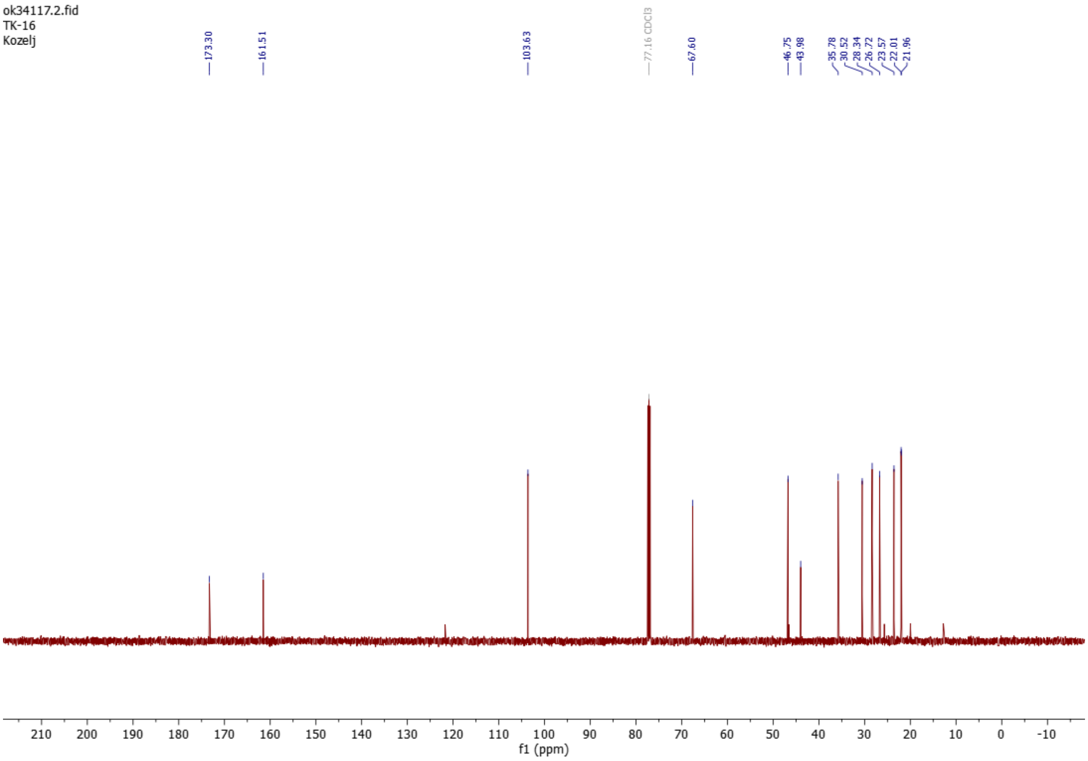

Isobutyl (*R*)-2-(2,2-dimethyl-3-methylenecyclopentyl)acetate (6f)

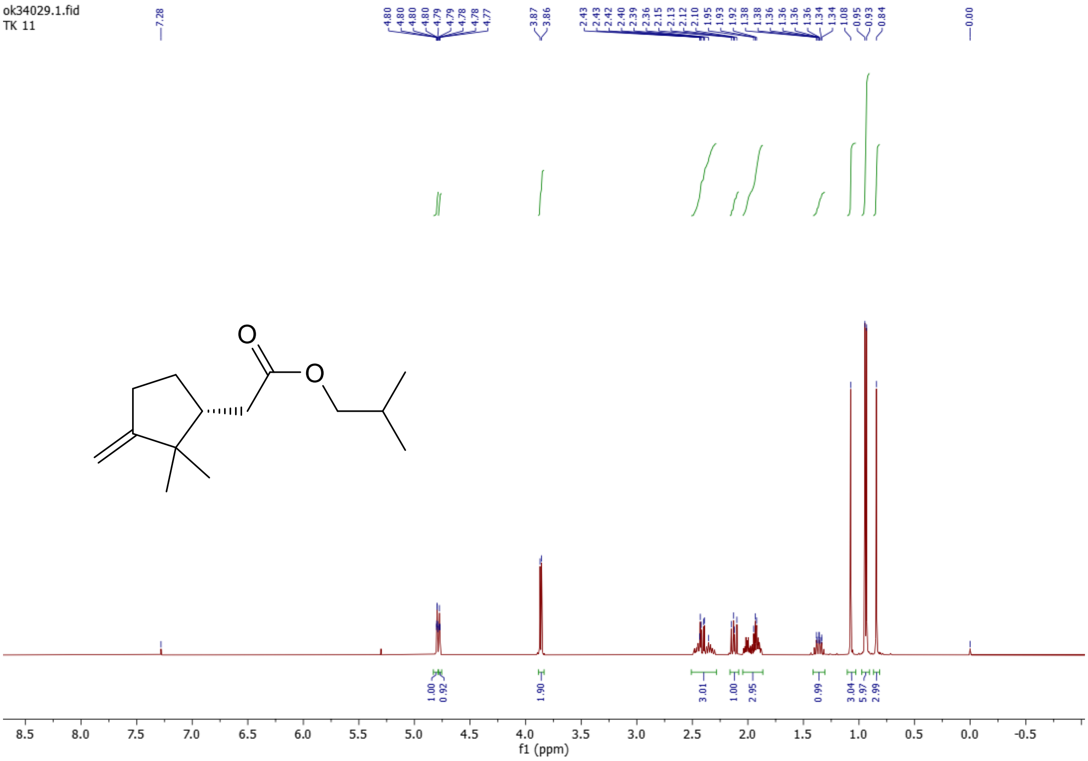

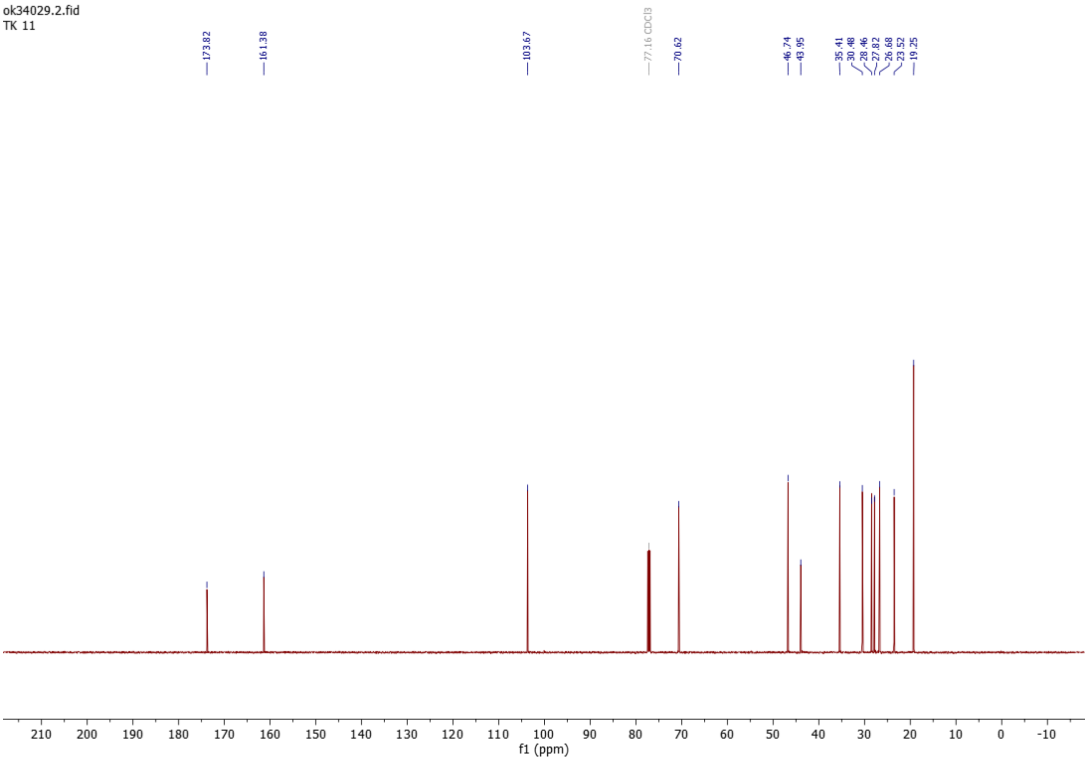

Allyl (*R*)-2-(2,2-dimethyl-3-methylenecyclopentyl)acetate (6g)

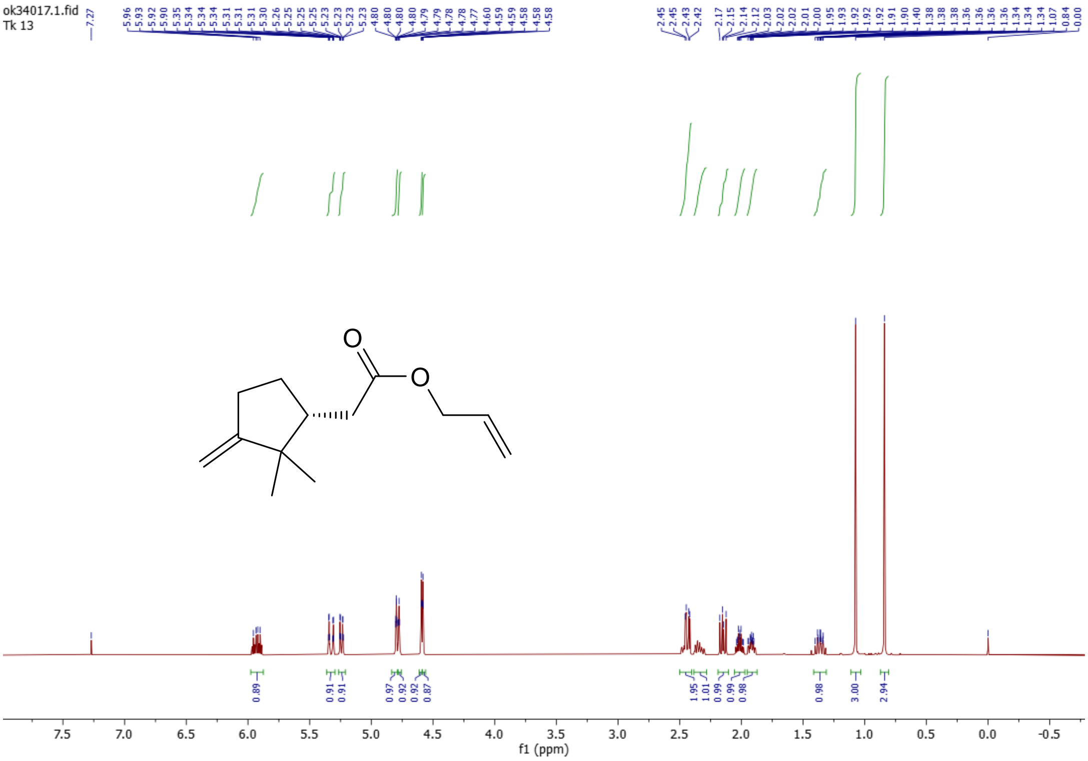

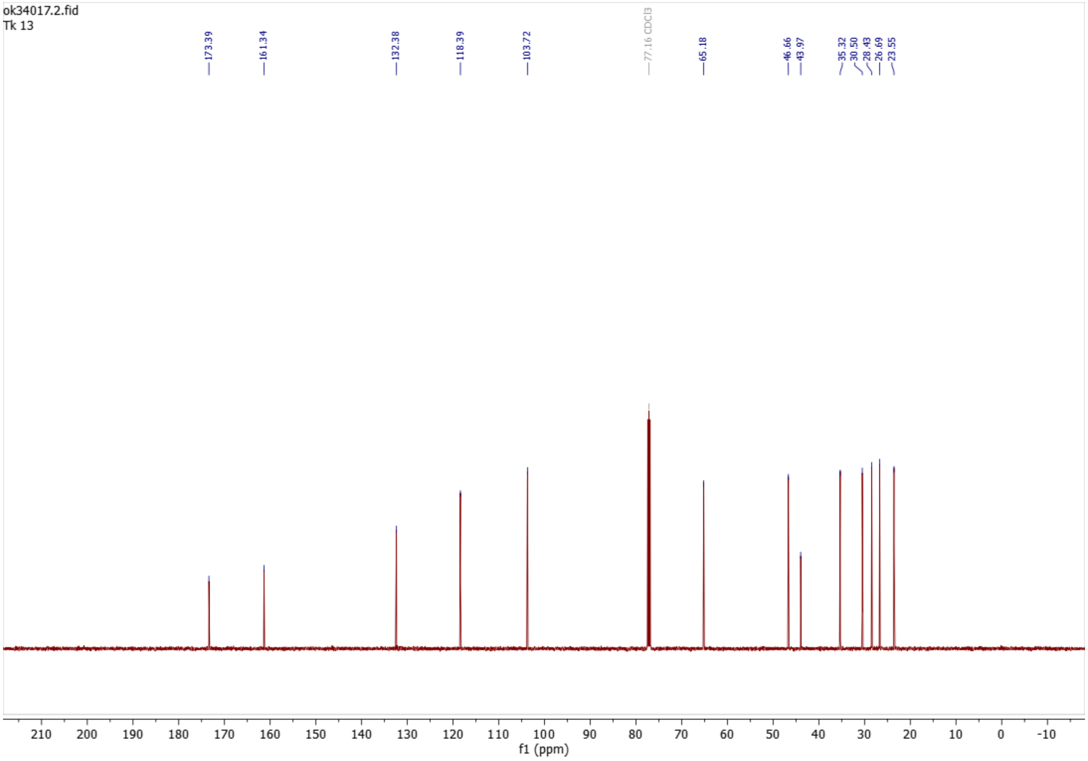

Benzyl (*R*)-2-(2,2-dimethyl-3-methylenecyclopentyl)acetate (6h)

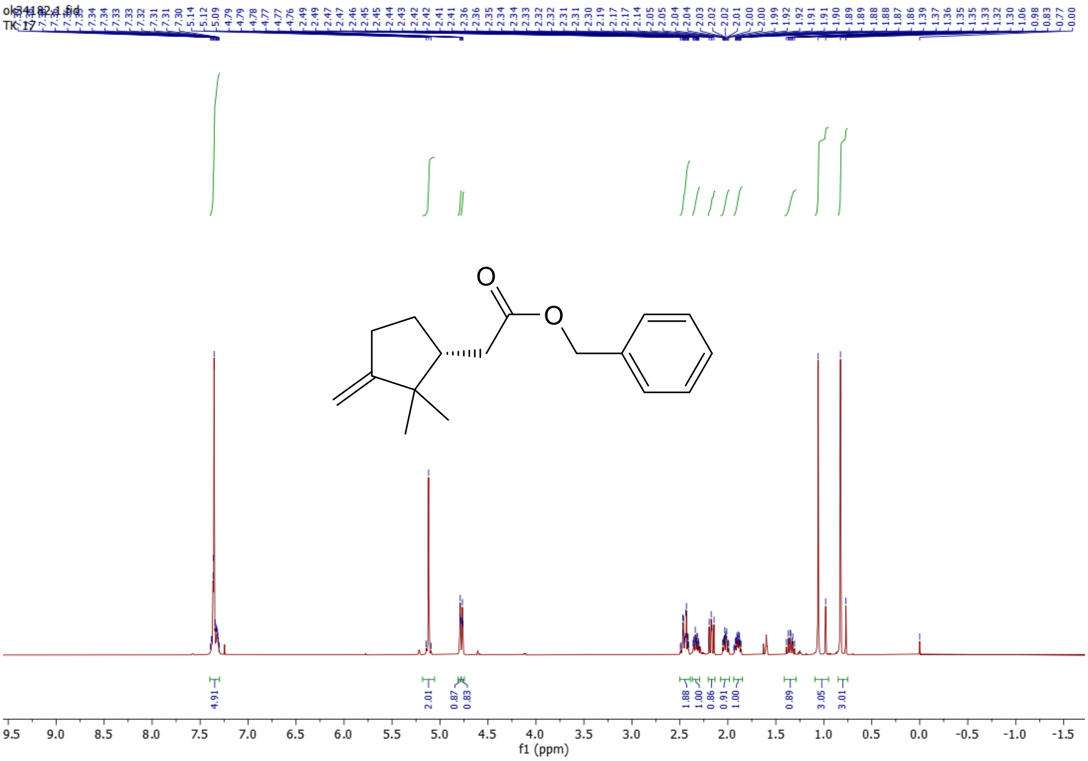

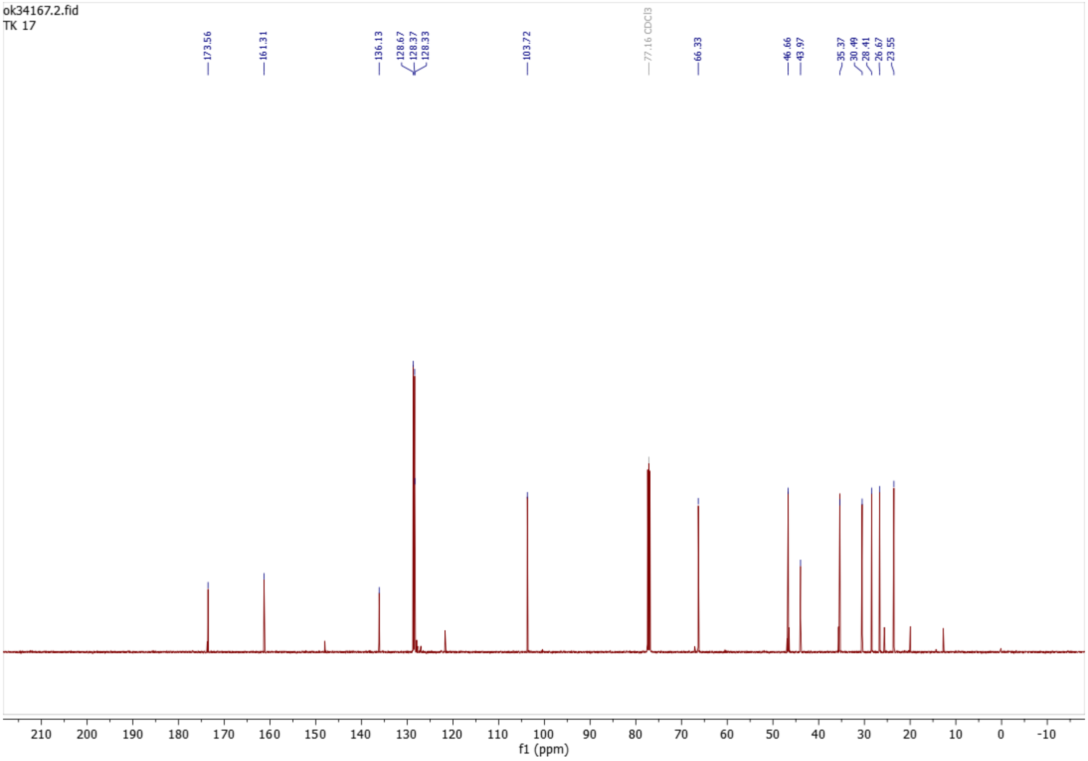

Dodecyl (*R*)-2-(2,2-dimethyl-3-methylenecyclopentyl)acetate (6i)

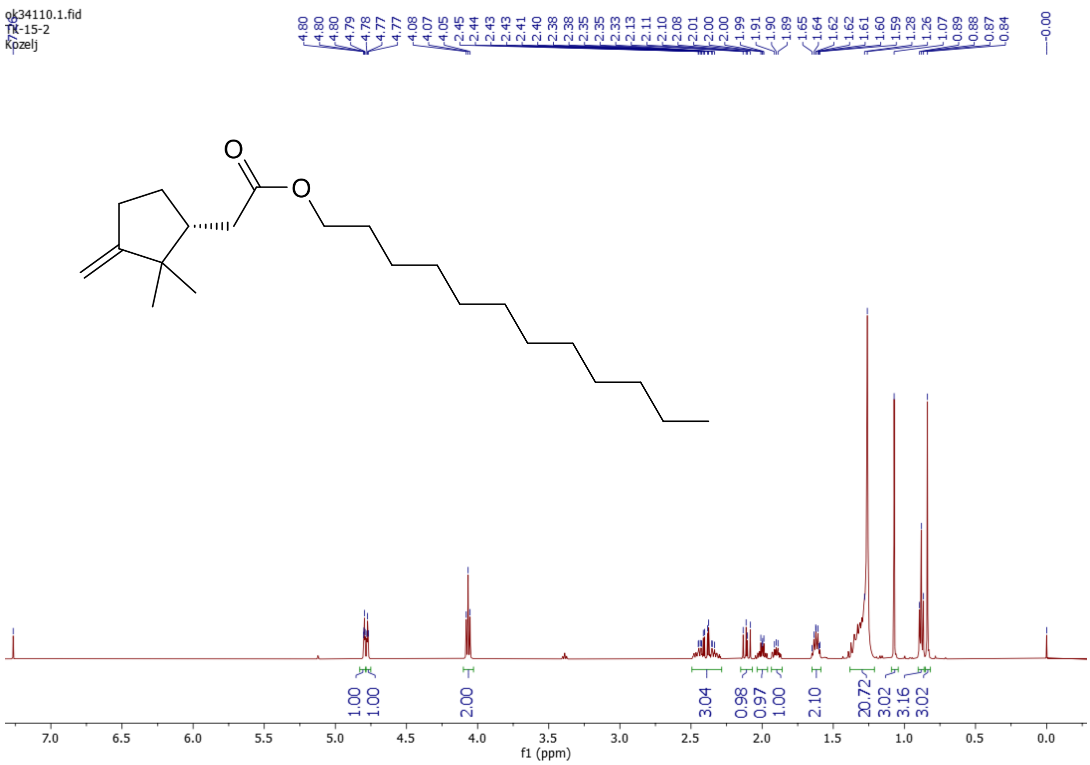

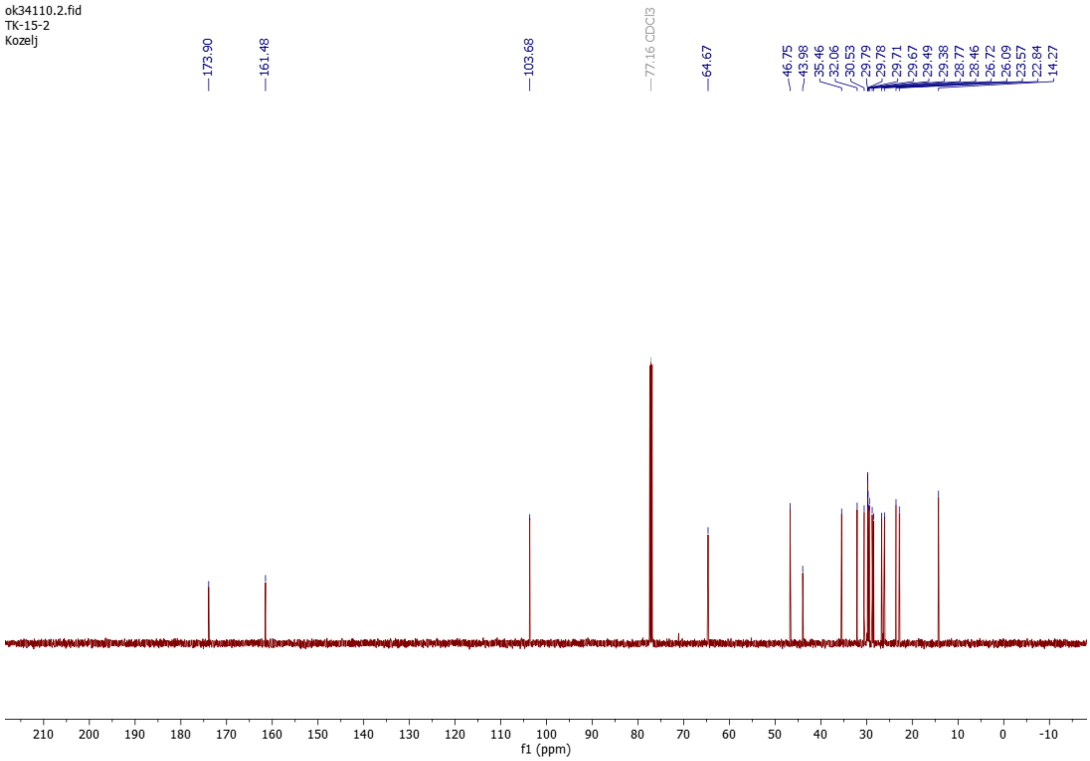

(*R*)-3,7-Dimethyloct-6-en-1-yl 2-((*R*)-2,2-dimethyl-3-methylenecyclopentyl)acetate (6j)

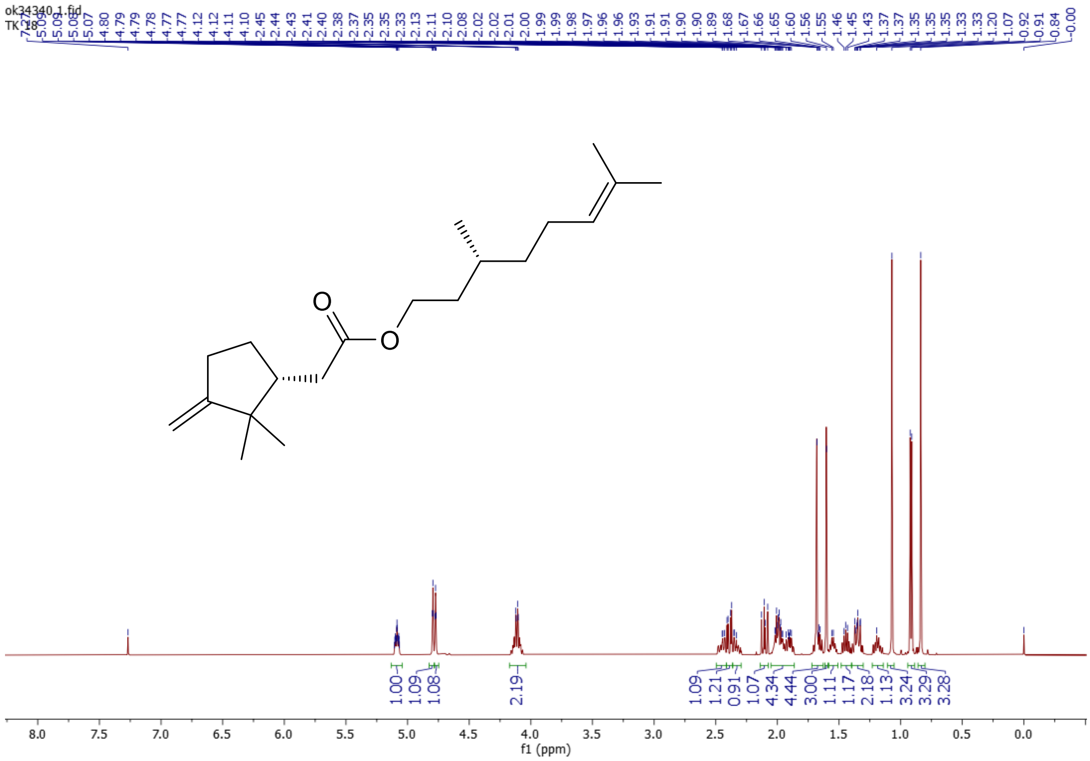

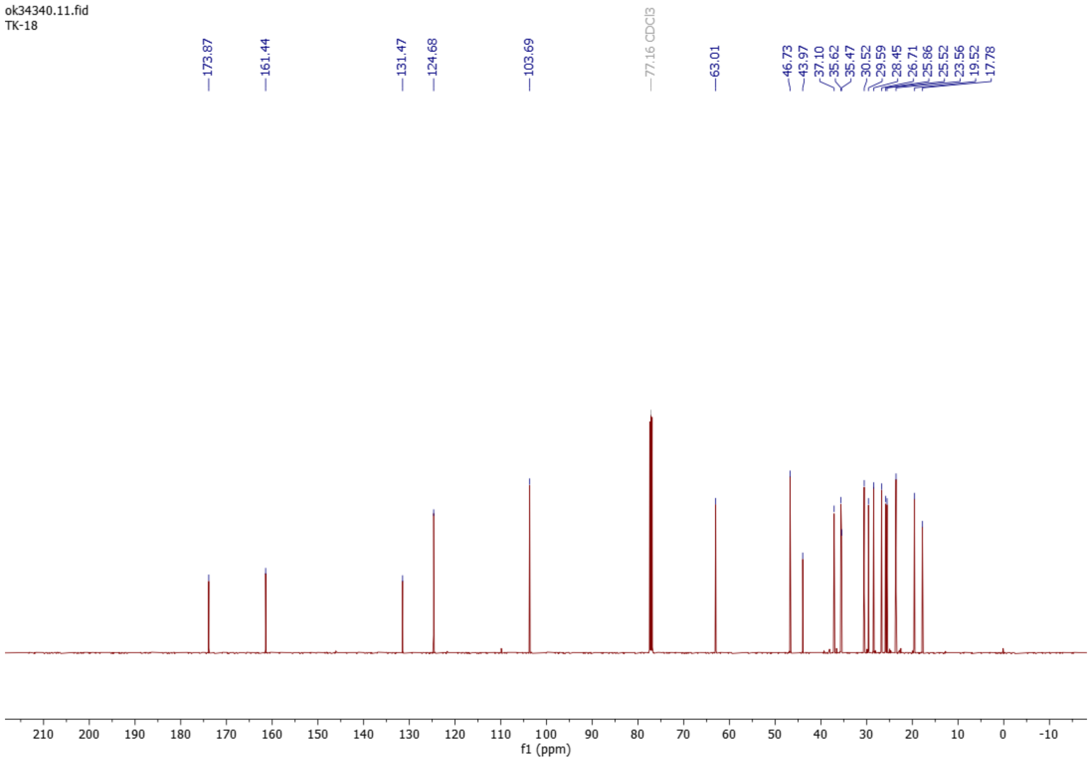

(*R*)-2-(2,2-Dimethyl-3-methylenecyclopentyl)ethyl acetate (7a)

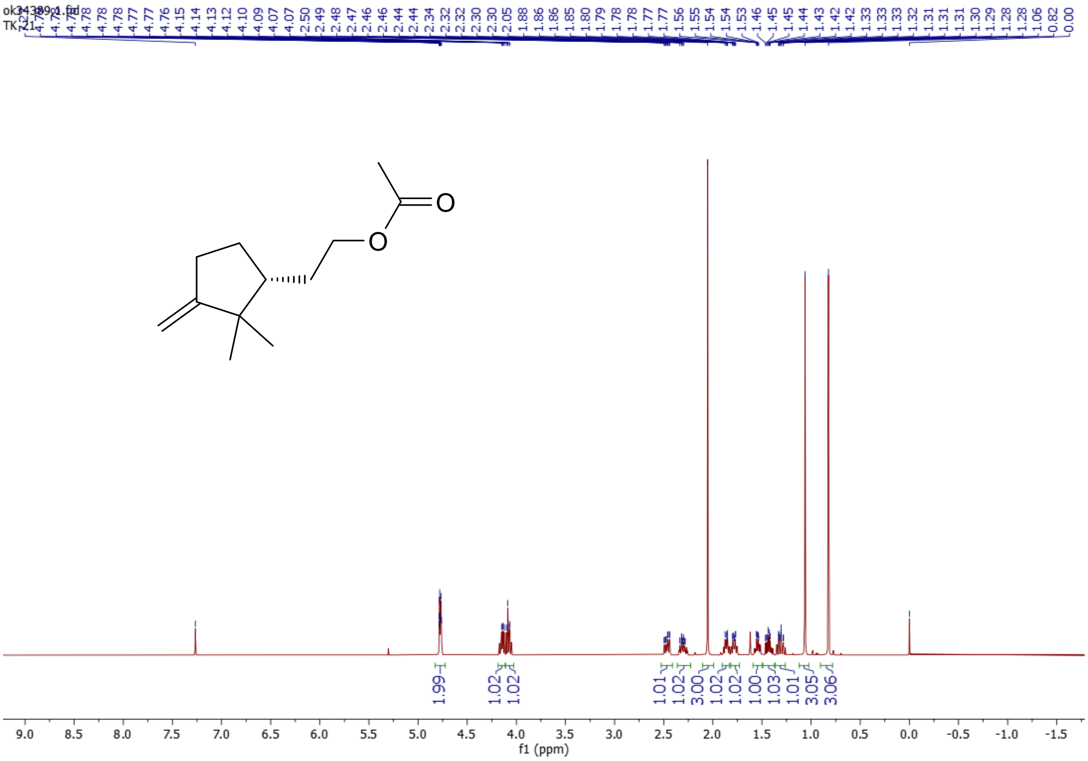

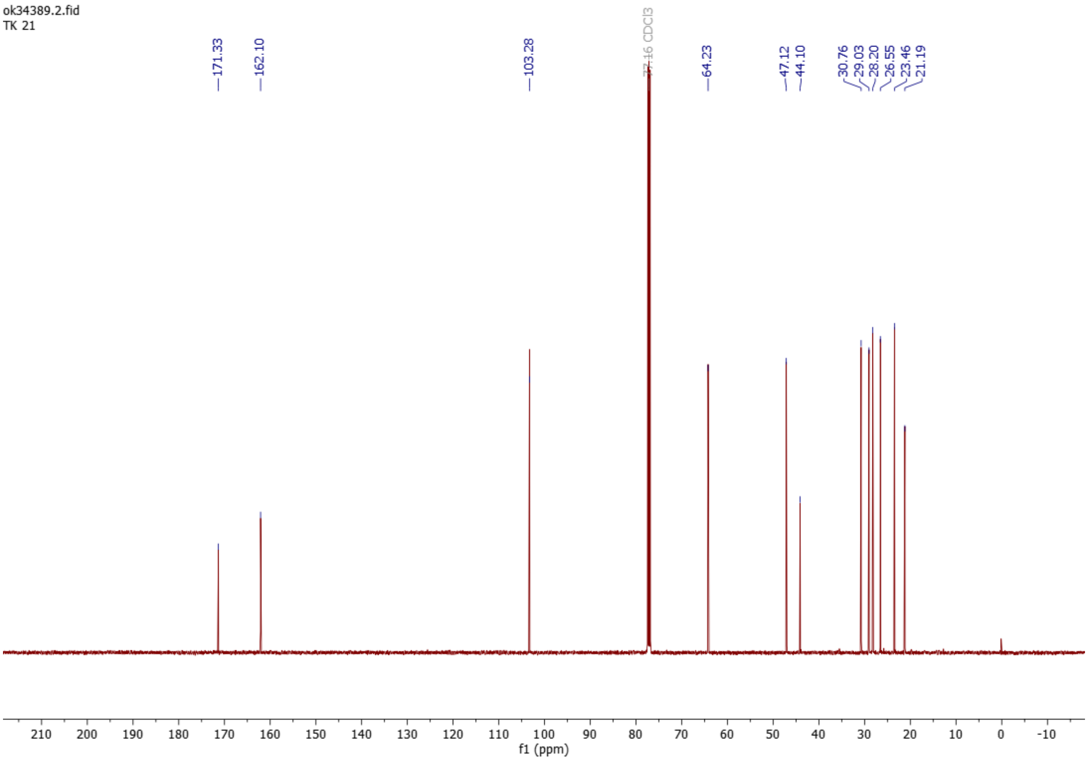

(*R*)-2-(2,2-Dimethyl-3-methylenecyclopentyl)ethyl propionate (7b)

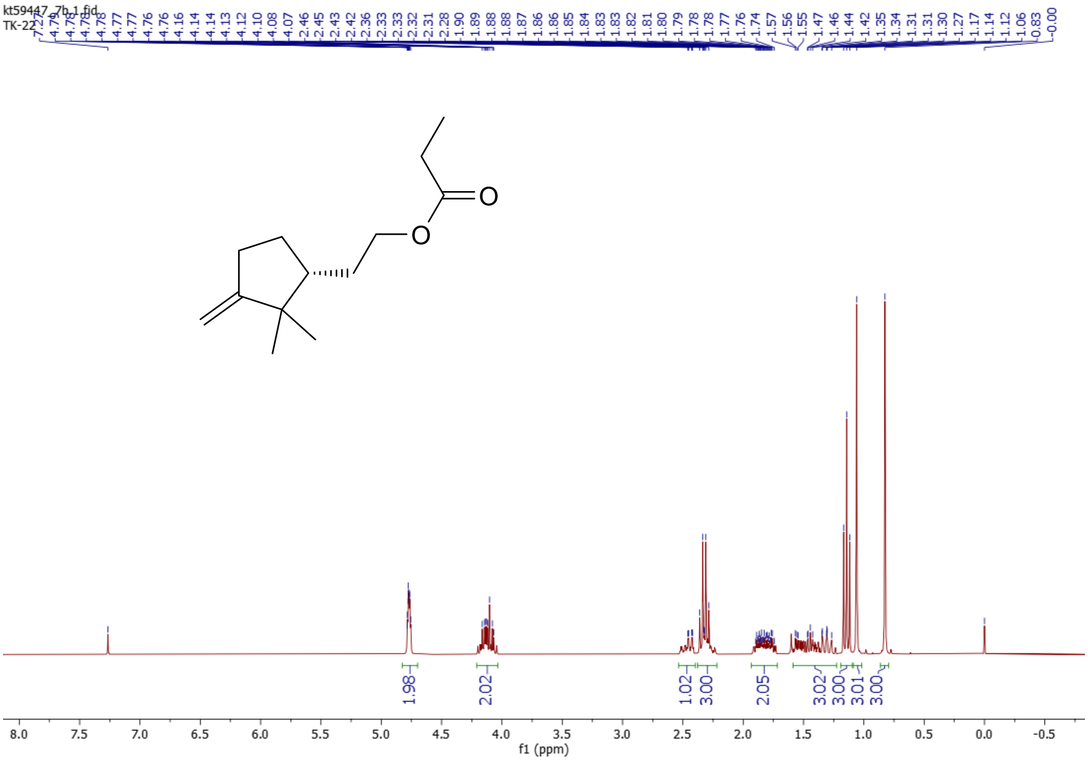

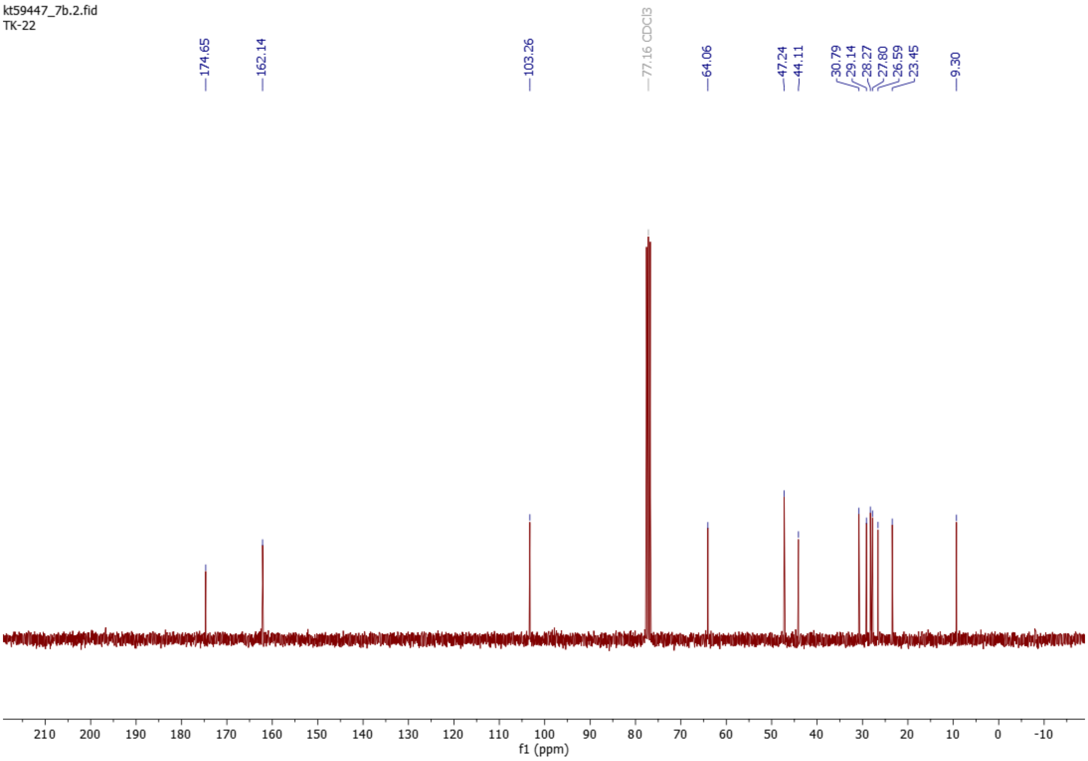

(*R*)-2-(2,2-Dimethyl-3-methylenecyclopentyl)ethyl butyrate (7c)

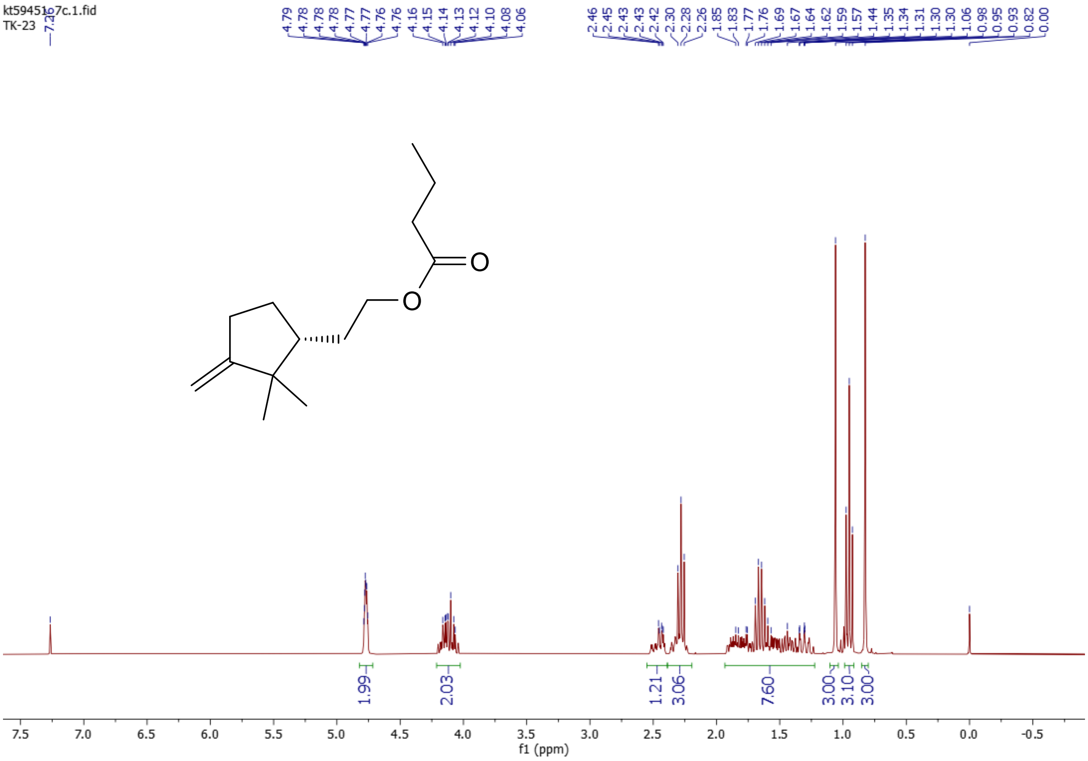

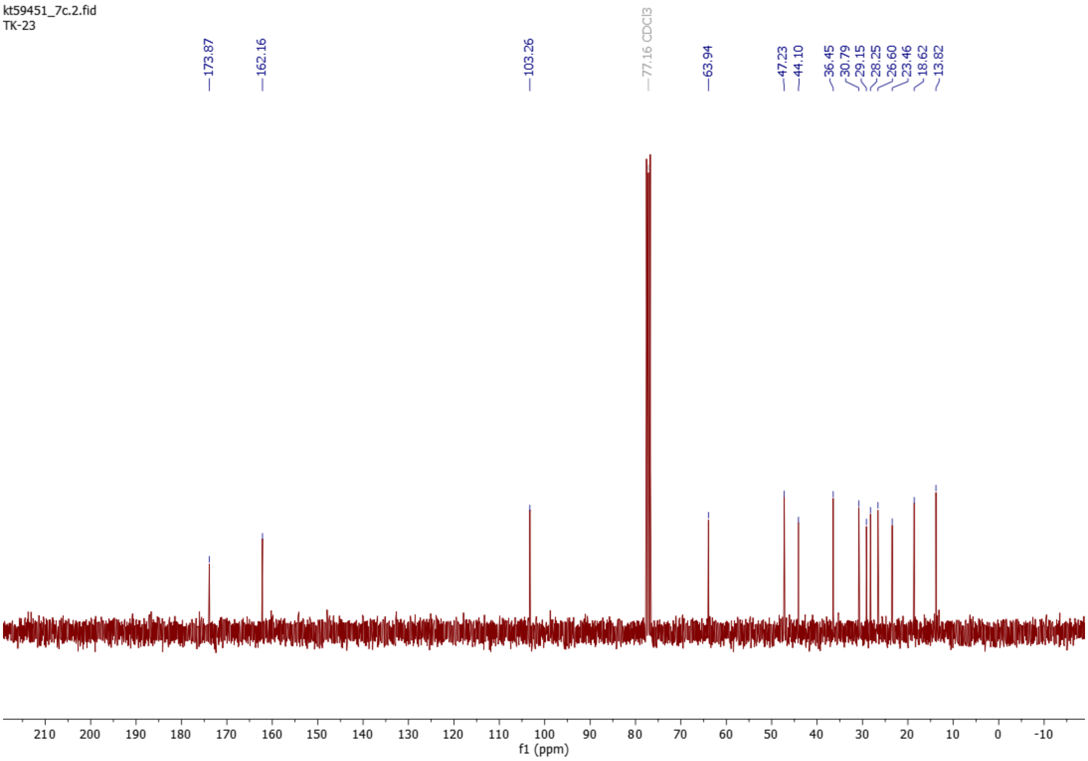

(*R*)-2-(2,2-Dimethyl-3-methylenecyclopentyl)ethyl isobutyrate (7d)

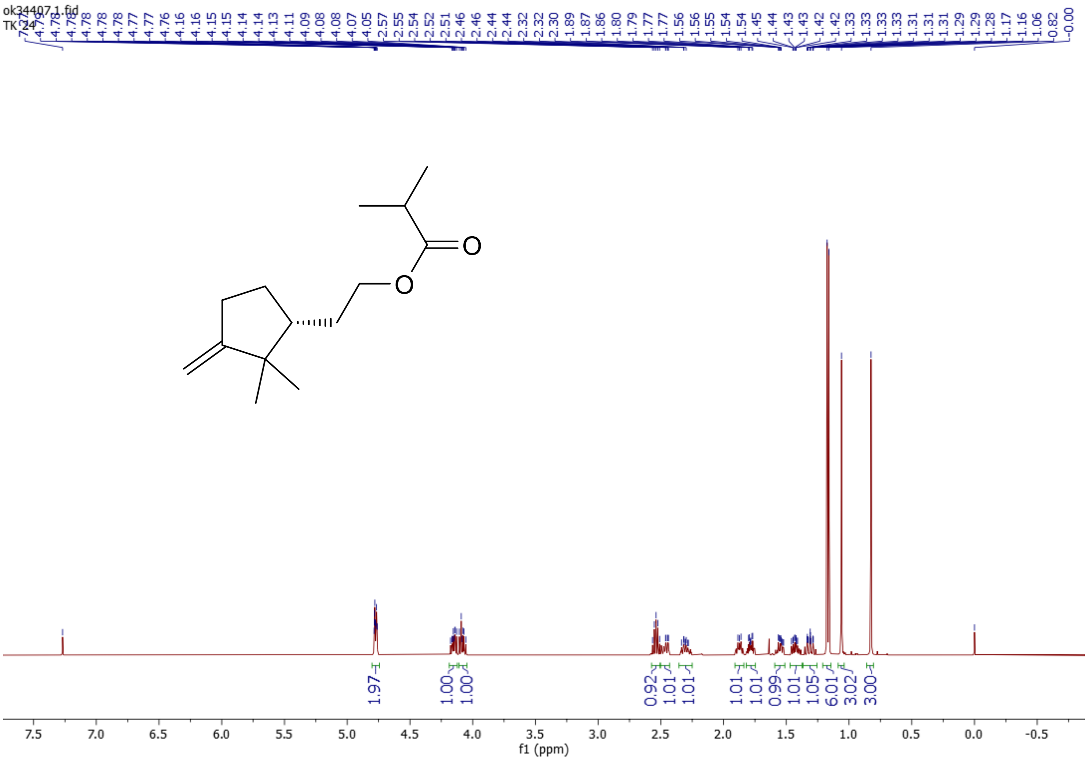

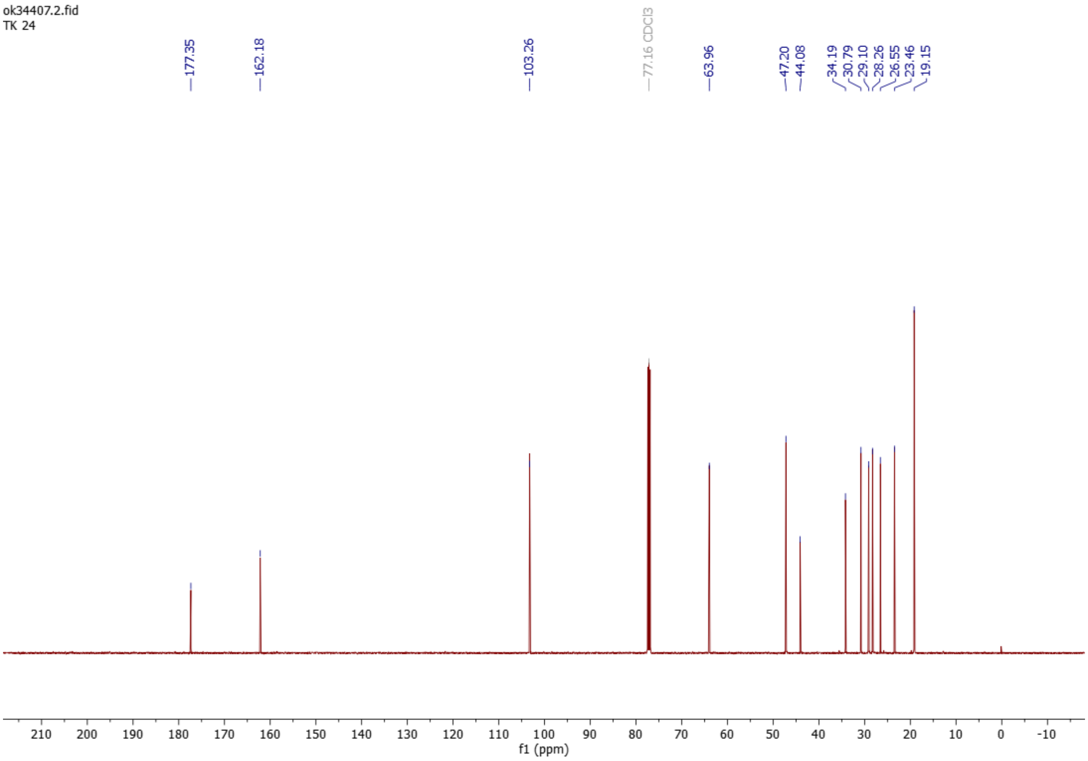

(*R*)-2-(2,2-Dimethyl-3-methylenecyclopentyl)ethyl 3,3-dimethylbutanoate (7e)

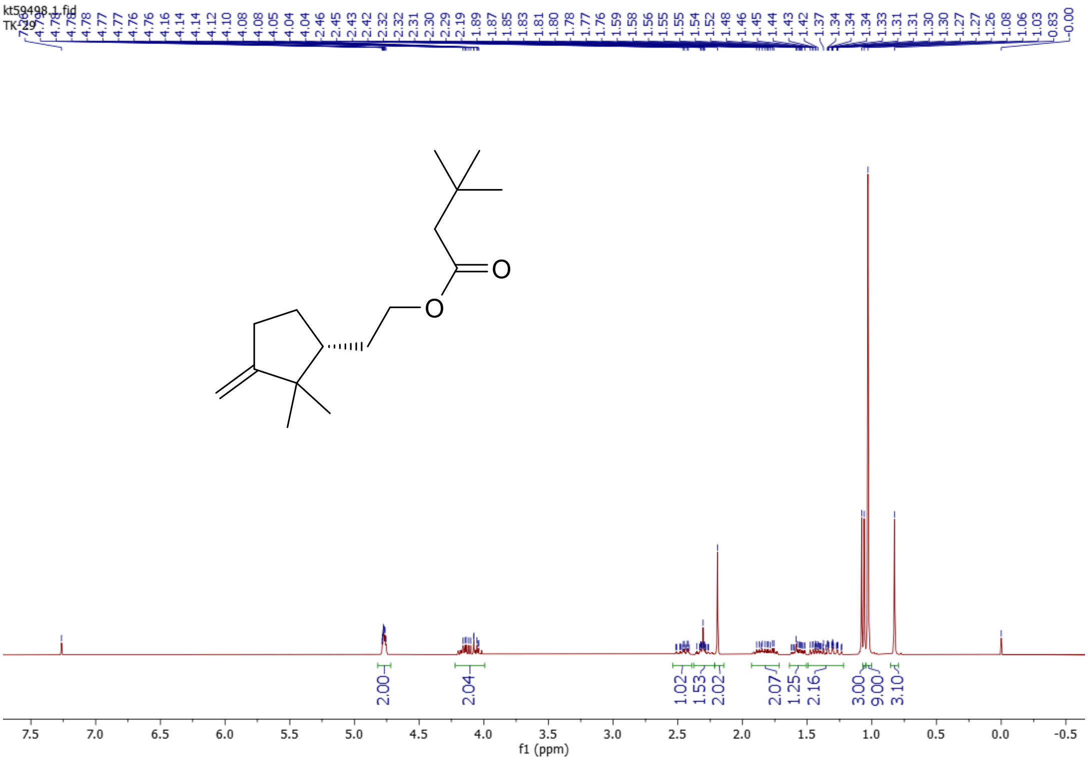

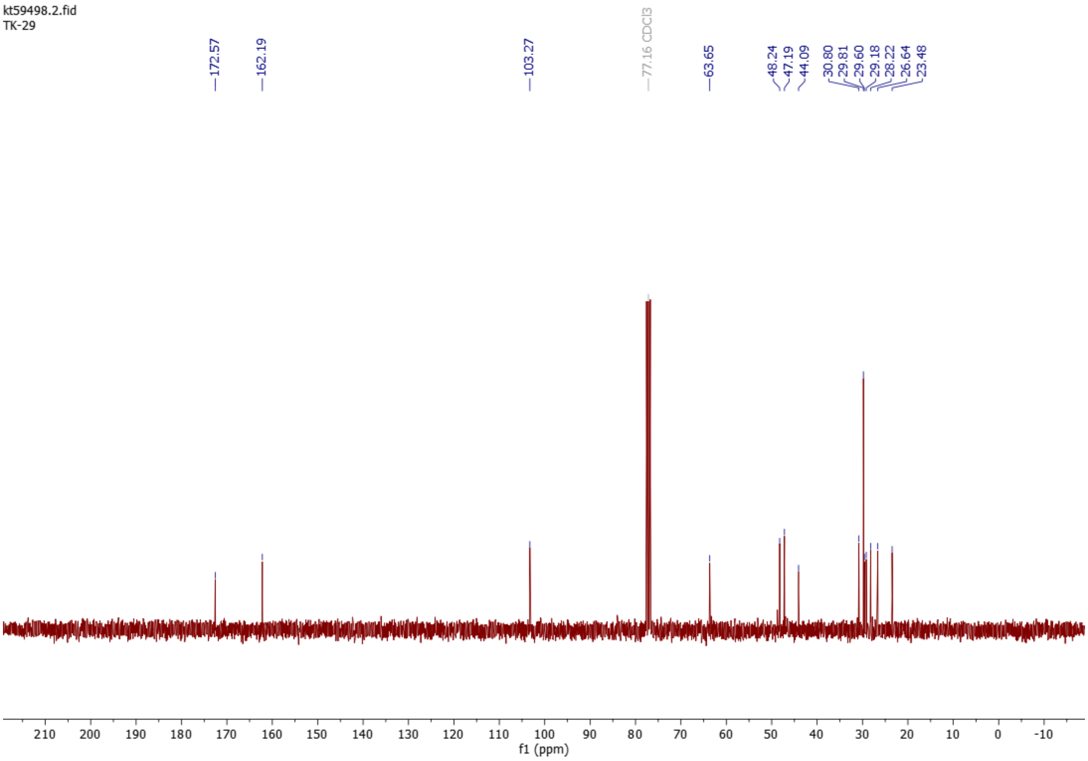

(*R*)-2-(2,2-Dimethyl-3-methylenecyclopentyl)ethyl pent-4-enoate (7f)

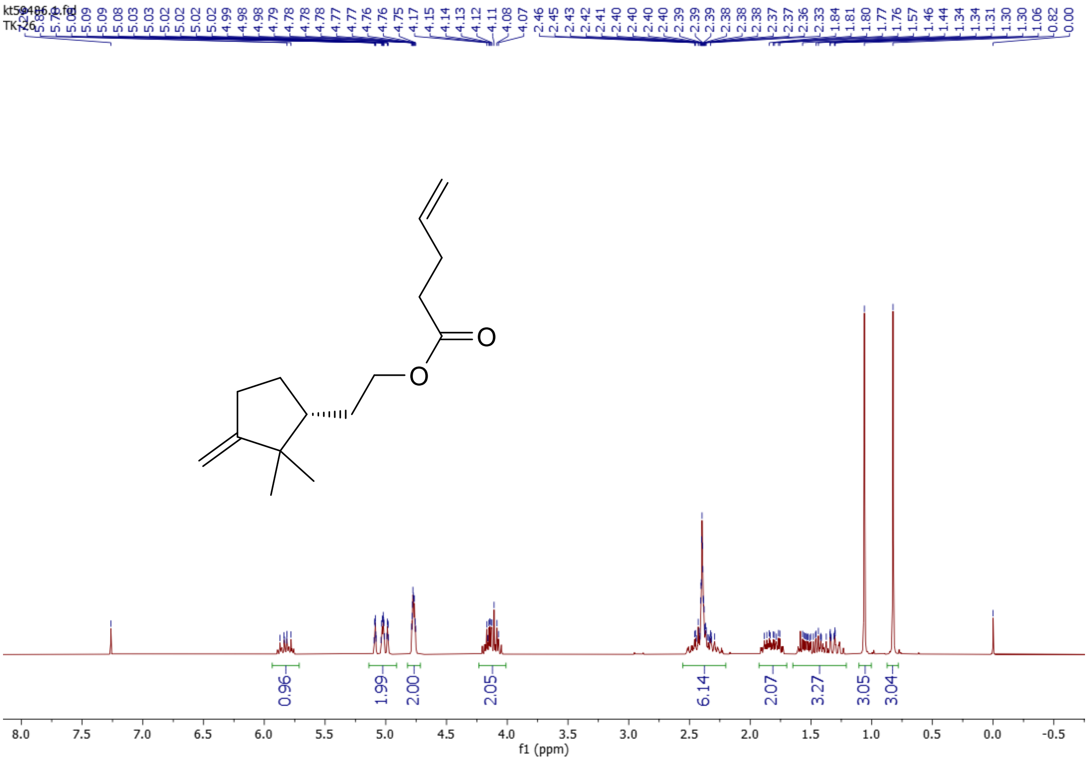

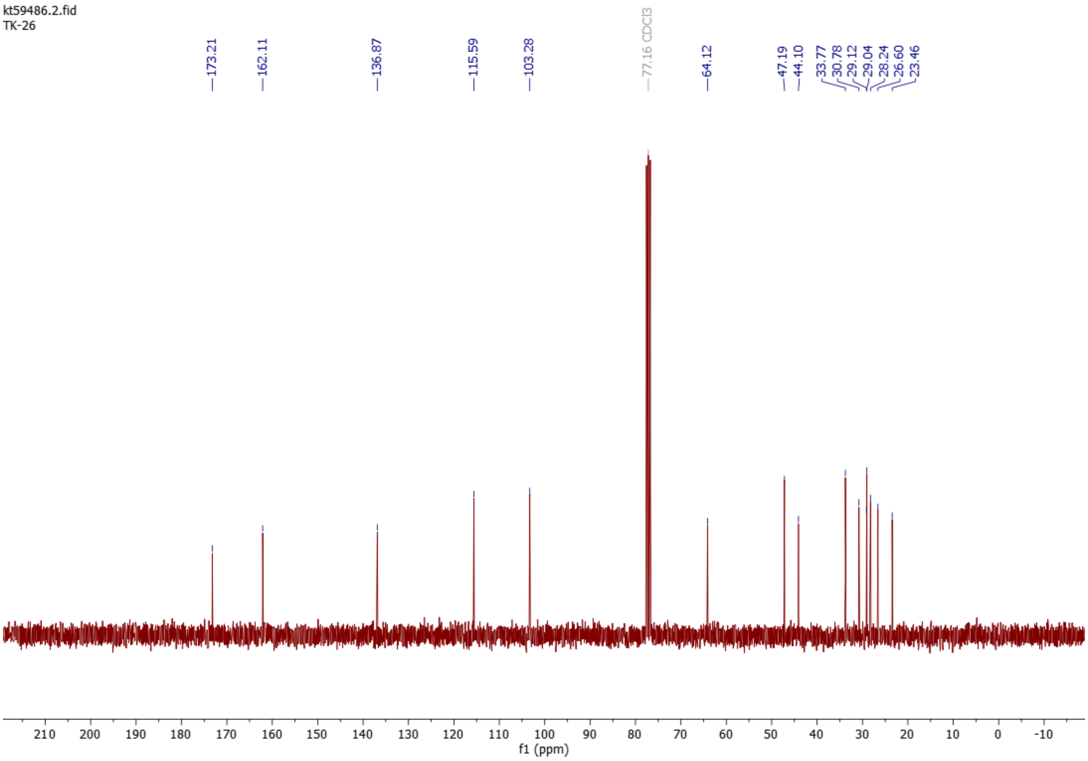

(*R*)-2-(2,2-Dimethyl-3-methylenecyclopentyl)ethyl benzoate (7g)

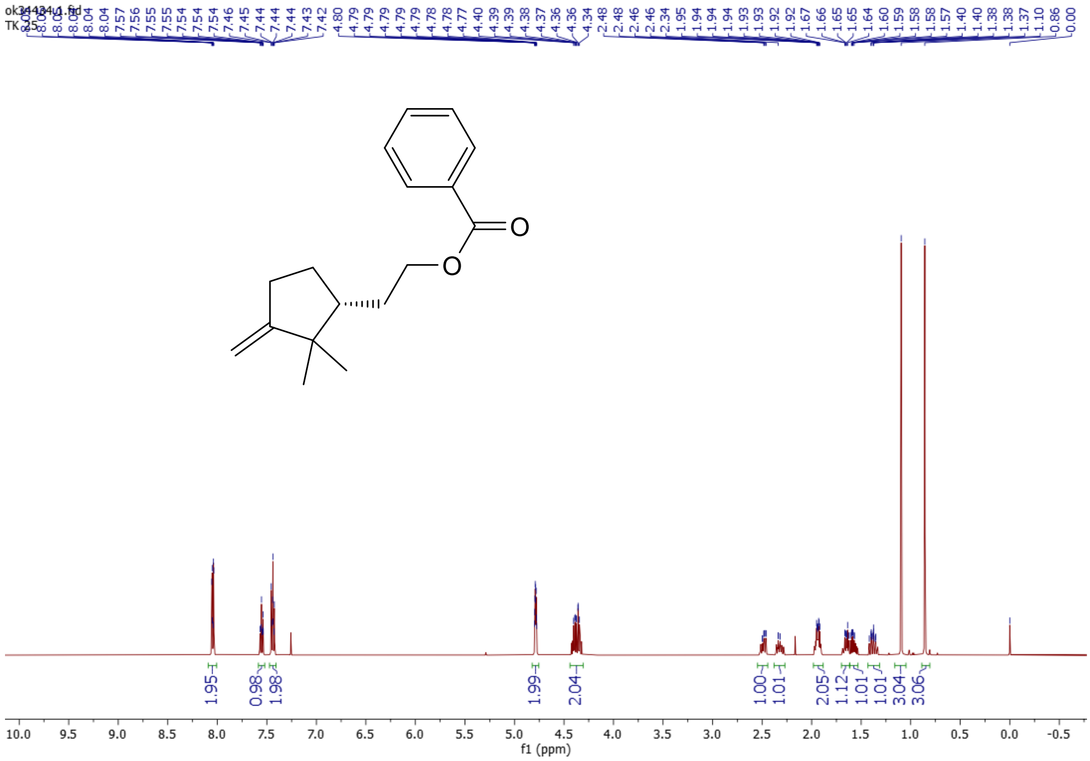

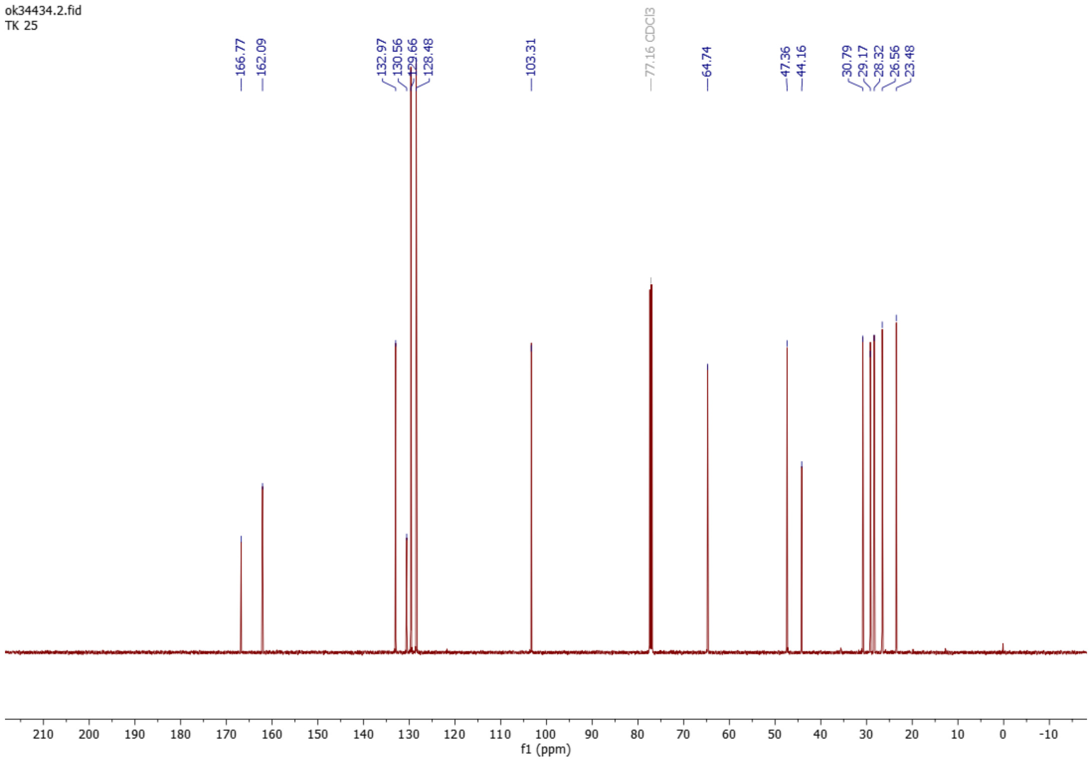

(*R*)-4-(2-(2,2-Dimethyl-3-methylenecyclopentyl)ethoxy)-4-oxobutanoic acid (7h)

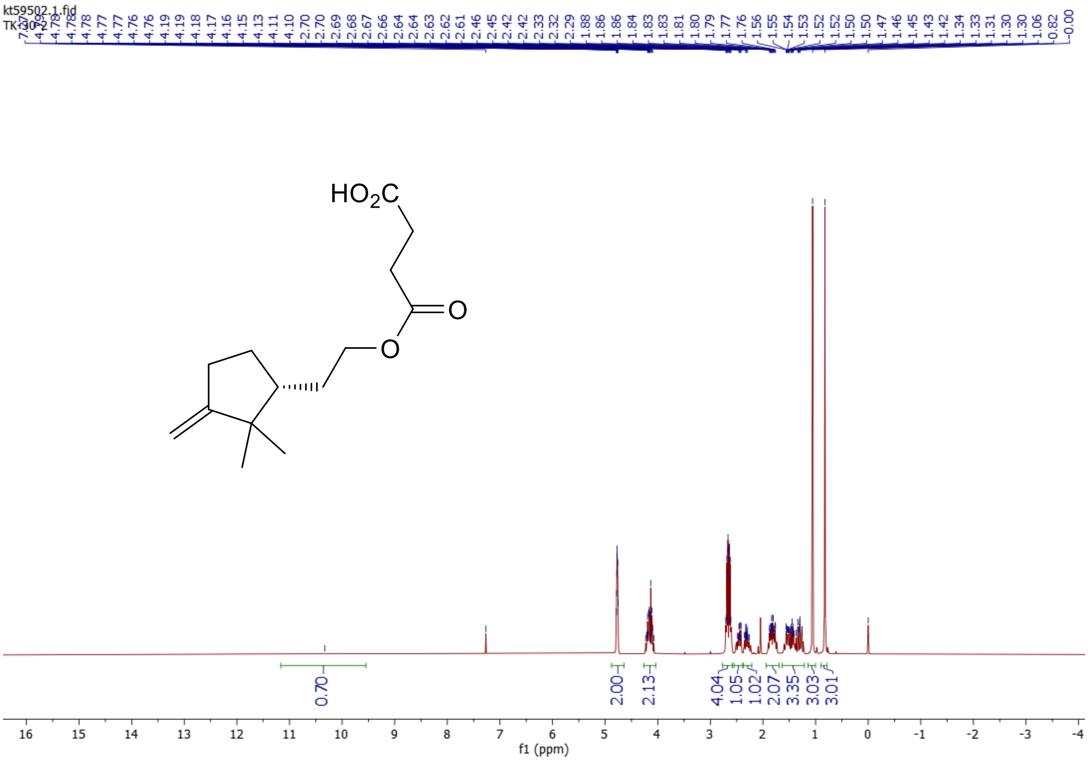

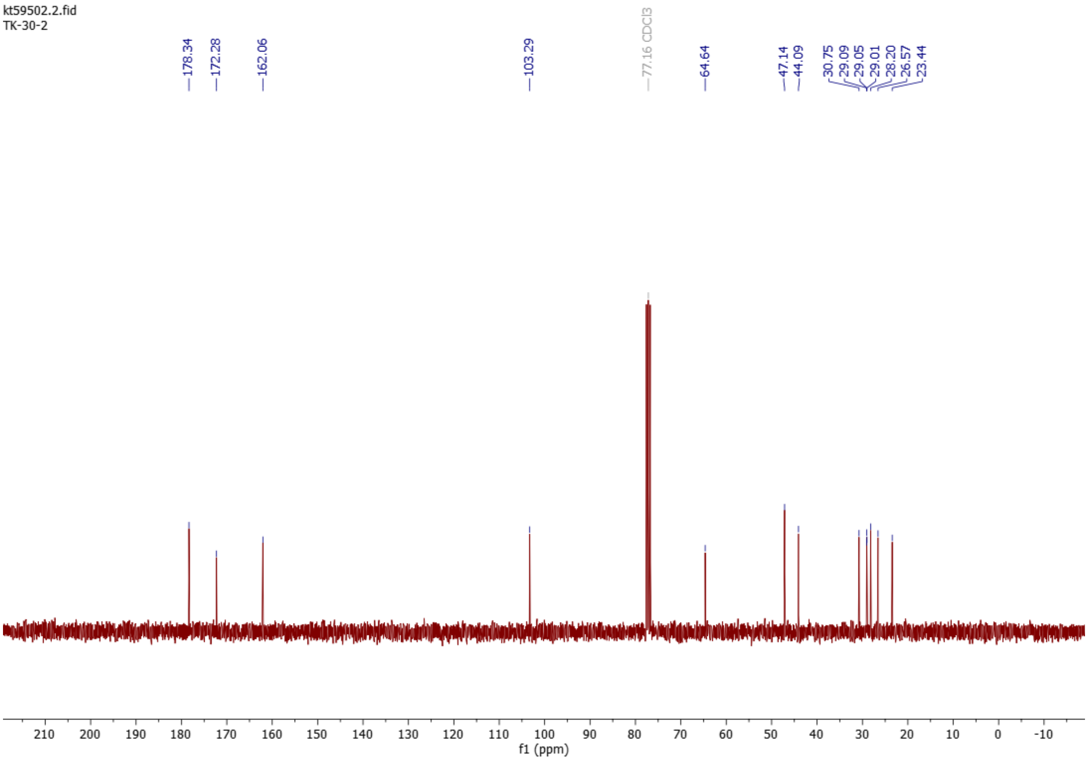

(*R*)-2-(2,2-Dimethyl-3-methylenecyclopentyl)ethyl methyl succinate (7i)

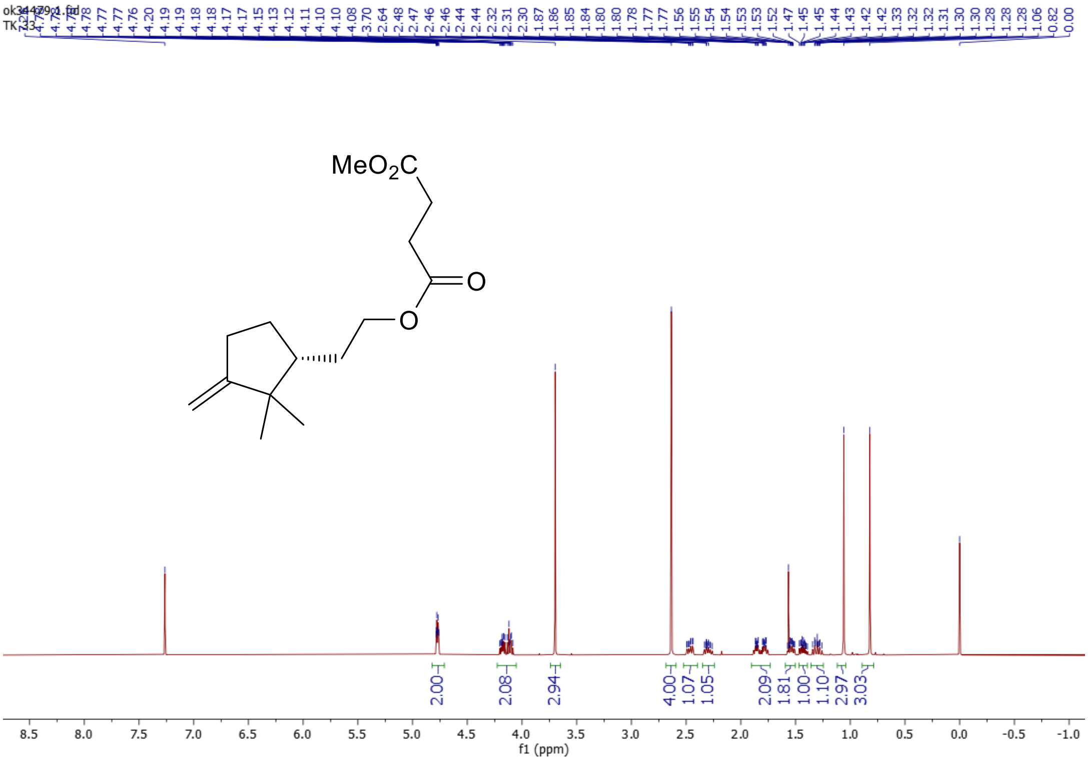

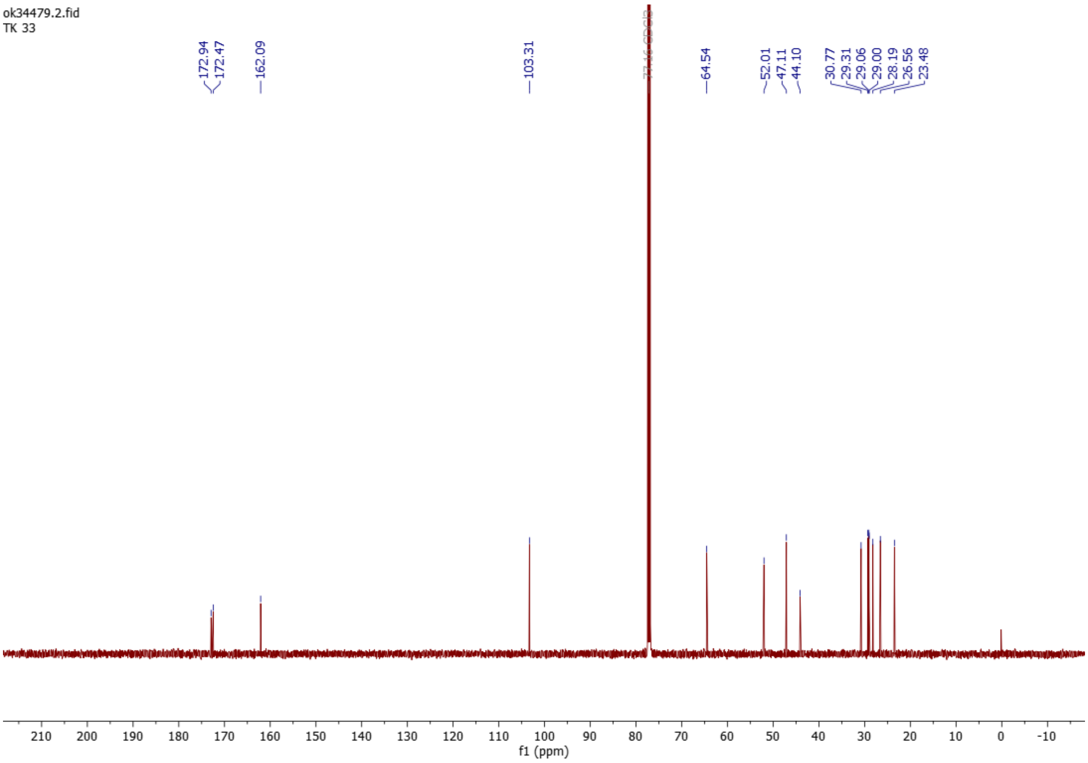

(*R*)-2-(2,2-Dimethyl-3-methylenecyclopentyl)ethyl ethyl succinate (7j)

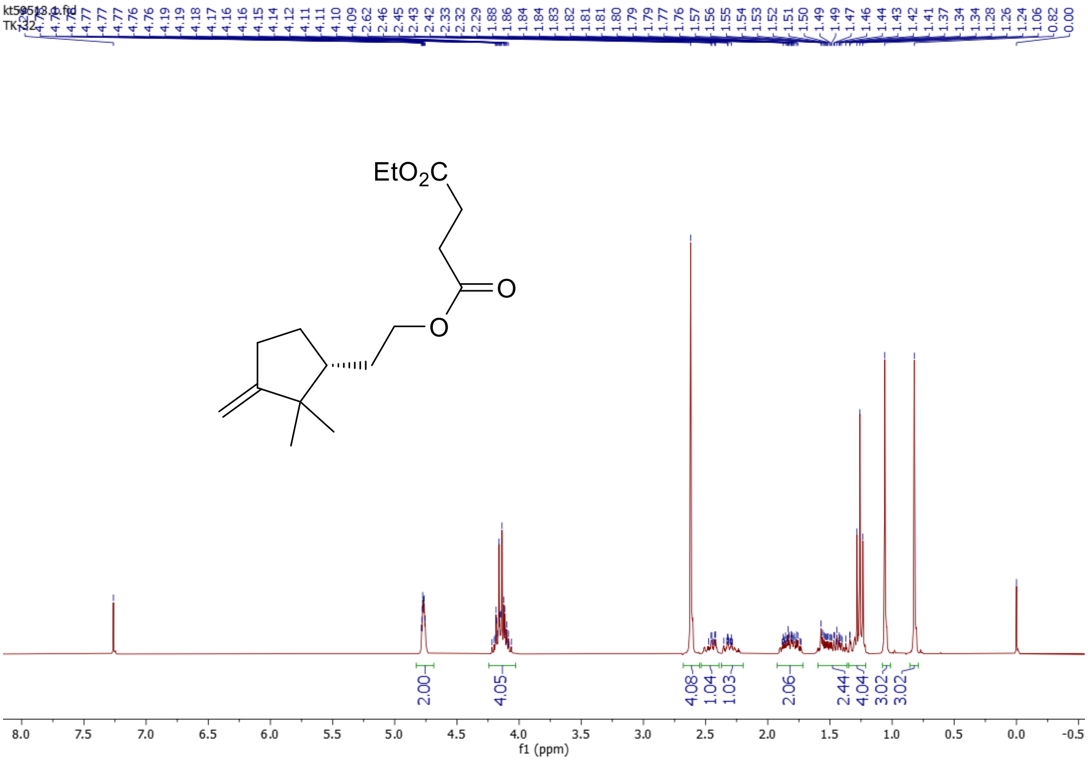

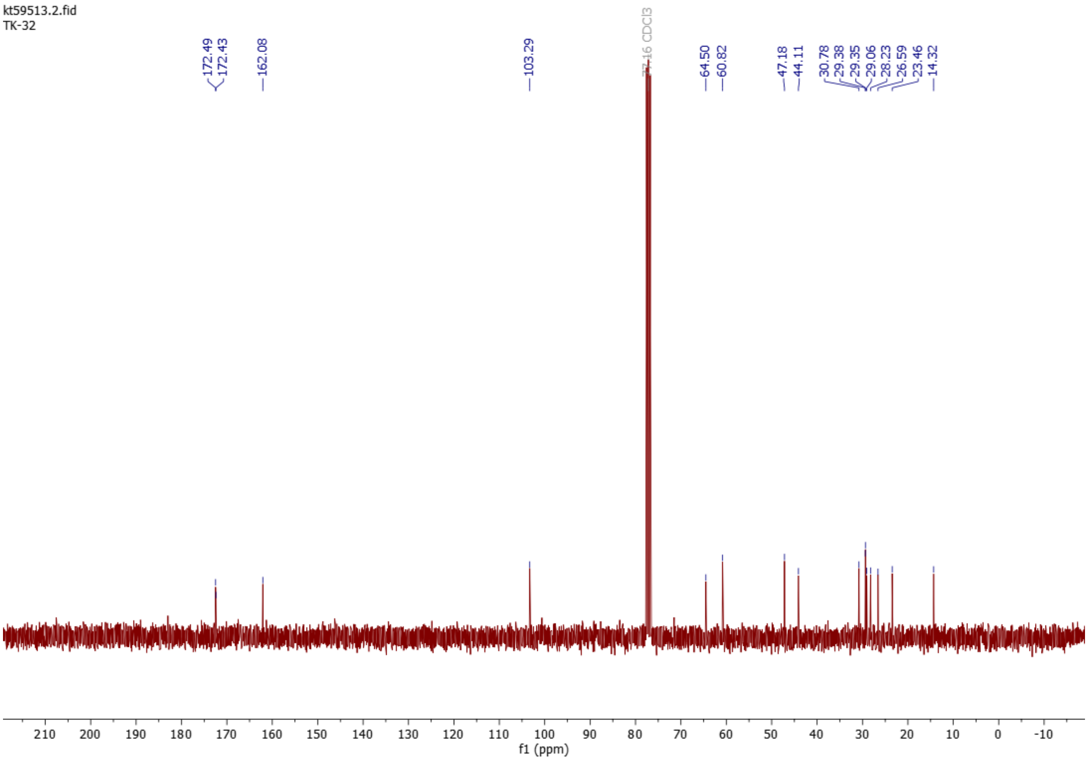

(*R*)-2-(2-Methoxyethyl)-1,1-dimethyl-5-methylenecyclopentane (8a)

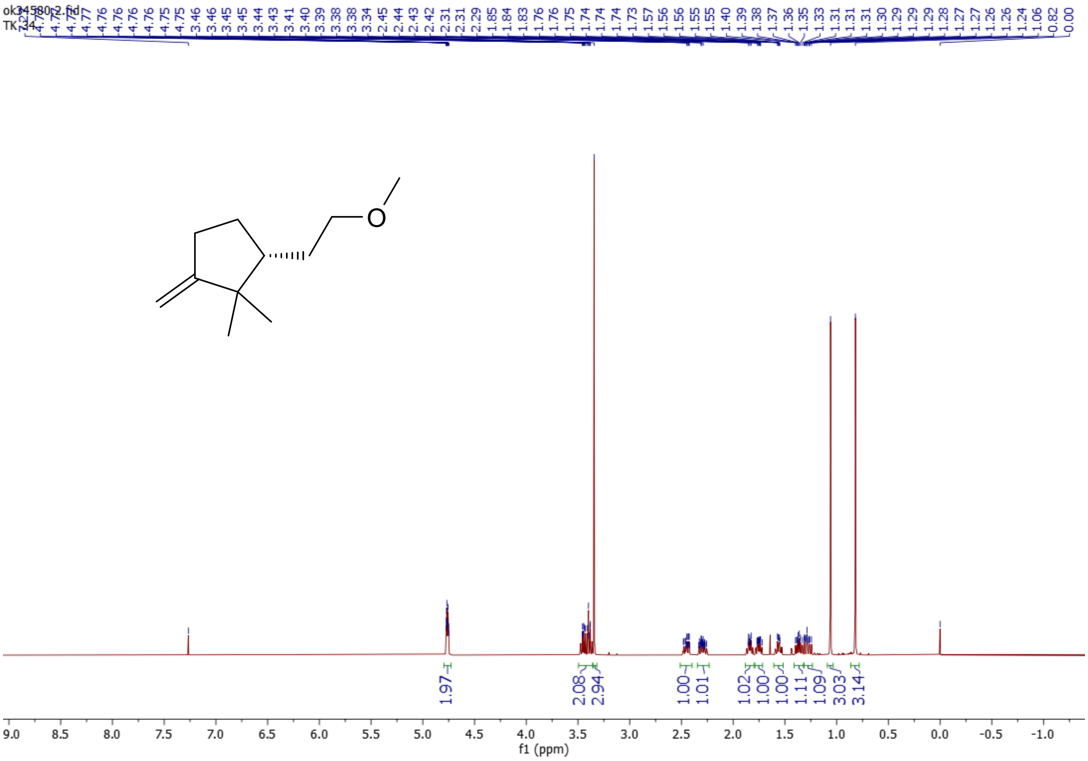

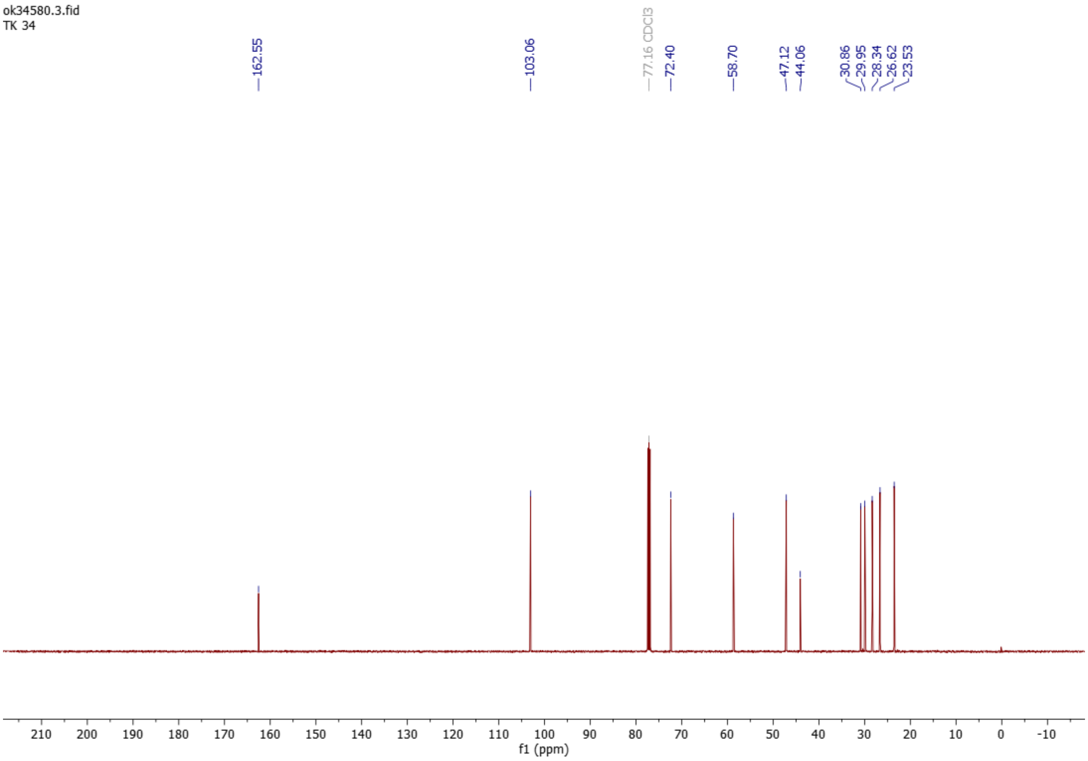

(*R*)-2-(2-Ethoxyethyl)-1,1-dimethyl-5-methylenecyclopentane (8b)

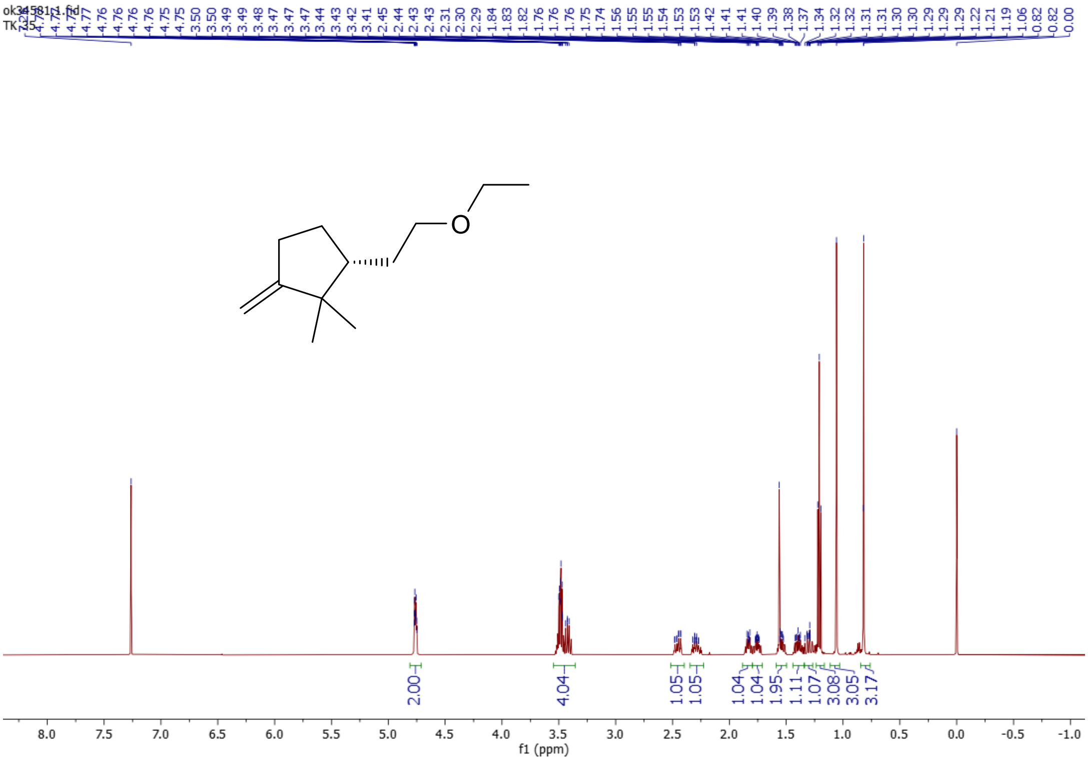

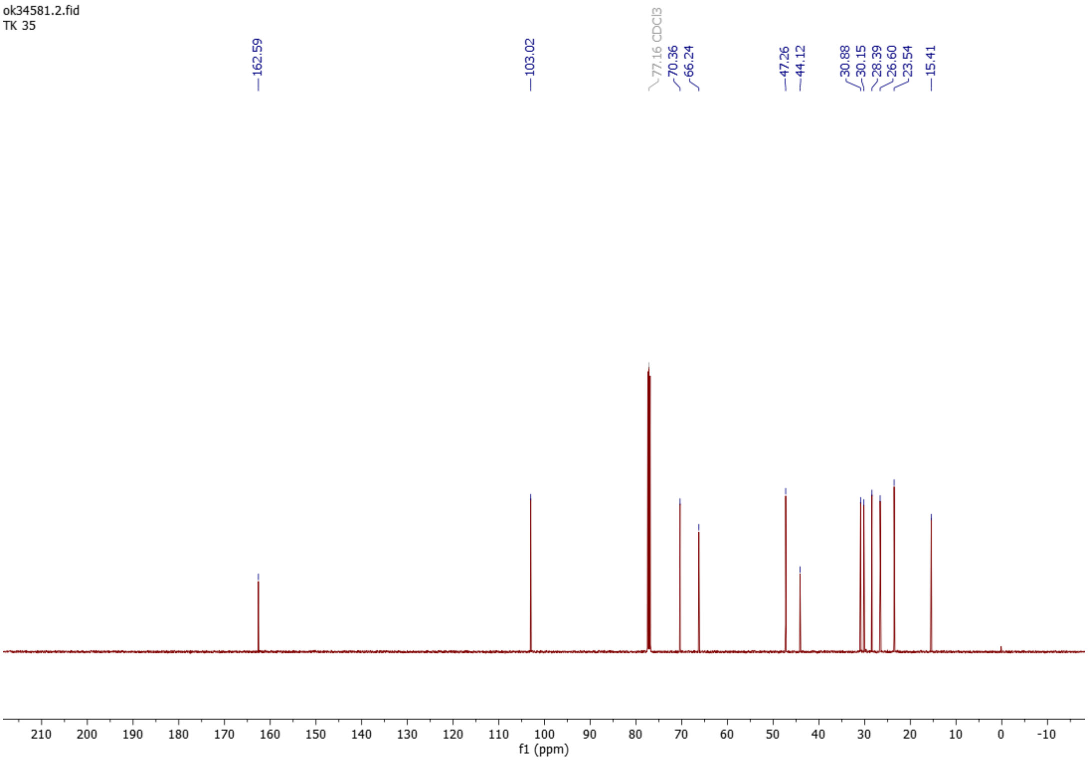

(*R*)-1,1-Dimethyl-2-methylene-5-(2-propoxyethyl)cyclopentane (8c)

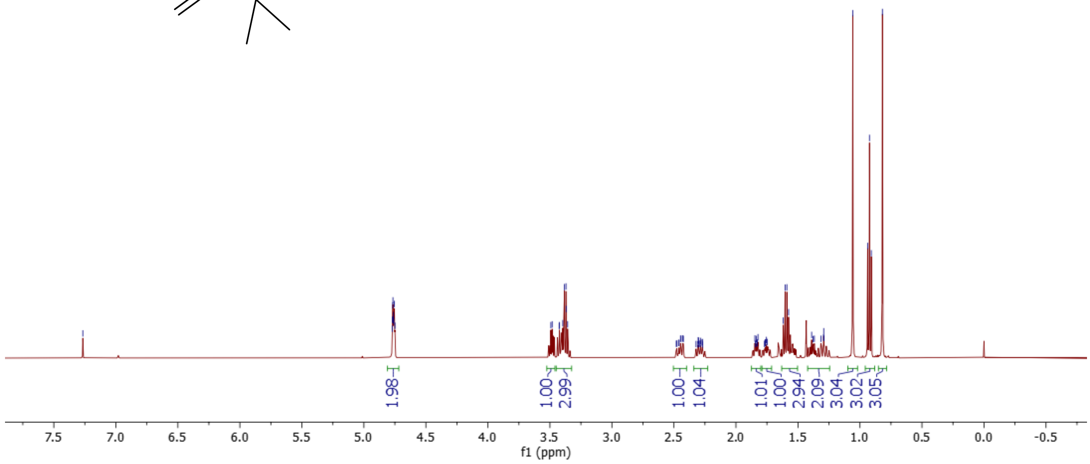

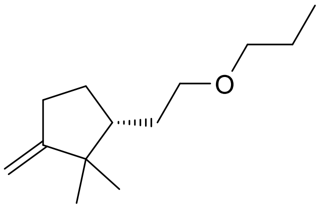

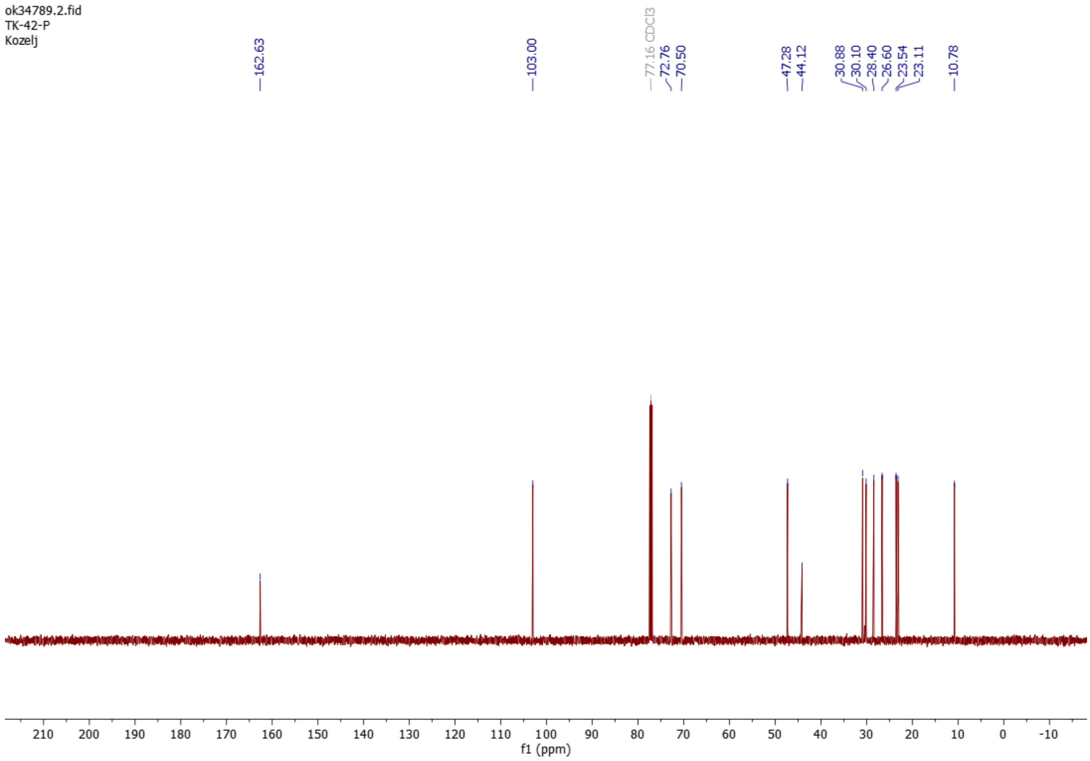

(*R*)-2-(2-Butoxyethyl)-1,1-dimethyl-5-methylenecyclopentane (8d)

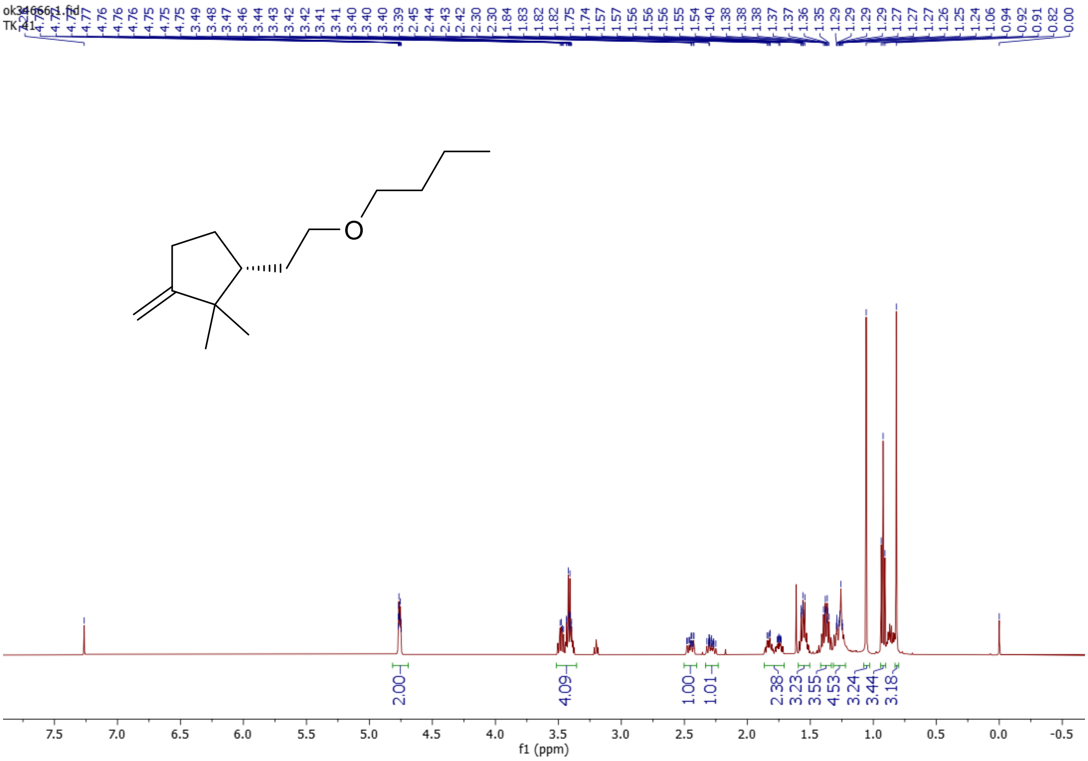

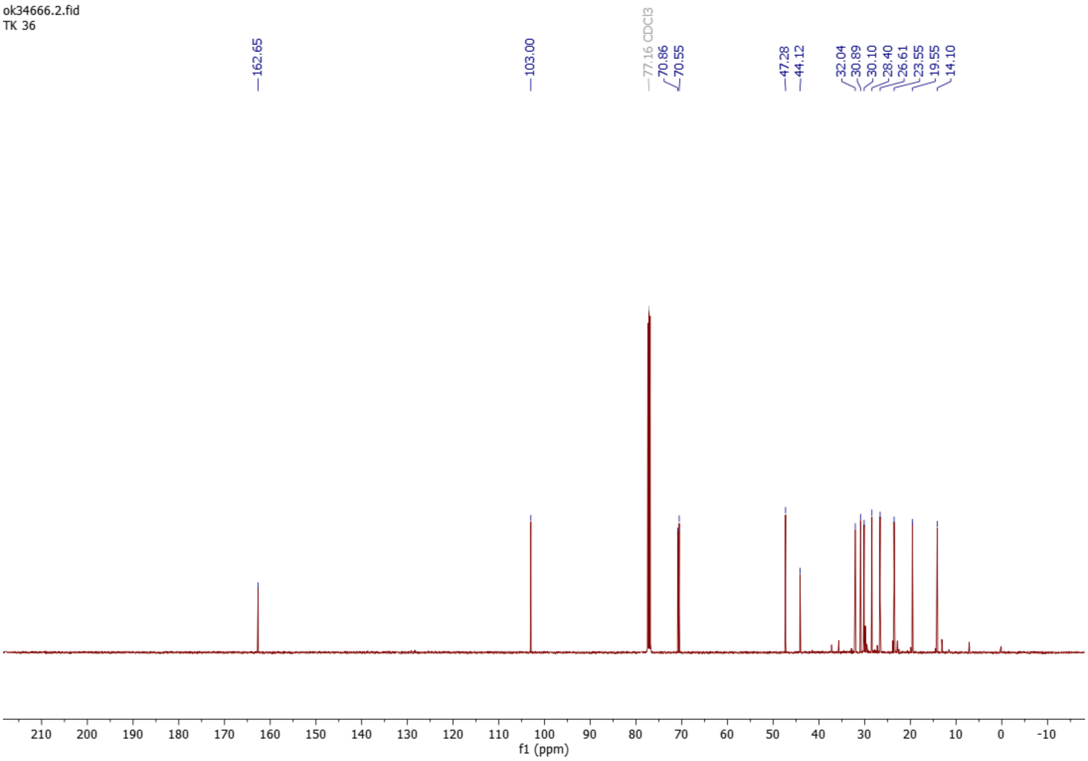

(*R*)-2-(2-(Allyloxy)ethyl)-1,1-dimethyl-5-methylenecyclopentane (8e)

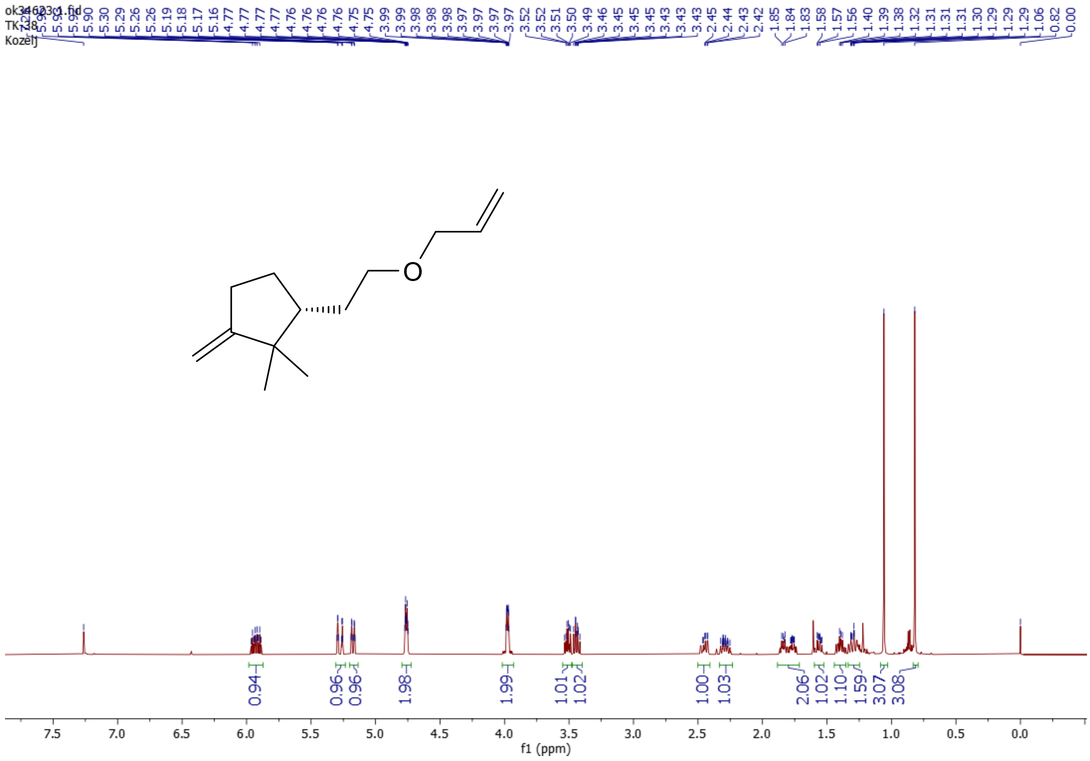

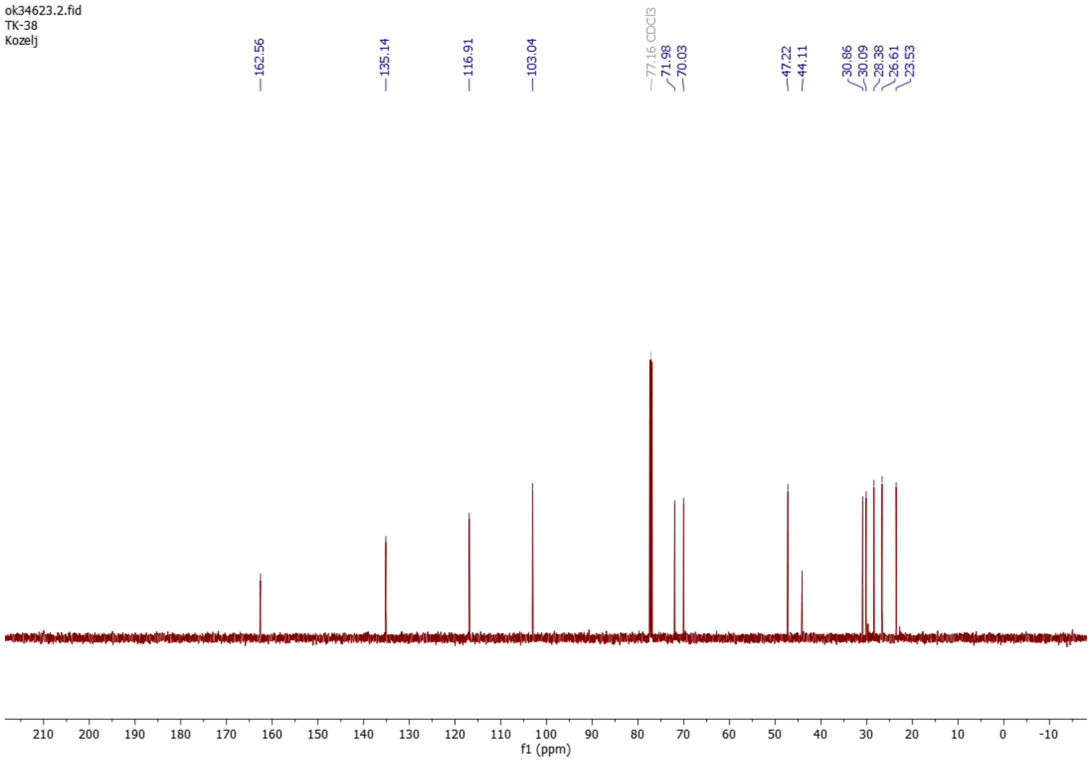

(*R*)-1,1-Dimethyl-2-(2-((3-methylbut-2-en-1-yl)oxy)ethyl)-5-methylenecyclopentane (8f)

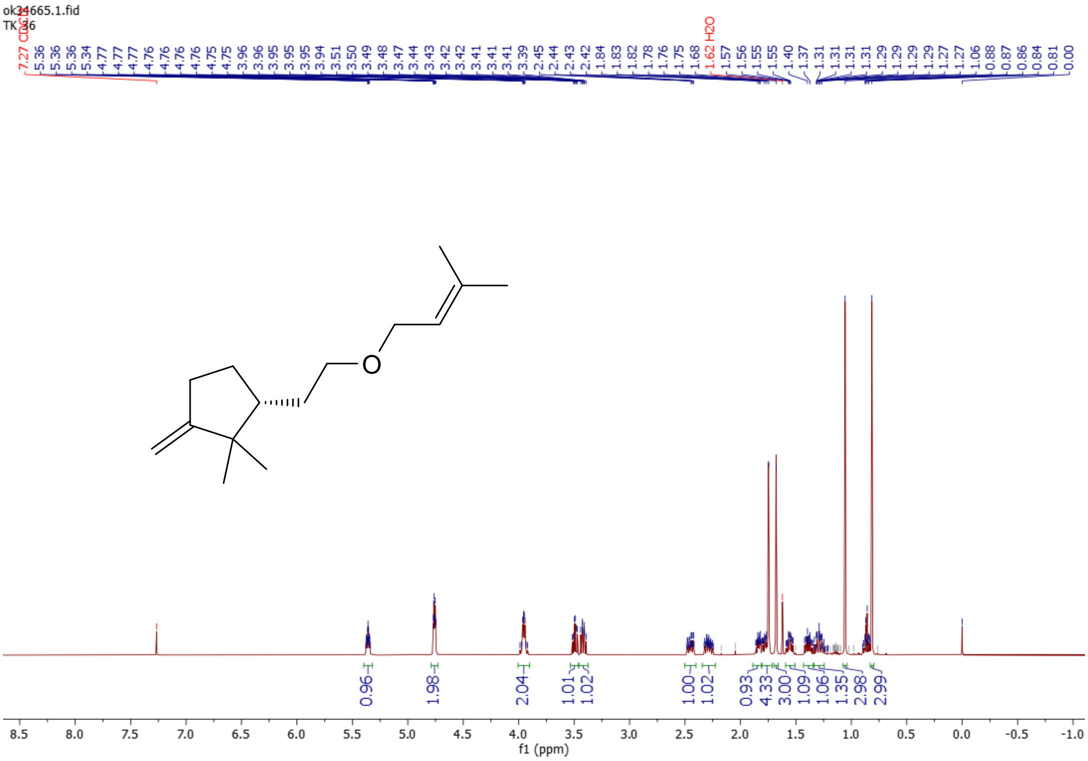

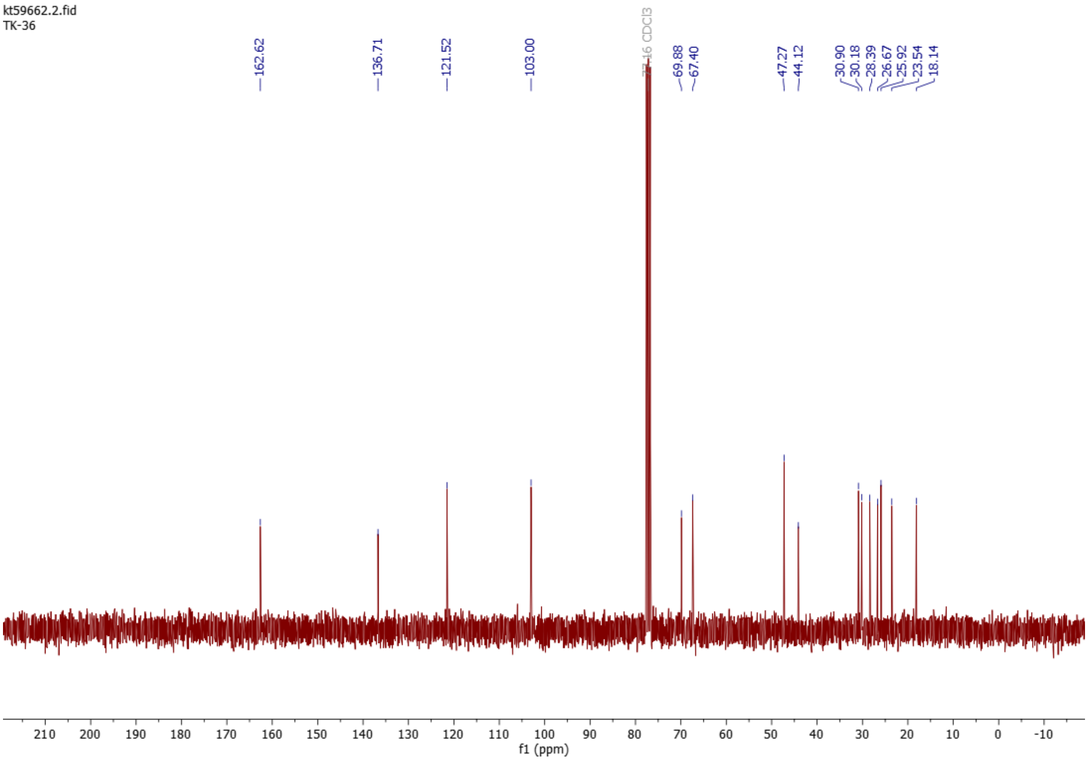

(*R*)-1,1-Dimethyl-2-methylene-5-(2-(prop-2-yn-1-yloxy)ethyl)cyclopentane (8g)

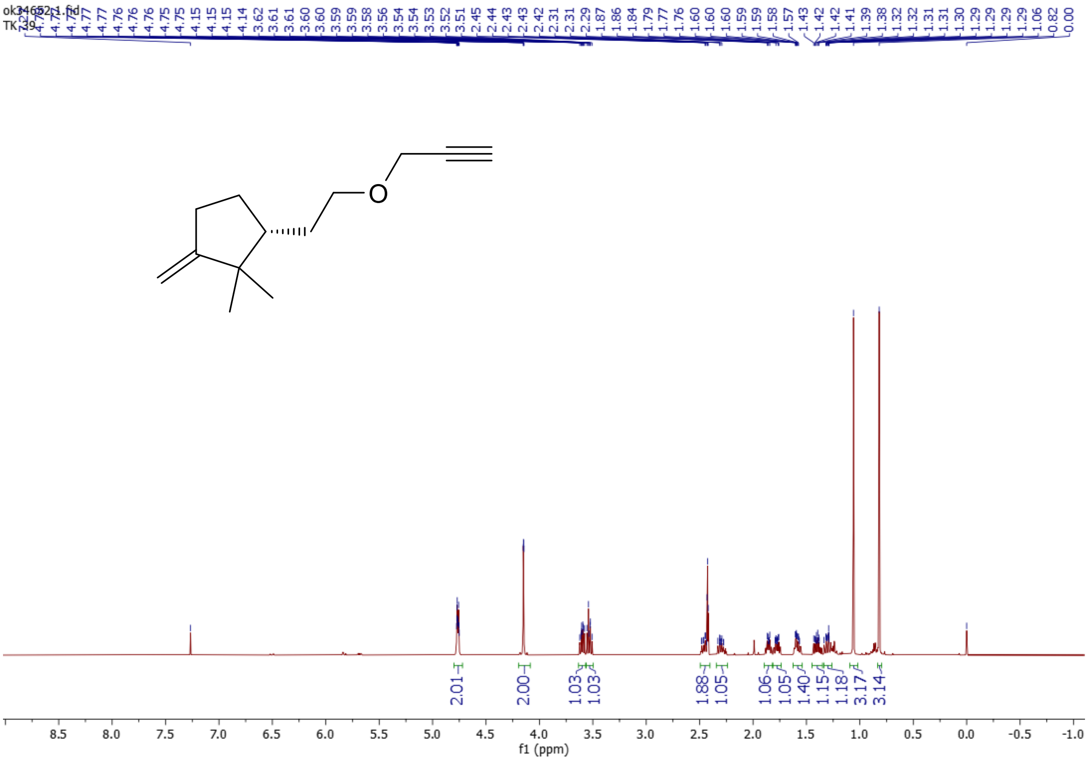

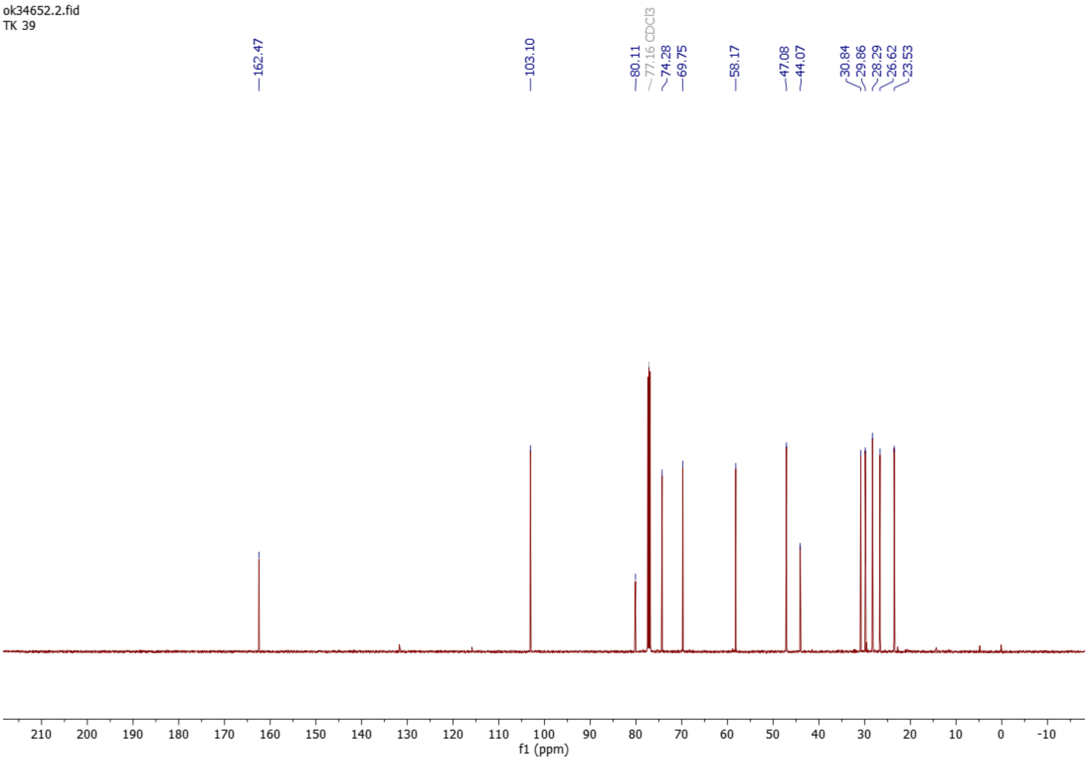

(*R*)-((2-(2,2-Dimethyl-3-methylenecyclopentyl)ethoxy)methyl)benzene (8h)

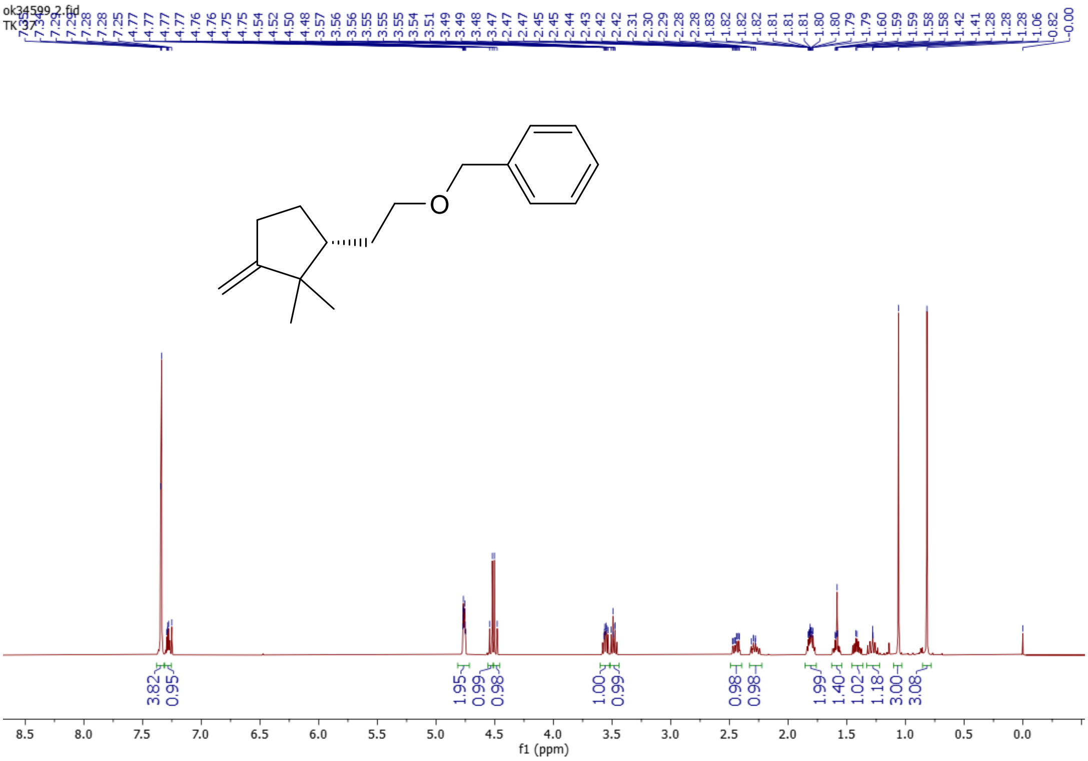

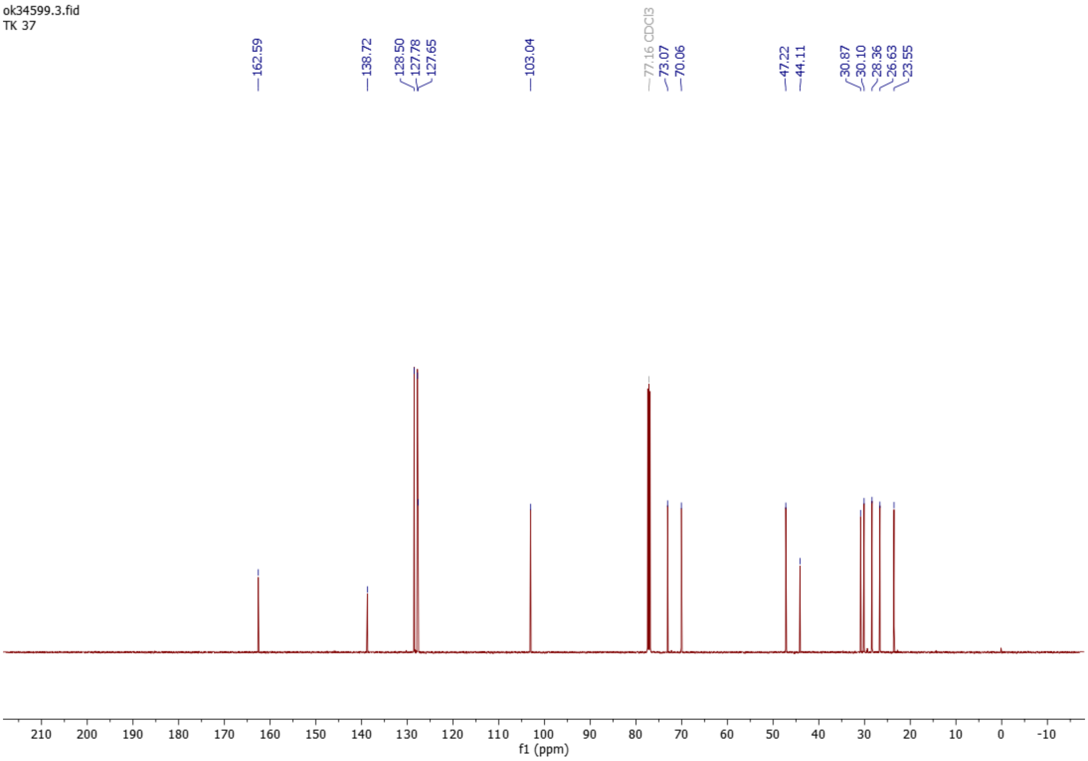

(*R*)-2-(2,2-Dimethyl-3-methylenecyclopentyl)-*N*-methoxy-*N*-methylacetamide (9)

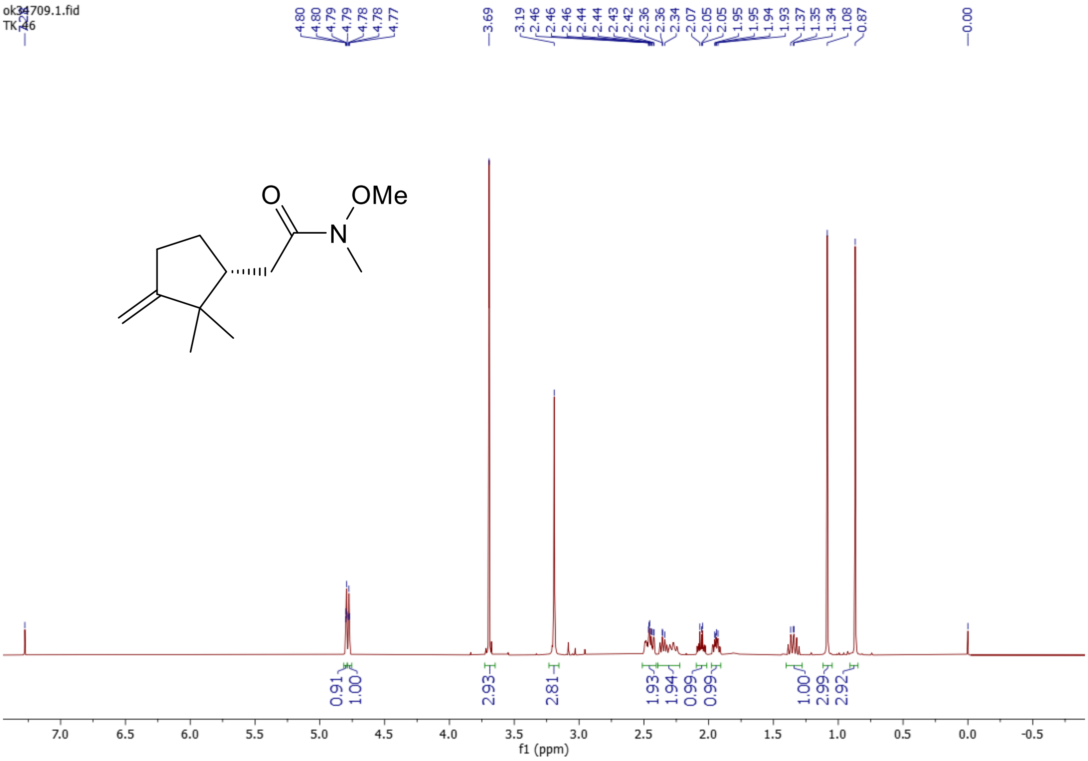

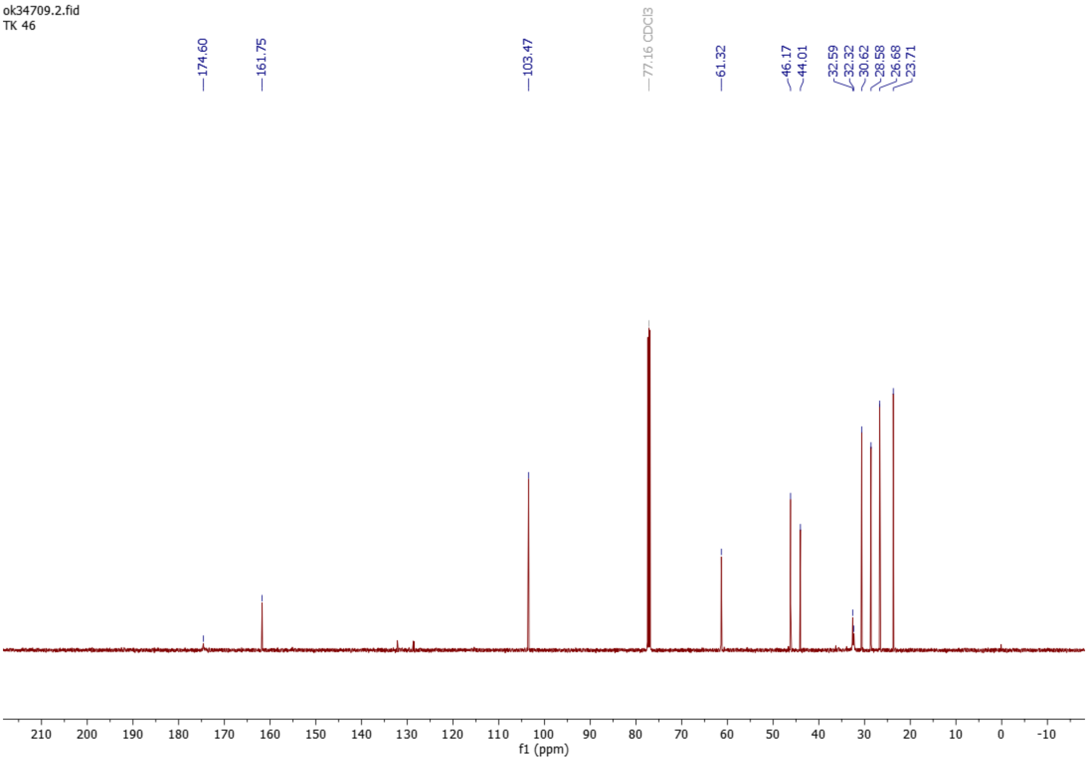

(*R*)-1-(2,2-Dimethyl-3-methylenecyclopentyl)propan-2-one (10a)

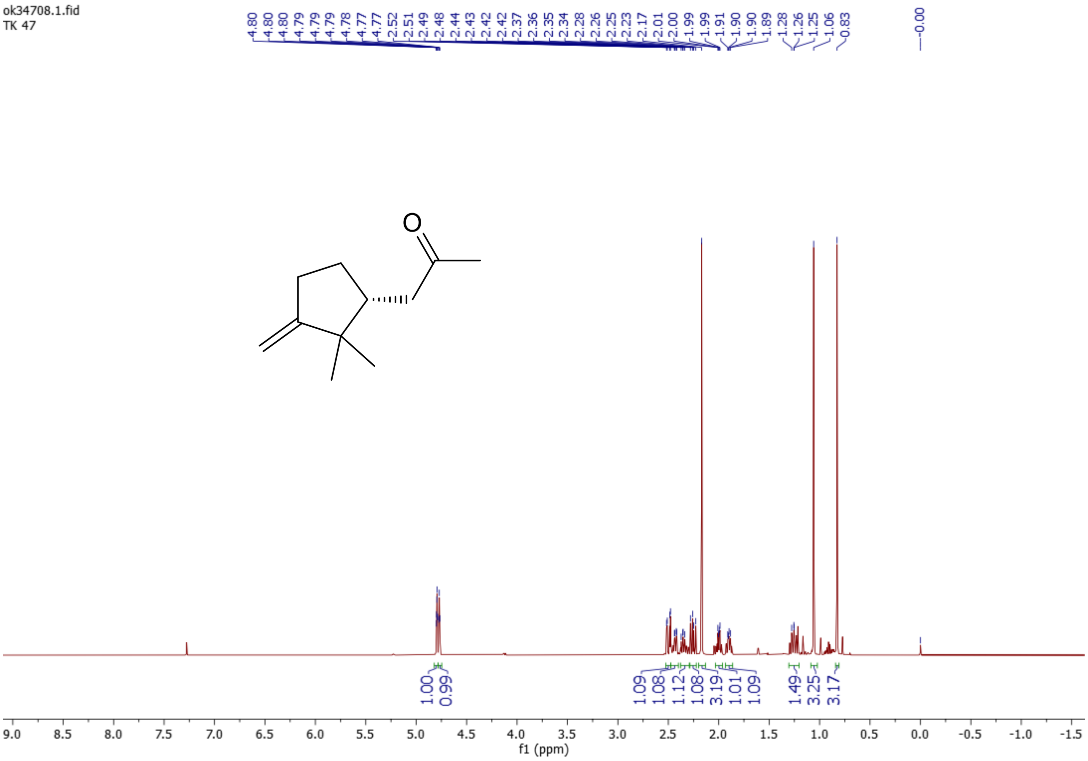

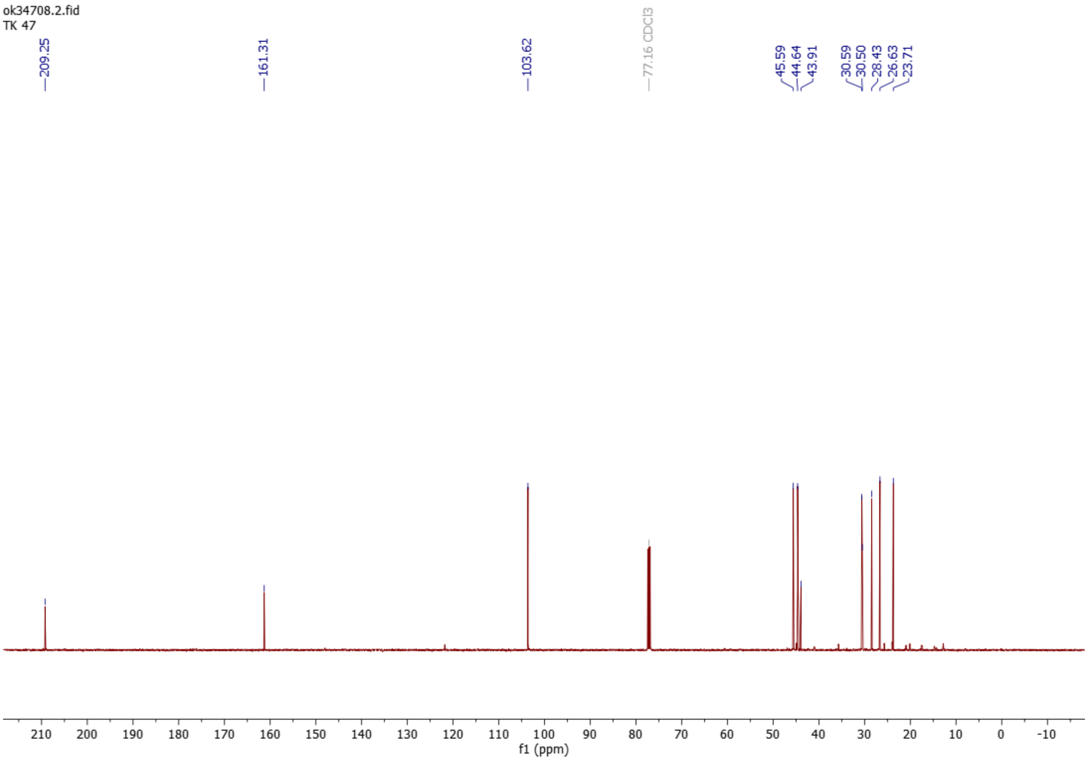

(*R*)-1-(2,2-Dimethyl-3-methylenecyclopentyl)butan-2-one (10b)

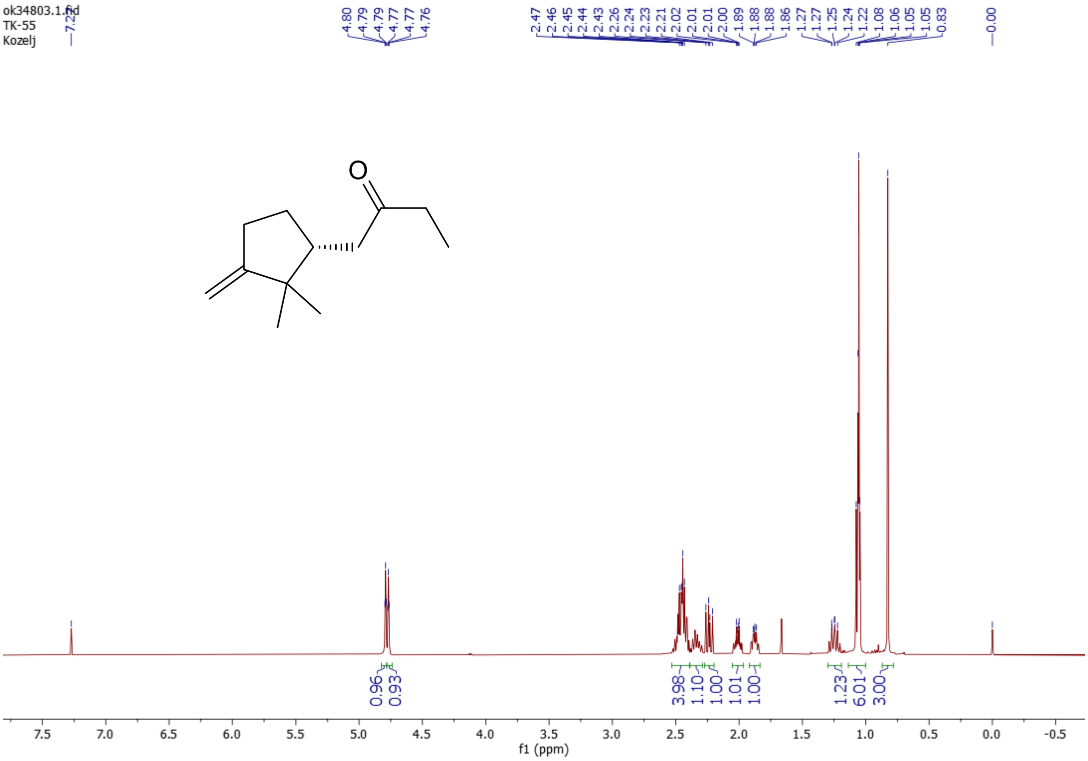

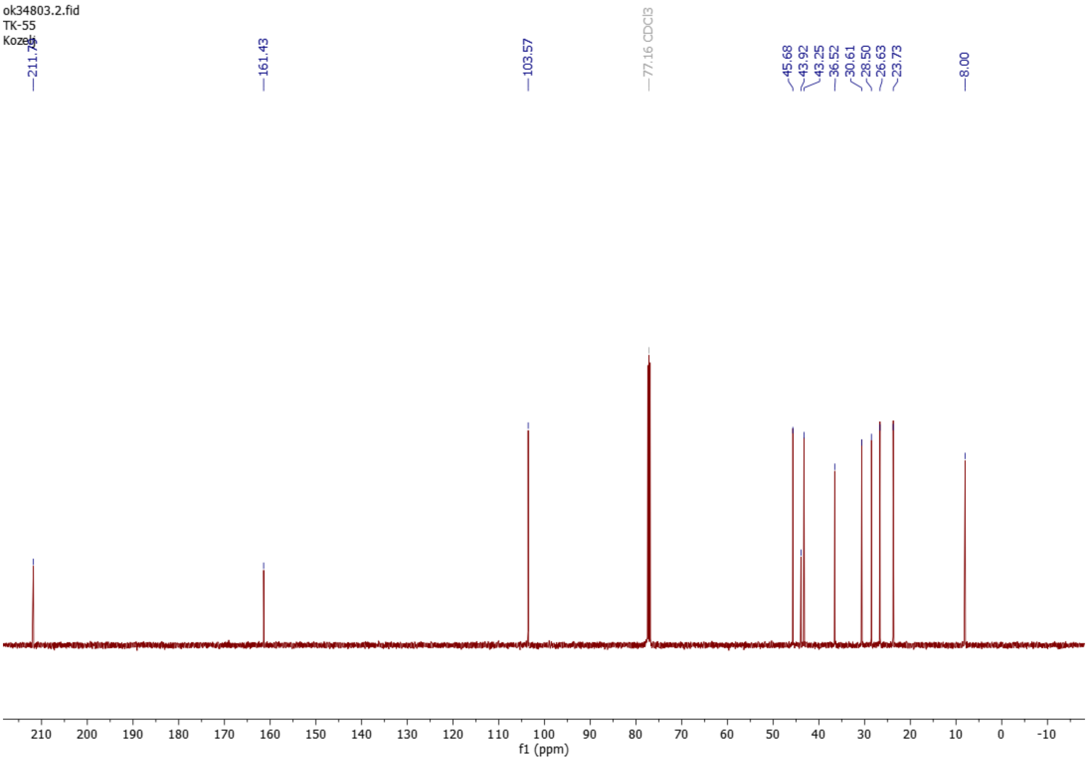

(*R*)-1-(2,2-Dimethyl-3-methylenecyclopentyl)pentan-2-one (10c)

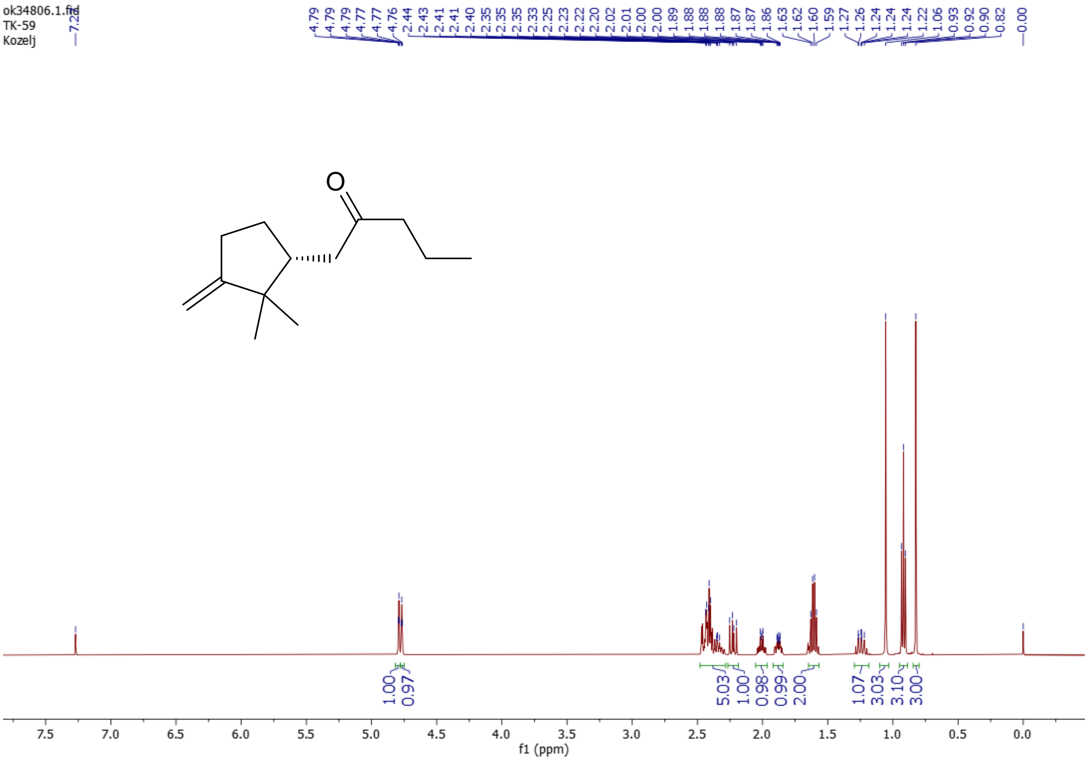

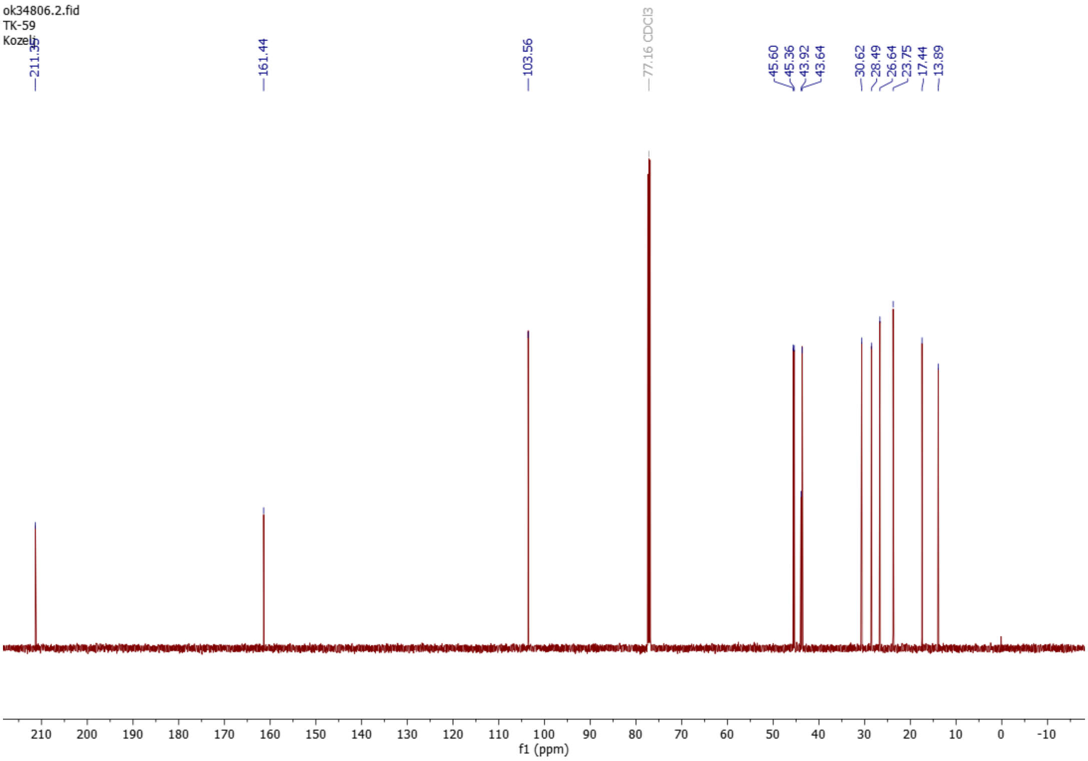

(*R*)-1-(2,2-Dimethyl-3-methylenecyclopentyl)hexan-2-one (10d)

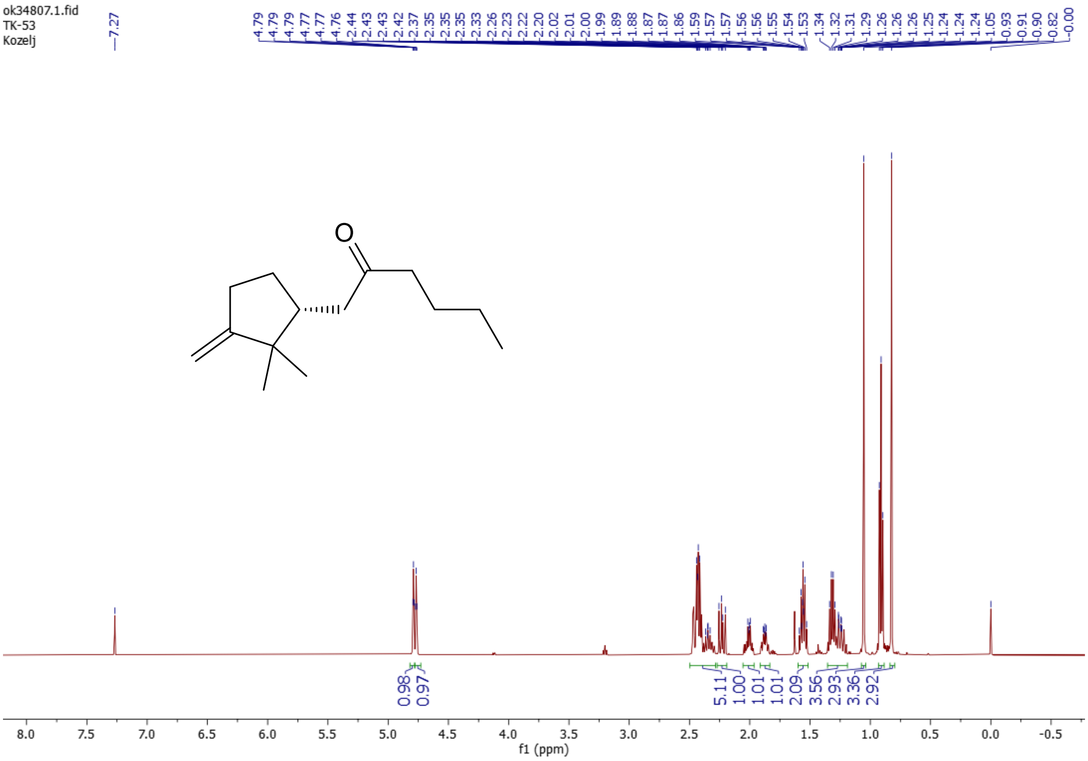

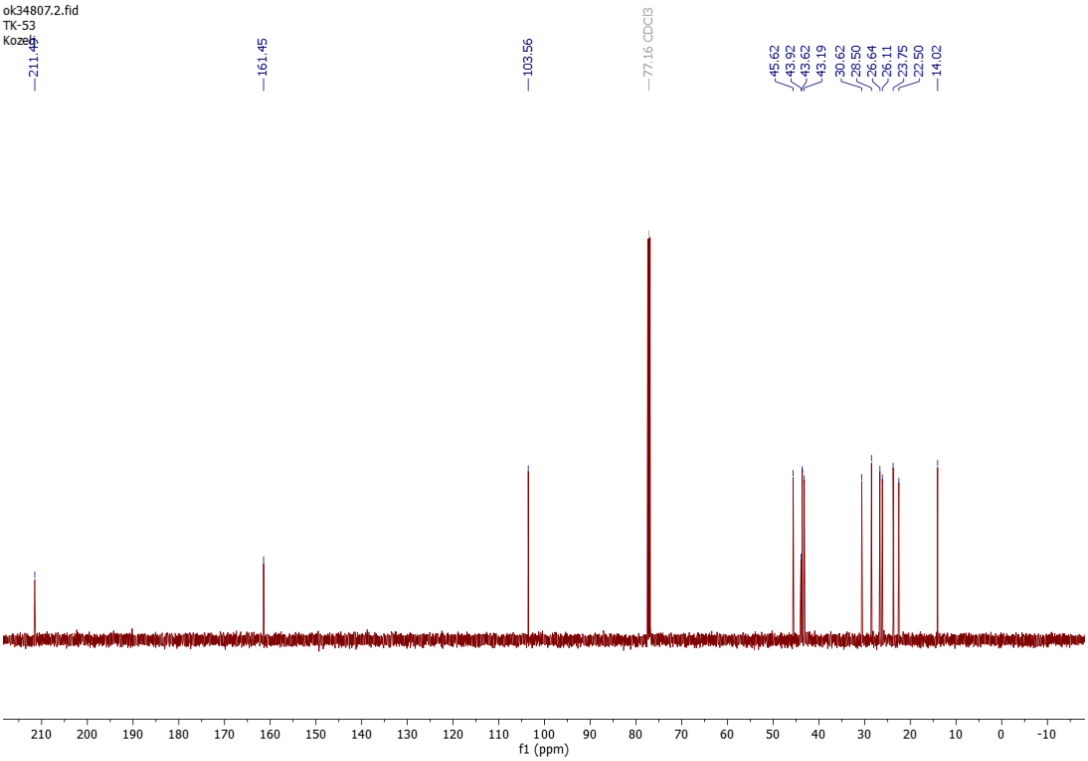

(*R*)-1-(2,2-Dimethyl-3-methylenecyclopentyl)-3-methylbutan-2-one (10e)

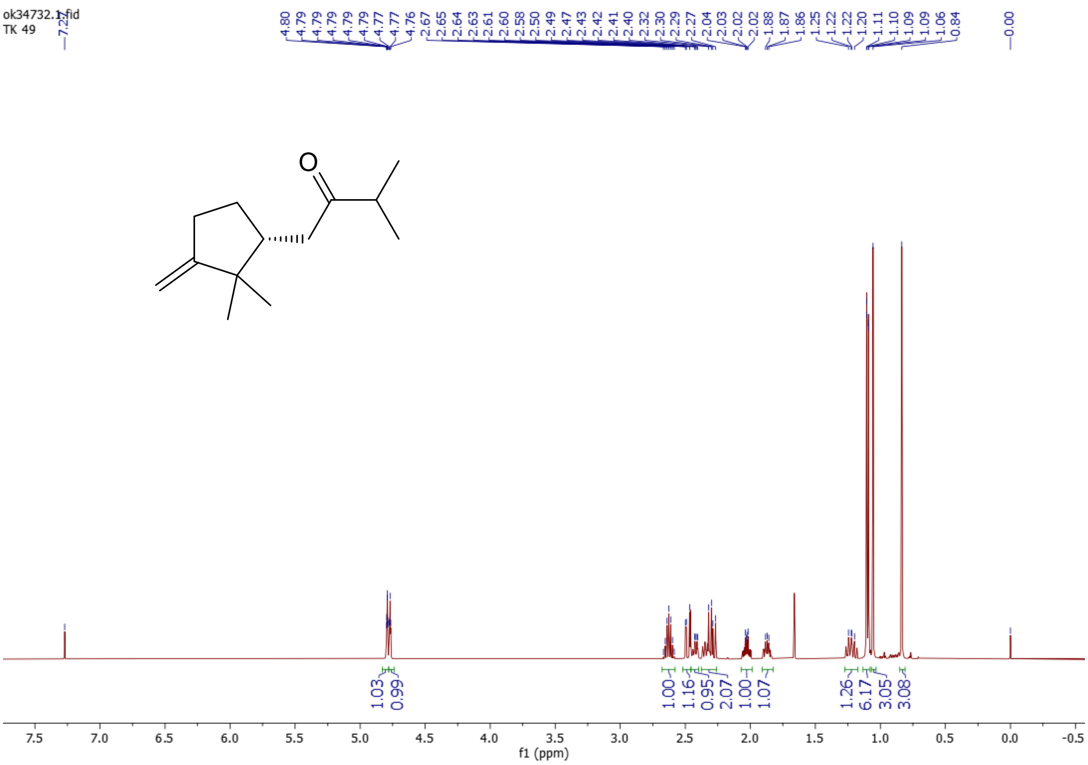

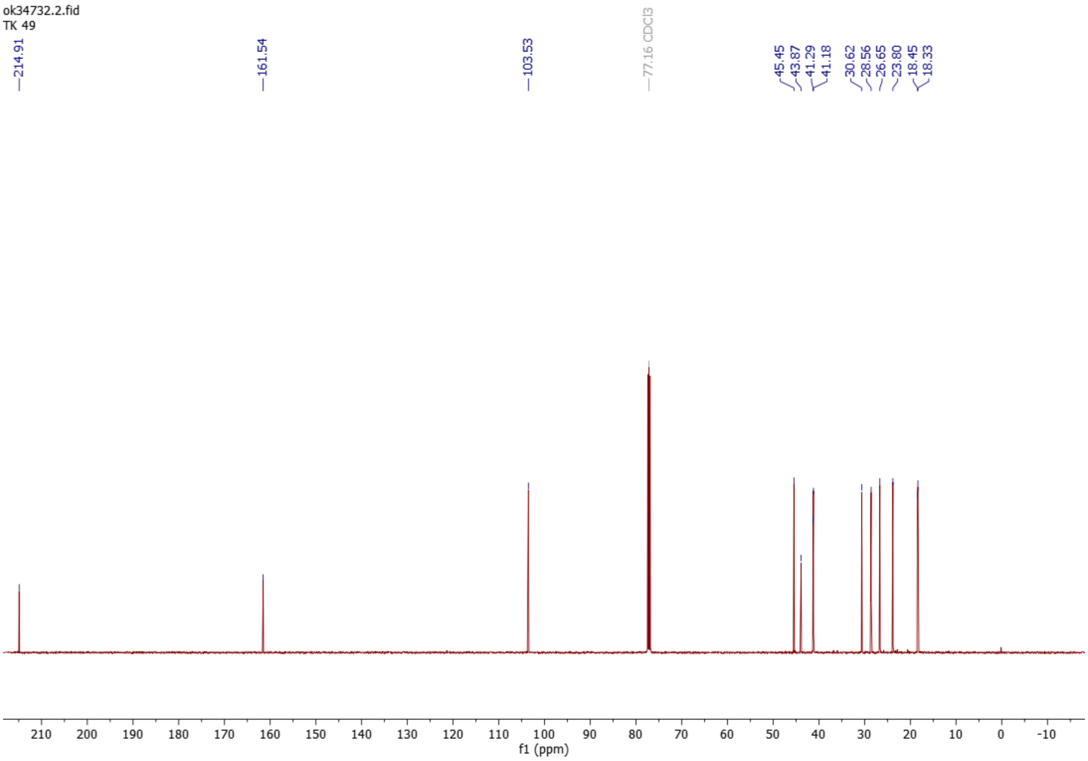

(*R*)-1-(2,2-Dimethyl-3-methylenecyclopentyl)pent-4-en-2-one (10f)

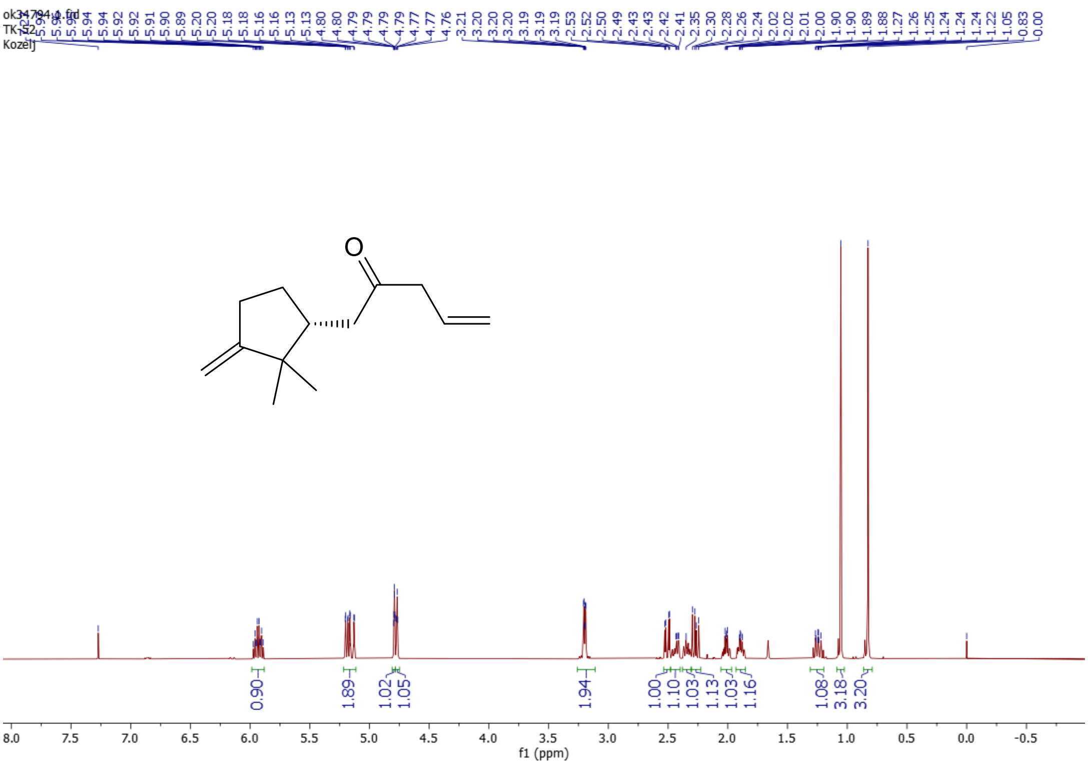

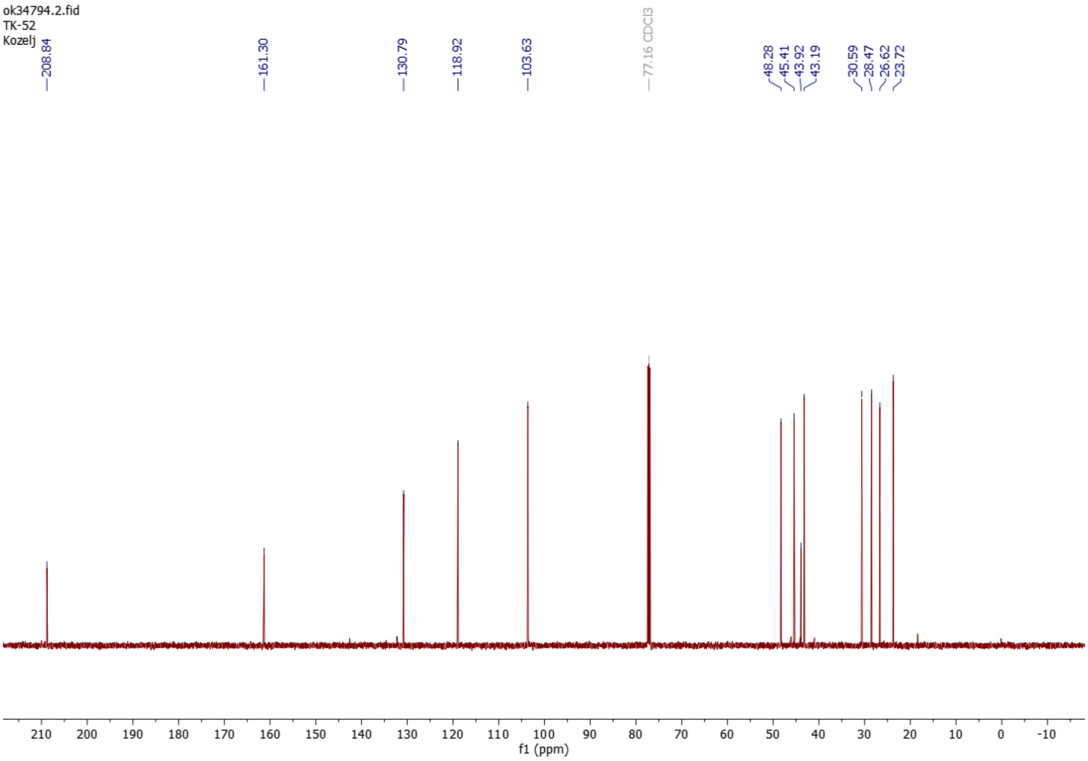

(*R*)-1-(2,2-Dimethyl-3-methylenecyclopentyl)-3-phenylpropan-2-one (10g)

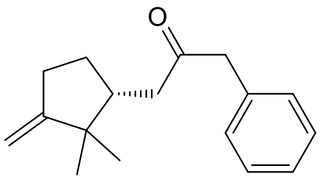

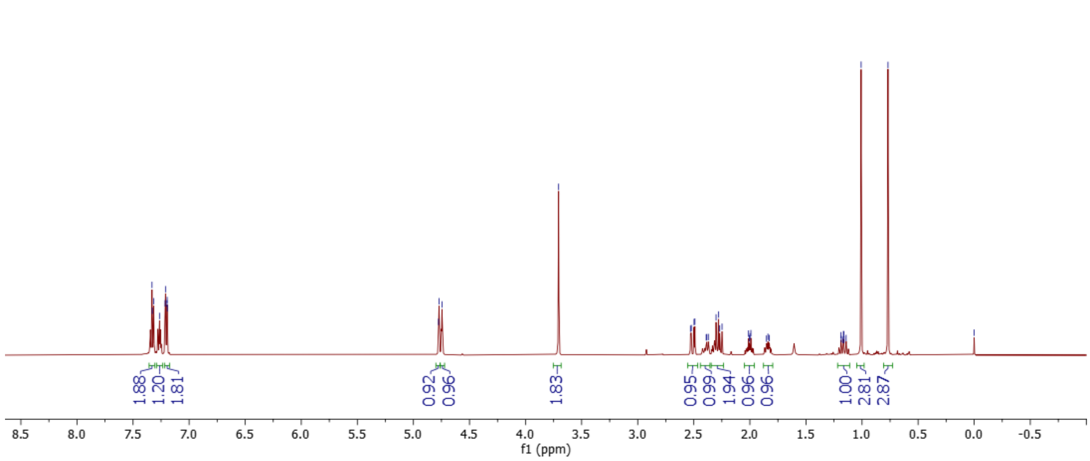

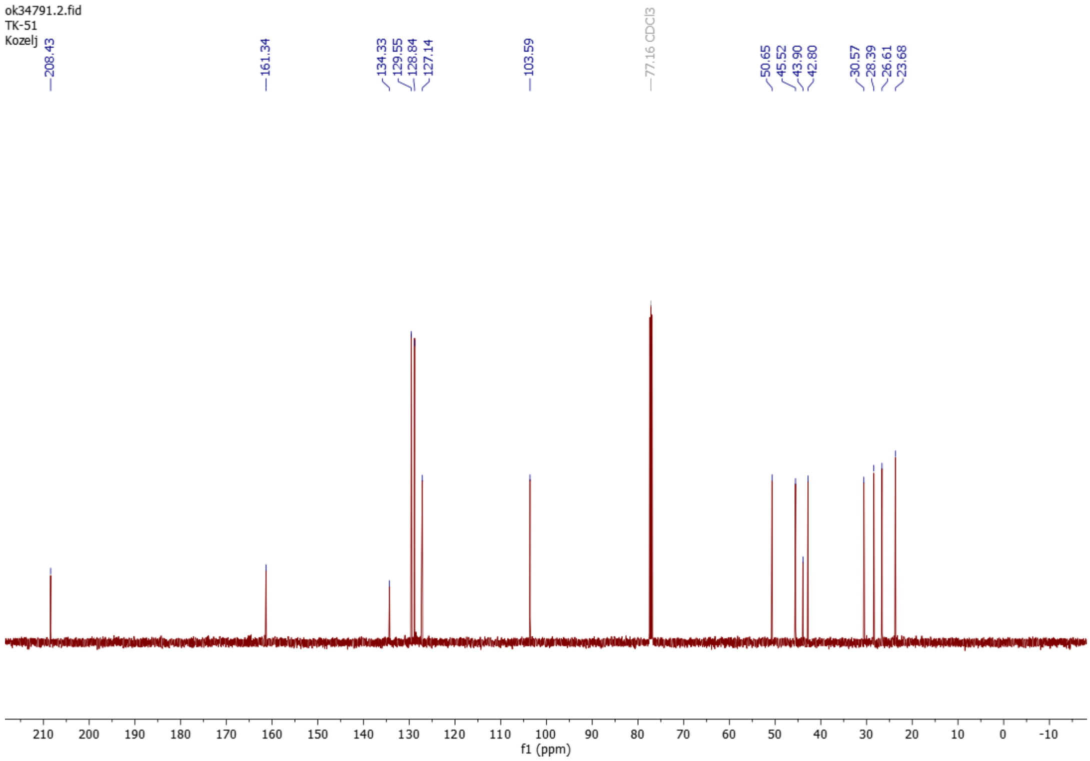

(*R*)-1-(2,2-Dimethyl-3-methylenecyclopentyl)but-3-en-2-one (10h)

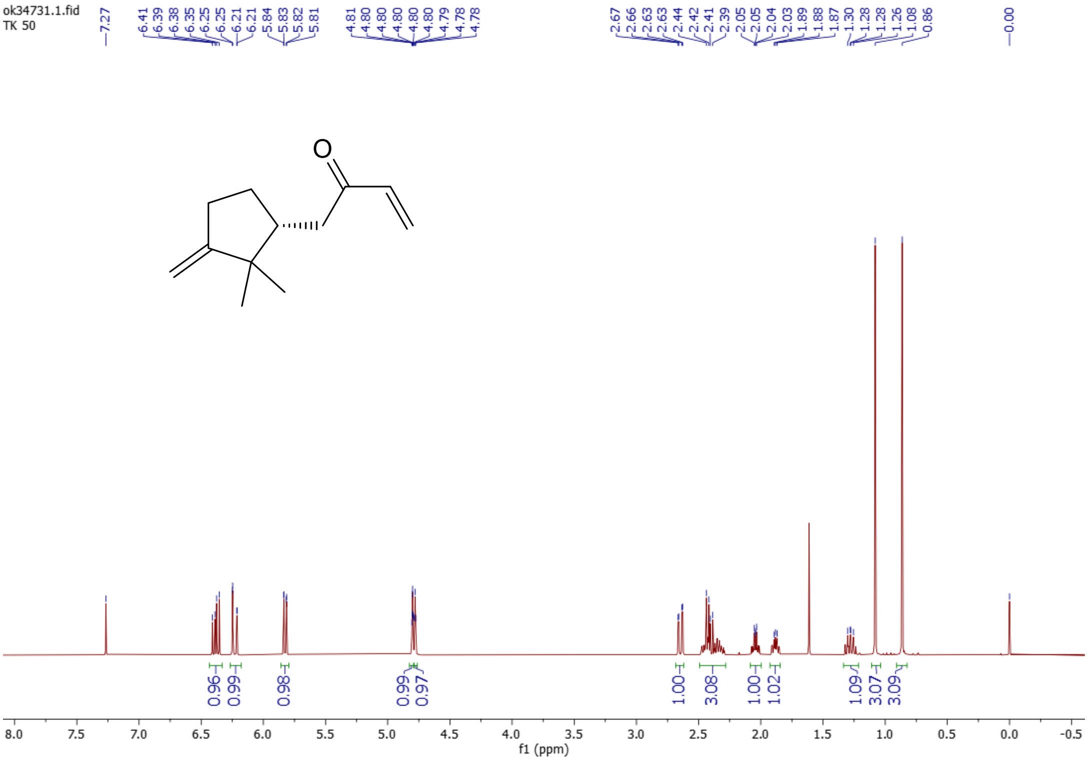

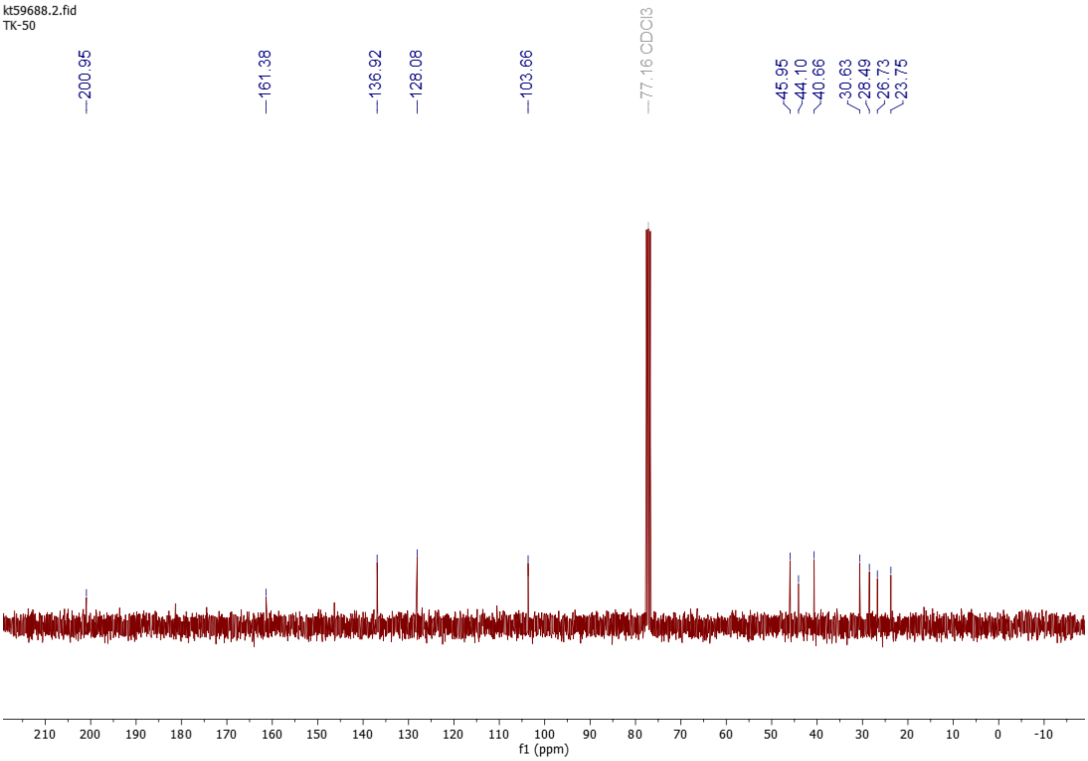

(*R*)-1-(2,2-Dimethyl-3-methylenecyclopentyl)but-3-yn-2-one (10i)

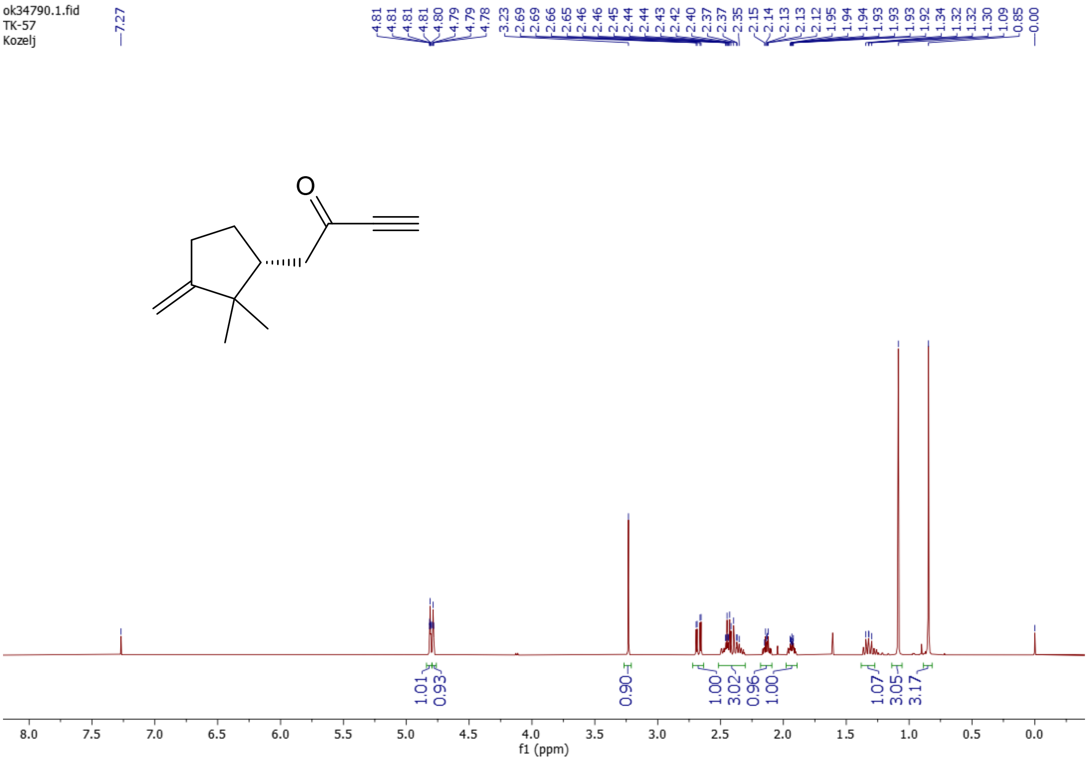

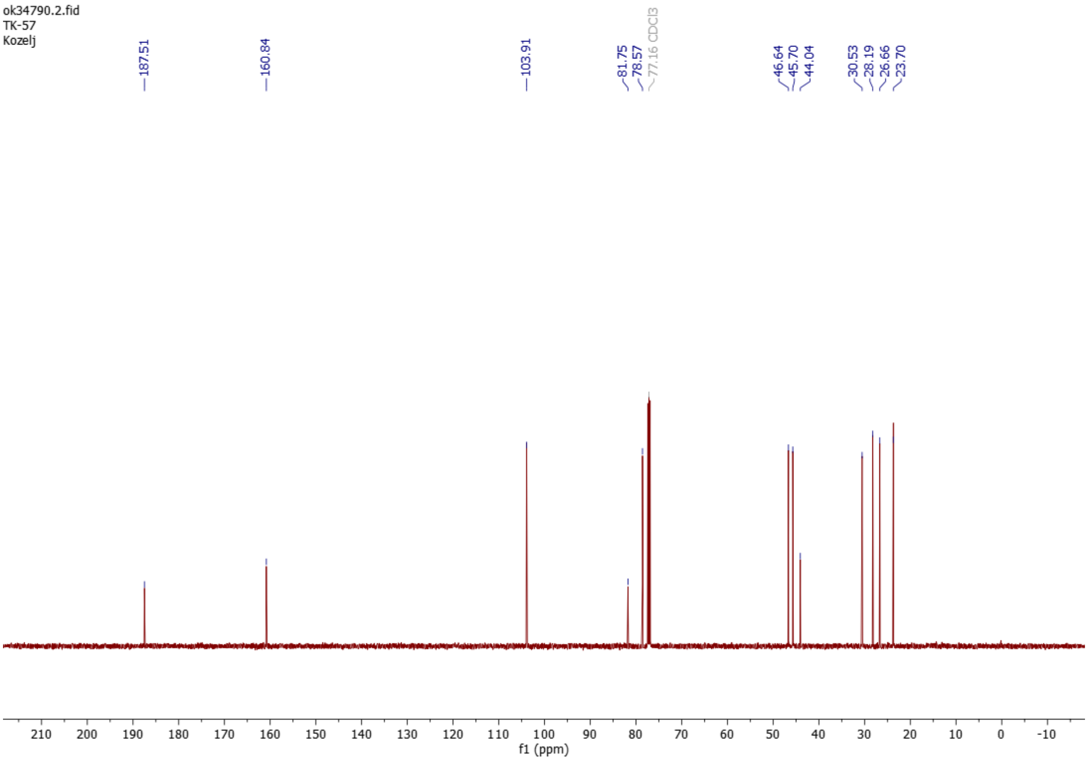

(*R*)-1-(2,2-Dimethyl-3-methylenecyclopentyl)pent-3-yn-2-one (10j)

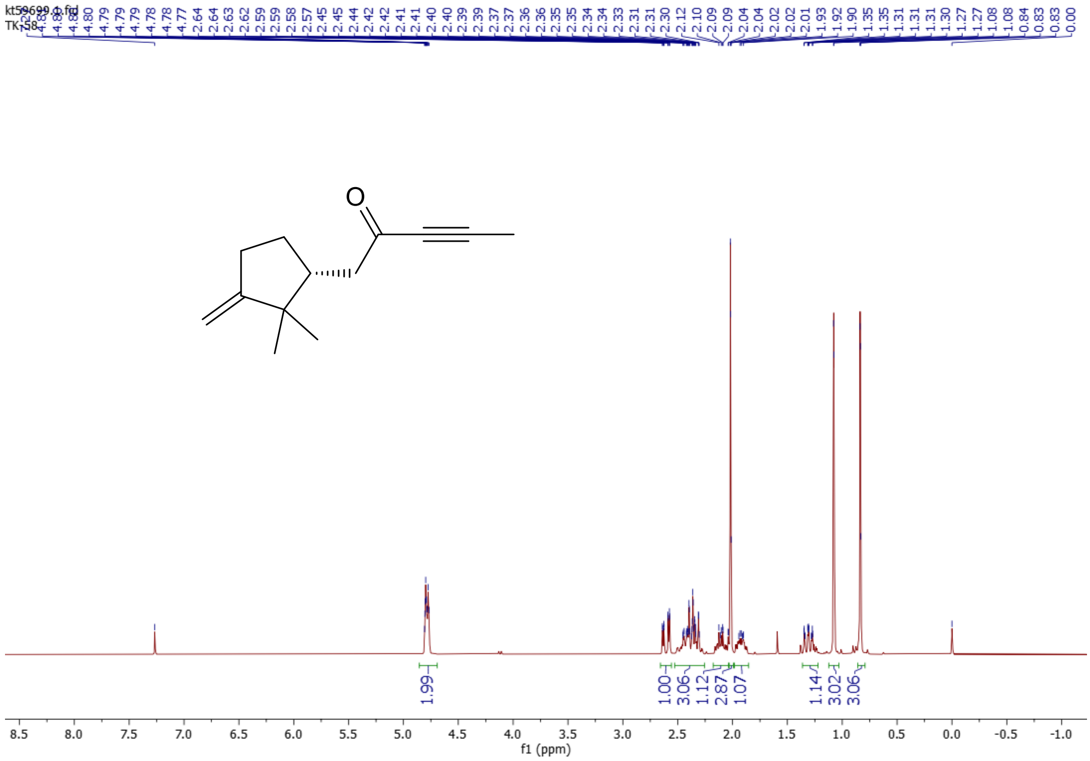

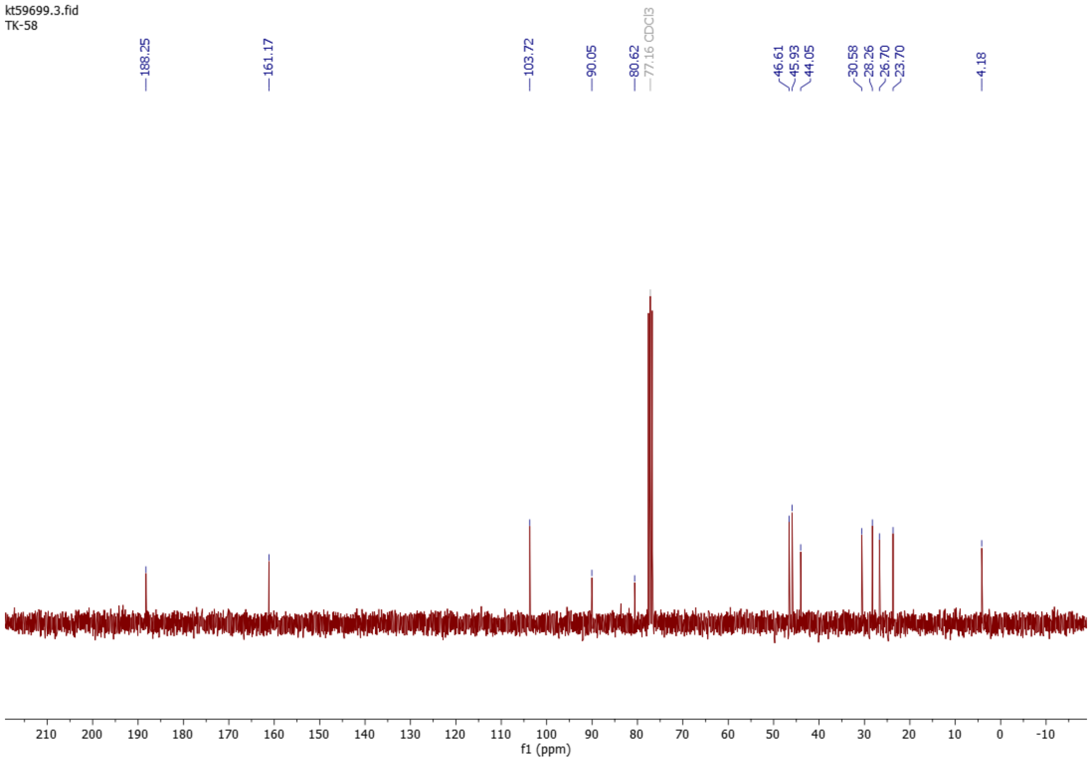

(*R*)-2-(2,2-Dimethyl-3-methylenecyclopentyl)-1-phenylethan-1-one (10k)

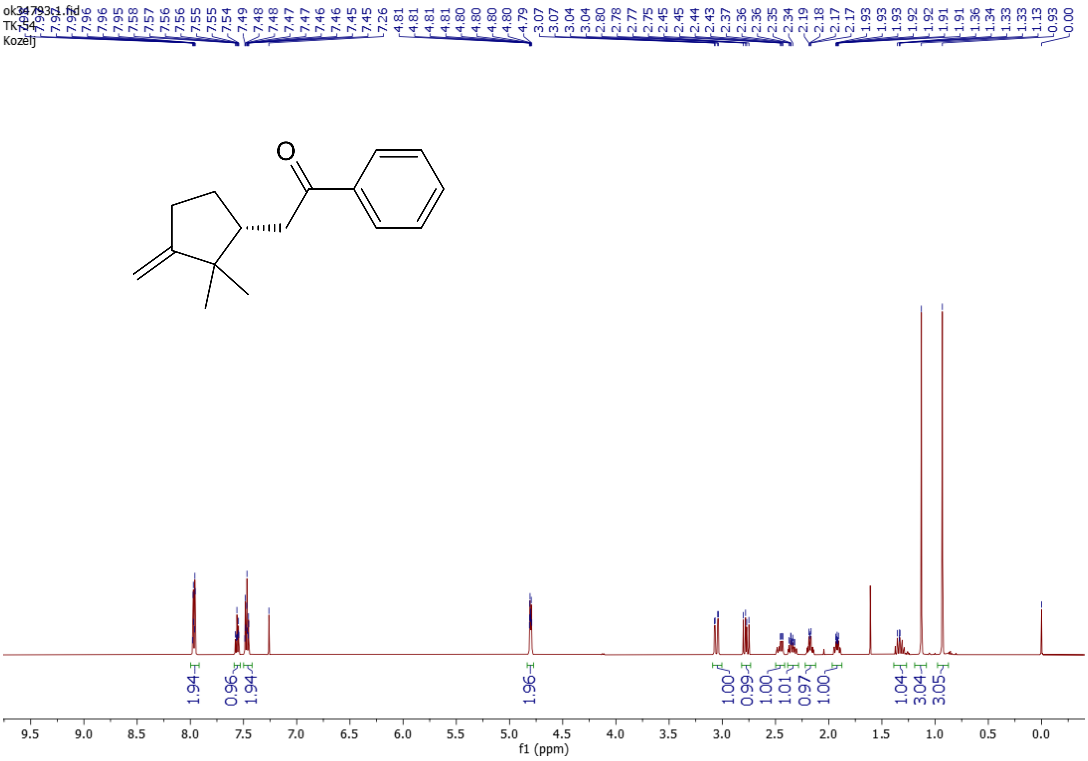

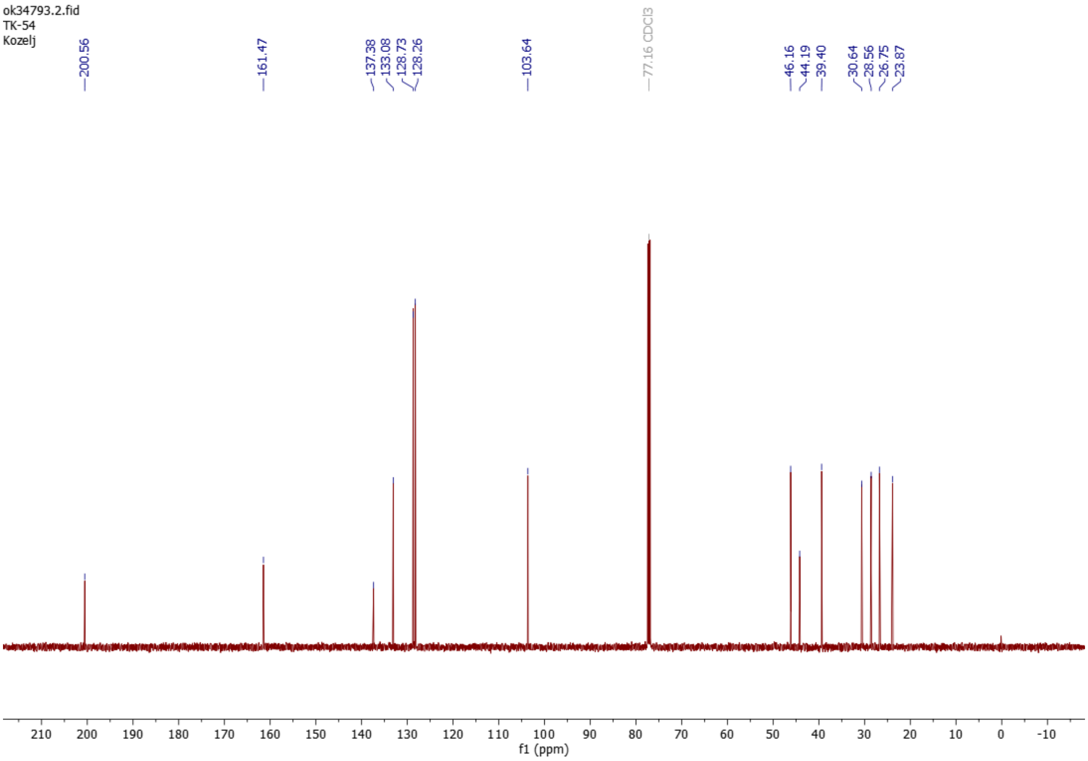

1-((*R*)-2,2-Dimethyl-3-methylenecyclopentyl)propan-2-ol (11a)

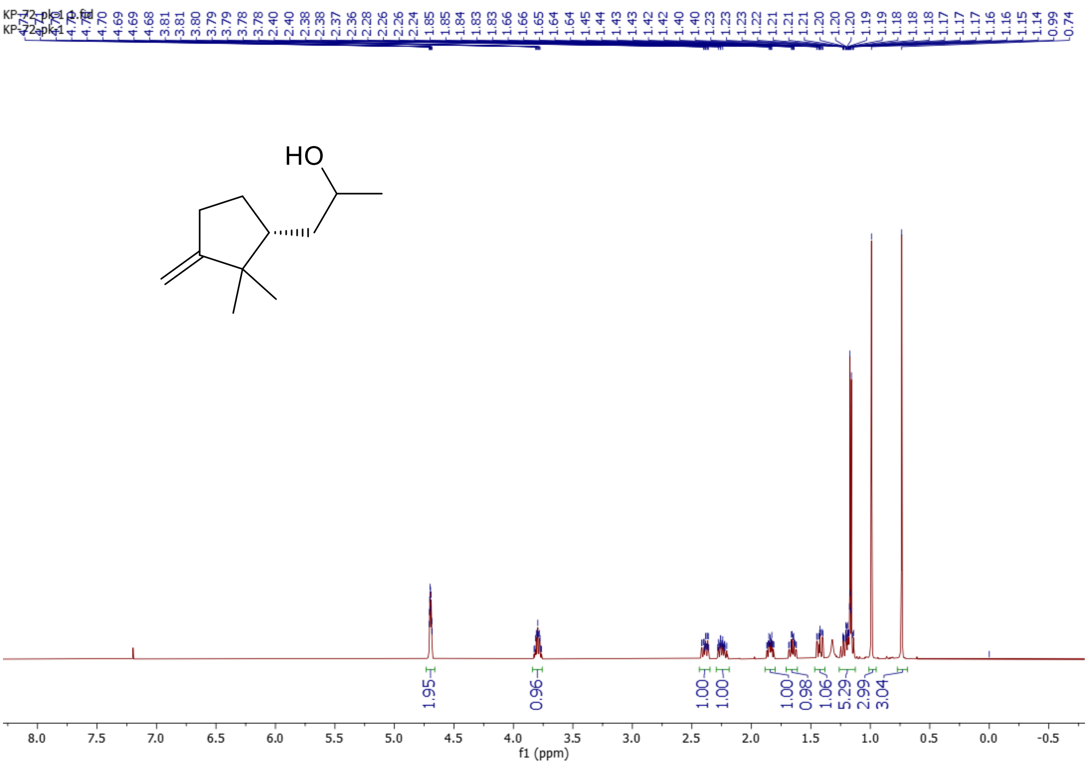

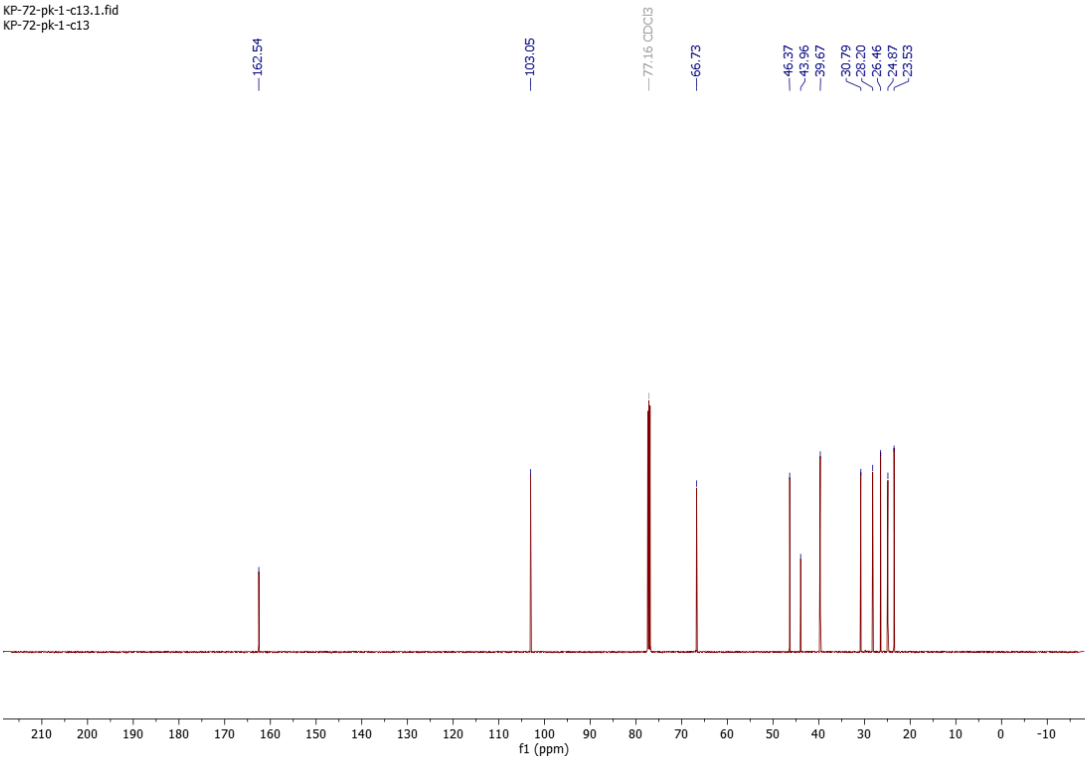

1-((*R*)-2,2-Dimethyl-3-methylenecyclopentyl)pent-4-en-2-ol (11b)

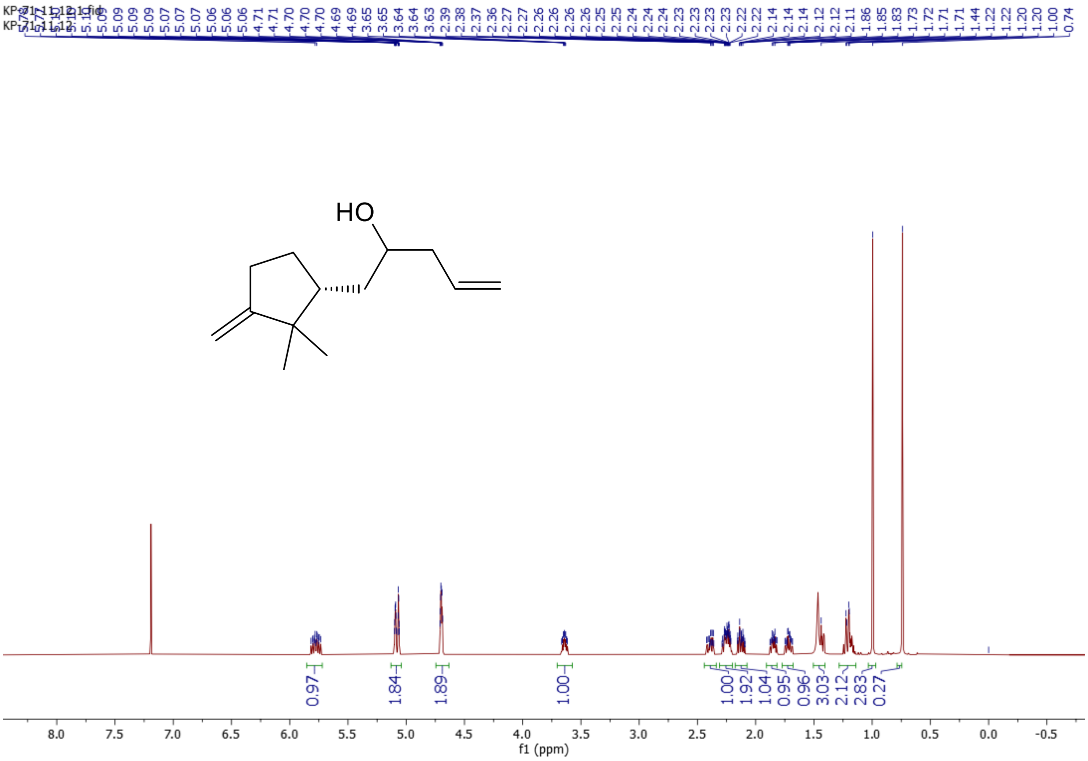

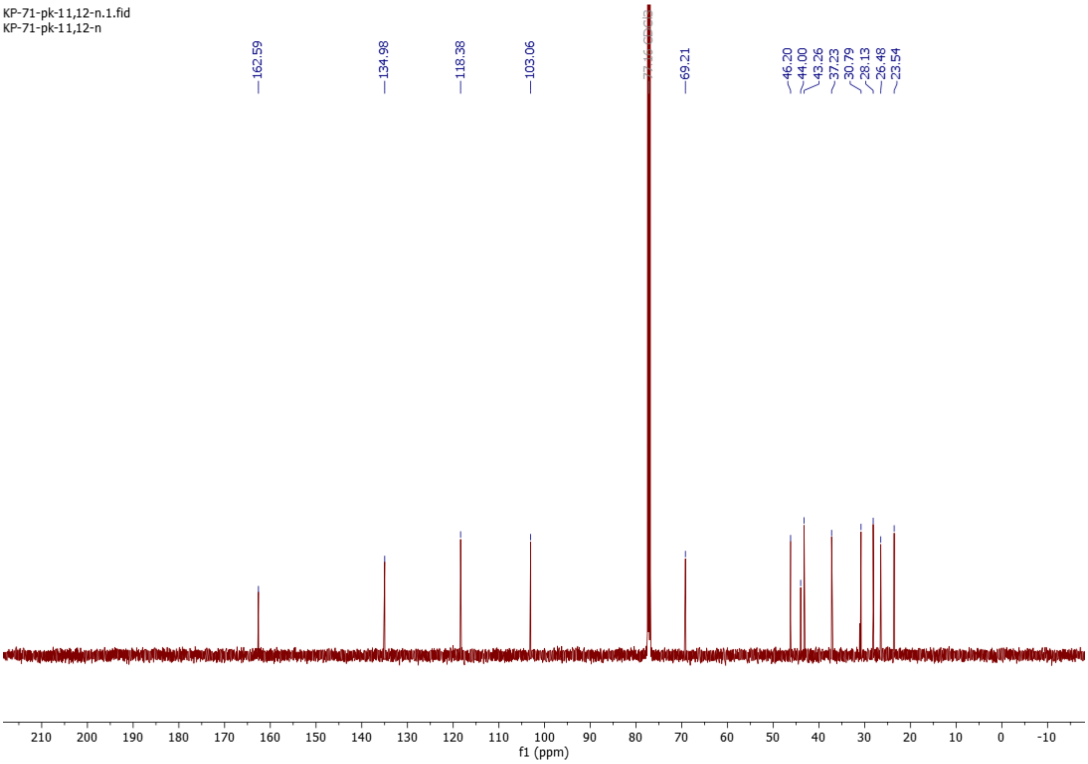

1-((*R*)-2,2-Dimethyl-3-methylenecyclopentyl)pent-4-en-2-ol (11b')

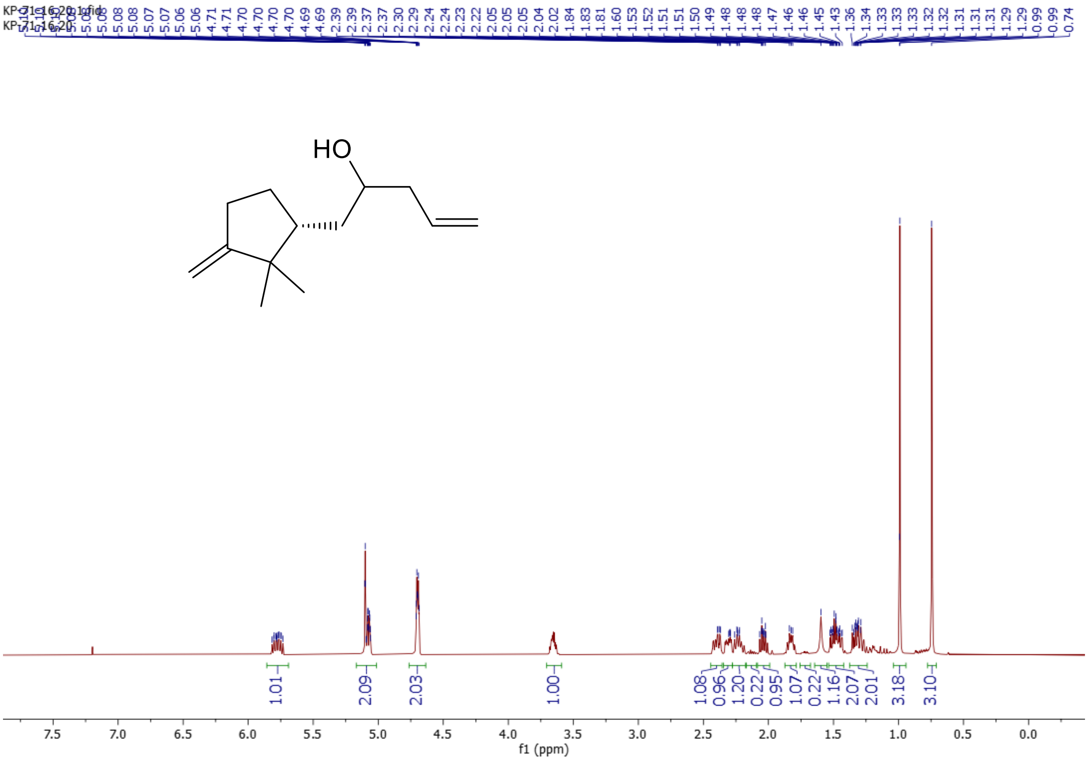

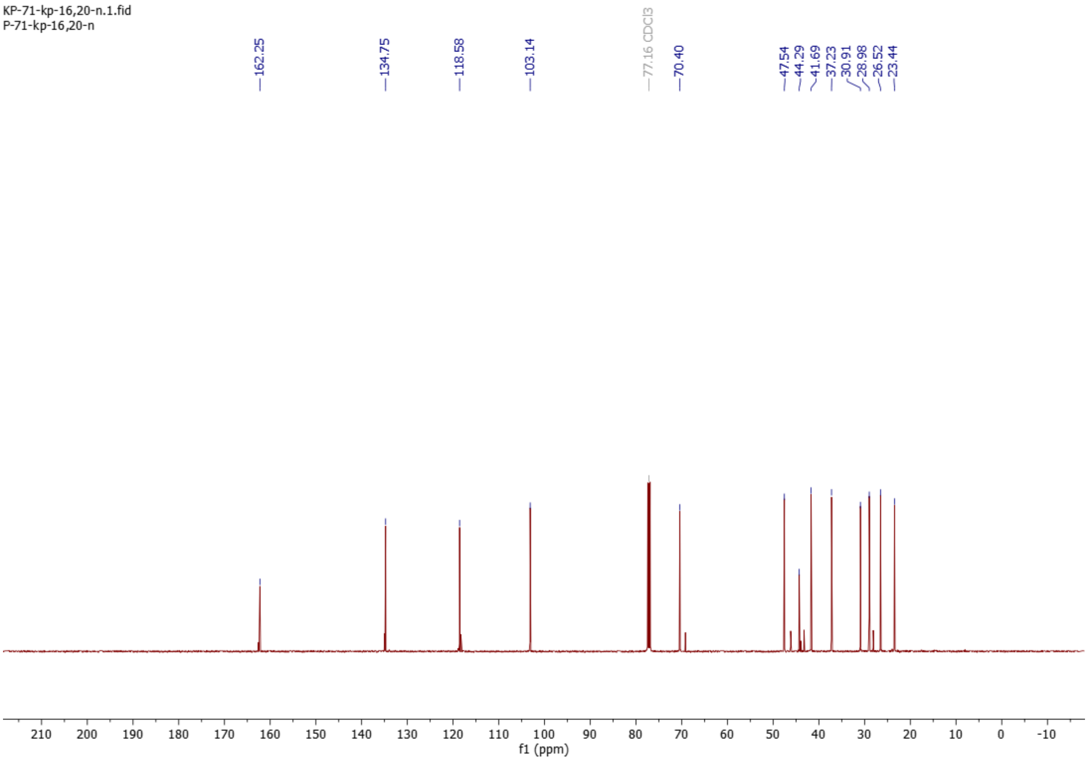

1-((*R*)-2,2-Dimethyl-3-methylenecyclopentyl)-3-methylbutan-2-ol (11c/11c')

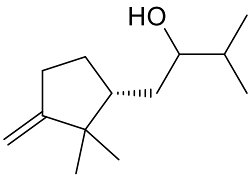

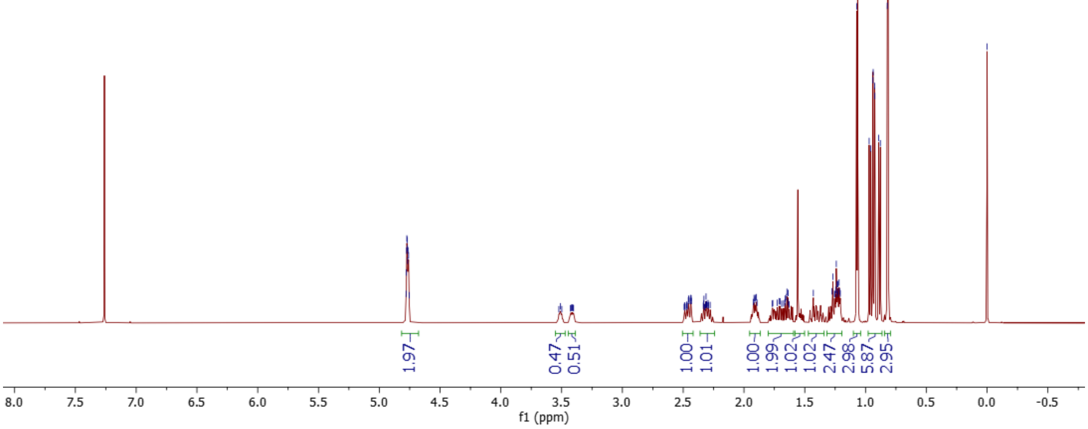

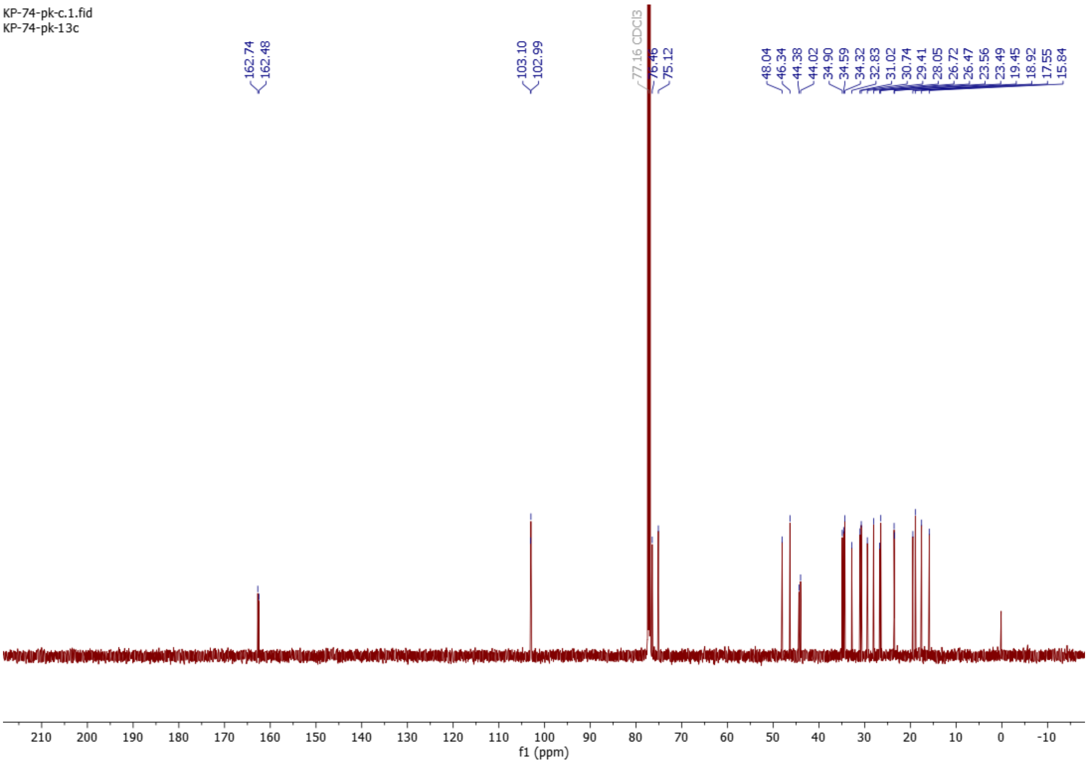

1-((*R*)-2,2-Dimethyl-3-methylenecyclopentyl)but-3-yn-2-ol (11d)

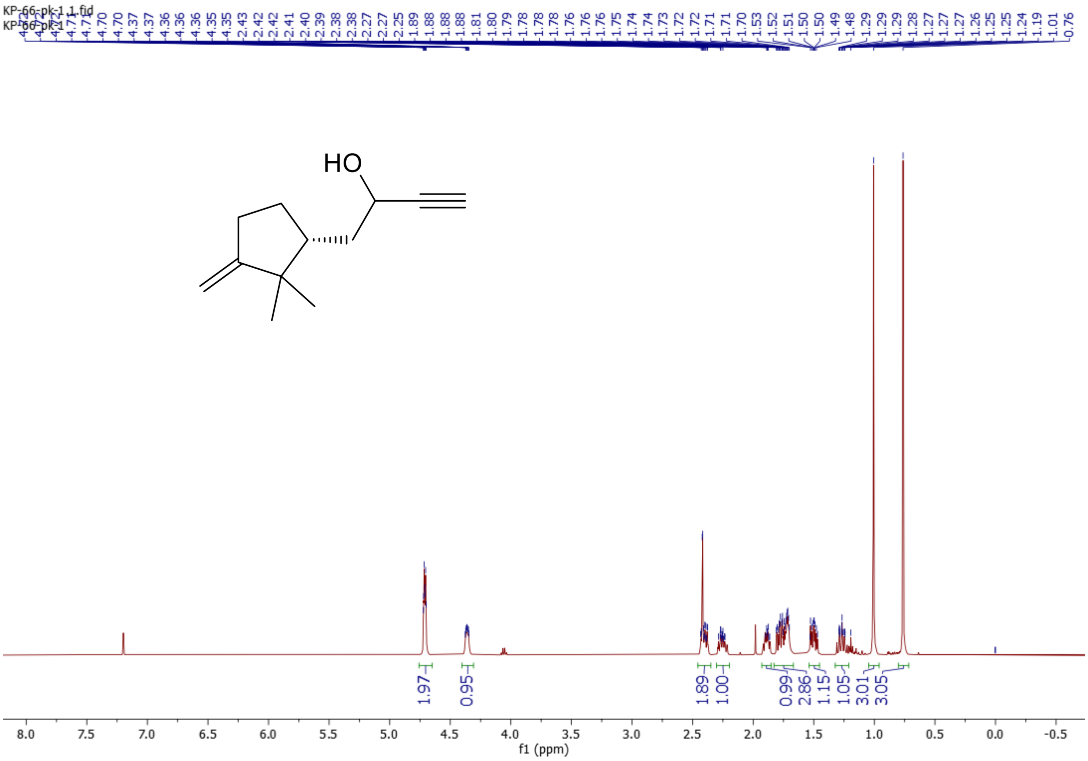

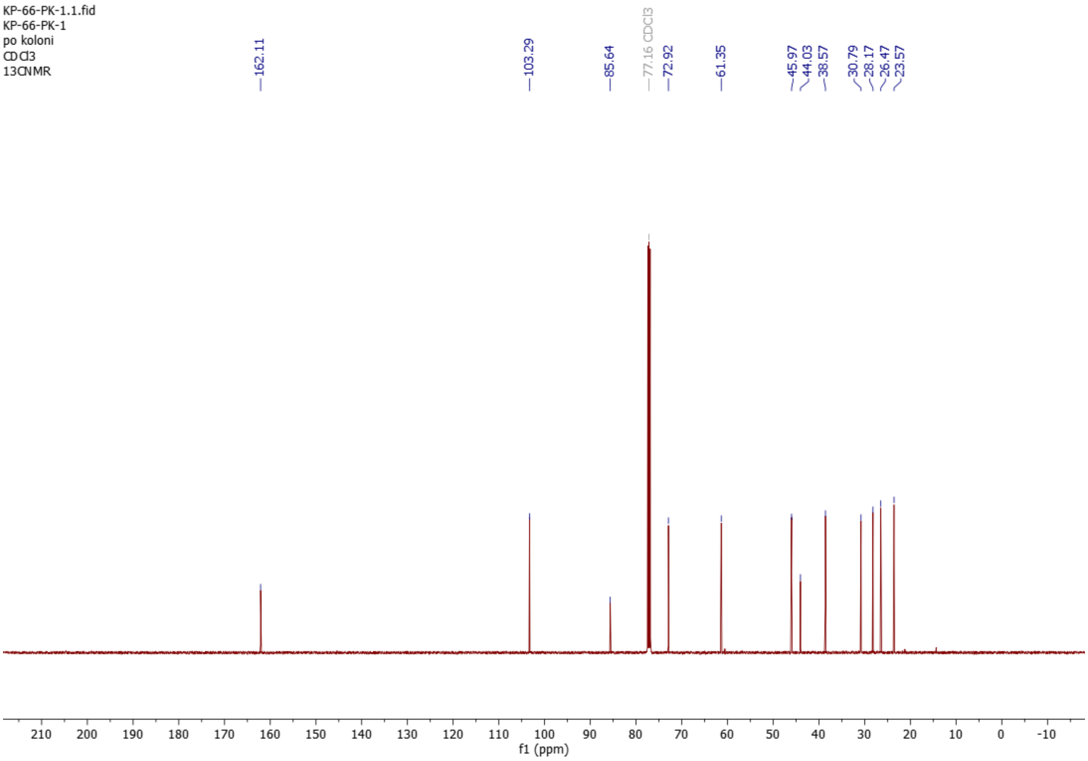

1-((*R*)-2,2-Dimethyl-3-methylenecyclopentyl)but-3-yn-2-ol (11d’)

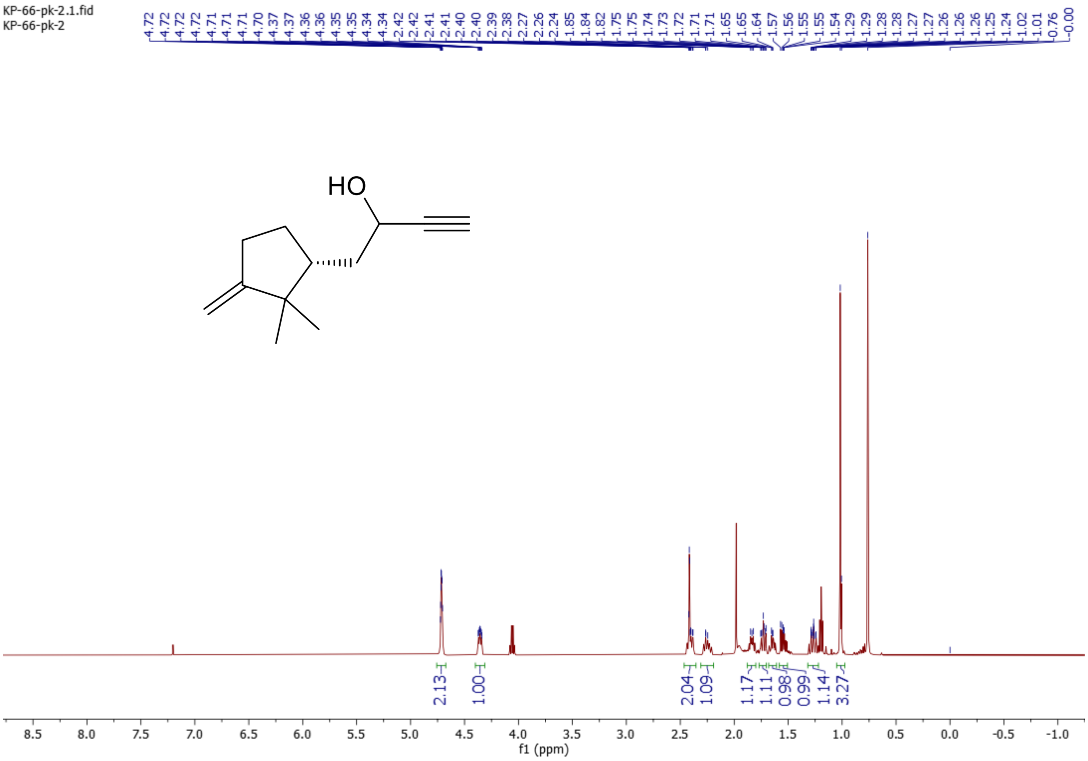

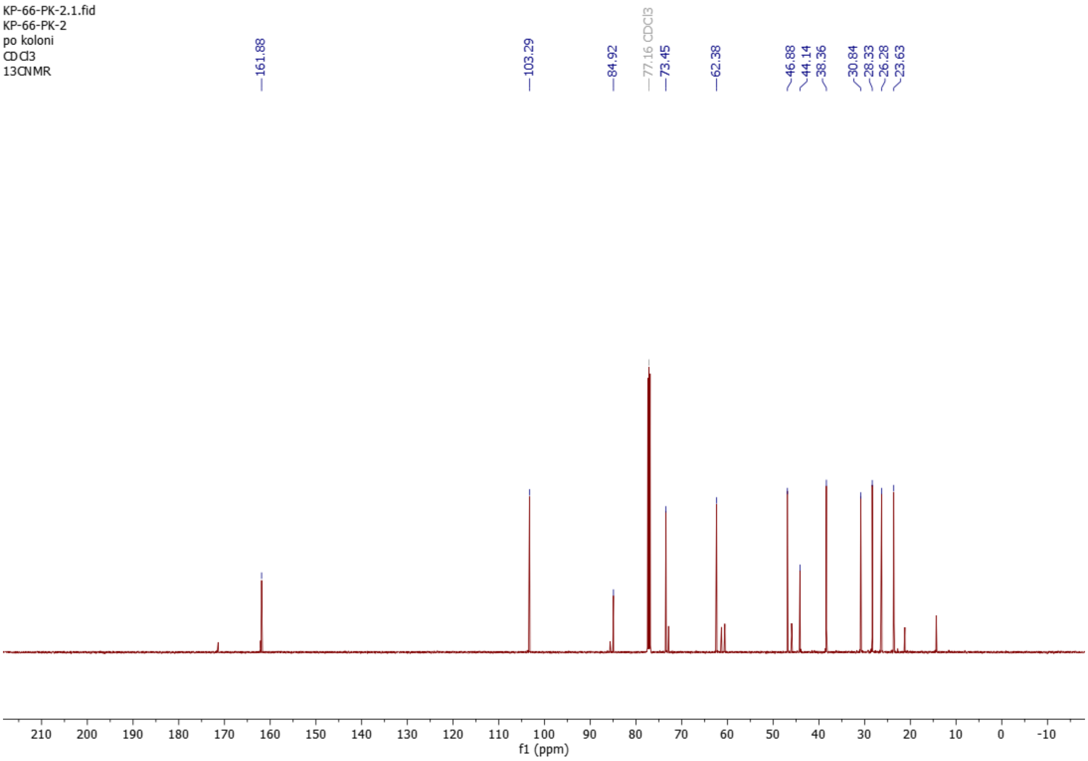

(*R*)-1-(2,2-Dimethyl-3-methylenecyclopentyl)-2-methylpropan-2-ol (12a)

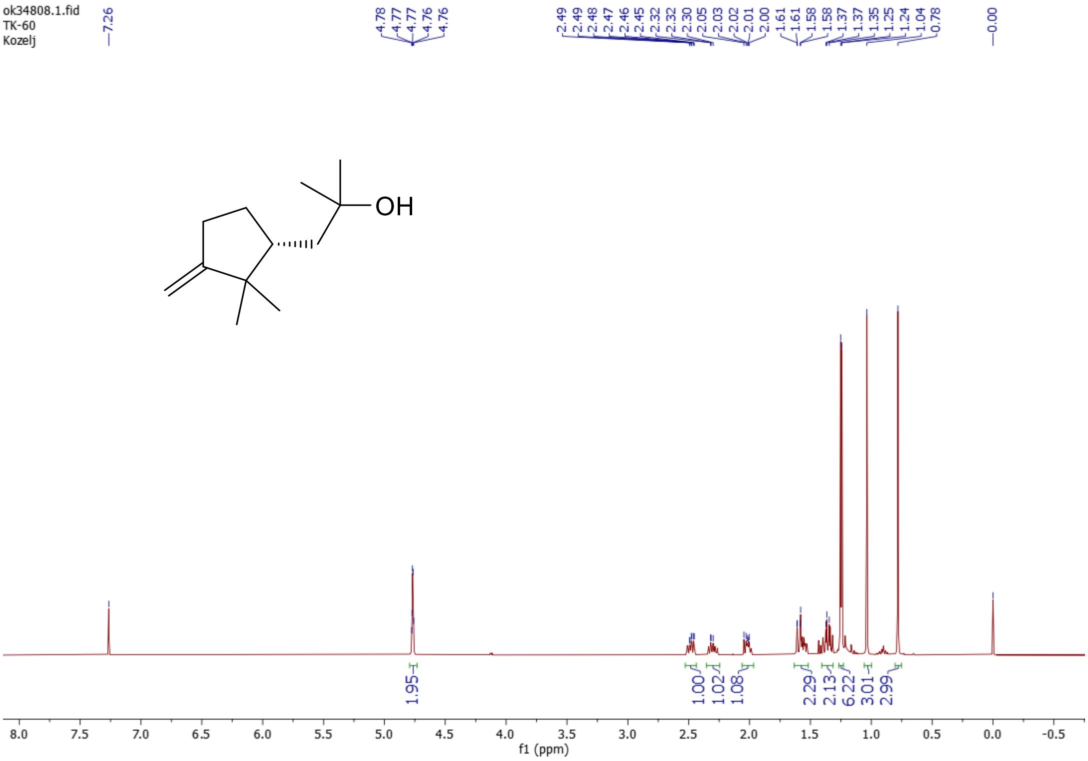

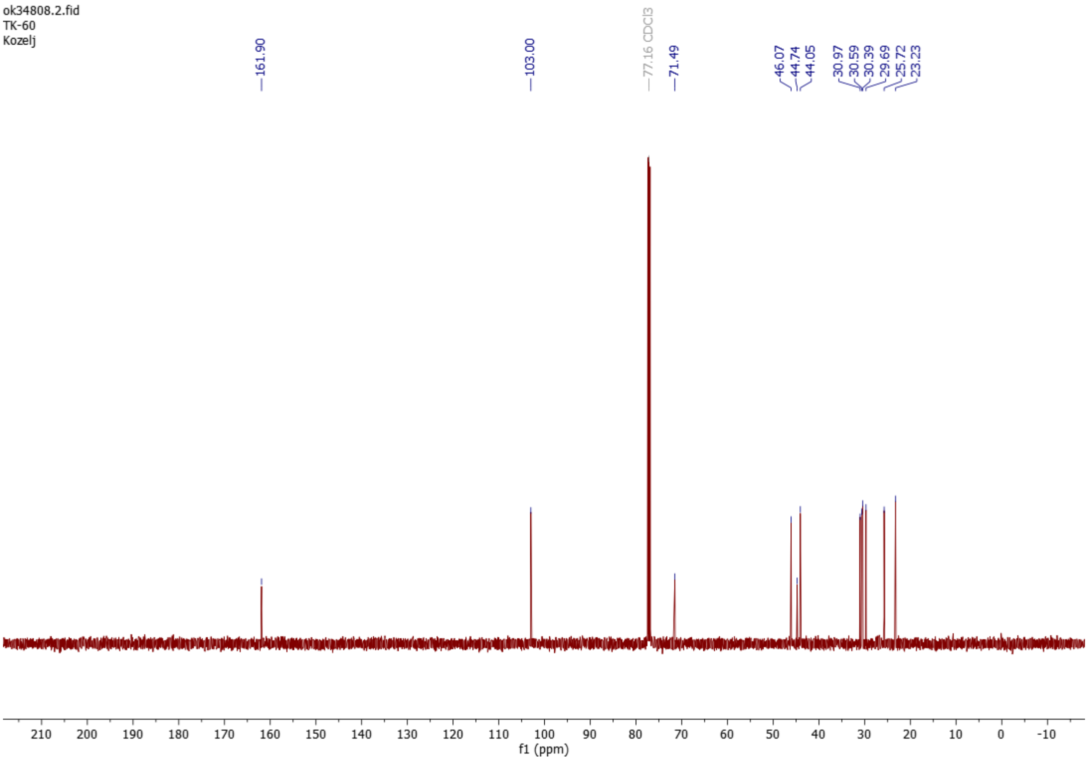

1-((*R*)-2,2-Dimethyl-3-methylenecyclopentyl)-2-methylbutan-2-ol (12b/12b’)

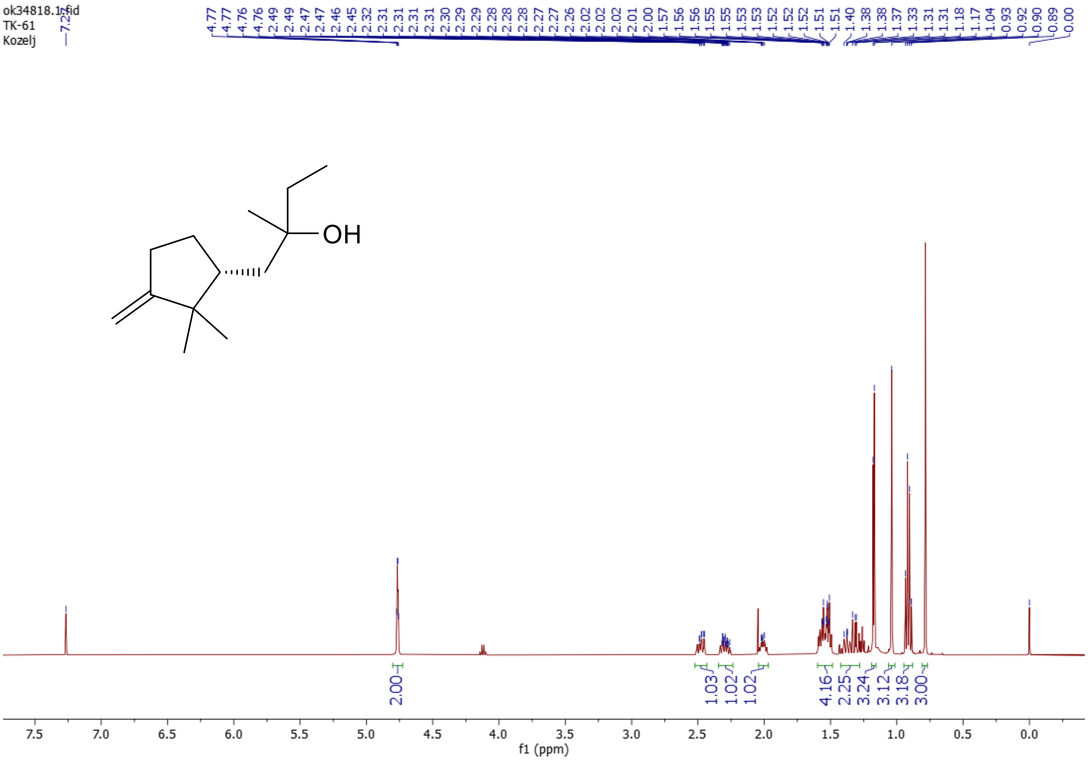

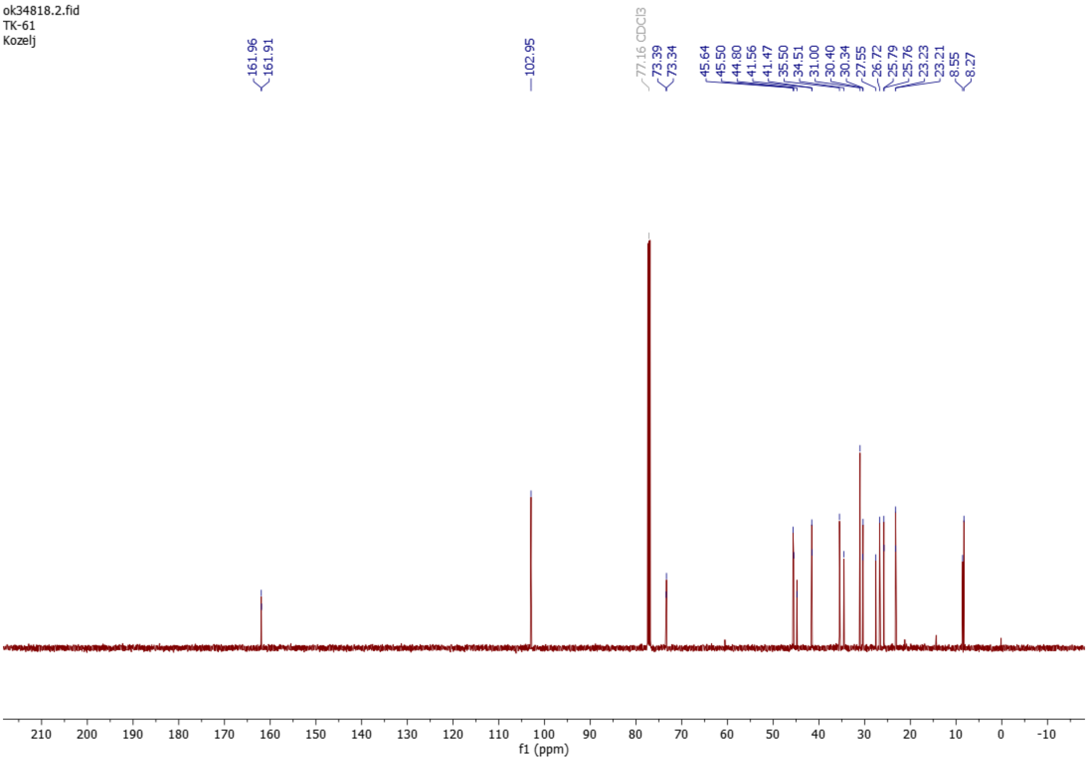

1-((*R*)-2,2-Dimethyl-3-methylenecyclopentyl)-2-methylpentan-2-ol (12c/12c')

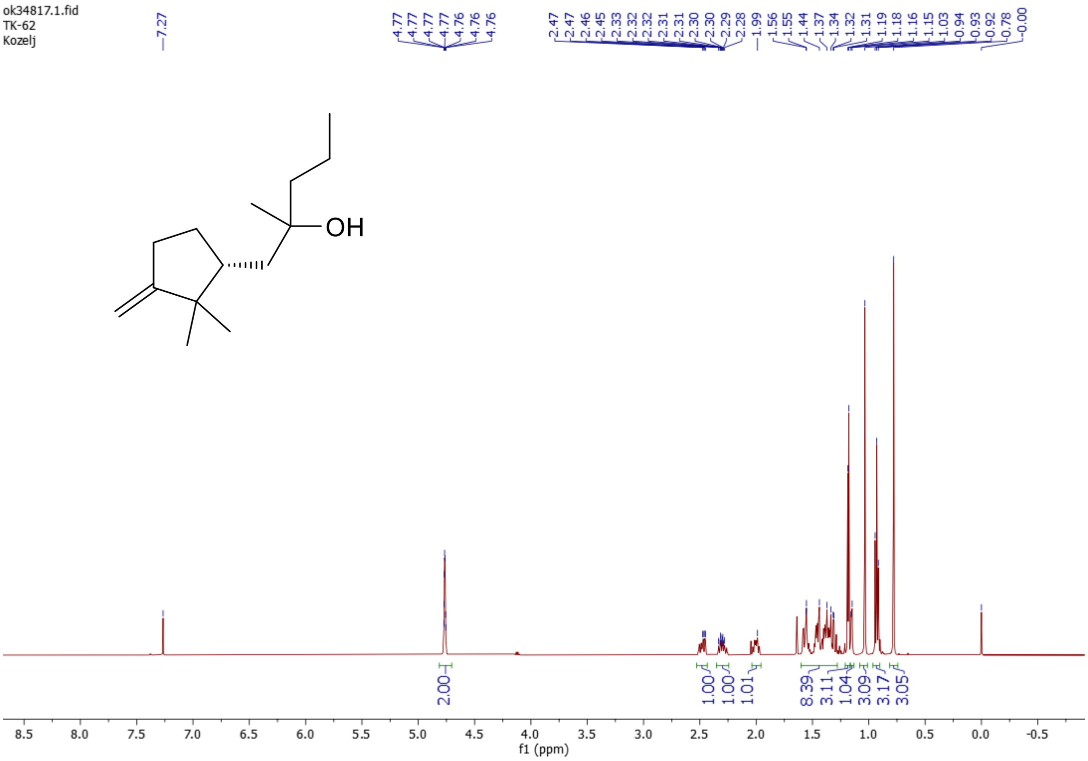

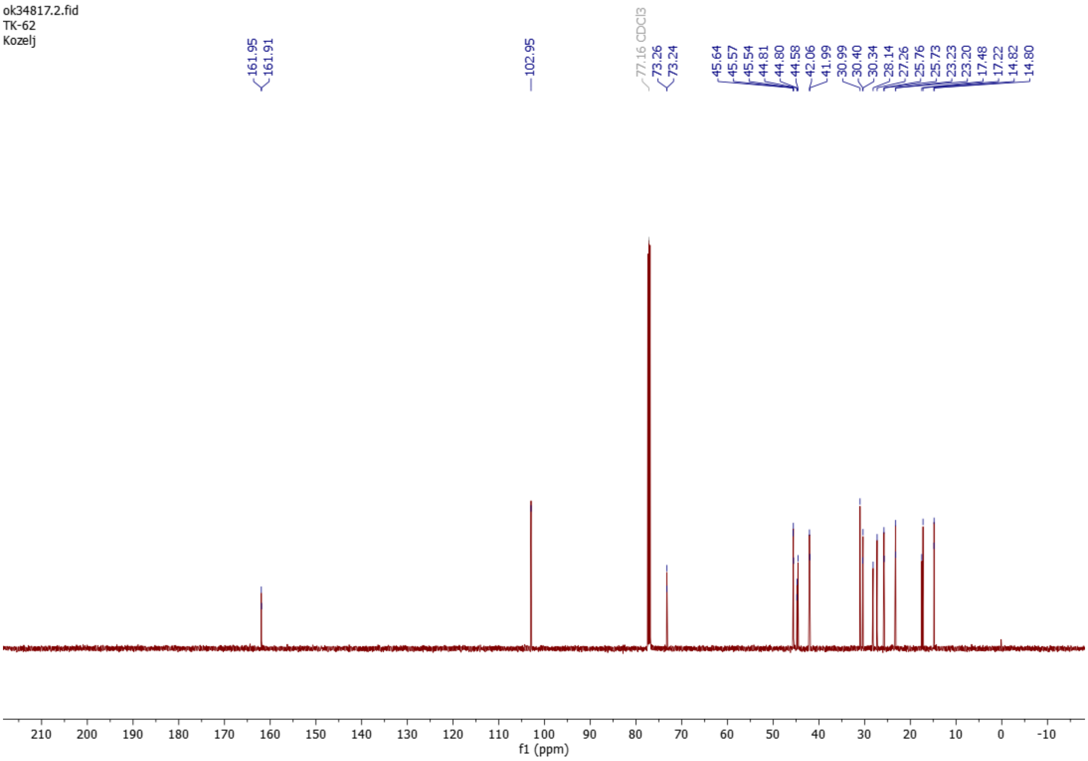

1-((*R*)-2,2-Dimethyl-3-methylenecyclopentyl)-2-methylhexan-2-ol (12d/12d')

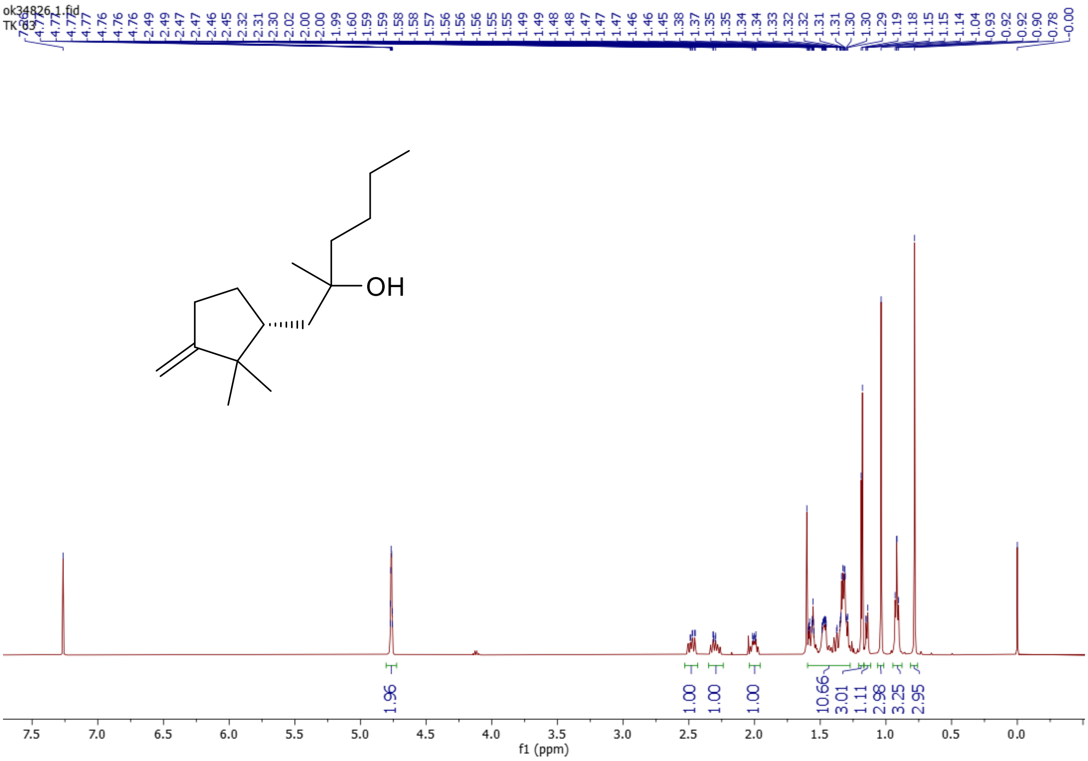

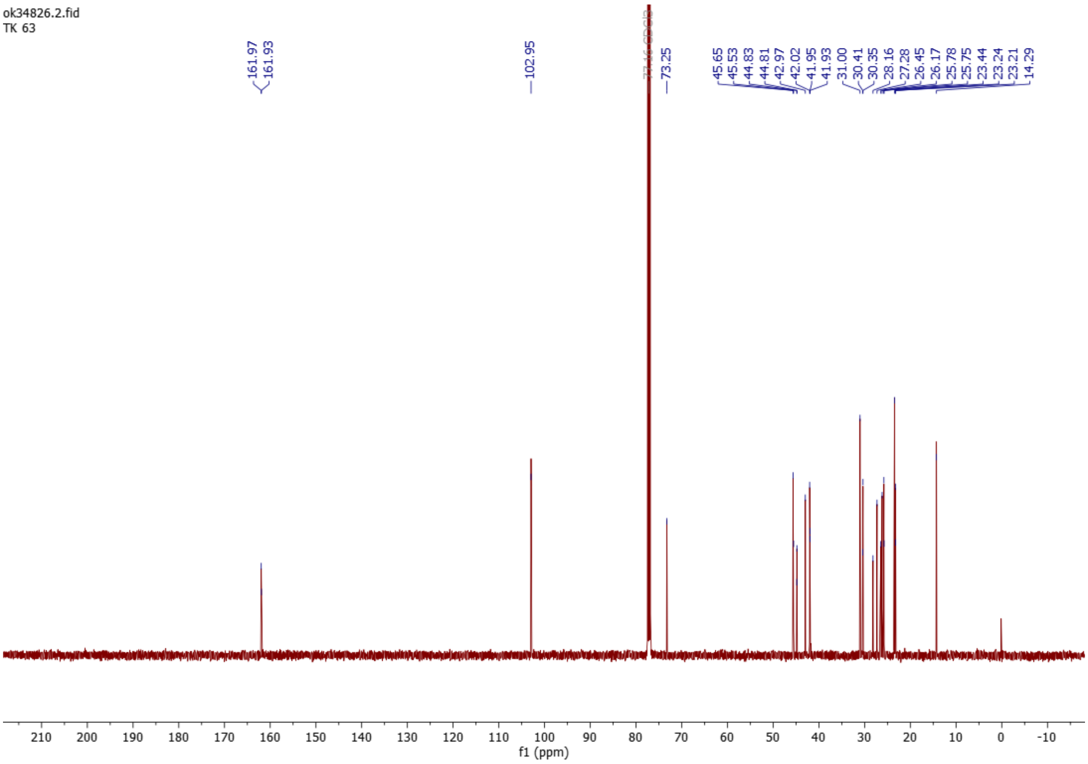

1-((*R*)-2,2-Dimethyl-3-methylenecyclopentyl)-2-methylpent-4-en-2-ol (12e/12e')

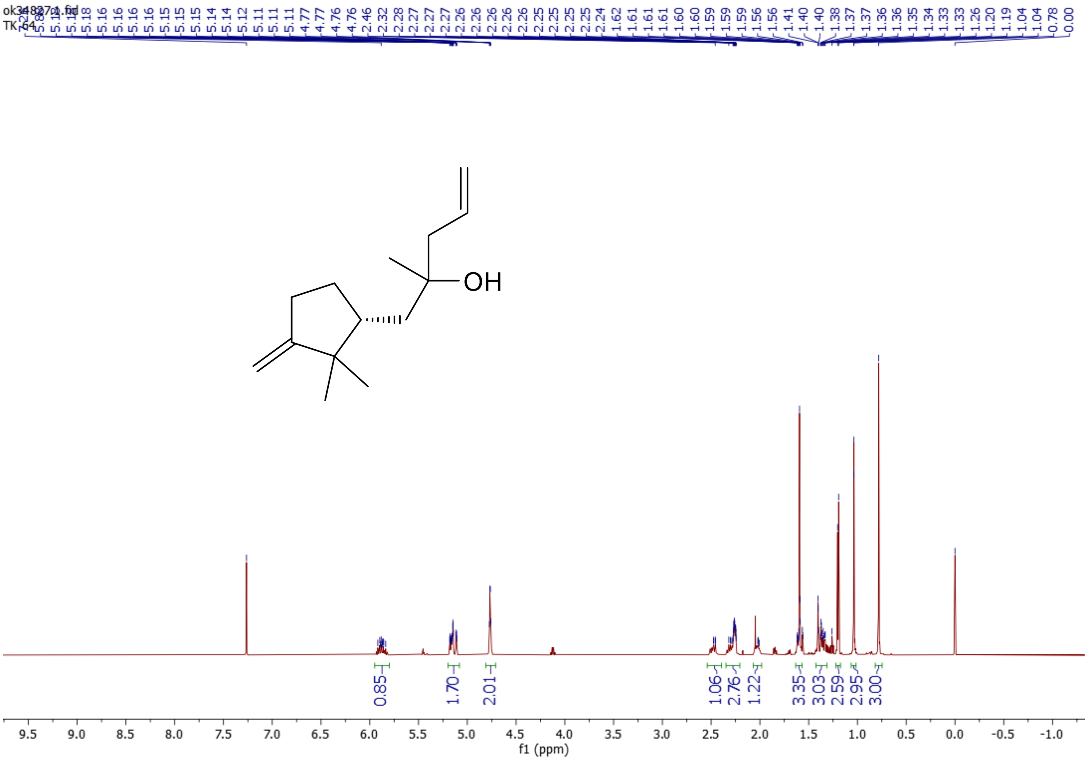

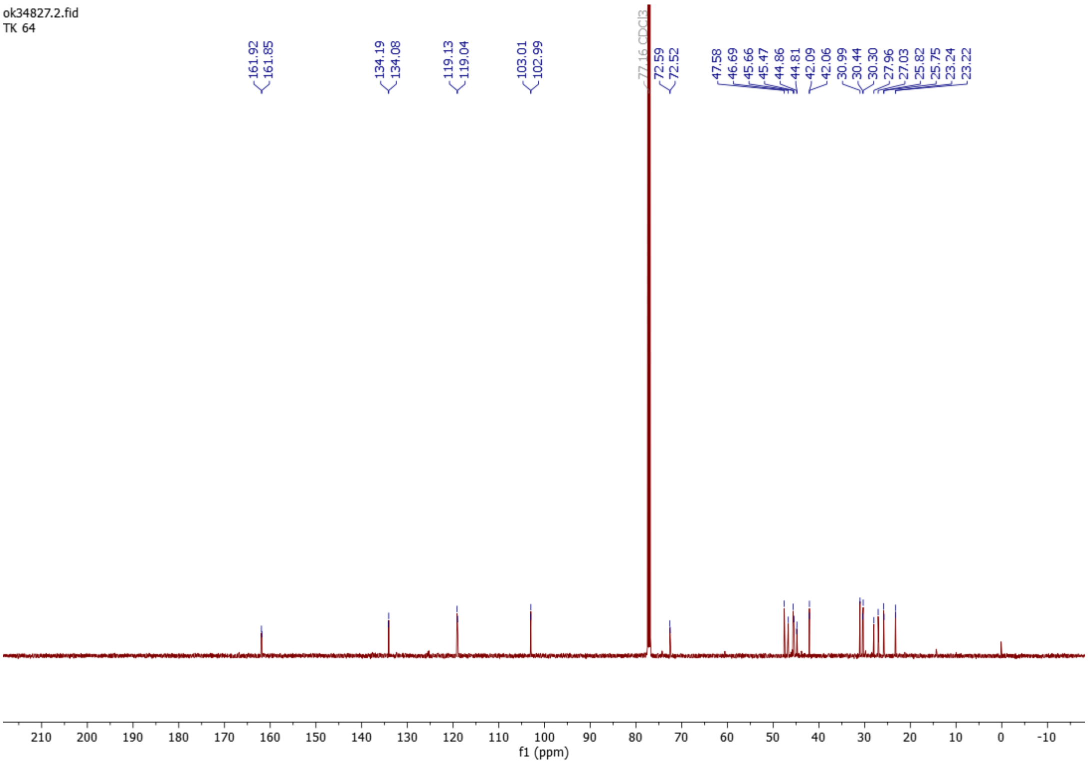

1-((*R*)-2,2-Dimethyl-3-methylenecyclopentyl)-2-phenylpropan-2-ol (12f/12f')

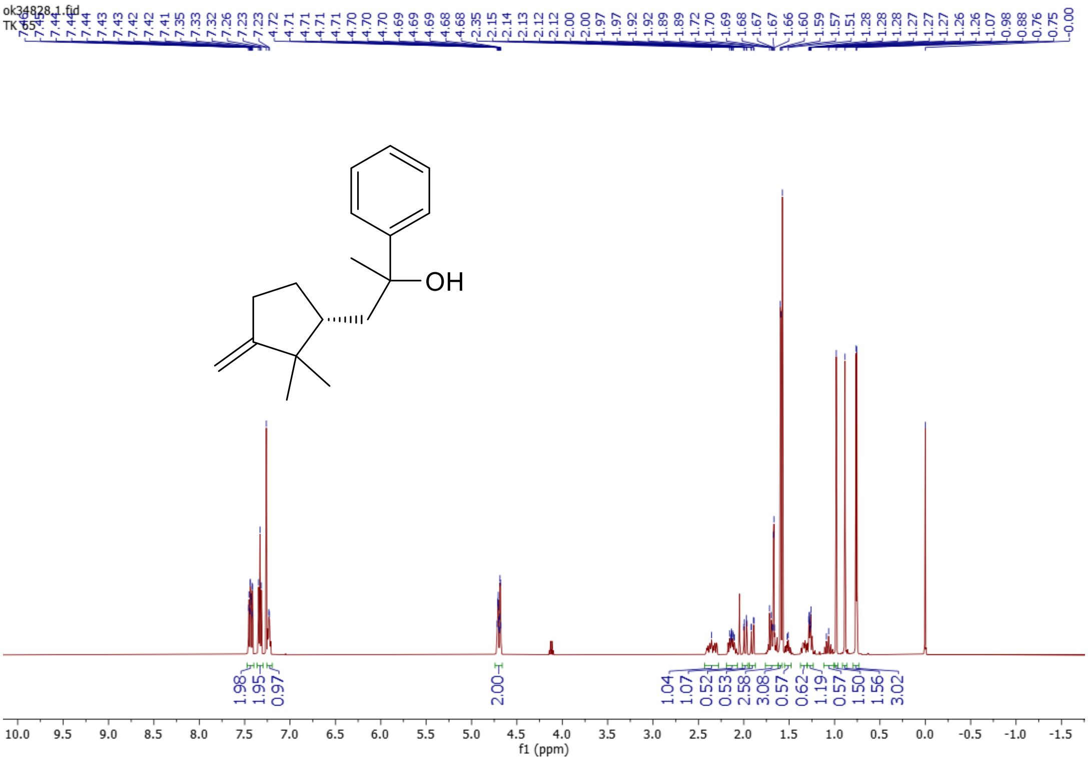

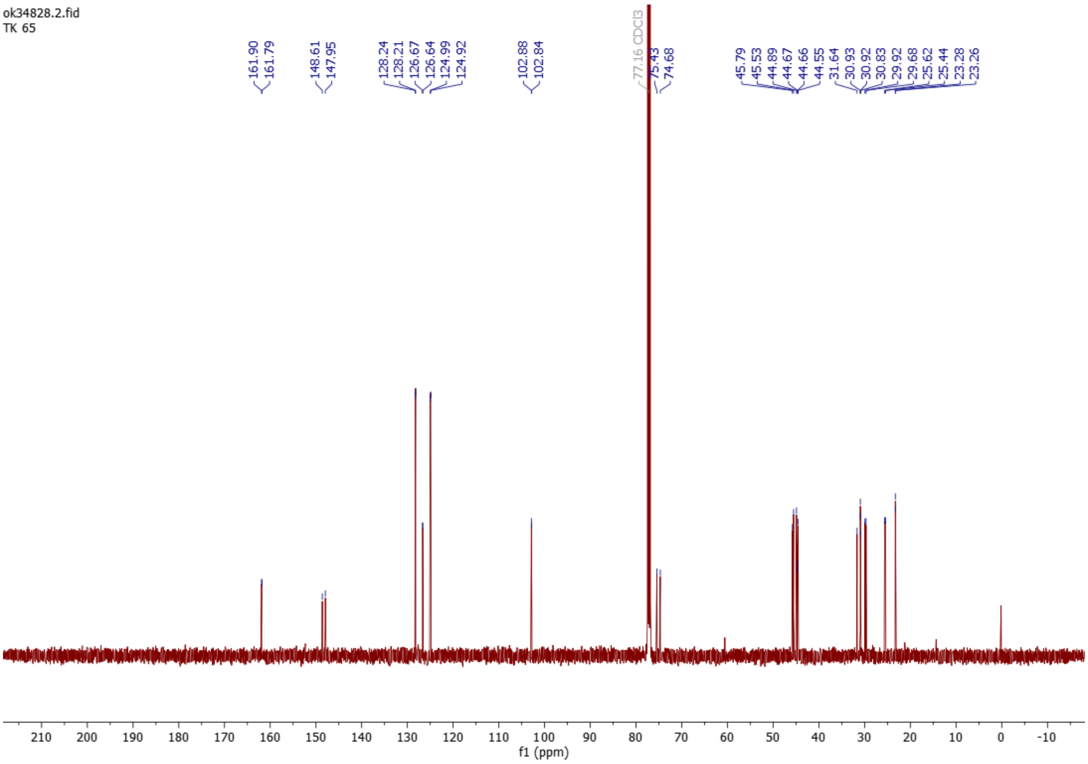

Ethyl (*R*,*E*)-4-(2,2-Dimethyl-3-methylenecyclopentyl)but-2-enoate (13)

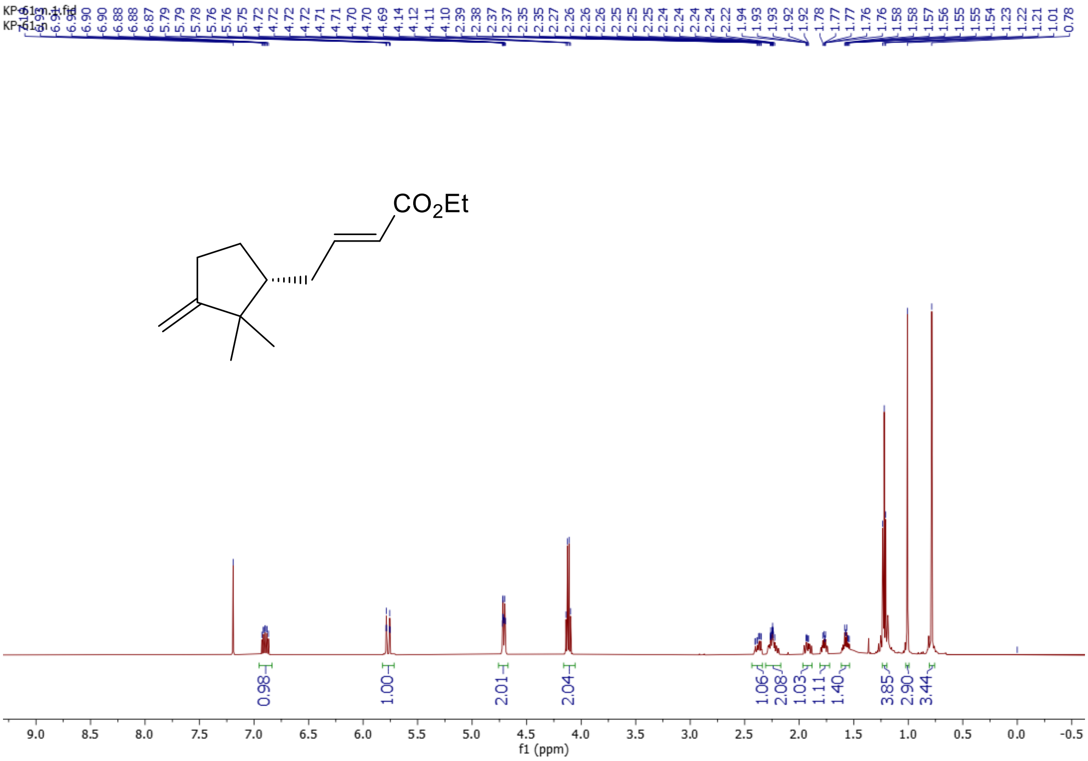

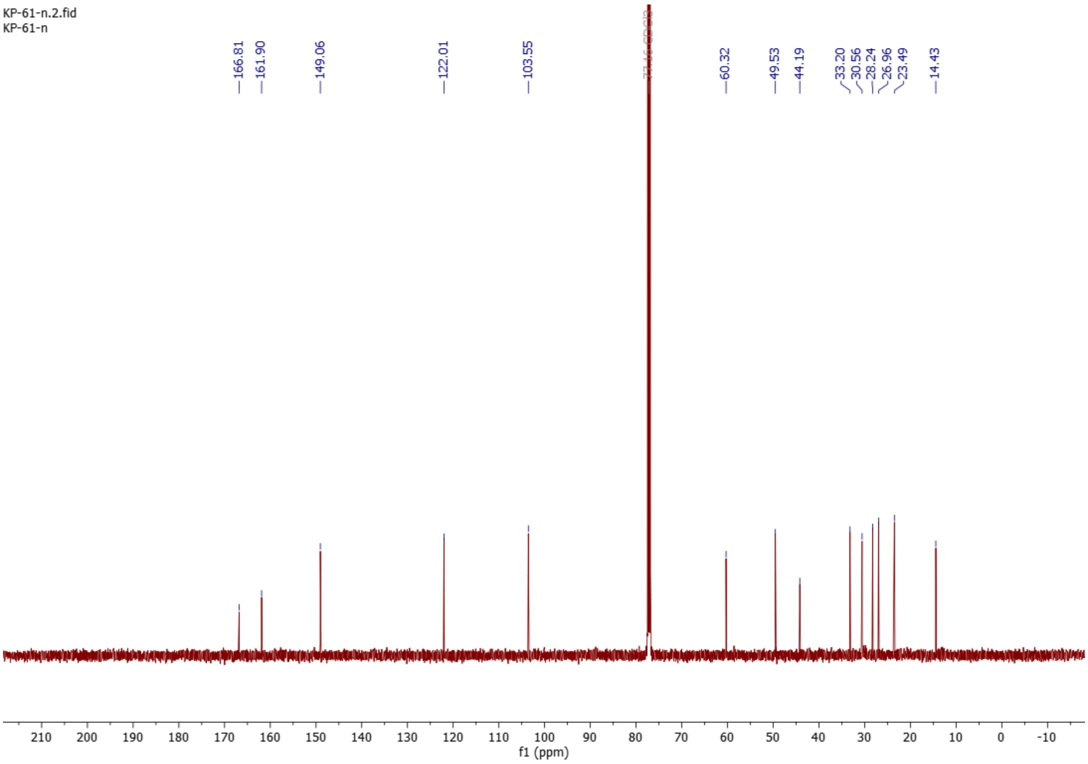

Methyl (*R*,*E*)-4-(2,2-dimethyl-3-methylenecyclopentyl)but-2-enoate (14)

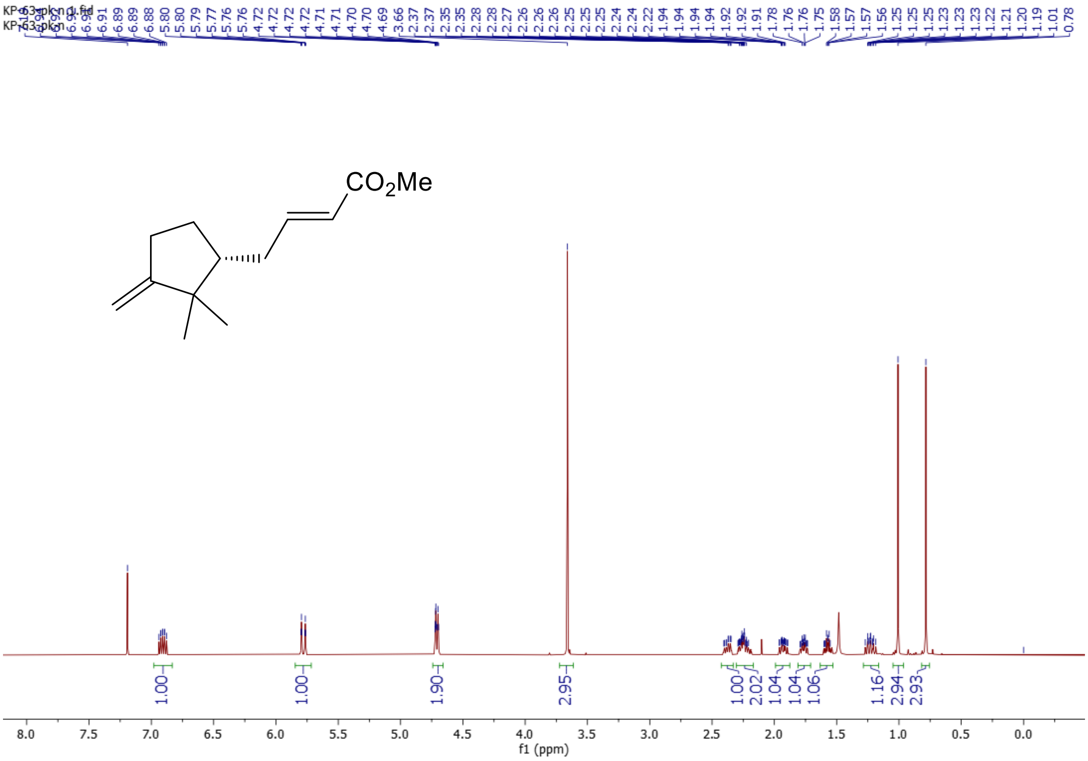

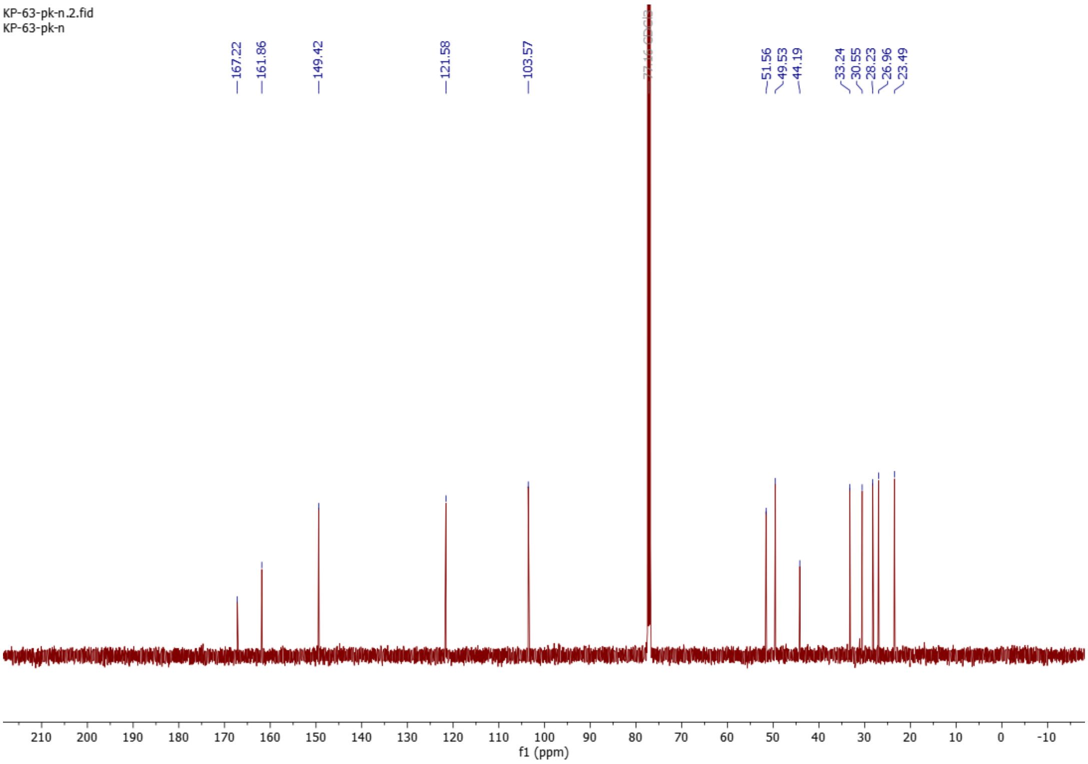

(*R*,*E*)-5-(2,2-Dimethyl-3-methylenecyclopentyl)pent-3-en-2-one (15)

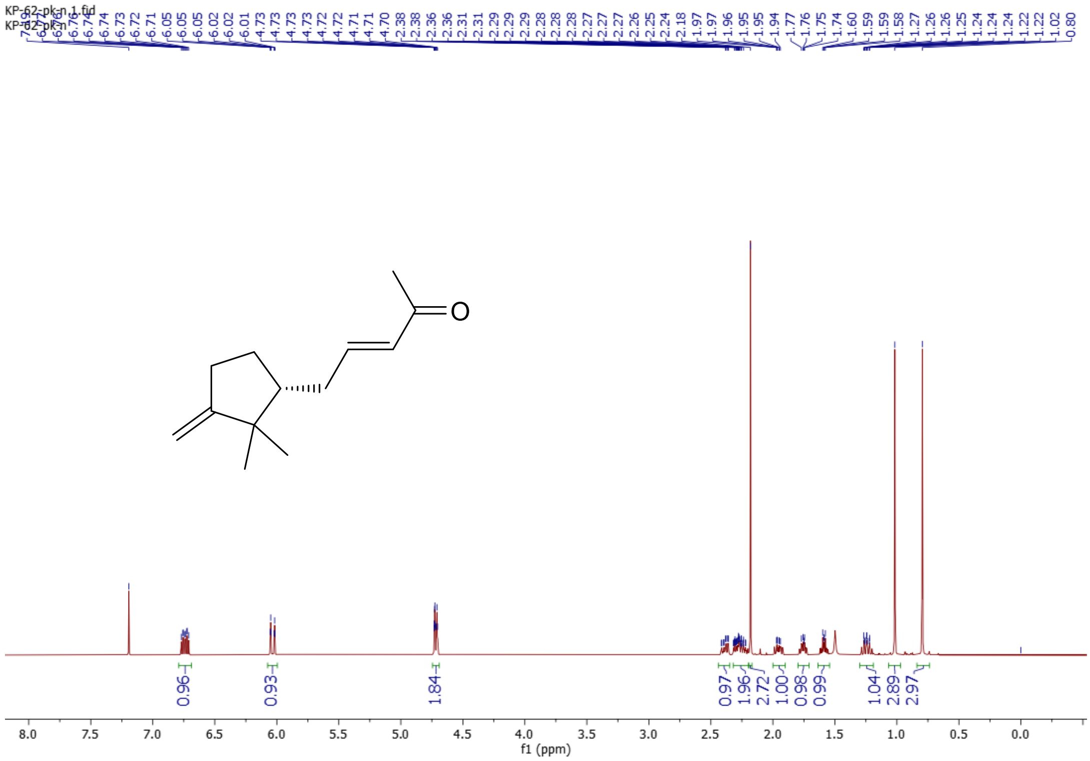

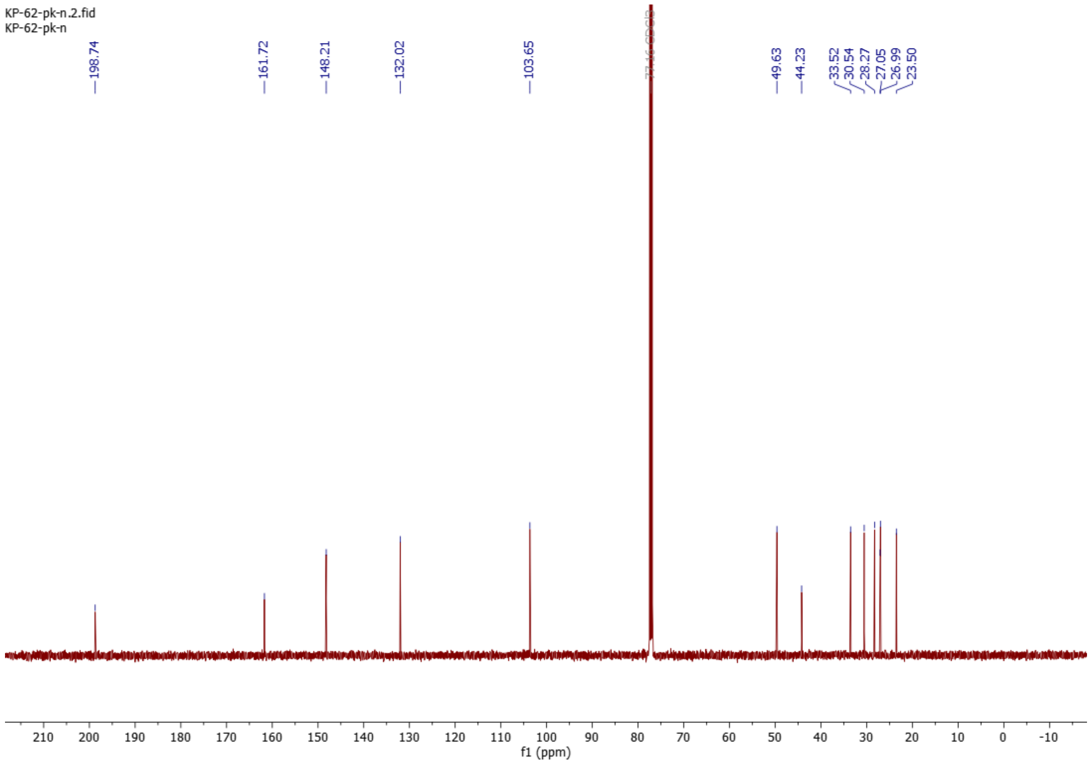

(*R*,*E*)-(3-(2,2-Dimethyl-3-methylenecyclopentyl)prop-1-en-1-yl)benzene (16) and (*R*,*Z*)-(3-(2,2-dimethyl-3-methylenecyclopentyl)prop-1-en-1-yl)benzene (16')

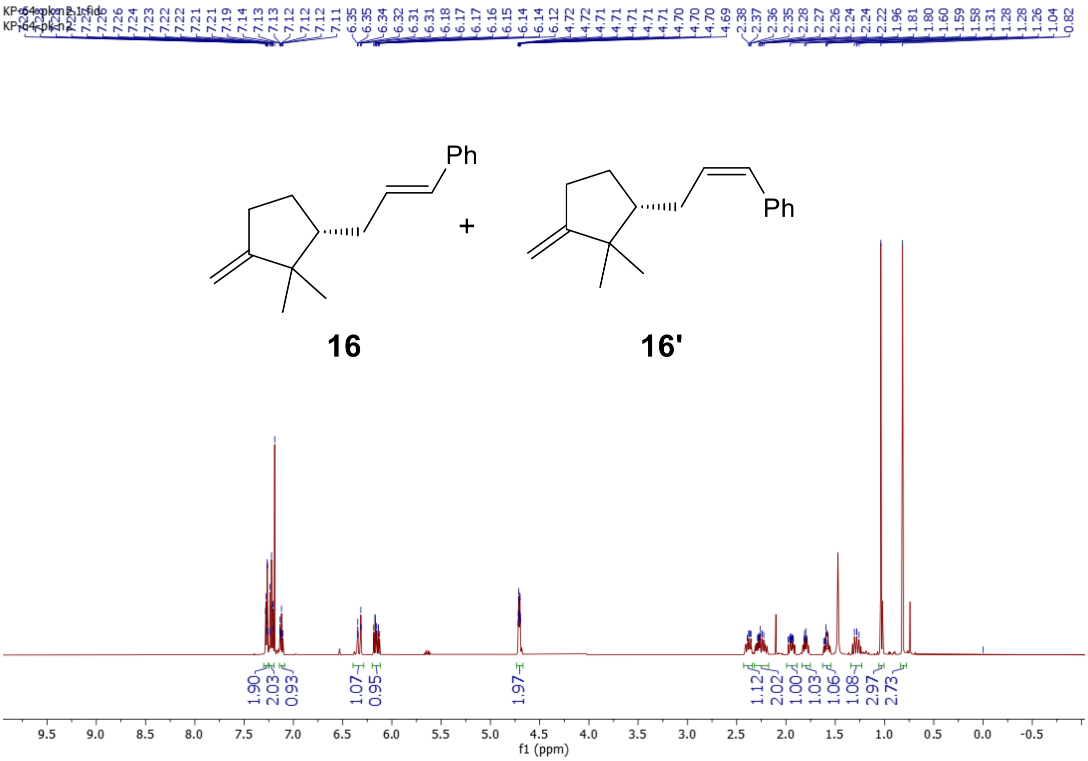

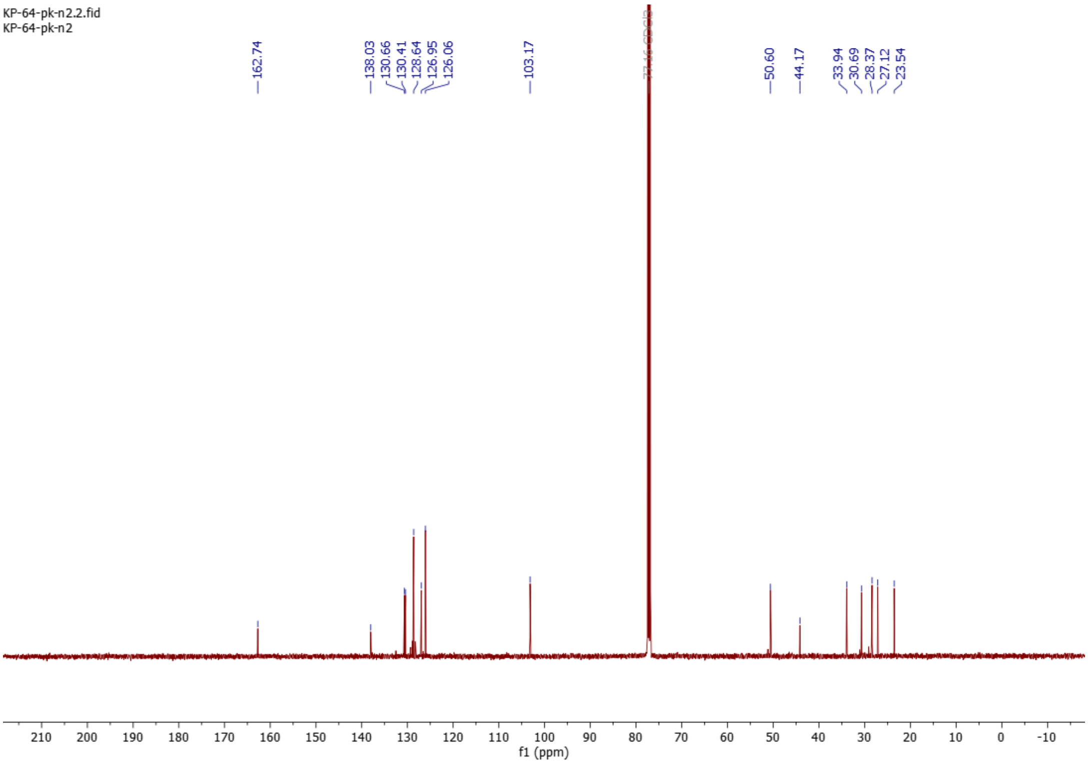

(*R*)-5-(2-methoxyethyl)-4,4-dimethylspiro[2.4]heptane (17)

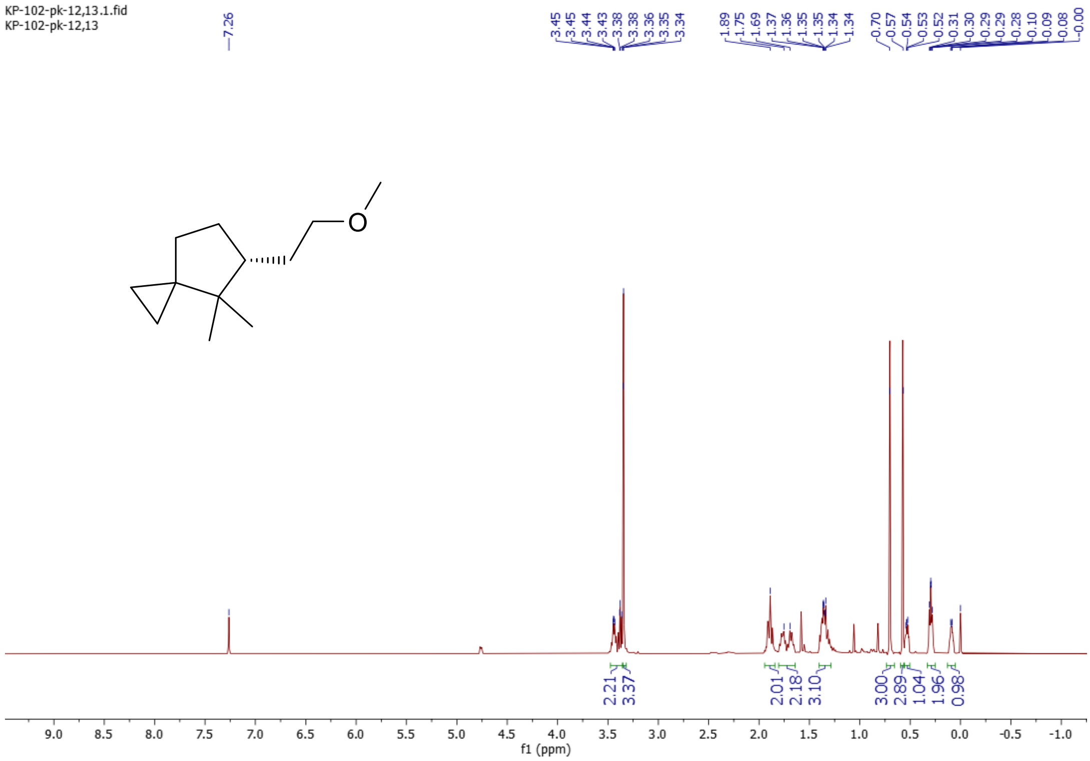

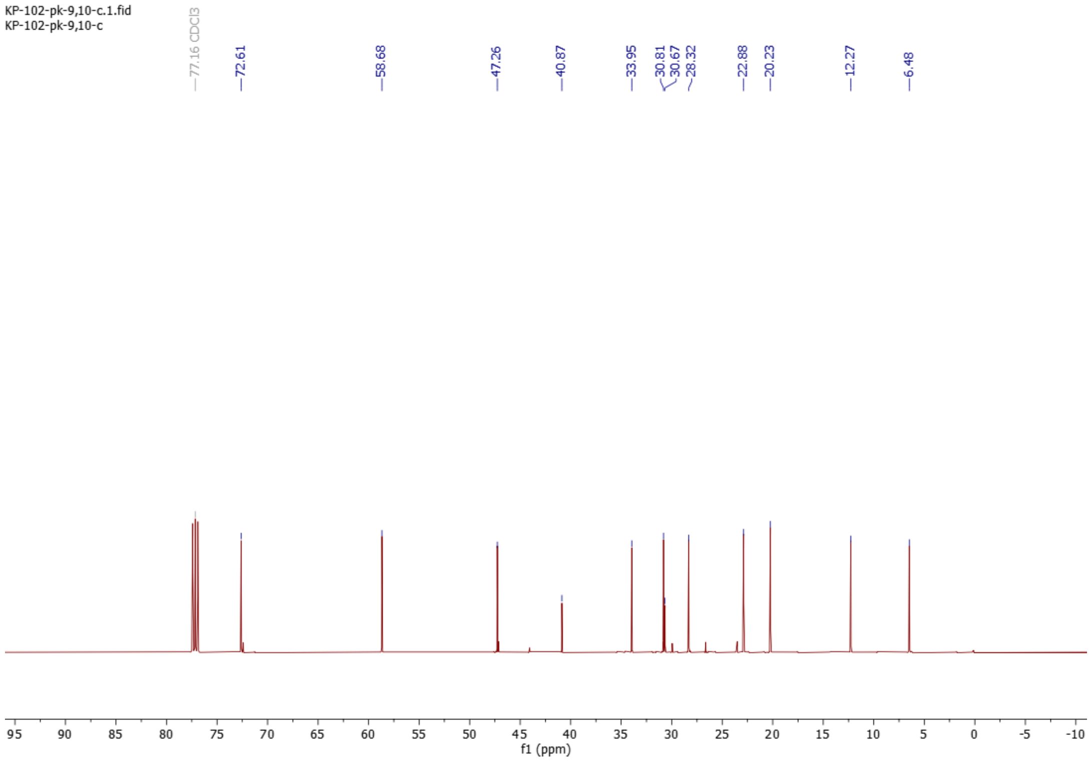

(*R*)-2-(4,4-Dimethylspiro[2.4]heptan-5-yl)-*N*-methoxy-*N*-methylacetamide (18)

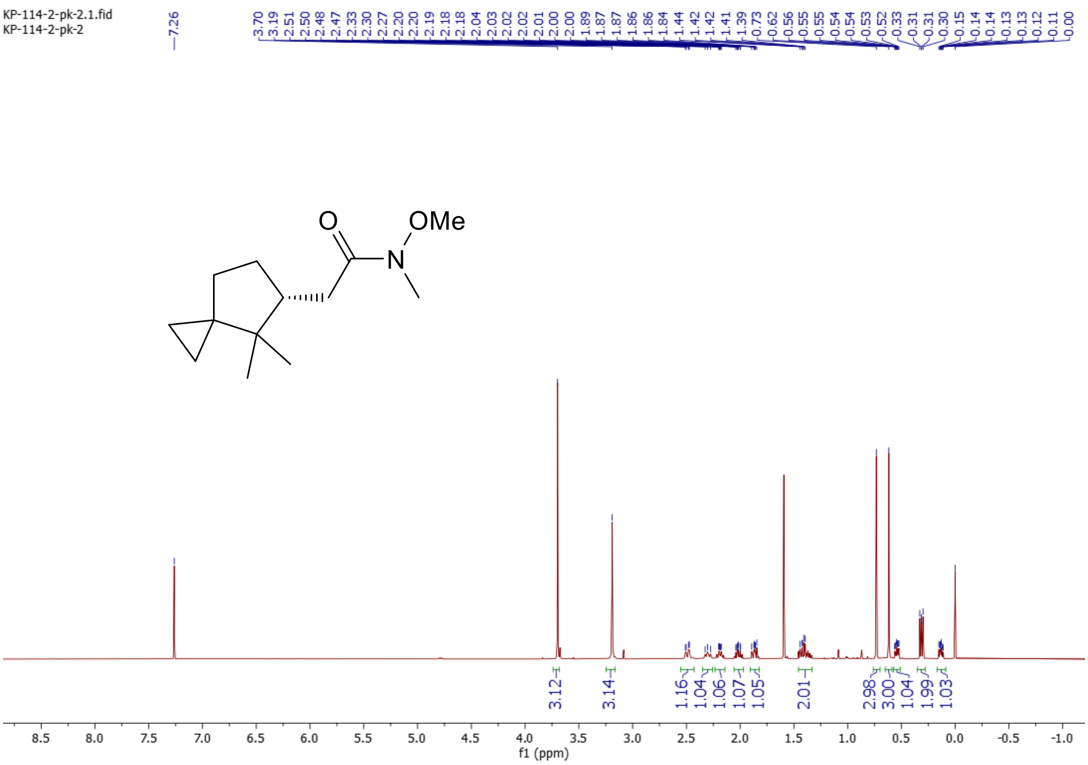

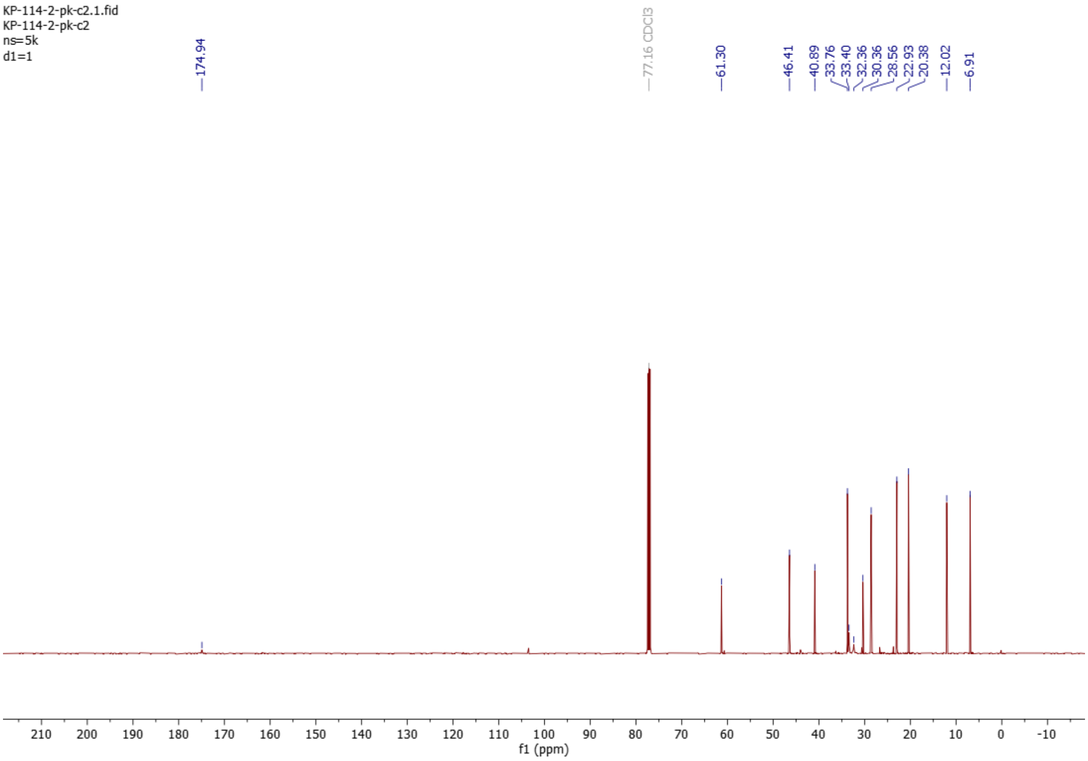

(*R*)-1-(4,4-Dimethylspiro[2.4]heptan-5-yl)propan-2-one (19a)

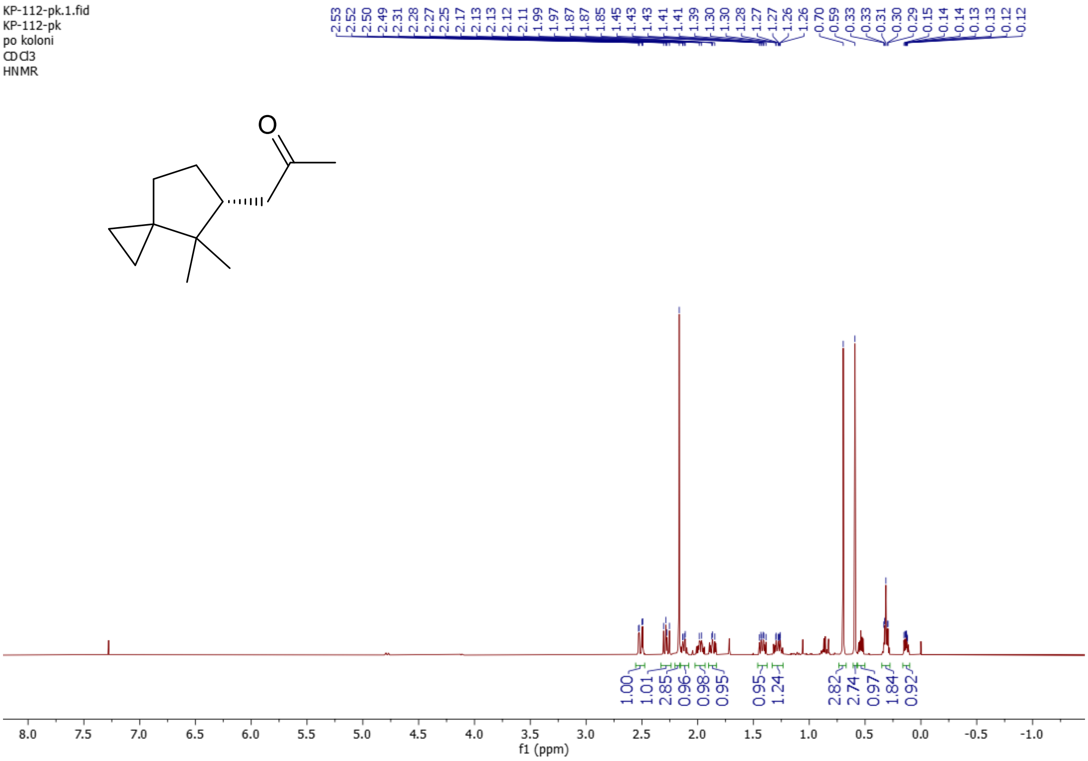

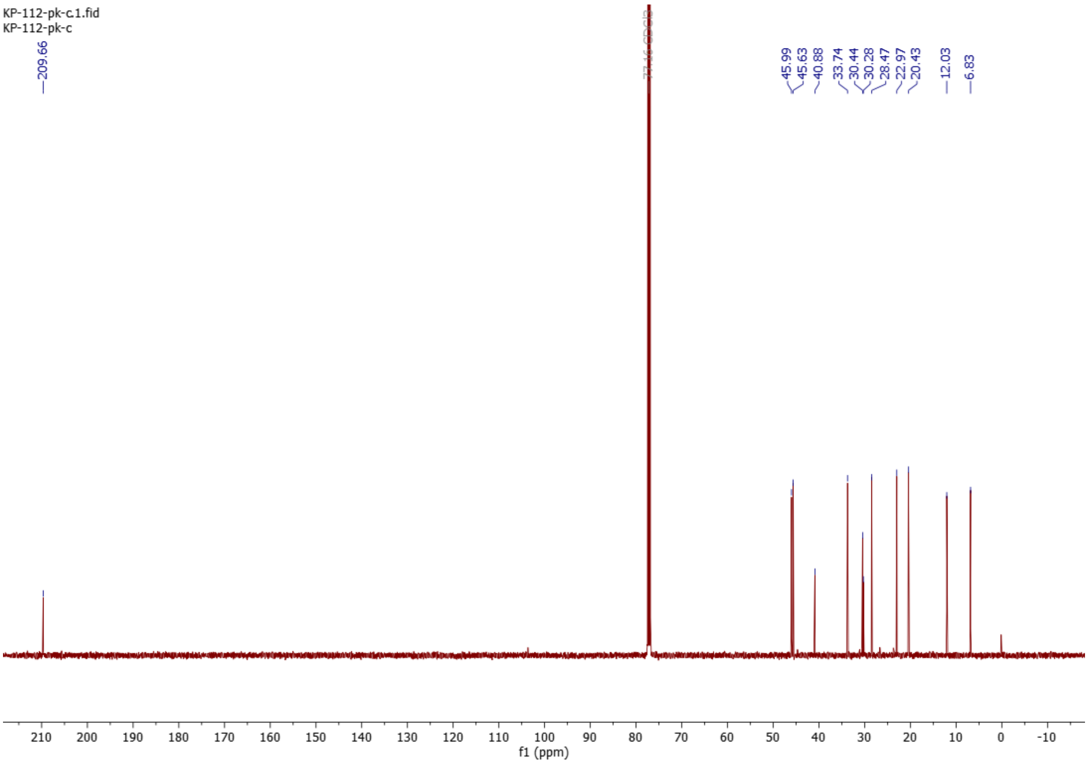

(*R*)-1-(4,4-Dimethylspiro[2.4]heptan-5-yl)pent-4-en-2-one (19b)

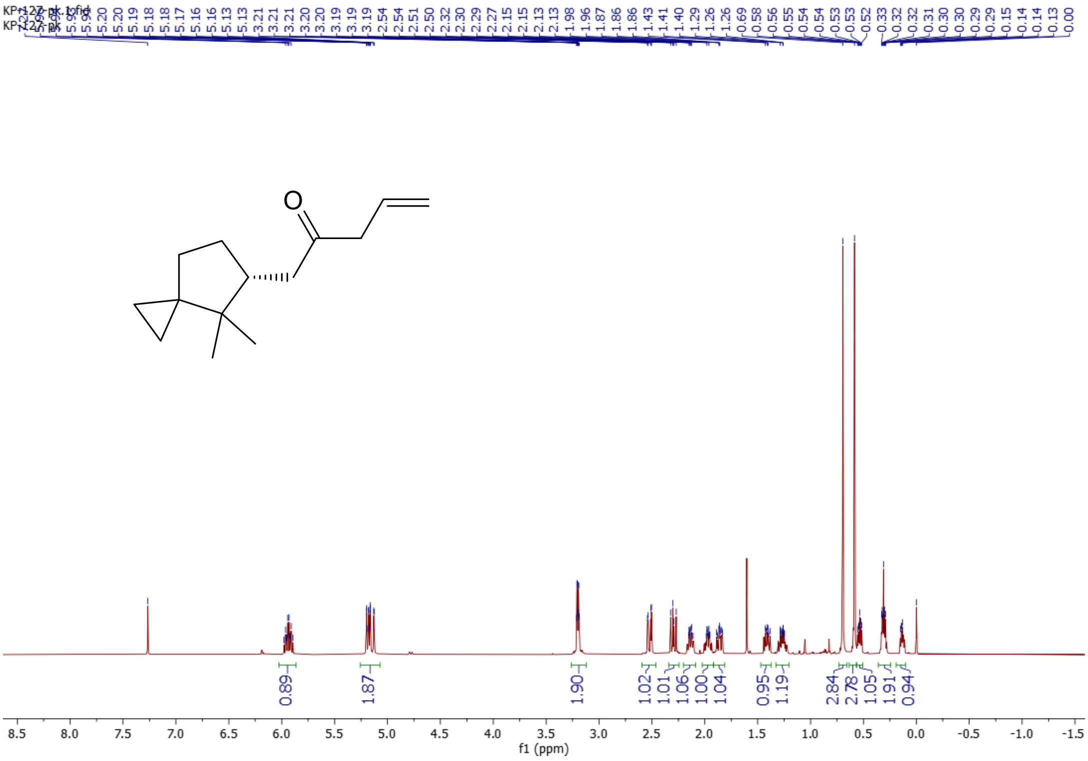

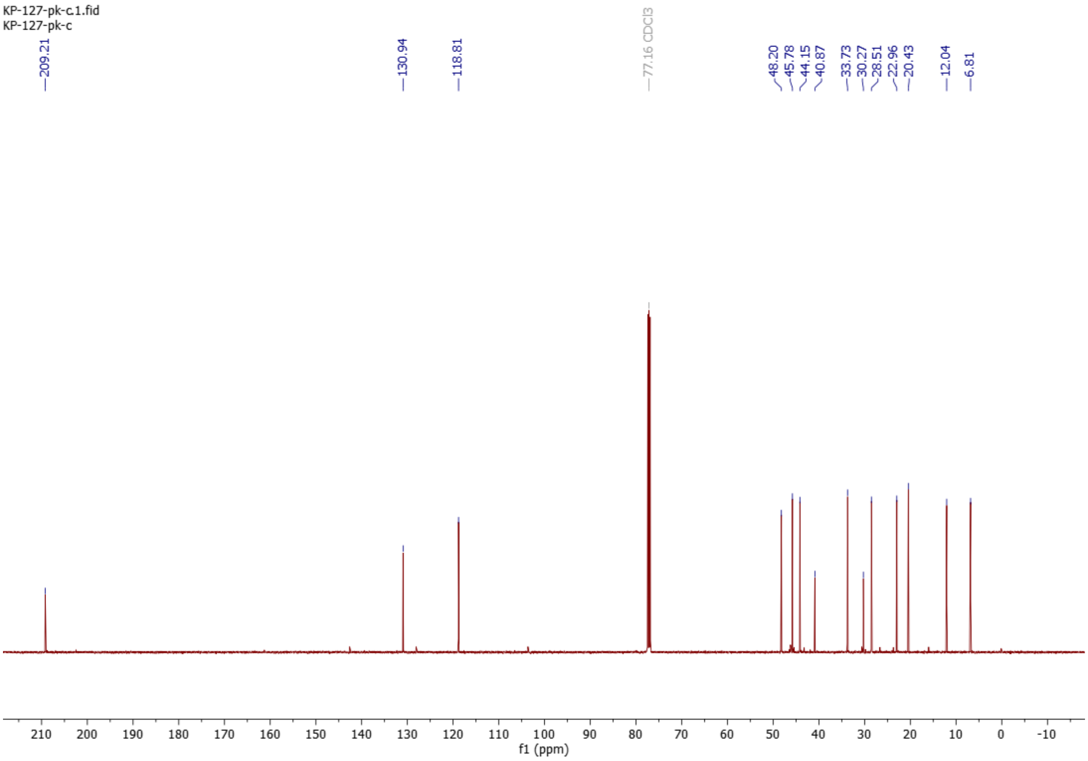

(*R*)-1-(4,4-Dimethylspiro[2.4]heptan-5-yl)but-3-en-2-one (19c)

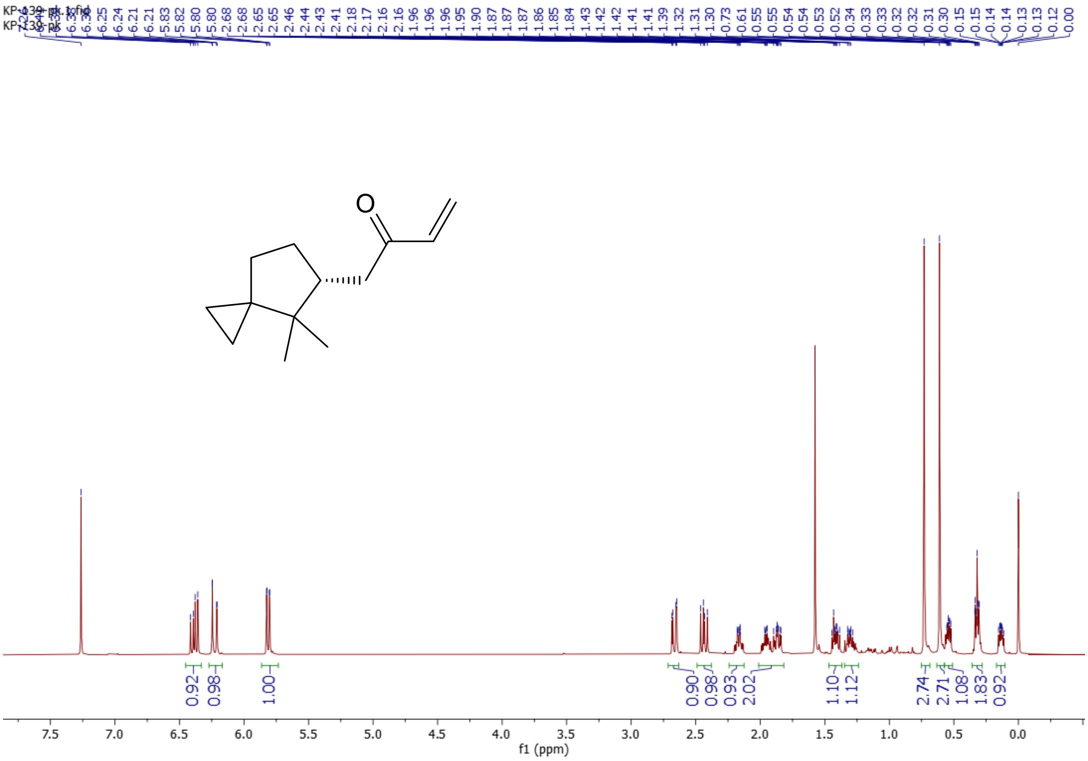

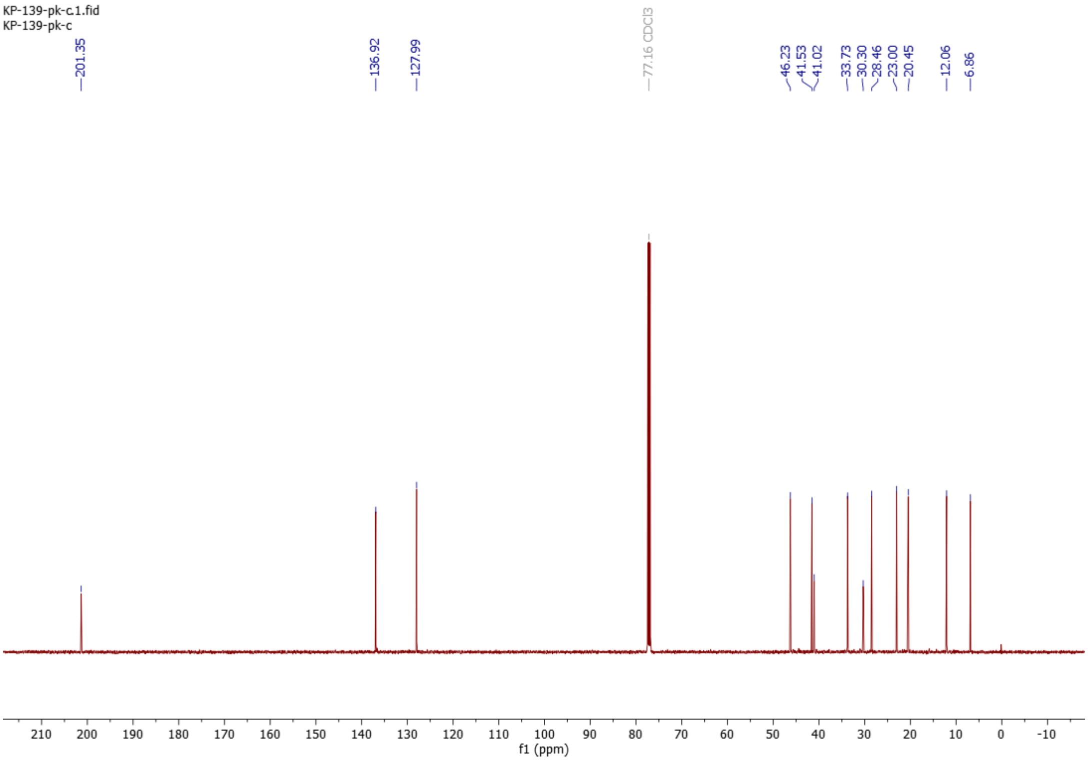

(*R*)-2-(4,4-Dimethylspiro[2.4]heptan-5-yl)-1-phenylethan-1-one (19d)

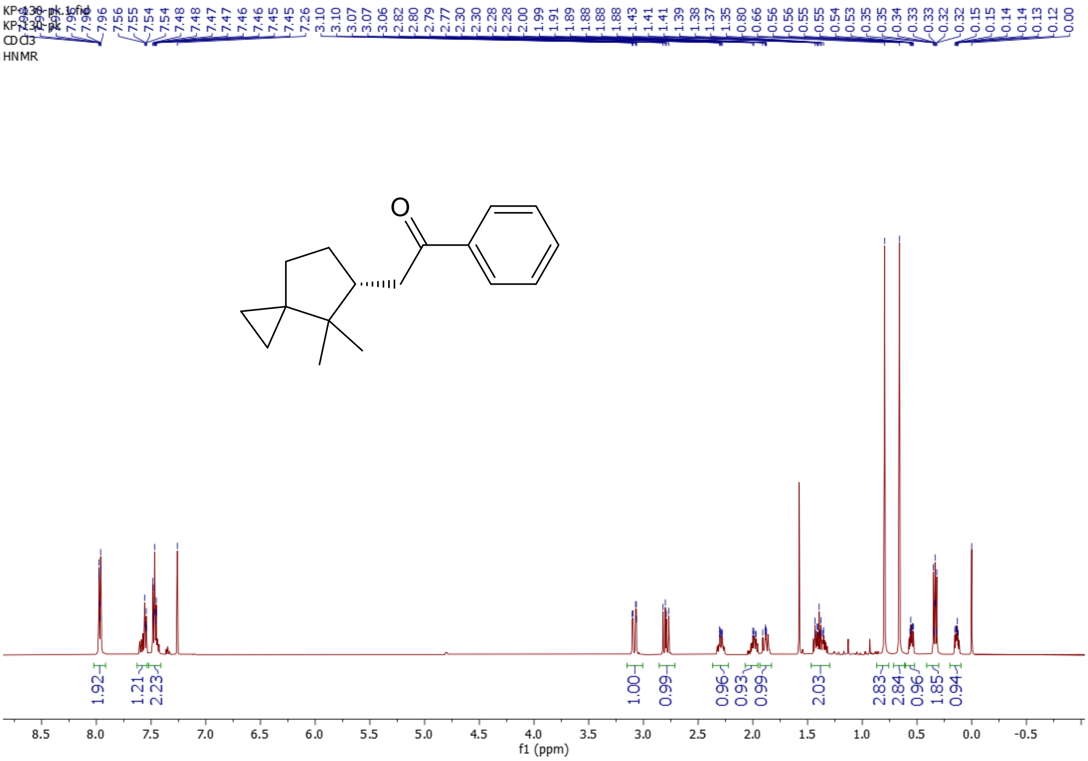

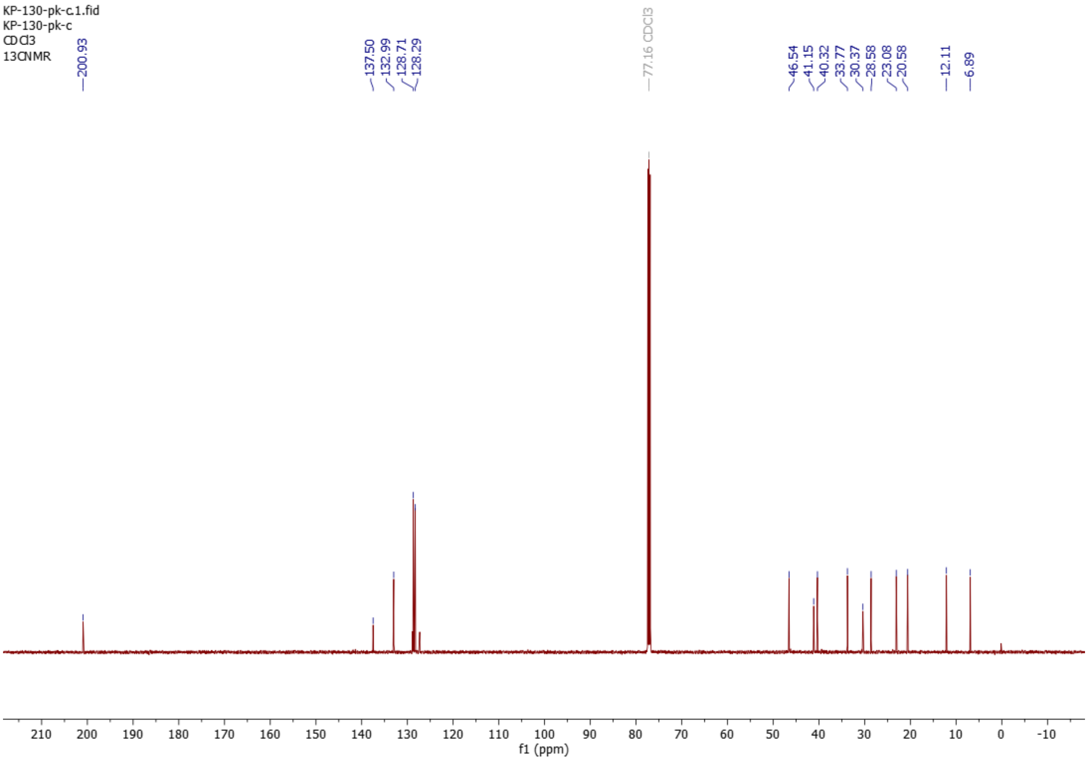

(1*R*,3*R*)-1-Methoxy-3-(2-methoxyethyl)-1,2,2-trimethylcyclopentane (20)

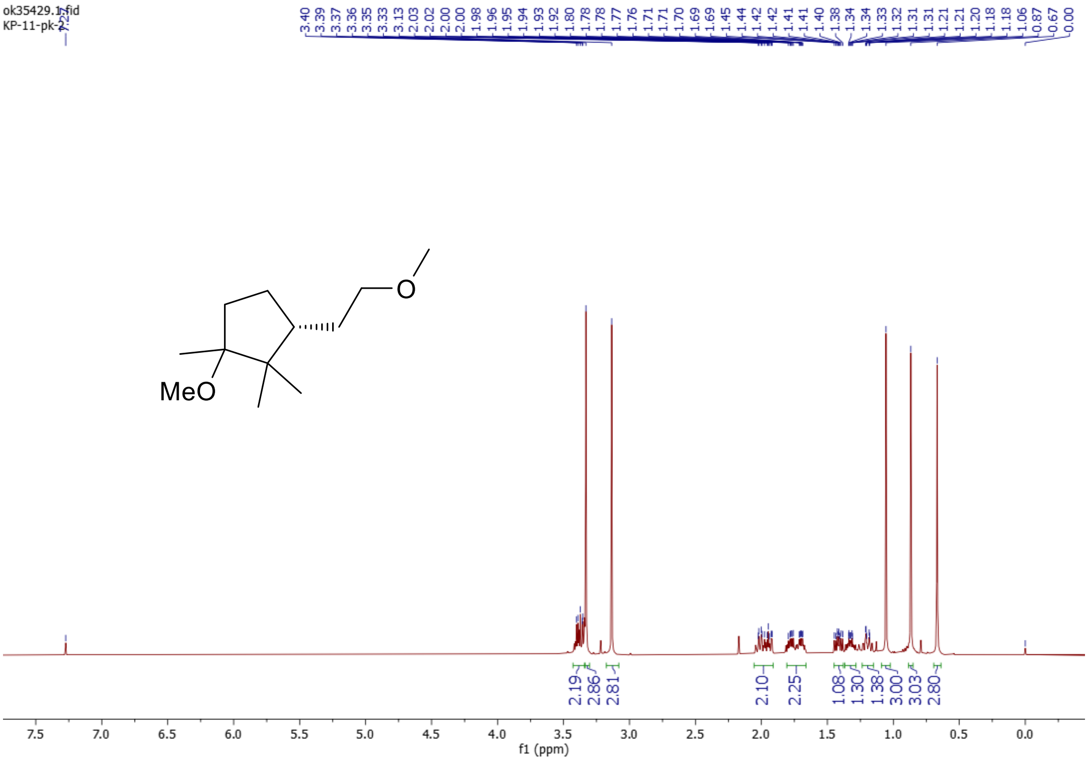

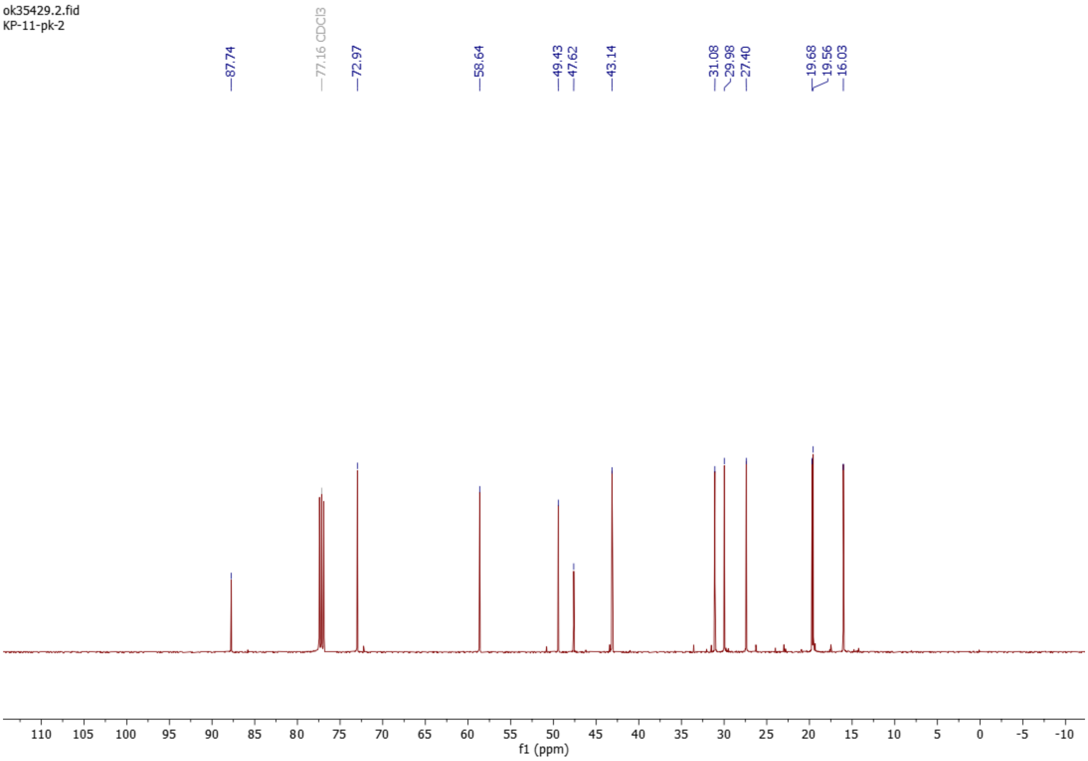

(3a*R*,6a*S*)-6,6,6a-Trimethylhexahydro-2*H*-cyclopenta[*b*]furan (21)

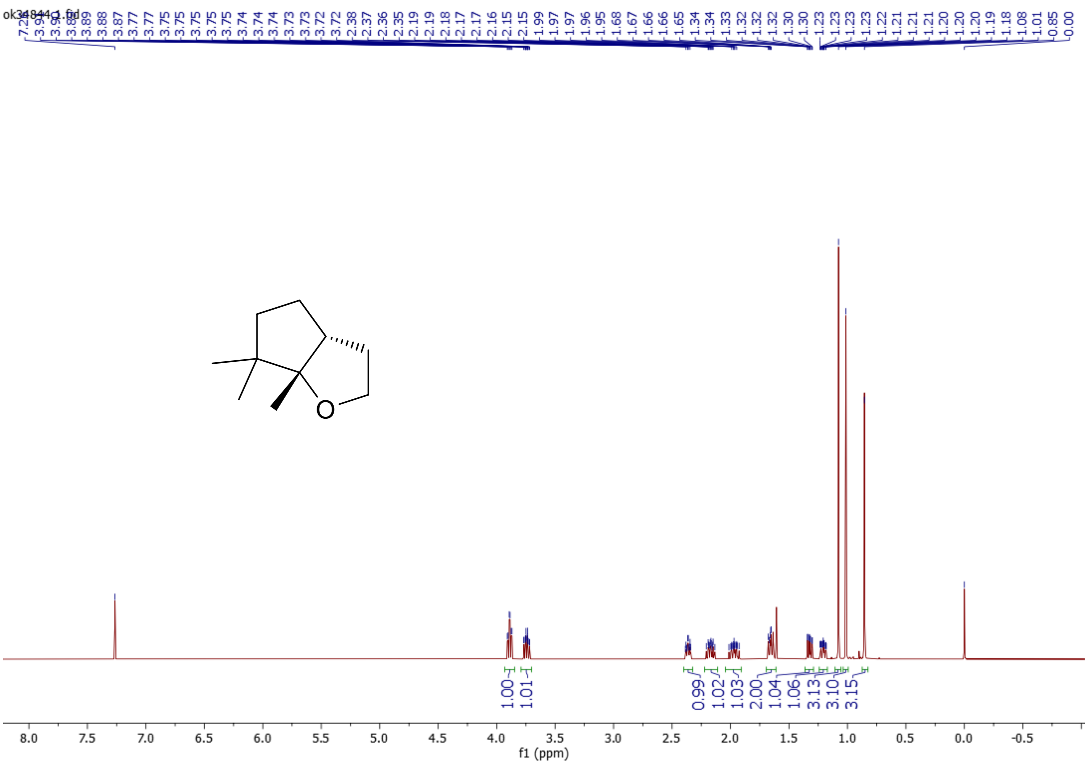

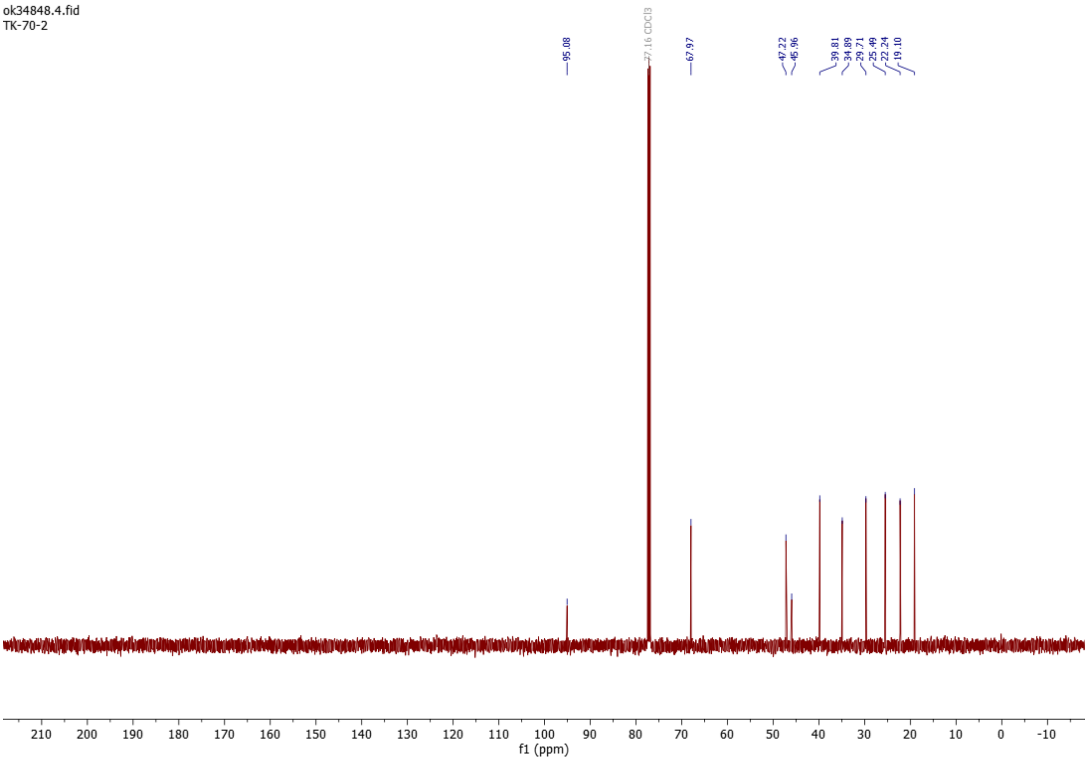

3. DFT calculation

Theoretical studies were carried out using AMS 2025.1, SCM, Theoretical Chemistry program.[11] Geometry optimizations of relevant equilibrium structures were performed using the M06-2X[12,13] density functional with the TZ2P basis set as implemented in ADF–AMS 2025.1. Normal mode vibrational analysis on the stationary points allowed us to confirm they are minima (zero imaginary frequencies). The M06-2X functional was chosen as it is better suited to handling kinetics, thermodynamics, and noncovalent interactions in organic molecular systems.[12,14,15,16,17,18,19] Solvation effect (in dichloromethane) was considered using COSMO model. An ultrafine grid was employed for all DFT calculations. Unless otherwise stated, relative Gibbs energies (ΔG) reported correspond to the M06-2X/TZ2P level at 298.13 K in dichloromethane. The 3D geometries were generated using GUI of AMS 2025.1 (Figure S1).

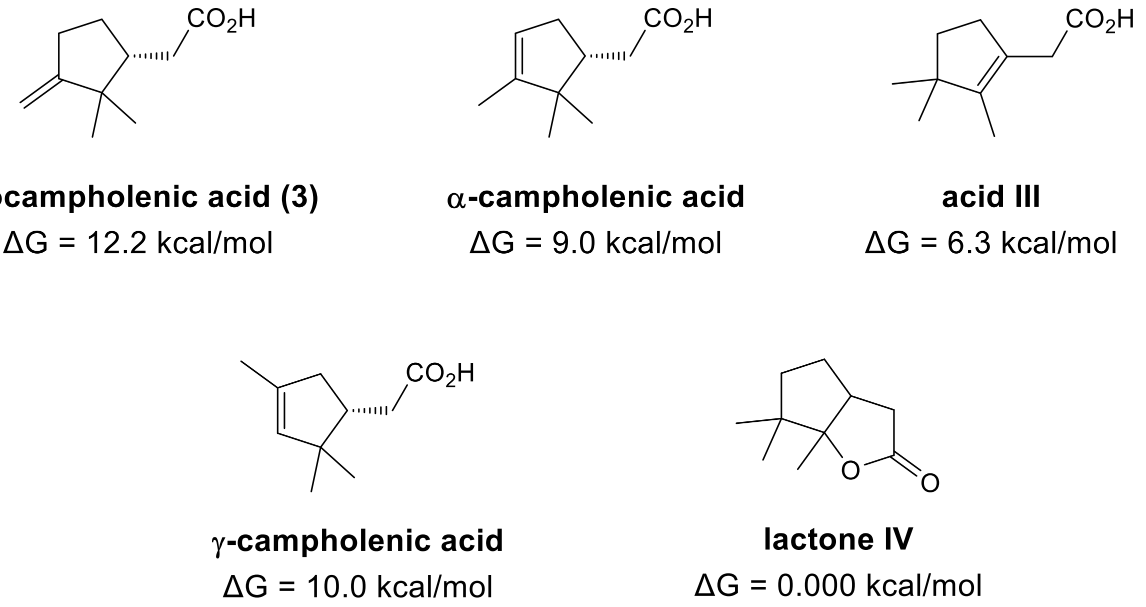

Figure S1. Relative free energies (relative to the energy of lactone **IV**) of the isomeric alkenes.

isocampholenic acid (3) Gibbs energy at 298.15 K (-5016.138099) kcal/mol

ΔG 12.2 kcal/mol

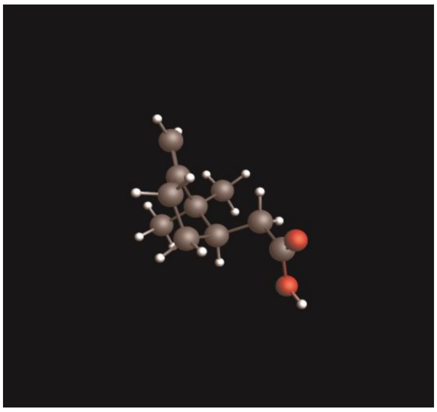

α-campholenic acid Gibbs energy at 298.15 K (-5019.345727) kcal/mol

ΔG 9.0 kcal/mol

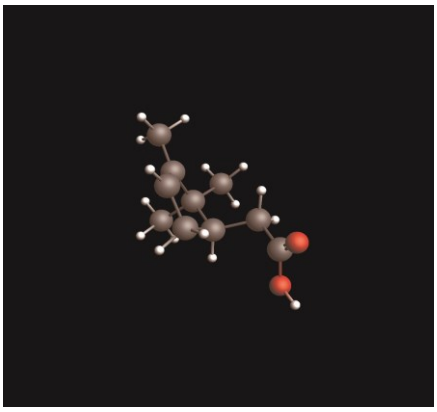

acid III Gibbs energy at 298.15 K (-5022.010891)kcal/mol

ΔG 6.3 kcal/mol

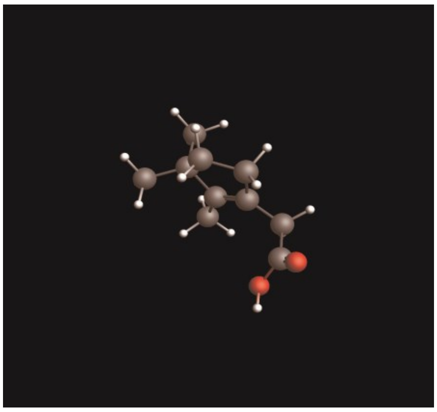

γ-campholenic acid Gibbs energy at 298.15 K (-5018.307839) kcal/mol

ΔG 10.0 kcal/mol

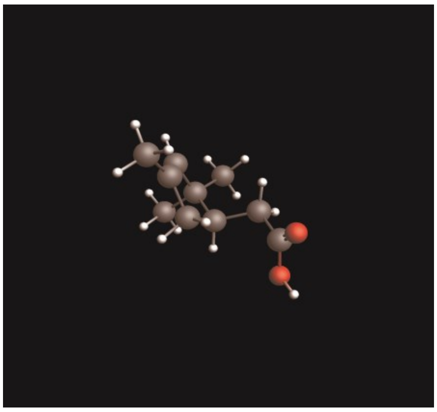

lactone IV Gibbs energy at 298.15 K (-5028.292983) kcal/mol

ΔG 0.000 kcal/mol

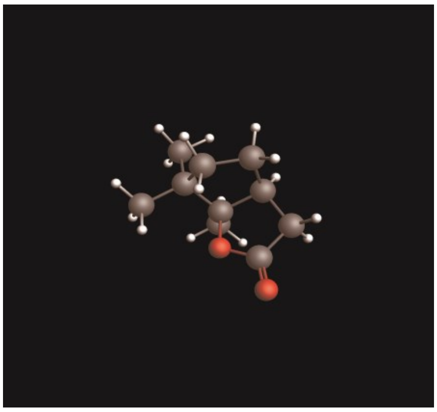

isocampholenic acid (3)

C	C(1)	0.315612	-0.82917	0.214009
C	C(2)	-0.49096	-0.76463	1.522116
C	C(3)	-0.48953	0.083448	-0.74725
C	C(4)	-0.93555	1.209156	0.174548
C	C(5)	-0.96789	0.69669	1.604499
H	H(6)	0.092553	-1.058	2.394428
H	H(7)	-1.3393	-1.44386	1.467159
C	C(8)	1.745182	-0.30262	0.421105
C	C(9)	-1.73665	-0.67428	-1.23669
C	C(10)	0.298968	0.557893	-1.96297

C	C(11)	-1.23015	2.444986	-0.20331
H	H(12)	-1.95954	0.78622	2.046049
C	C(13)	2.477549	-1.08074	1.474053
O	O(14)	2.894809	-0.63388	2.514676
O	O(15)	2.616797	-2.37283	1.149767
H	H(16)	0.372188	-1.84479	-0.17872
H	H(17)	1.737605	0.742262	0.729537
H	H(18)	2.313453	-0.38659	-0.50527
H	H(19)	-1.44089	-1.55102	-1.81566
H	H(20)	-2.33577	-0.02338	-1.87357
H	H(21)	-2.36641	-1.00469	-0.41155
H	H(22)	1.128307	1.206496	-1.68377
H	H(23)	-0.3474	1.117137	-2.63975
H	H(24)	0.694182	-0.29686	-2.51526
H	H(25)	-1.15894	2.755935	-1.23803
H	H(26)	-1.55528	3.189363	0.513206
H	H(27)	3.088905	-2.82194	1.867221
H	H(28)	-0.29247	1.292799	2.220003

α-campholenic acid

C	C(1)	0.293058	-0.83081	0.260144
C	C(2)	-0.42173	-0.59948	1.611234
C	C(3)	-0.4845	0.076879	-0.73741
C	C(4)	-1.02434	1.169763	0.178196
C	C(5)	-1.00323	0.781648	1.448716
H	H(6)	0.256716	-0.67536	2.463364
H	H(7)	-1.21219	-1.3364	1.776083
C	C(8)	1.774735	-0.44275	0.371837
C	C(9)	-1.66899	-0.71291	-1.31636
C	C(10)	0.353631	0.625794	-1.88972
C	C(11)	-1.56077	2.451829	-0.37007
H	H(12)	-1.39078	1.360959	2.277095
C	C(13)	2.501651	-1.30945	1.358072

O	O(14)	3.025979	-0.92796	2.376582
O	O(15)	2.505926	-2.6002	0.999254
H	H(16)	0.23524	-1.87251	-0.05559
H	H(17)	1.880167	0.590008	0.699336
H	H(18)	2.271826	-0.56004	-0.59129
H	H(19)	-1.31203	-1.49362	-1.99018
H	H(20)	-2.33336	-0.05724	-1.88098
H	H(21)	-2.24963	-1.18237	-0.5212
H	H(22)	1.148239	1.286626	-1.54278
H	H(23)	-0.27869	1.19582	-2.5719
H	H(24)	0.805597	-0.18755	-2.46007
H	H(25)	-0.773	3.025652	-0.8621
H	H(26)	-1.9928	3.066137	0.4178
H	H(27)	-2.33076	2.263386	-1.12181
H	H(28)	2.984035	-3.10585	1.673738

acid III

C	C(1)	0.694077	0.050477	0.27972
C	C(2)	0.725382	1.108649	1.356804
C	C(3)	-0.44496	0.073183	-0.41112
C	C(4)	-1.35483	1.19936	0.067216
C	C(5)	-0.74509	1.542094	1.443543
H	H(6)	1.38419	1.929421	1.055487
H	H(7)	1.110419	0.727993	2.303968
C	C(8)	1.874917	-0.85992	0.06554
C	C(9)	-0.84859	-0.79826	-1.55477
C	C(10)	-2.81665	0.773795	0.176486
H	H(11)	-1.24637	0.94748	2.209355
C	C(12)	1.847311	-1.94473	1.110135
O	O(13)	2.360716	-1.87238	2.199795
O	O(14)	1.134644	-3.00811	0.722263
H	H(15)	2.802991	-0.30514	0.193309
H	H(16)	1.855331	-1.31319	-0.92196

H	H(17)	-0.04356	-1.45398	-1.87983
H	H(18)	-1.16813	-0.19574	-2.4079
H	H(19)	-1.69752	-1.42554	-1.2749
H	H(20)	-3.40811	1.572935	0.62691
H	H(21)	-2.91772	-0.11734	0.798066
H	H(22)	-3.24068	0.556917	-0.80557
H	H(23)	1.114049	-3.64755	1.450475
C	C(24)	-1.23353	2.391095	-0.89215
H	H(25)	-0.873	2.593893	1.699056
H	H(26)	-1.88332	3.205663	-0.56734
H	H(27)	-1.52445	2.113063	-1.90644
H	H(28)	-0.20725	2.760812	-0.92146

γ-campholenic acid

C	C(1)	0.284443	-0.82556	0.265443
C	C(2)	-0.42482	-0.56794	1.612515
C	C(3)	-0.48272	0.082904	-0.7448
C	C(4)	-1.01827	1.167395	0.166039
C	C(5)	-1.00952	0.817116	1.447187
H	H(6)	0.252319	-0.62888	2.468392
H	H(7)	-1.22419	-1.29111	1.796989
C	C(8)	1.771978	-0.45865	0.37334
C	C(9)	-1.65793	-0.70658	-1.34122
C	C(10)	0.366437	0.63982	-1.88584
C	C(11)	-1.52976	1.585191	2.616441
C	C(12)	2.485731	-1.32842	1.366579
O	O(13)	3.008132	-0.94822	2.386353
O	O(14)	2.480456	-2.62032	1.011997
H	H(15)	0.211466	-1.86971	-0.03862
H	H(16)	1.892235	0.575085	0.693083
H	H(17)	2.266083	-0.59064	-0.58927
H	H(18)	-1.29426	-1.49089	-2.00781
H	H(19)	-2.31045	-0.04636	-1.9144

H	H(20)	-2.2531	-1.17109	-0.55366
H	H(21)	1.16778	1.283519	-1.52389
H	H(22)	-0.2613	1.233208	-2.55223
H	H(23)	0.808408	-0.16589	-2.47522
H	H(24)	2.950434	-3.1281	1.690554
H	H(25)	-1.42313	2.095027	-0.22297
H	H(26)	-0.73999	1.749154	3.35229
H	H(27)	-2.31946	1.024405	3.1214
H	H(28)	-1.93098	2.550784	2.314055

lactone IV

C	C(1)	1.084057	0.532134	-0.10707
C	C(2)	0.791003	1.670864	0.899525
C	C(3)	-0.2689	-0.2007	-0.30849
C	C(4)	-1.35545	0.771948	0.190935
C	C(5)	-0.64789	1.435608	1.382524
H	H(6)	0.870014	2.634318	0.398747
H	H(7)	1.505582	1.677745	1.720882
C	C(8)	1.993402	-0.56638	0.429387
C	C(9)	-0.47192	-0.79915	-1.68381
C	C(10)	-2.65287	0.07947	0.590543
H	H(11)	-0.65759	0.751794	2.233422
C	C(12)	1.038567	-1.54577	1.065914
O	O(13)	1.288241	-2.43574	1.838374
O	O(14)	-0.20525	-1.31231	0.640976
H	H(15)	2.736124	-0.23591	1.150447
H	H(16)	2.515043	-1.10113	-0.3661
H	H(17)	0.32924	-1.50471	-1.90597
H	H(18)	-0.46034	-0.02157	-2.44463
H	H(19)	-1.42148	-1.33139	-1.73852
H	H(20)	-3.36718	0.821239	0.950838
H	H(21)	-2.48653	-0.648	1.382919
H	H(22)	-3.10742	-0.43181	-0.2598

C	C(23)	-1.6461	1.813384	-0.89525
H	H(24)	-1.14735	2.353332	1.691202
H	H(25)	-2.26968	2.605036	-0.47922
H	H(26)	-2.18379	1.368116	-1.73226
H	H(27)	-0.73538	2.271159	-1.2844
H	H(28)	1.45206	0.923681	-1.05165

4. Olfactory properties and chemical shifts

Of the 57 synthesized isocampholenic acid derivatives (compounds **3–16**) that contain an intact methylene group, 29 isomers derived from **α-campholenic acid** and 13 isomers of the type **III** isomer were found in the literature (CAS SciFinder®, including patent literature). Only the isomers (isomers derived from **α-campholenic acid** and type **III** isomer) for which reliable proton NMR data in deuterated chloroform are available are shown in Figure S1. Thus, for the **isocampholenic acid** derivatives and the isomers derived from **α-campholenic acid**,[20,21,22,23,24,25,26] the vinylic protons are shown, while for the type **III** isomer[27,28] the Me-group signals are shown. References are included in Figure S2.

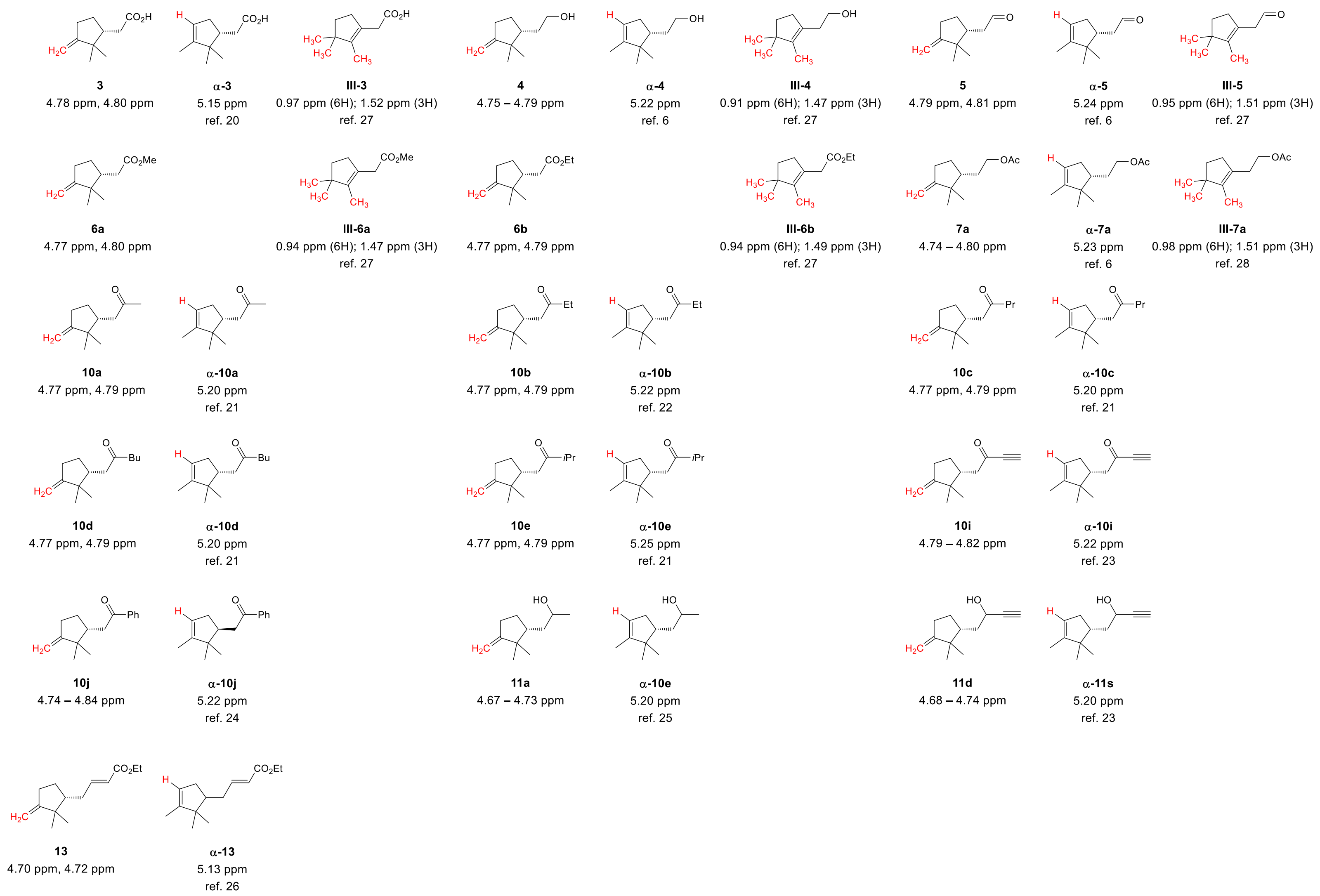

Figure S2. Typical proton chemical shifts of three types of isomeric compounds measured in $CDCl_3$. Multiplicities and coupling constants are omitted for clarity.

The odor profiles of the selected products were tentatively determined by an untrained panel of five laypersons, with the average description shown in Table S1. The amounts of α-campholenic acid-derived isomer impurities or, in the case of cyclopropanated products **17–19**, the unreacted alkenes, are included in Table 1 as determined by proton NMR. The samples were evaluated as neat oils. No dilution experiments or trashold determinations were performed, i.e. methods combining instrumental analysis (GC-MS) with human olfactory perception. The average odor profiles determined are therefore only a rough description.

Table S1. The odor profiles of the selected products.

	R = 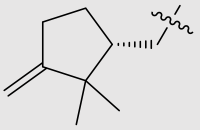	**Impurities[a] [%]**	**Average odor profile**
1	**6a** 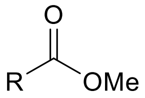	0 [b]	woody scent, green note "peppermint"
2	**6b** 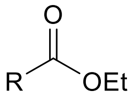	0 [b]	smell of wood smell of herbs
3	**6c** 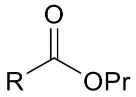	1 [b]	faint smell of wood
4	**6d** 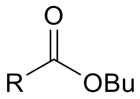	1 [b]	smell of the sea
5	**6e** 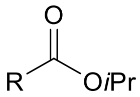	8 [b]	floral scent weak fruity scent
6	**6f** 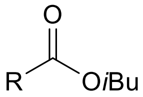	0 [b]	faint smell of wood
7	**6g** 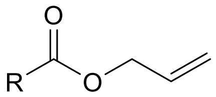	0 [b]	smell of the forest "spruce tops"
8	**6h** 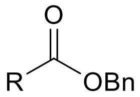	12 [b]	fruity scents "melon, mango"
9	**7a** 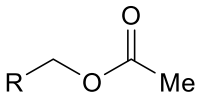	1 [b]	fruity scent
10	**7b** 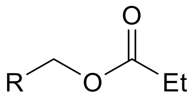	1 [b]	fruity scent

11	**7c** 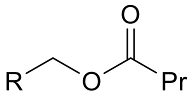	1 [b]	smell of milk
12	**7d** 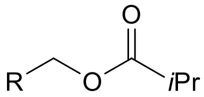	1 [b]	sweet smell<br>fruity smell, "pear"
13	**7g** 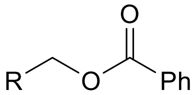	1 [b]	fruity scent<br>"grape"
14	**8a** 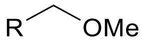	0 [b]	smell of wood
15	**8b** 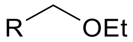	1 [b]	smell of wood
16	**8c** 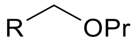	0 [b]	faint smell of wood
17	**8d** 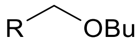	0 [b]	faint smell of wood
18	**8e** 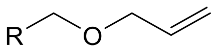	0 [b]	green note<br>herbal scent
19	**8h** 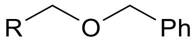	0 [b]	faint smell of wood
20	**10a** 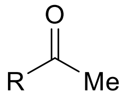	3 [b]	smell of wood<br>smell of the forest
21	**10b** 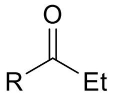	1 [b]	smell of the forest
22	**10c** 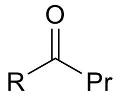	0 [b]	sweet smell<br>"rum"
23	**10d** 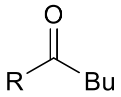	0 [b]	fresh smell<br>sweet smell
24	**10e** 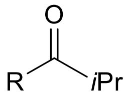	2 [b]	"sharp" smell
25	**10f** 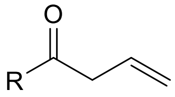	0 [b]	sweet smell<br>"sharp" smell
26	**10g** 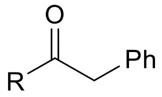	0 [b]	sweet smell
27	**10h** 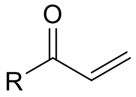	0 [b]	"sharp" smell

28	**10k** 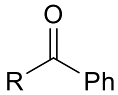	0 [b]	sweet smell<br>"cinnamon, cloves"<br>"sharp" smell
29	**11a** 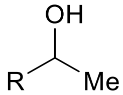	0 [b]	sweet smell<br>faint herbal smell
30	**11b** 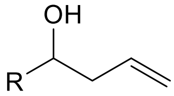	0 [b]	fresh mint<br>menthol scent
31	**11c/11c'** (*dr* = 53:47) 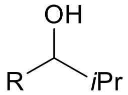	0 [b]	fresh mint scent
32	**11d** 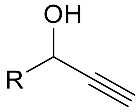	0 [b]	slightly fresh mint scent<br>partly unpleasant goat's milk scent
33	**12a** 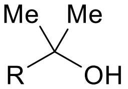	1 [b]	smell of wood<br>smell of the forest<br>"honey"
34	**12b/12b'** (*dr* = 60:40) 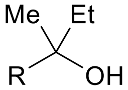	0 [b]	smell of the forest
35	**12c/12c'** (*dr* = 60:40) 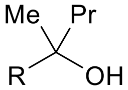	0 [b]	smell of the forest<br>"conifers"
36	**12d/12d'** (*dr* = 59:41) 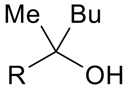	1 [b]	smell of the forest
37	**12f/12f'** (*dr* = 57:43) 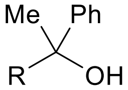	0 [b]	pleasant smell
38	**13** 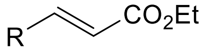	0 [b]	very sweet smell<br>like an "energy drink"
39	**14** 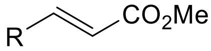	4 [b]	herbal scent of thyme and sage
40	**15** 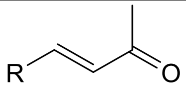	2 [b]	pleasant smell of spruce resin

41	**16/16'** *E*:*Z* = 88:12 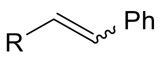	2 [b]	a faint smell of horseradish mixed with the smell of synthetic rubber or plastic
	R[1] = 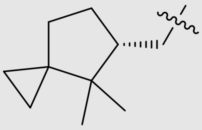	**Impurities[a] [%]**	**Average odor profile**
42	**17** 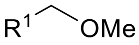	8 of **8a** [c]	fresh and fruity scent, slightly artificial blackberry scent
43	**18** 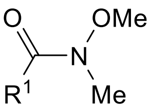	5 of **9** [c]	fresh mint scent, mixed with the sweet scent of chocolate
44	**19a** 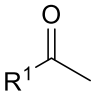	4 of **9** [c]	scent of conifers, spruce tops, slightly fruity after "artificial strawberry scent"
45	**19b** 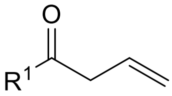	3 of **9** [c]	smell of camphor cut grass, parsley
46	**19c** 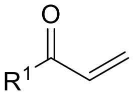	0 of **9** [c]	smell of camphor, conifers and eucalyptus
47	**19d** 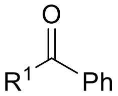	3 of **9** [c]	herbal scent of wormwood and parsley
48	**21** 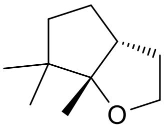	-	peppermint

[a] Determined by ^{1}H-NMR in $CDCl_3$. [b] α-Campholene isomer as impurity. [c] Alkene as impurity.

5. References

[1] Cossio, F.P.; Lopez, M.C.; Palomo, C. Pyridine assisted oxidations of alcohols to carbonyl compounds by means of 3-carboxypyridinium dichromate (ndc) reagent, *Tetrahedron* **1987**, *43*, 3963–3974. https://doi.org/10.1016/S0040-4020(01)81678-1

[2] López, C.; González, A.; Cossío, F.P.; Palomo, C. 3-Carboxypyridinium Dichromate (NDC) and (4-Carboxypyridinium Dichromate (INDC). Two New Mild, Stable, Efficient, and Inexpensive Chromium (VI) Oxidation Reagents, *Synth. Commun.* **1985**, *15*, 1197–1211. https://doi.org/10.1080/00397918508077265

[3] Ričko, S.; Svete, J.; Štefane, B.; Perdih, A.; Golobič, A.; Meden, A.; Grošelj, U. 1,3-Diamine-Derived Bifunctional Organocatalyst Prepared from Camphor, *Adv. Synth. Catal.* **2016**, *358*, 3786–3796. https://doi.org/10.1002/adsc.201600498

[4] Grošelj, U.; Golobič, A.; Knez, D.; Hrast, M.; Gobec, S.; Ričko, S.; Svete, J. Synthesis and preliminary biological evaluations of (+)-isocampholenic acid-derived amides, *Mol. Divers.* **2016**, *20*, 667–676. https://doi.org/10.1007/s11030-016-9668-9

[5] Kagawa, M. Action of Alkaline Substances on 10-Bromocamphor. I, *Pharm. Bull.* **1956**, *4*, 423–427. https://doi.org/10.1248/cpb1953.4.423

[6] Chapuis, C.; Brauchli, R. Preparation of Campholenal Analogues: Chirons for the lipophilic moiety of sandalwood-like odorant alcohols, *Helv. Chim. Acta* **1992**, *75*, 1527–1546. https://doi.org/10.1002/hlca.19920750507

[7] Yong, W.; Ling, S.; D'Halleweyn, C.; Van Haver, D.; De Clercq, P.; Vandewalle, M.; Bouillon, R.; Verstuyf, A. Synthesis of CD-ring modified 1α,25-dihydroxy vitamin D analogues: Five-membered D-ring analogues, *Bioorg. Med. Chem. Lett.* **1997**, *7*, 923–928. https://doi.org/10.1016/S0960-894X(97)00137-6

[8] Spallek, M.J.; Storch, G.; Trapp, O. Straightforward Synthesis of Poly(dimethylsiloxane) Phases with Immobilized (1R)-3-(Perfluoroalkanoyl)camphorate Metal Complexes and Their Application in Enantioselective Complexation Gas Chromatography, *Eur. J. Org. Chem.* **2012**, *2012*, 3929–3945. https://doi.org/10.1002/ejoc.201200075

[9] Rüedi, G.; Hansen, H.-J. Thermal Isomerization of Isoborneols and Dehydroisoborneols to New Chiral Building Blocks in Terpenoid Synthesis, *Helv. Chim. Acta* **2004**, *87*, 1968–1989. https://doi.org/10.1002/hlca.200490179

[10] De Pascual, T.J.; Bellido, S.I.; Benito, G.M. Rearrangement of (+)-2-hydroxypinacamphone in anhydrous acidic medium. Absolute configuration of (+)-dihydro-β-campholenolactone, *An. Quím.* **1973**, *69*, 217–228.

[11] AMS 2025.1, SCM, Theoretical Chemistry, Vrije Universiteit, Amsterdam, The Netherlands, http://www.scm.com. List of authors and contributors: R. Rüger, M. Franchini, T. Trnka, A. Yakovlev, E. van Lenthe, P. Philipsen, T. van Vuren, B. Klumpers, T. Soini.

[12] Zhao, Y.; Truhlar, D.G. The M06 suite of density functionals for main group thermochemistry, thermochemical kinetics, noncovalent interactions, excited states, and transition elements: two new functionals and systematic testing of four M06-class functionals and 12 other functionals. *Theor. Chem. Acc.* **2008**, *120*, 215–241. https://doi.org/10.1007/s00214-007-0310-x

[13] Zhao, Y.; Truhlar, D.G. Density Functionals with Broad Applicability in Chemistry. *Acc. Chem. Res.* **2008**, *41*, 157–167. https://doi.org/10.1021/ar700111a

[14] Rokob, T.A.; Hamza, A.; Pápai, I. Computing Reliable Energetics for Conjugate Addition Reactions. *Org. Lett.* **2007**, *9*, 4279–4282. https://doi.org/10.1021/ol701872z

[15] Yang, H.; Wong, M.W. β-Amino Acid Catalyzed Asymmetric Michael Additions: Design of Organocatalysts with Catalytic Acid/Base Dyad Inspired by Serine Proteases. *J. Org. Chem.* **2011**, *76*, 7399–7405. https://doi.org/10.1021/jo2011413

[16] Cho, B.; Tan, C.-H.; Wong, M.W. Origin of Asymmetric Induction in Bicyclic Guanidine-Catalyzed Thio-Michael Reaction: A Bifunctional Mode of Lewis Acid-Brønsted Acid Activation. *J. Org. Chem.* **2012**, *77*, 6553–6562. https://doi.org/10.1021/jo301158c

[17] Yang, H.; Wong, M.W. Oxyanion Hole Stabilization by C–H···O Interaction in a Transition State–A Three-Point Interaction Model for Cinchona Alkaloid-Catalyzed Asymmetric Methanolysis of meso-Cyclic Anhydrides. *J. Am. Chem. Soc.* **2013**, *135*, 5808–5818. https://doi.org/10.1021/ja4005893

[18] Wong, M.W.; Ng, A.M.E. Asymmetric Michael Addition Using Bifunctional Bicyclic Guanidine Organocatalyst: A Theoretical Perspective. *Aust. J. Chem.* **2014**, *67*, 1100–1109. https://doi.org/10.1071/CH14340

[19] Xue, H.; Jiang, D.; Jiang, H.; Kee, C.W.; Hirao, H.; Nishimura, T.; Wong, M.W.; Tan, C.-H. Mechanistic Insights into Bicyclic Guanidine-Catalyzed Reactions from Microscopic and Macroscopic Perspectives. *J. Org. Chem.* **2015**, *80*, 5745–5752. https://doi.org/10.1021/acs.joc.5b00709

[20] Crist, B.V.; Rodgers, S.L.; Lightner, D.A. Chair-boat conformational equilibrium in (+)-(1*S*,5*R*)-1,8,8-trimethylbicyclo[3.2.1]octan-3-one. *J. Am. Chem. Soc.* **1982**, *104*, 6040–6045. https://doi.org/10.1021/ja00386a033

[21] Siewiński, A.; Dmochowska-Gładysz, J.; Kołek, T.; Zabża, A.; Derdziński, K.; Nespiak, A. Microbiological transformations-XV: Microbiological transformation of 1-(2,2,3-trimethylcyclo-pent-3-en-yl)-propan-2-one and their homologues by acremonium roseum s.s. gams 1971. *Tetrahedron* **1983**, *39*, 2265–2270. https://doi.org/10.1016/S0040-4020(01)91951-9

[22] Ondet, P.; Filippi, J.-J.; Lemière, G.; Duñach, E. Stereocontrolled Cascade Cyclisation of Campholenic Enol Ether Derivatives: En Route to Vetiver-Scented Spiroxides. *Eur. J. Org. Chem.* **2018**, *2018*, 980–989. https://doi.org/10.1002/ejoc.201701578

[23] Oakdale, J.S.; Sit, R.K.; Fokin, V.V. Ruthenium-Catalyzed Cycloadditions of 1-Haloalkynes with Nitrile Oxides and Organic Azides: Synthesis of 4-Haloisoxazoles and 5-Halotriazoles. *Chem. Eur. J.* **2014**, *20*, 11101–11110. https://doi.org/10.1002/chem.201402559

[24] Battersby, A.R.; Laing, D.G.; Ramage, R. Biosynthesis. Part XIX. Concerning the biosynthesis of (–)-camphor and (–)-borneol in Salvia officinalis. *J. Chem. Soc., Perkin Trans. 1* **1972**, 2743–2748. https://doi.org/10.1039/P19720002743

[25] Siewiński, A.; Dmochowska-Gładysz, J.; Kołek, T.; Zabża, A.; Derdziński, K. Microbiological transformations—XII: Microbiological reduction of racemic 1-(2',2',3'-trimethyl-cyclopent-3'-en-1'-yl)propan-2-one and 1-(2',2',3'-trimethyl-cyclopent-3'-en-1'-yl)butan-2-one by *rhodotorula mucilaginosa*. *Tetrahedron* **1979**, *35*, 1409–1414. https://doi.org/10.1016/0040-4020(79)85035-8

[26] Ungureanu, I.M.; Brune, N.E.I.; Slack, J.P.; Gray K.;, Simons, C.T.; Evans J.E. Pennimpede, Off-note blocking sensory organic compounds. US8409649, 2013.

[27] Schulze, K.; Wyßuwa, K.; Trauer, H.; Habermann, A.-K. Synthese und Reaktionen von β-Campholenverbindungen. *J. Prakt. Chem.* **1993**, *335*, 537–543. https://doi.org/10.1002/prac.19933350607

[28] Lewis, J.B.; Hedrick, G.W. Reaction of α-Pinene Oxide with Zinc Bromide and Rearrangement of 2,2,3-Trimethyl-3-cyclopentene Products Derived Therefrom1. *J. Org. Chem.* **1965**, *30*, 4271–4275. https://doi.org/10.1021/jo01023a064
